# Identification of *Drosophila* Mitotic Genes by Combining Co-Expression Analysis and RNA Interference

**DOI:** 10.1371/journal.pgen.1000126

**Published:** 2008-07-18

**Authors:** Maria Patrizia Somma, Francesca Ceprani, Elisabetta Bucciarelli, Valeria Naim, Valeria De Arcangelis, Roberto Piergentili, Antonella Palena, Laura Ciapponi, Maria Grazia Giansanti, Claudia Pellacani, Romano Petrucci, Giovanni Cenci, Fiammetta Vernì, Barbara Fasulo, Michael L. Goldberg, Ferdinando Di Cunto, Maurizio Gatti

**Affiliations:** 1 Dipartimento di Genetica e Biologia Molecolare, Istituto di Biologia e Patologia Molecolari del CNR, Rome, Italy; 2 Dipartimento di Biologia di Base ed Applicata, Università dell'Aquila, L'Aquila, Italy; 3 Department of Molecular Biology and Genetics, Cornell University, Ithaca, New York, United States of America; 4 Molecular Biotechnology Center, Università di Torino, Torino, Italy; Stowers Institute for Medical Research, United States of America

## Abstract

RNAi screens have, to date, identified many genes required for mitotic divisions of *Drosophila* tissue culture cells. However, the inventory of such genes remains incomplete. We have combined the powers of bioinformatics and RNAi technology to detect novel mitotic genes. We found that *Drosophila* genes involved in mitosis tend to be transcriptionally co-expressed. We thus constructed a co-expression–based list of 1,000 genes that are highly enriched in mitotic functions, and we performed RNAi for each of these genes. By limiting the number of genes to be examined, we were able to perform a very detailed phenotypic analysis of RNAi cells. We examined dsRNA-treated cells for possible abnormalities in both chromosome structure and spindle organization. This analysis allowed the identification of 142 mitotic genes, which were subdivided into 18 phenoclusters. Seventy of these genes have not previously been associated with mitotic defects; 30 of them are required for spindle assembly and/or chromosome segregation, and 40 are required to prevent spontaneous chromosome breakage. We note that the latter type of genes has never been detected in previous RNAi screens in any system. Finally, we found that RNAi against genes encoding kinetochore components or highly conserved splicing factors results in identical defects in chromosome segregation, highlighting an unanticipated role of splicing factors in centromere function. These findings indicate that our co-expression–based method for the detection of mitotic functions works remarkably well. We can foresee that elaboration of co-expression lists using genes in the same phenocluster will provide many candidate genes for small-scale RNAi screens aimed at completing the inventory of mitotic proteins.

## Author Summary

Mitosis is the evolutionarily conserved process that enables a dividing cell to equally partition its genetic material between the two daughter cells. The fidelity of mitotic division is crucial for normal development of multicellular organisms and to prevent cancer or birth defects. Understanding the molecular mechanisms of mitosis requires the identification of genes involved in this process. Previous studies have shown that such genes can be readily identified by RNA interference (RNAi) in *Drosophila* tissue culture cells. Because the inventory of mitotic genes is still incomplete, we have undertaken an RNAi screen using a novel approach. We used a co-expression–based bioinformatic procedure to select a group of 1,000 genes enriched in mitotic functions from a dataset of 13,166 *Drosophila* genes. This group includes roughly half of the known mitotic genes, implying that it should contain half of all mitotic genes, including those that are currently unknown. We performed RNAi against each of the 1,000 genes in the group. By limiting the number of genes to be examined, we were able to perform a very detailed phenotypic analysis of RNAi cells. This analysis allowed the identification of 70 genes whose mitotic role was previously unknown; 30 are required for proper chromosome segregation and 40 are required to maintain chromosome integrity.

## Introduction

RNA interference (RNAi) in *Drosophila* cell cultures is a powerful tool for the identification of proteins involved in mitotic cell division. The addition of a double stranded RNA (dsRNA) to the cell medium leads to rapid downregulation of the corresponding mitotic protein, resulting in a specific and penetrant phenotype [1]–[10]. Identification of mitotic genes/proteins by RNAi has thus far relied on two general approaches. The first involved genome-wide screens to detect gross changes in cell and nuclear morphology [3],[6],[8], defects in cytokinesis [7],[8] or in spindle and centrosome structure [10]. Most of these screens were performed using automated microscopy [3],[6],[8] or the visual analysis of a very simple phenotype [7]. In a second approach, RNAi experiments were performed on selected gene groups, such as those encoding kinesins, actin-binding proteins, kinases or phosphatases [2],[4],[5],[9]. Cells depleted for these proteins were examined by standard fluorescence microscopy that allowed detection of a wide spectrum of mitotic abnormalities.

Although genome-wide and gene-specific approaches have identified many mitotic functions, the inventory of such proteins is likely to be largely incomplete. For example, RNAi has never been used to detect genes involved in establishing proper mitotic chromosome morphology or required to maintain mitotic chromosome integrity. We present here a novel approach for the identification of mitotic proteins by RNAi. Using a co-expression-based bioinformatic procedure, we generated a list of 1000 *Drosophila* genes highly enriched in mitotic functions. We then performed RNAi experiments for each of these 1000 genes, and examined both mitotic chromosome structure and spindle morphology in the treated cells. This screen has led to the identification of 142 mitotic genes, 70 of which have not been previously implicated in mitosis.

## Results

### Construction of a Mitotic-Gene-Enriched Co-Expression List

Numerous studies indicate that genes involved in the same biological process tend to be transcriptionally co-expressed (see, for example,[11]–[13]. We thus exploited extant microarray data [14] to rank the complete set of annotated *Drosophila* genes according to their co-expression with six well-characterized genes representative of different aspects of mitosis: *gluon* (*glu*) encodes a condensin [15]; *ida/APC5*, specifies a subunit of the anaphase promoting complex (APC/C [16]); *cid/CenpA* is the gene for the centromere-specific histone H3 variant required for kinetochore assembly [17],[18]; *Eb1* encodes a microtubule (MT)-associated protein required for spindle assembly [19]; *zw10* specifies a component of the RZZ complex that helps target cytoplasmic dynein to the kinetochore and that is involved in the spindle checkpoint [20]; and finally *sti* (*Citron kinase*) encodes a serine/threonin protein kinase required for the completion of cytokinesis [21]–[23]. Using the Pearson correlation coefficient, the expression of these prototype genes was correlated with the expression levels of most *Drosophila* genes across 89 different microrray experiments [14]. The 13,166 probesets contained in this dataset were separately ranked for their co-expression with each prototype gene. We then generated a ranked consensus co-expression list by combining the six gene-specific lists (Table S1).

**Figure 1:**
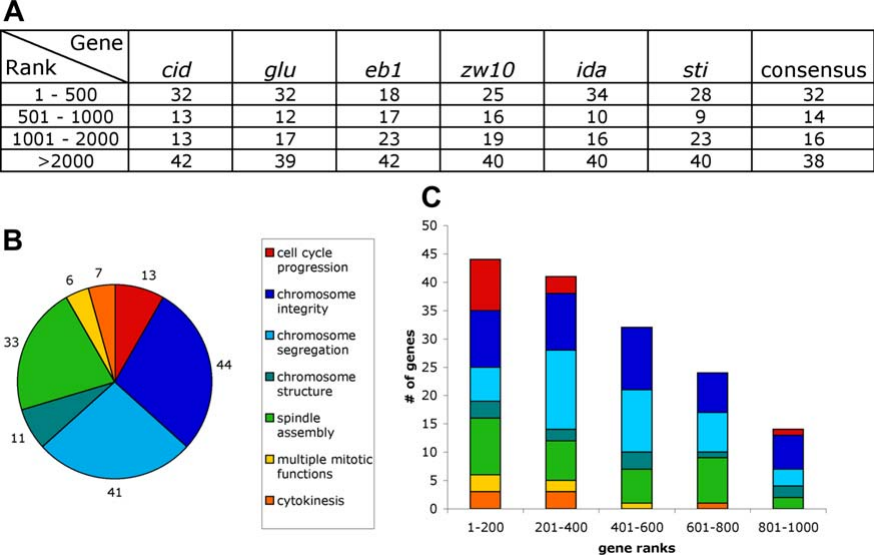
The functions of 155 *Drosophila* genes detected by an RNAi screen of 1000 genes enriched in mitotic functions by co-expression analysis. (A) Distribution of 164 known mitotic genes in co-expression lists with *cid*, *glu*, *eb1*, *zw10*, *ida* and *sti* (*Citron kinase*); ‘consensus’, is a consensus co-expression list constructed by combining the six single gene lists. Numbers in columns are percentages. (B) Quantitative grouping of the 155 genes detected in the screen according to the observed RNAi phenotypes. (C) Distribution of the 155 genes in the consensus co-expression list; note that the frequency of mitotic genes decreases with the increase of the rank in the list.

To validate our bioinformatic approach, we determined the rank in the consensus co-expression lists of 164 *Drosophila* mitotic genes; these genes represent most of the *Drosophila* mitotic genes so far identified but do not include the genes identified in the recent genome-wide screen performed by Goshima et al. [10]. As shown in Figure 1A and Tables S2 and S3, the first 1000 genes of our consensus co-expression list include 46% of the 164 known mitotic genes. This implies that the first 1000 genes of the same list should contain roughly half of all mitotic genes, including those that are currently unknown.

### Identification of Genes Involved in Mitotic Division

To identify new mitotic genes, we synthesized a dsRNA for each of the first 1000 genes in our consensus co-expression list. In designing the primers for such RNAs, we minimized gene overlap to avoid off-target effects of dsRNAs [24],[25] (Table S4). Each dsRNA was then added to S2 cells grown in 3 milliliters (ml) of culture medium. After a 96 h treatment with dsRNA, the cells were split into two aliquots. 2 ml were fixed with formaldehyde and then stained for both tubulin and DNA. The resulting preparations were then blindly scored by at least two independent observers for abnormalities in spindle morphology and chromosome segregation. The remaining 1 ml of cell suspension was incubated with colchicine for two hours, hypotonically swollen, fixed with methanol/acetic acid, and stained with DAPI. The metaphase chromosomes obtained in this way were then blindly examined by at least two observers for abnormalities in chromosome structure and/or the presence of chromosome aberrations. We performed two independent RNAi experiments for each gene. In most of these experiments, we examined 50 colchicine-arrested metaphases and 50 tubulin-stained mitotic figures. If the results of the two experiments were significantly different, we performed additional experiments to define the RNAi phenotype. An RNAi phenotype was considered positive only when the frequency of affected cells was significantly different from controls with p<0.001 (using the χ^2^ contingency test; see Methods).

**Figure 2:**
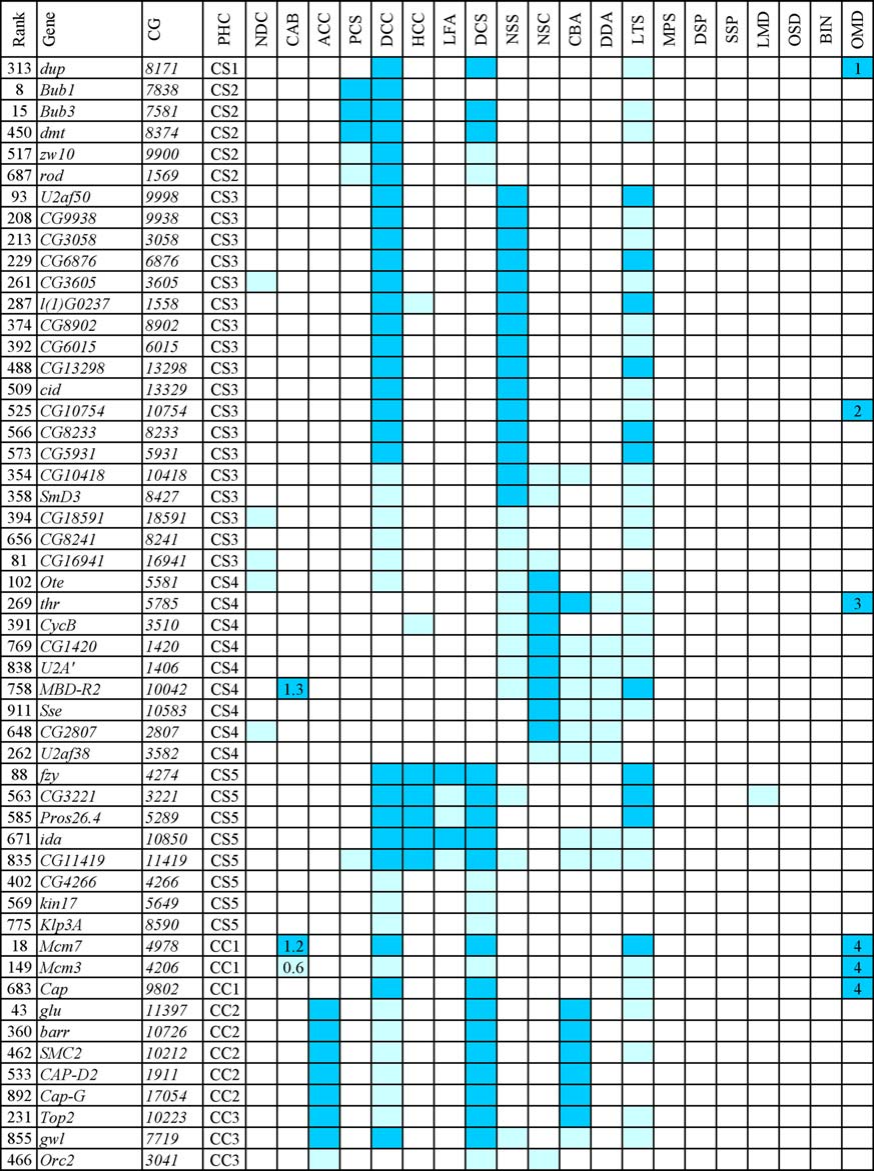
RNAi phenotypes elicited by the genes detected in the screen (CS and CC phenoclusters). Colors refer to the strength of the phenotype: pale blue, weak; blue, strong. The numbers in the CAB column are frequencies of chromosome aberrations per cell. The other numbers refer to relatively rare phenotypic traits. PHC, phenocluster: CS, chromosome segregation; CC, chromosome condensation. Main phenotypic traits: NDC no dividing cells; CAB chromosome aberrations; ACC abnormal chromosome condensation; PCS precocious sister chromatid separation; DCC defective chromosome congression at metaphase; HCC hypercontracted chromosomes; LFA, low relative frequency of anaphases; DCS defective chromosome segregation following sister chromatid separation; NSS no sister chromatid separation with scattered chromosomes; NSC no sister chromatid separation with chromosomes at the center of the cell; CBA chromatin bridges at anaphase; DDA, chromosome decondensation during anatelophase; LTS long anatelophase spindles; MPS monopolar spindles; DSP defective spindle poles (defective asters and/or broad poles); SSP short spindles; LMD, spindles with low MT density; OSD other spindle defects; BIN binucleated cells (cytokinesis failure); OMD, other mitotic defects. Other phenotypic traits: (1) metaphase-like figures contain unreplicated chromosomes; (2) many anaphase-like figures but few telophase-like figures; (3) endoreduplicated metaphases (diplochromosomes); (4) lack of sister chromatid cohesion in the heterochromatic regions of the chromosomes.

**Figure 3:**
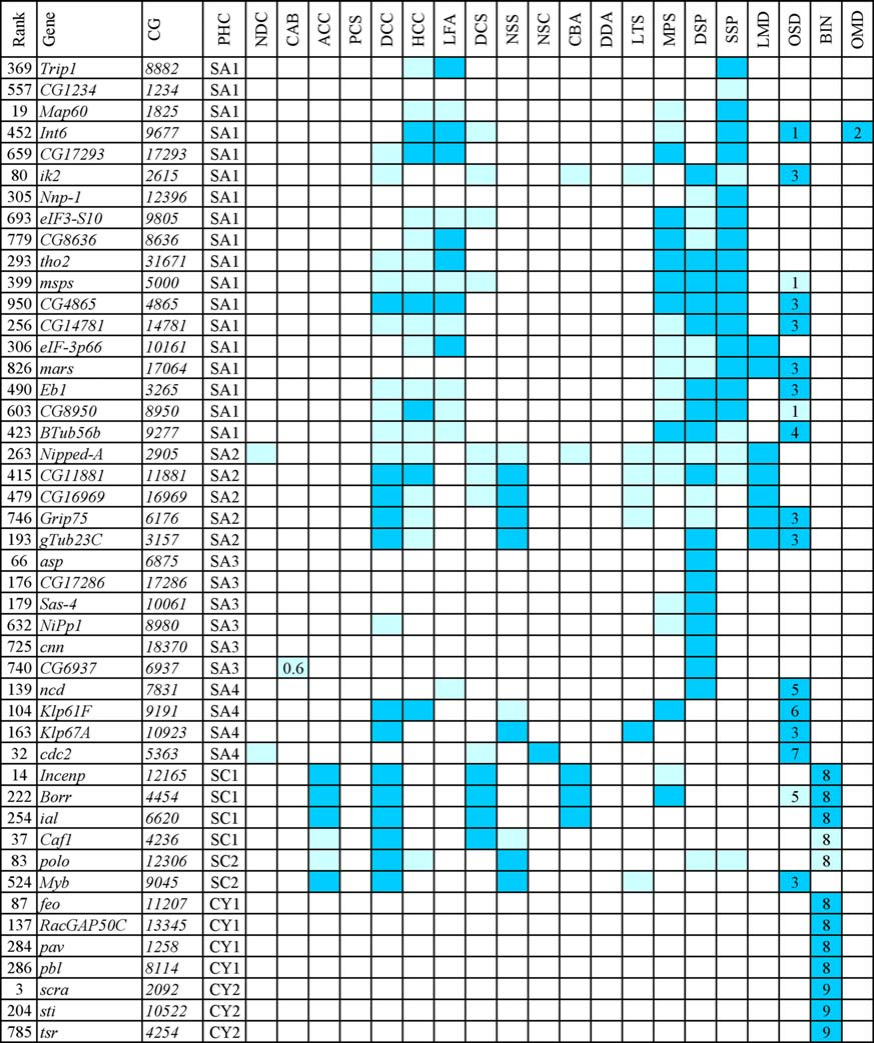
RNAi phenotypes elicited by the genes detected in the screen (SA, SC and CY phenoclusters). Colors refer to the strength of the phenotype: pale blue, weak; blue, strong. The numbers in the CAB column are frequencies of chromosome aberrations per cell. The other numbers refer to relatively rare phenotypic traits. PHC, phenocluster: SA, spindle assembly; SC, spindle assembly and chromosome condensation; CY, cytokinesis. See legend of Figure 2 for main phenotypic traits. Other phenotypic traits: (1) long astral MTs in telophase; (2) drastic undercondensation of the heterochromatic regions of the chromosomes; (3) disorganized spindles; (4): split spindle poles; (5) multipolar spindles; (6) umbrella-like telophase spindles with all chromosomes at the astral pole; (7) centrosomes/asters detached from the spindle poles; (8) early cytokinesis defects: central spindle and contractile ring are both abnormal; (9) late cytokinesis defects: central spindle and contractile ring are normally assembled.

**Figure 4:**
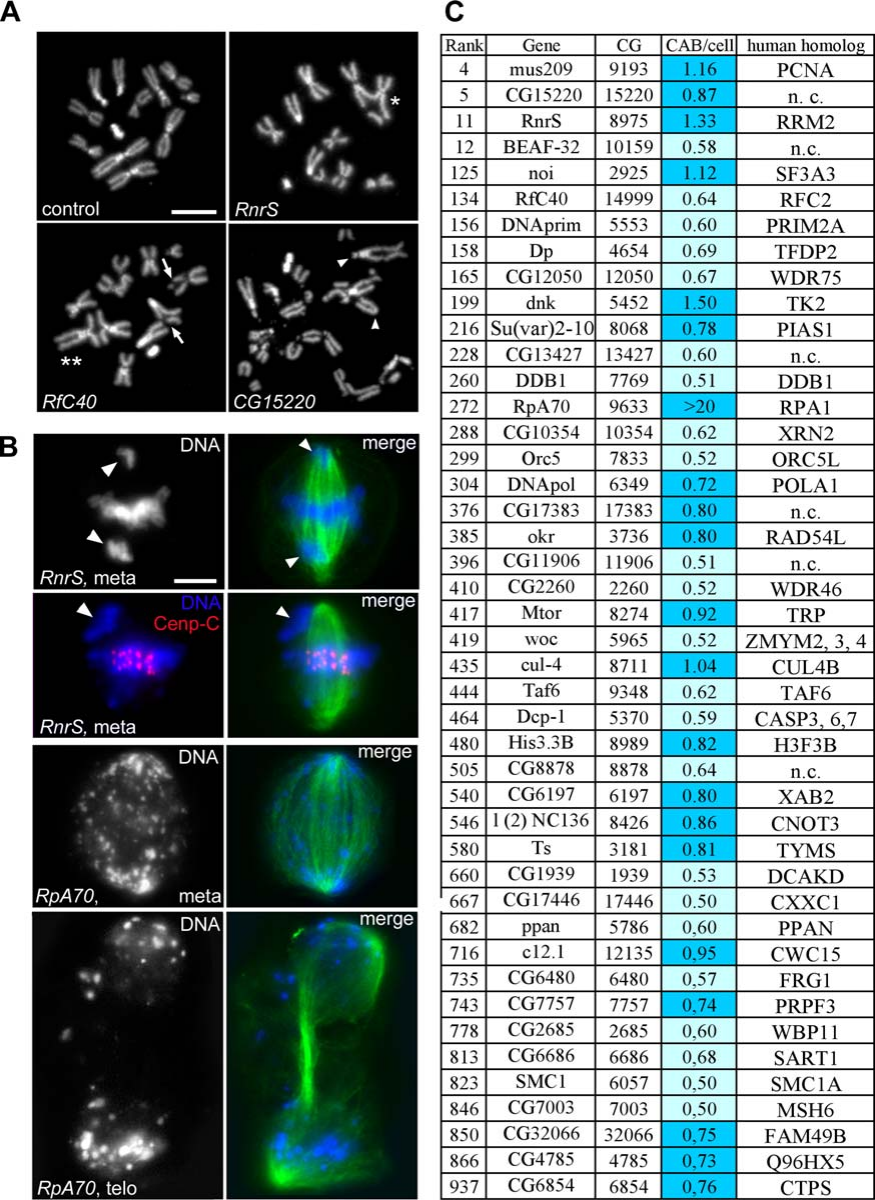
Genes required for the maintenance of chromosome integrity. (A) Examples of chromosome aberrations in colchicine/hypotonic-treated cells. Arrows and arrowheads point to chromatid and isochromatid deletions, respectively. Asterisks indicate asymmetric (U-type; one asterisk) and symmetric (X-type; two asterisks) chromatid exchanges. (B) Mitotic cells with acentric chromosome fragments (arrowheads) that have migrated to the cell poles. Staining for Cenp-C shows that the fragment at the pole of the RnrS-depleted metaphase (arrowhead) lacks the kinetochore (see text for further explanation). meta, metaphase; telo, telophase. In the merged figures, DNA is blue, Cenp-C red, and tubulin green. Scale bars, 5 µm. (C) The 44 genes required for chromosome integrity. Colors in the CAB (chromosome aberrations)/cell column denote the strength of the phenotype: light blue, weak; blue, strong. Numbers in this column are the frequencies of CABs per cell (see Methods for details). In control cells, the frequency of spontaneous chromosome aberrations is 0.24±0.014 per cell (n = 941; from 12 independent experiments).

Our screen identified 155 genes whose inactivation by RNAi causes a strong mitotic phenotype. Based on phenotypic analysis, these genes can be grouped in seven broad categories that we further subdivided in 18 phenoclusters (PHCs) [26]: 13 genes required for progression through the cell cycle, identified by dsRNAs that result in a complete (or nearly complete) absence of mitotic figures (PHC: NM); 44 genes required for chromosome integrity, identified by dsRNAs that cause chromosome aberrations (PHC: CA); 11 genes required for proper mitotic chromosome condensation (PHCs: CC1–CC3); 41 genes required for regular chromosome segregation (PHCs: CS1–CS5); 33 genes required for spindle assembly (PHCs: SA1–SA4); 7 genes required for cytokinesis (PHCs: CY1 and CY2); and 6 genes required for multiple mitotic functions (PHCs: SC1 and SC2) (Figures 1B, 2, 3, and 4C; Table S5; a synopsis on the functions of these genes can be found in Table S6). Remarkably, the distribution of these mitotic genes in our co-expression list was clearly nonrandom: their frequency decreased with an increase of their rank, further validating our co-expression approach (Figure 1C).

### Genes Required for Progression through the Cell Cycle

We identified 13 dsRNAs that result in the absence of dividing cells at 96 h after treatment initiation (Table S5). Six of these genes (*cdc2c*, *cyclinA*, *cyclinE*, *geminin*, *ran* and *string*) are well-known cell-cycle regulators. Two genes, *RpII140 and RpII215*, encode the 140 and 215 kDa subunits of RNA polymerase II, respectively. Three genes are involved in RNA metabolism and encode either canonical splicing factors (Prp8 and SF1) or the small DebB ribonucleoprotein, which is also likely to be involved in RNA splicing. Defects in *PRPF8*, the human homolog of *Prp8*, are one cause of Retinitis pigmentosa. Of the remaining two genes, *CG9273* encodes a protein with similarity to a subunit of DNA replication factor A, and *Bx42* specifies a protein involved in Notch signal transduction (Table S6).

### Genes Required for Chromosome Integrity

Although the 1000 genes in our list were selected for their co-expression with mitotic genes, our screen uncovered several functions required for chromosome integrity (Figures 1 and 4; Tables S5 and S6). As shown in Figure 4, we found 44 dsRNAs that significantly increase the frequency of spontaneous chromosome aberrations. Of the 44 genes identified by these dsRNAs, 38 have apparent human orthologs (Figure 4C) yet only 4 have previously been implicated in the maintenance of chromosome integrity (Table S6). These genes can be subdivided in several broad classes, based on their putative functions: (1) genes required for DNA replication, including *Ribonucleotide reductase (RnrS)*, *DNA primase (DNAprim)*, *DNA polymerase alpha (DNApol)*, *Orc5*, *RfC40*, *Rpa70*/*RPA1* and *peterpan (ppan)*; (2) genes involved in both DNA replication and repair, such as *mus209*/*PCNA*, *cul-4*, *thymidilate synthetase (Ts)* and *CG6854/CTP synthase*; (3) genes that mediate different aspects of DNA repair but are not known to participate in DNA replication, such as *DDB1*, *okra/RAD54L*, *CG6197/XAB2* and *CG7003/MSH6*; (4) genes involved in transcription and RNA maturation, including *Dp/TFPD2*, *CG10354/XRN2*, *Taf6*, *l(2)NC136/CNOT3*, *noi/SF3A3*, *CG7757/PRPF3)*, *without children (woc)*, *CG6480/FRG1* and *CG6686/SART1* (Table S6). Chromosome aberrations were also induced by RNAi against 8 genes whose diverse functions are not easily classified into the four groupings above. These include *BEAF-32*, that encodes a chromatin insulator factor; *dnk*, that specifies a deoxyribonucleotide kinase similar to the human mitochondrial kinase TK2; *Su(var)2-10*, whose product is an E3 SUMO ligase; the *H3.3B* histone variant gene; *SMC1*, that encodes a conserved cohesin involved in the Cornelia de Lange syndrome in humans; *Dcp-1*, that specifies a caspase precursor; *Megator (Mtor)* that encodes a component of the putative spindle matrix; and *CG17446*, whose product is homologous to a subunit of the mammalian Set1 histone methyltransferase complex (Table S6). Our screen also identified 12 chromosome stability genes without any assigned putative functions, 7 of which are conserved in humans. Together, these results indicate that the maintenance of chromosome stability requires a large number of functions, many of which remain to be identified.

The analysis of tubulin-stained mitotic figures revealed an interesting phenotype associated with the presence of chromosome aberrations. We observed many metaphase figures with the centric portions of broken chromosomes aligned at the metaphase plate and the acentric fragments near the cell poles (Figure 4B). Immunostaining for the kinetochore marker Cenp-C [27] verified that most chromosome fragments at the poles of these metaphases were indeed devoid of centromere (Figure 4B). This phenotype suggests that chromosome fragments severed from their kinetochores are transported to the cell poles. A similar phenomenon has been observed in plants *(Hemanthus)* and in crane fly spermatocytes. In both systems, when a metacentric chromosome is cut with the laser, the resultant acentric fragment moves to the closest cell pole at the same velocity as anaphase chromosomes [28],[29]. To explain this phenomenon, it has been suggested that the acentric chromosomes fragments adhere to the lateral surfaces or plus ends of microtubules and are transported poleward by the microtubule flux [29]. We believe that this mechanism also occurs in *Drosophila* S2 cells. Strong support for this view comes from observations on RPA70-depleted cells, which exhibit extreme chromosome fragmentation but form regular spindles. In these cells, most acentric fragments accumulate at the poles of ana/telophase figures, suggesting that they are driven poleward by microtubule-based forces (Figure 4B).

### Genes Required for Accurate Chromosome Segregation

**Figure 5:**
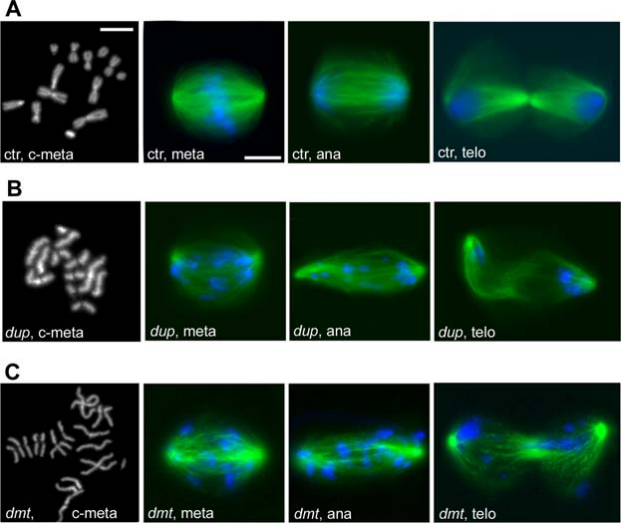
Mitotic phenotypes observed in the CS1 and CS2 phenoclusters. In all merged figures, DNA is blue and tubulin green. (A) Colchicine/hypotonic-treated metaphase chromosomes (c-meta) from control (ctr) cells; metaphase (meta), anaphase (ana) and telophase (telo) from untreated S2 cells. (B) Unreplicated chromosomes and impaired chromosome migration towards the spindle poles in cells treated with *dup* dsRNA (CS1). (C) Precocious sister chromatid separation and defective chromosome segregation observed after RNAi for *dmt* (CS2). Scale bars, 5 µm.

We identified 41 genes required for regular chromosome segregation. These genes are not required for spindle formation, as cell depleted for their products do not exhibit defects in late prophase/early prometaphase spindles. However, metaphase and ana/telophase spindles are often highly abnormal with respect to spindle morphology and the distribution of chromosomes along the spindle. The genes required for chromosome segregation (CS) can be subdivided into five phenoclusters (CS1, CS2, CS3, CS4 and CS5) based upon differences and similarities in the RNAi phenotypes (Figure 2). The CS1 group includes only the *doubleparked* (*dup*) gene. In most *dup* RNAi metaphase-like figures, the chromosomes are not replicated and have the appearance of single chromatid (Figure 5B). This is likely to results in a merotelic attachment of the spindle fibers to the kinetochore, leading to an impairment of chromosome movement during anaphase (Figure 5B). This phenotype has been previously observed in embryonic cells of *dup* mutants, suggesting that *dup* is required for both DNA replication and the checkpoint that prevents mitosis until completion of S-phase [30],[31]. RNAi for the 5 genes in the CS2 group resulted in precocious sister chromatid separation, lack of chromosome congression to the cell equator at metaphase, and unequal or otherwise abnormal sister chromatid segregation (Figures 5C and S1). Four of the genes included in this CS2 phenocluster (*bub1*, *bub3*, *zw10* and *rod*) are well known components of the spindle checkpoint machinery (Table S6). The other gene, *dalmatian* (*dmt*) has never been implicated in this checkpoint. However, since studies in *C. elegans* have clearly shown that genes with similar RNAi phenotypes are often required for a common process [26], [32]–[34], we propose that *dmt* might play a role in the spindle checkpoint.

**Figure 6:**
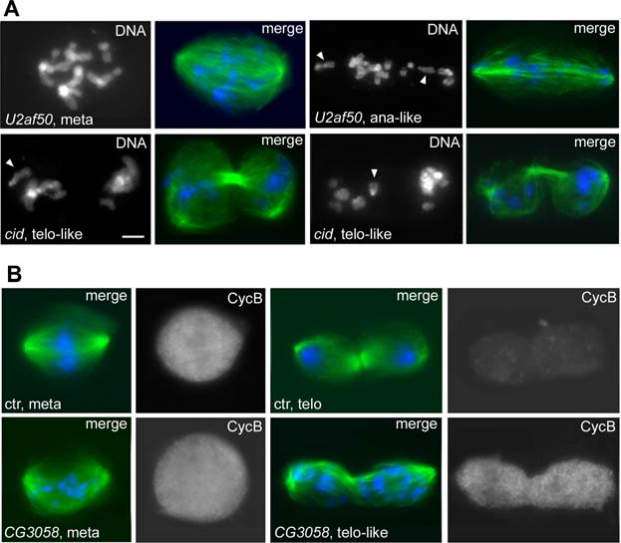
Mitotic phenotypes observed in the CS3 phenocluster. In merges, DNA is blue and tubulin green. (A) Top panels, metaphase- and late anaphase-like figures from *U2af50* (splicing factor) RNAi cells. Bottom panels, telophase-like figures from *cid* RNAi cells. Arrowheads point to chromosomes comprised of both sister chromatids. (B) Cyclin B distribution in control (top panels) and in *CG3058* (splicing factor) RNAi cells (bottom panels). The Cyclin B-stained cells correspond to the mitotic figures at their left. Scale bar 5 µm.

**Figure 7:**
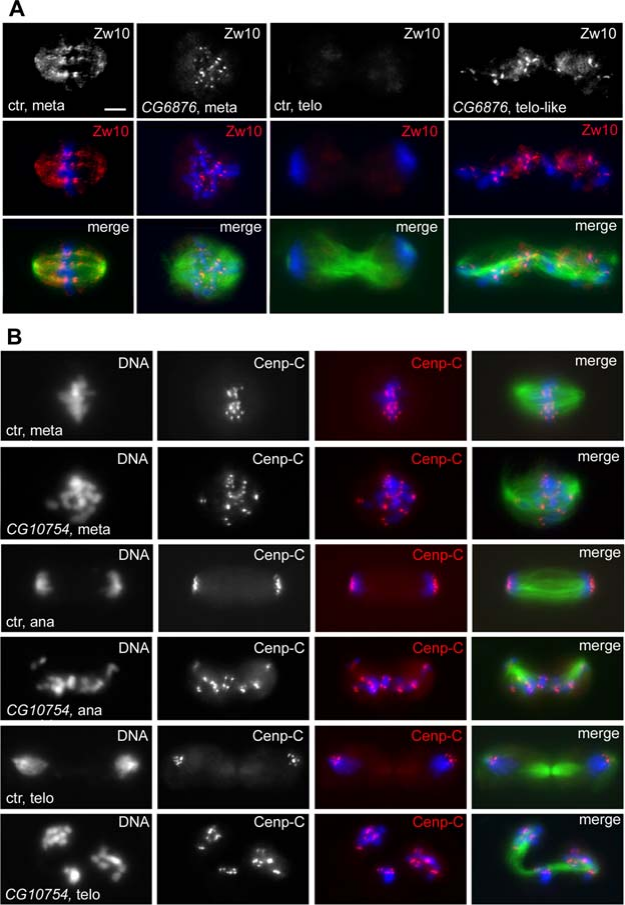
Lack of ZW10 streaming and failure in sister chromatid separation observed after RNAi for genes of the CS3 phenocluster. In merges, DNA is blue and tubulin green. (A) ZW10 does not stream towards the spindle poles in metaphase and remains associated with kinetochores in telophase after RNAi for *CG6876* (splicing factor). The ZW10 signal is white in the top panels and red in merges below each of these panels. (B) Cenp-C staining (red in merges) shows that ana-telophase-like figures generated by RNAi to the genes of the CS3 group contain chromosomes that comprise both sister kinetochores. Ctr, control; meta, metaphase; ana, anaphase; telo, telophase. Scale bar, 5 µm.

Inactivation of the 18 genes in the CS3 phenocluster (Figure 2) resulted in a peculiar mitotic phenotype. The chromosomes of metaphase-like figures were not connected to the spindle poles by bundles of kinetochore microtubules (MTs) and thus never congressed to the equator of the spindle. In addition to metaphase-like spindles, the RNAi cells of the CS3 phenocluster also showed many elongated ana/telophase spindles. However, these spindles contained chromosomes with unseparated sisters chromatids; these chromosomes usually appeared to segregate to the poles at random (Figures 6, 7 and S2–S4). Some of these peculiar ana/telophase-like figures displayed both a central spindle and an actin-based contractile ring (Figure S5). However, most of these structures were morphologically irregular and were thus probably unable to mediate cytokinesis.

To define their phenotype in more detail, RNAi cells for the CS3 genes were stained for the checkpoint proteins ZW10 and BubR1 and also for the cell cycle marker Cyclin B. In most ana/telophase-like figures, Cyclin B was still high, whereas in control cells it was degraded during anaphase and absent from telophases (Figure 6B). In the metaphase-like RNAi figures, ZW10 did not exhibit any streaming towards the cell poles as occurs in normal metaphases (Figure 7A), consistent with a defect in microtubule attachments to the kinetochore [35]. Moreover, the ana/telophase-like figures showed strong ZW10 and BubR1 centromeric signals; these signals were mostly absent from control ana/telophase chromosomes (Figure 7A and data not shown). Finally, the chromosomes of the ana/telophase-like cells displayed two centromeric spots after staining for the kinetochore marker Cenp-C (Figure 7B). These findings confirm that the chromosomes at the poles of the ana/telophase spindles seen in the CS3 phenocluster are indeed comprised of both sister chromatids.

The CS3 phenocluster includes the *CG9938/Hec1/Ndc80*, *CG8902/Nuf2* and *CG1558/Nsl1* genes, which encode interacting components of the *Drosophila* kinetochore [36],[37], as well as *cid/Cenp-A*, that encodes the *Drosophila* centromere-specific histone H3 variant [17],[18]. Of the remaining 14 genes in the CS3 group, one specifies a conserved product of unknown function *(CG8233)* and 13 encode highly conserved splicing factors (Table S6). The RNAi phenotypes of the genes in the CS3 phenocluster suggest that their products are required for proper kinetochore-microtubule interactions. We propose that in the absence of these interactions, the spindle checkpoint remains engaged and sister chromatid separation does not occur. The high levels of Cyclin B and the lack of ZW10 streaming in CS3 RNAi cells are both consistent with this hypothesis [38]. We further posit that the chromosomes are driven to the spindle poles by the same forces that act on the acentric chromosome fragments. As the chromosomes move towards the poles, the spindle elongates so as to resemble an ana/telophase spindle; some of these spindles manage to assemble a defective central spindle and attempt to undergo cytokinesis. Collectively, these results provocatively indicate that in S2 cells typical telophase events, such as central spindle assembly and initiation of cytokinesis, can occur in the absence of sister chromatid separation.

**Figure 8:**
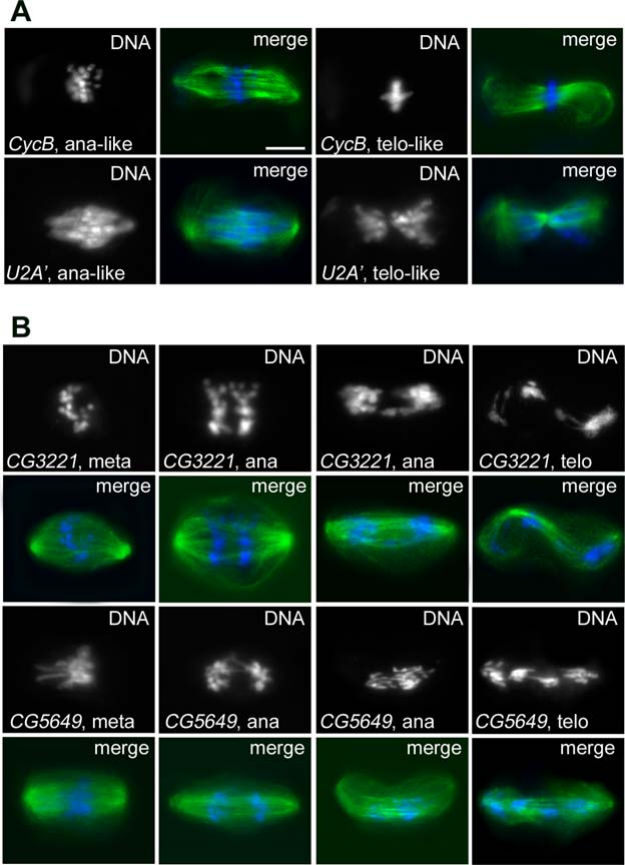
Mitotic phenotypes observed in the CS4 and CS5 phenoclusters. In merges, DNA is blue and tubulin green. (A) In RNAi cells of the CS4 phenocluster, sister chromatids do not separate so that the chromosomes remain at the center of the cell, while the spindles elongate and assume morphologies typical of ana/telophase figures. Top panels, anaphase-like and telophase-like spindles from *Cyclin B* RNAi cells. Bottom panels, anaphase-like and telophase-like spindles from *U2A′* (splicing factor) RNAi cells. Note that chromosome arms are parallel to the spindle axis (see text for explanation). (B) Mitotic figures from the CS5 phenocluster. In both *CG3221* and *CG5649* RNAi cells, the two sets of segregating chromosomes remain close to each other and fail to reach the spindle poles (compare with control cells in Figure 4A). Meta, metaphase; ana, anaphase; telo, telophase. Scale bars, 5 µm.

RNAi for the 9 genes in the CS4 group resulted in a pseudo metaphase-arrest phenotype (Figures 2, 8A, and Figure S6). Most dsRNA-treated cells with spindles of metaphase shape displayed apparently normal kinetochore fibers and normal chromosome congression. However, we also observed many mitotic figures with elongated ana/telophase-like spindles and unsegregated chromosomes at the center of the cell. In these peculiar mitotic figures, the centromeres of most chromosomes had congressed to the middle of the spindle, while the chromosomes arms were parallel to the spindle axis with the telomeres pointing towards the spindle poles. In addition, in many cells with long telophase-like spindles, the chromosomes stuck at the cell equator displayed variable degrees of decondensation, as through they were undergoing the decondensation process that occurs during normal telophase (Figure 8A and Figure S6). Finally, in most ana/telophase-like figures, Cyclin B remained high, as observed in RNAi cells for the CS3 genes (data not shown).

The CS4 phenocluster includes the *Separase* and *three rows* (*thr*) genes, which encode interacting proteins required for sister chromatid separation at anaphase (Figure 2 and Table S6). Previous studies have shown that embryonic cells of *thr* mutants display metaphase arrest with congressed chromosomes, followed by an irregular extension of the spindle without chromosome segregation and by chromosome decondensation [39],[40]. This phenotype is fully comparable to that we observed in S2 cells after *thr* downregulation by RNAi. The CS4 group also includes the *CyclinB* gene and *Otefin*, a gene encoding a non-conserved protein that may interact with lamin (Table S6). All the remaining genes in the group are involved in RNA metabolism: one specifies a putative transcription factor while the others encode conserved splicing factors (Table S6). The genes included in the CS4 phenocluster are likely to be required for sister chromatid separation at the anaphase onset. We propose that upon inactivation of these genes, the opposing forces exerted by the MTs attached to the sister kinetochores keep the centromeres aligned at the metaphase plate. At the same time, however, the same forces that mediate the poleward motion of acentric fragments act on the chromosome arms, orienting them parallel to the spindle axis. Our observations also suggest that the latter forces can occasionally prevail over those exerted by the kinetochore fibers, so that some chromosomes leave the metaphase plate and move towards the poles with unseparated chromatids. The finding that RNAi cells for the CS4 genes undergo spindle elongation and chromosome decondensation while arrested in a metaphase-like state provides further support for the view that in S2 cells telophase events do not require sister chromatid separation.

RNAi for the 9 genes in the CS5 phenocluster resulted in defective chromosome congression at metaphase and abnormal chromosome segregation at anaphase (Figure 2). Knockdowns of the expression of most of these genes caused a partial metaphase arrest characterized by extremely contracted chromosomes. However, even though sister chromatid separation did occur in most of the cases, ana/telophases were severely defective. The segregating chromatids were highly contracted and the two chromatid sets remained close to each other in many cells (Figure 8B and Figure S7). These unusual ana/telophases resemble very early anaphase figures, which are quite rare in untreated cells. These observations suggest that chromosome movement towards the poles is partially impaired in RNAi cells, resulting in delayed and irregular chromosome segregation.

The CS5 phenocluster includes *ida/APC5* and *CG11419/APC10*, that encode two subunits of the APC complex; and *fizzy (fzy)/Cdc20*, whose product regulates APC/C activity (Table S6). This phenocluster also includes *Pros26.4*, that specifies a proteasome subunit; *Klp3A*, that encodes a kinesin-like protein; *CG4266* and *kin17*, whose products are conserved proteins implicated in RNA metabolism and the stress response, respectively; and *CG3221*, that encodes a poorly conserved product of unknown function. The finding that the phenotype elicited by depletion of the APC components is substantially different from that caused by Separase inhibition strongly suggests that the APC/C is required not only for Securin and Cyclin B degradation, but also for the regulation of other aspects of spindle dynamics and spindle-kinetochore interactions.

Inactivation of the genes in the CS1–CS5 groups often resulted in very elongated ana/telophase spindles (Figure S8); in some cases, these spindles were twice as long as their counterparts in control cells. Long spindles were often bent or S-shaped, probably due to mechanical constraints imposed by the plasma membrane (Figures S1, S2, S3, S4, S5, S6, S7, and S8). In addition, we observed that the degree of spindle elongation correlates with the presence of scattered chromosomes between the spindle poles. Long spindles have been observed previously in both *Drosophila* and mammalian cells with defective kinetochores [2],[10],[37],[41], and have been attributed to a misregulation of tubulin addition at the plus ends of kinetochore MTs [2],[41]. We observed long ana/telophase-like spindles in cells containing chromosomes with either functional or nonfunctional kinetochores. Thus, spindle elongation may depend on factors other than kinetochore dysfunction. For example, the chromosomes scattered within the aberrant ana/telophase figures may induce MT growth and/or stabilization [42], leading to the formation of particularly long spindles.

### Genes Required to Maintain Proper Chromosome Structure

**Figure 9:**
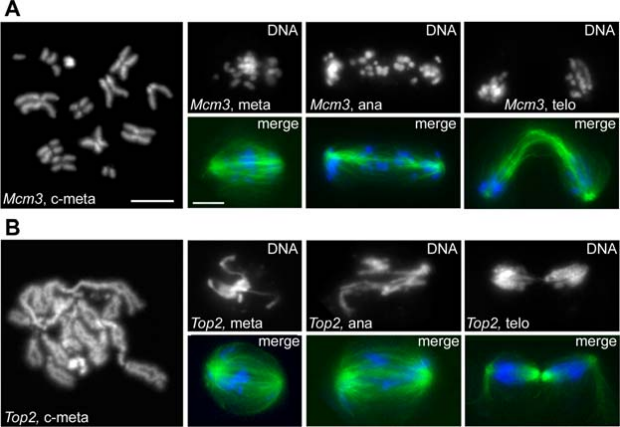
Mitotic phenotypes observed in the CC1 and CC3 phenoclusters. In merges, DNA is blue and tubulin green. (A) colchicine/hypotonic-treated metaphase chromosomes (c-meta); metaphase (meta), anaphase (ana) and telophase (telo) observed after RNAi for *Mcm3* (CC1). Note the relative lack of sister chromatid cohesion in the heterochromatic regions of c-metaphase chromosomes, as well as difficulties in chromosome congression and segregation. (B) C-metaphase (c-meta) and mitotic figures in *Top2* (CC3) RNAi cells. Metaphase chromosomes are poorly condensed, leading to problems in chromosome separation during ana/telophase. Scale bars, 5 µm.

We identified 11 dsRNAs that cause defects in chromosome structure without affecting spindle assembly. The phenotypes produced by these RNAs can be grouped into three phenoclusters we call CC1, CC2 and CC3 (Figure 2). The CC1 group includes *Minichromosome maintenance 3 (Mcm3)*, *Mcm7* and *cap*. *Mcm3* and *Mcm7* encode the orthologs of two components of the human (MCM)2-7 helicase complex (Table S6), while *Cap* encodes a protein orthologous to the SMC3 cohesin whose mutant form is responsible for a mild variant of the Cornelia de Lange syndrome [43]. RNAi for these genes resulted in loss of sister chromatid cohesion in the heterochromatic regions of the chromosomes and defective chromosome congression and segregation (Figure 9A and Figure S9). A similar phenotype was previously observed in mutants in the *wings-apart like (wapl) Drosophila* gene [44]; the human ortholog of Wapl interacts with cohesin and regulates its association with chromatin [45],[46].

The CC2 phenocluster includes 5 genes that encode well-known condensins: SMC2, Gluon/SMC4, CapD2, Cap-G and Barren/CAP-H (Figure 2 and Table S6). RNAi for these genes resulted in very similar phenotypes. In all cases, chromosomes displayed an abnormal mitotic condensation: although their longitudinal axis was shortened normally, their sister chromatids were swollen and fuzzy. In addition, ana/telophase figures displayed frequent lagging chromosomes and chromatin bridges, consistent with a strong defect in sister chromatid resolution during anaphase (Figure S10).

In contrast, in RNAi cells for either gene in the CC3 group, *Topoisomerase II (Top2)*, *greatwall (gwl)* (encoding a conserved kinase; see Table S6) and *Orc-2*, metaphase chromosomes were abnormally elongated and irregularly condensed, suggesting a defect in chromosome shortening. In RNAi cells for these genes, chromosome congression and segregation were also affected, consistent with previously published results (Figure 9B; Table S6).

### Genes Required for Spindle Assembly

**Figure 10:**
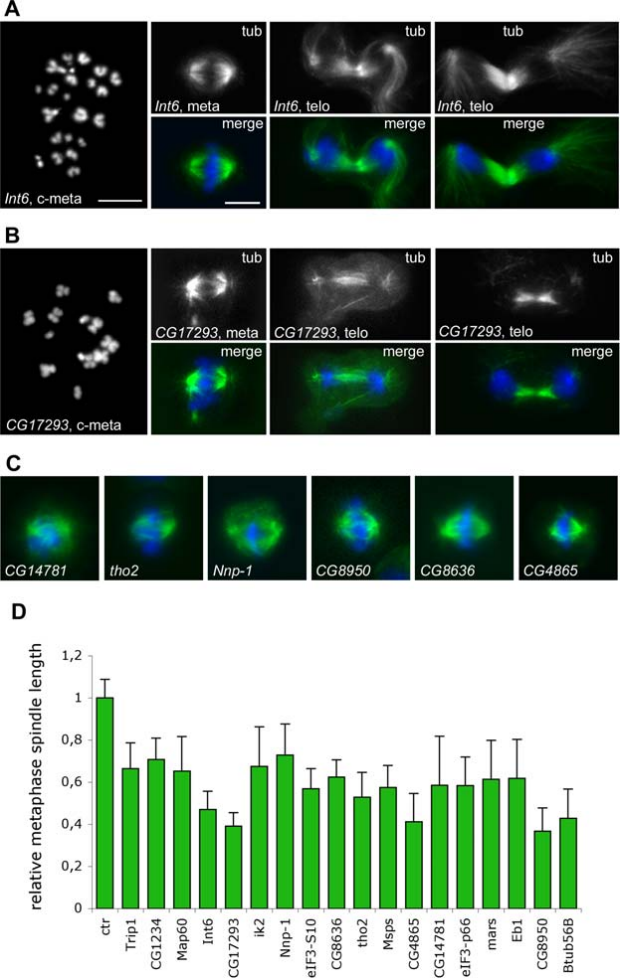
Mitotic phenotypes observed in the SA1 phenocluster. In merges, DNA is blue and tubulin green. (A) C-metaphase (c-meta) and mitotic figures observed after RNAi for *int6*. The heterochromatic regions of c-metaphase chromosomes are drastically undercondensed; note also the long astral microtubules in the telophase figures. (B) Extremely contracted chromosomes (c-meta) and short spindles in *CG17293* RNAi cells. (C) Examples of short metaphase spindles observed after RNAi for the SA1 genes. (D) pole-to-pole metaphase spindle lengths (mean±SE) observed in the SA1 phenocluster. The average metaphase spindle lengths observed after RNAi for the 17 SA1 genes are all significantly different (with p<0.001 in Student's t-test) from the average spindle length of control (ctr) metaphases. Scale bars, 5 µm.

RNAi for 33 genes caused defects in spindle structure that were apparent as early as prophase or the beginning of prometaphase. Most of these genes (29/33) can be grouped in three broad phenoclusters (SA1, SA2 and SA3); although the phenotypes associated with the remaining 4 genes do not resemble each other, we assign them to a single miscellaneous group (SA4) for convenience (Figure 3). Inactivation of the 18 genes in the SA1 group resulted in the formation of bipolar spindles that were significantly shorter than control spindles. Knockdowns of most of these genes also caused poorly focused spindle poles, monopolar spindles, hypercontracted chromosomes and defects in chromosome congression and segregation (Figures 3, 10; Figures S11 and S12). The monopolar spindles observed in these RNAi cells might not reflect defective centrosome separation at prophase, but instead be a consequence of the instability of short bipolar spindles. This is suggested by previous observations of Orbit/Mast-depleted S2 cells. Live imaging of these cells has shown that the centrosomes of bipolar minispindles often collapse towards each other during prometaphase to form a monopolar spindle [47]. The excessive chromosome contraction is the likely outcome of a delayed progression through mitosis and could be responsible for a partial impairment of kinetochore function, resulting in defective chromosome congression and segregation.

The SA1 phenocluster includes the β-tubulin gene *β-tub56B*, 4 genes that encode MT-interacting proteins [Map60/CP60, Eb1, Minispindles (Msps) and Mars/HURP], the mitotic kinase gene *ik2*, and 3 genes (*CG4865*, *CG14781* and *CG17293*) that encode proteins of unknown function (Table S6). The remaining 9 genes of the SA1 group are involved in either transcription or translation. *CG8950* encodes a PolII transcription factor; *tho2* specifies a component of the conserved THO complex, which couples splicing and mRNA export (Table S6). *Trip1/eIF3-S2*, *CG8636*/*eIF3-S4*, *Int6*/*eIF3-S6*, *eIF3-p66* and *eIF3-S10* encode different subunits of the highly conserved eukaryotic translation initiation factor 3, while *Nnp-1* and *CG1234* are involved in ribosome biogenesis or maturation (Table S6). The *Int6* gene is a frequent integration site of the MMTV virus in mouse mammary tumors and its silencing leads to mitotic defects in human cells (Table S6). In addition to short spindles, Int6-depleted cells displayed two unusual phenotypic traits: a severe undercondensation of the pericentric regions of the chromosomes and abnormally long astral MTs in telophase figures (Figure 10A). Horse tail-like telophase asters were also observed in *msps* and *CG8950* RNAi cells (Figure 3 and Figure S11).

**Figure 11:**
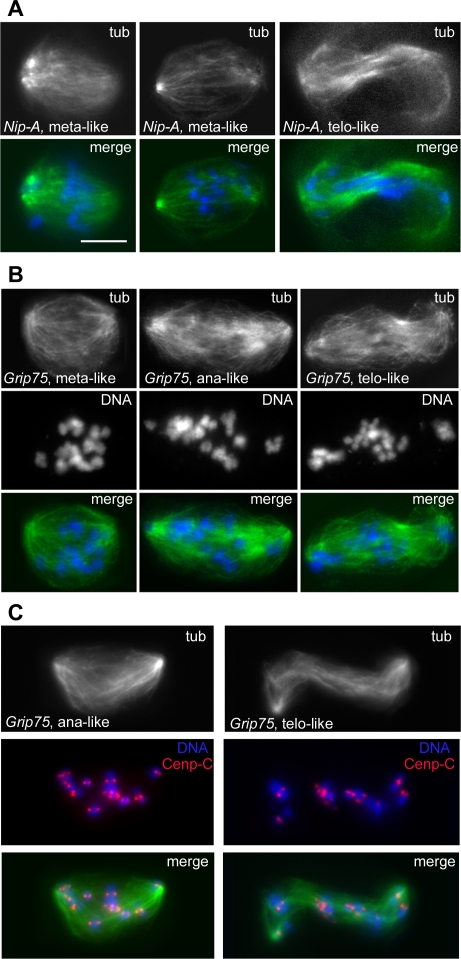
Mitotic phenotypes observed in the SA2 phenocluster. In merges, DNA is blue and tubulin green. (A) Mitotic figures with low microtubule density showing defective chromosome segregation after RNAi for *NippedA*. (B) Disorganized spindles with low microtubule density in *Grip75* RNAi cells; note that the ana/telophase-like spindles contain chromosomes with unseparated sister chromatids. (C) Staining for Cenp-C (red) shows that the chromosomes of the ana/telophase-like figures of *Grip75* RNAi cells comprise both sister kinetochores. Scale bar, 5 µm.

The SA2 phenocluster includes only 5 genes: *CG11881* and *CG16969*, that encode proteins of unknown function; *Grip75* and *γ tubulin 23C*, that specify components of the gamma tubulin ring complex; and *NippedA*, that encodes a subunit of the conserved TRRAP complex implicated both in transcriptional regulation and DNA repair (Table S6). Interestingly, loss of the TRRAP complex affects gene expression at mitotic stages [48]. Downregulation of the genes in the SA2 group results in spindles with a low MT density and poorly focused poles (Figure 11 and Figure S13). Aberrant spindles with low MT density have previously been observed in S2 cells depleted of gamma tubulin ring components [49]. RNAi cells for the SA2 genes were also defective in chromosome congression and sister chromatid separation, just as those of the CS3 phenocluster (Figure 11). This phenotypic profile suggests that the products of the SA2 genes are required for the stability of spindle MTs and for their interaction with the kinetochores.

**Figure 12:**
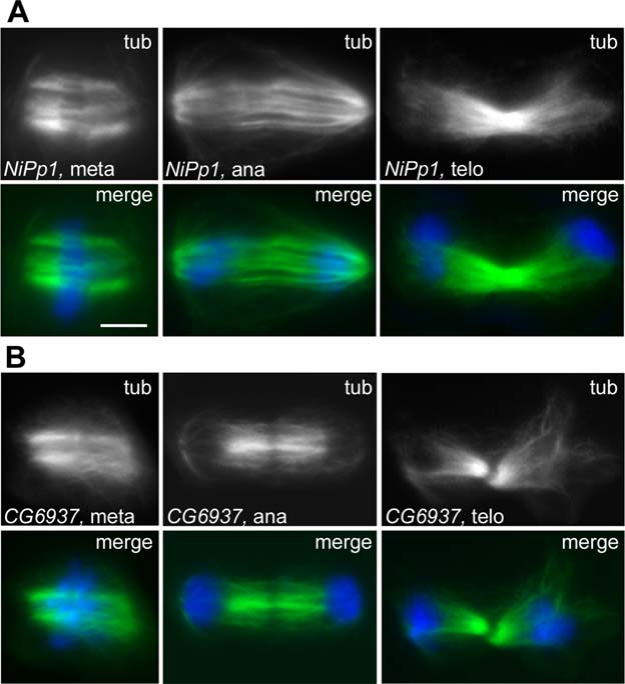
Mitotic phenotypes observed in the SA3 phenocluster. In merges, DNA is blue and tubulin green. Spindles with broad, anastral poles in *NiPp1* (A) and CG6937 (B) RNAi cells. Note that these spindles exhibit a normal microtubule density. Scale bar, 5 µm.

RNAi for the 6 the genes in the SA3 phenocluster resulted in anastral and poorly focused spindles with normal MT density (Figure 12 and Figure S14). These genes encode the DSas-4 protein required for centriole duplication; the PCM component Centrosomin (Cnn) required for MT nucleation; the *CG17826* product homologous to the *C. elegans* centrosomal protein Spd2; and Abnormal spindle (Asp), a protein that associates with both the centrosomes and the MT minus ends (Table S6). The other two genes in the SA3 group encode the NiPp1 inhibitor of protein phosphatase type 1 (PP1); and the *CG6937* product, which is homologous to the human MKI67IP protein that contains an RNA recognition motif and interacts with the Ki-67 mitotic protein (Table S6). The phenotypic differences between *DSas-4*, *cnn* or *CG17826* RNAi cells (SA3 phenocluster) and those depleted of either Dgrip75 or γ tubulin (SA2) are intriguing; they support the view that the latter proteins are not only involved in MT nucleation from the centrosomes but are also required for either MT stability or chromatin and/or kinetochore-induced MT growth [50].

We have included in the SA4 group 4 genes that are essential for spindle assembly but elicit different phenotypic profiles when inactivated by RNAi. Consistent with previous results, RNAi of *Klp61F* and *ncd* resulted in monopolar spindles and disorganized bipolar or multipolar spindles, respectively, while *Klp67A* downregulation led to abnormally long MTs that are unable to interact properly with the kinetochores (Figure 3 and Figure S15; Table S6). The phenotype of cells treated with dsRNA for *cdc2* is reminiscent of that of the CS3 phenocluster, with chromosomes that remain congressed in a metaphase plate even when the spindle assumes an ana/telophase configuration. However, *cdc2* RNAi cells often show an additional phenotype in which the centrosomes/asters are detached from the spindle poles (Figure 3 and Figure S15).

### Genes Required for Chromosome Condensation, Spindle Formation, and/or Cytokinesis

We identified 6 genes required for both chromosome condensation and spindle formation, which can be subdivided into two phenoclusters (SC1 and SC2). The SC1 phenocluster includes the three components of the chromosome passenger complex (Incenp, Ial/Aurora B and Borealin), as well as chromatin assembly factor 1 (Caf1). Consistent with previous studies, downregulation of these proteins resulted in elongated and poorly condensed chromosomes, disorganized spindles, defective chromosome congression and segregation, and frequent failures in cytokinesis (Figure 3 and Figure S16; Table S6).

The SC2 group includes Polo kinase and the *Drosophila* homolog of the Myb transcriptional activator (Figure 3 and Table S6). In *polo* RNAi cells, the chromosomes were fuzzy and irregularly condensed, while in Myb-depleted cells the chromosomes were overcontracted and swollen with no resolution between sister chromatids. Dowregulation of either of these two proteins disrupts MT-kinetochore interactions, leading to failures of chromosome congression and sister chromatid separation (Figure 3 and Figure S16).

### Genes Required for Cytokinesis

The RNAi phenotypes of the 7 genes required for cytokinesis (Figure 3) have been described previously in greater detail (Table S6). They can be subdivided into two phenoclusters (CY1 and CY2). Inactivation of the genes in the CY1 group [*fascetto (feo)*, *racGAP50*, *pavarotti (pav)* and *pebble (pbl)*] results in early cytokinetic defects in both the central spindle and the contractile ring. In contrast, ablation of the CY2 genes [*anillin (ani)*, *citron kinase (sti)* and *twinstar (tsr)*] does not affect either central spindle or contractile ring assembly, but it does disrupt the final stages of cytokinesis (Figure 3 and Table S6).

## Discussion

Our RNAi screen for mitotic genes differs from those previously performed in two important ways. First, we used a bioinformatic approach to focus our experiments on a group of genes that was enriched in mitotic functions. Second, we analyzed potential mitotic phenotypes not only by examining cells stained for tubulin and DNA, but also by looking at colchicine-treated chromosome preparations. Since this latter technique allows the analysis of well spread metaphase chromosomes with excellent cytological resolution, we were able to identify 44 genes required to prevent spontaneous chromosome breakage, most of which have not previously been implicated in the maintenance of chromosome integrity. The human orthologs of some of these genes may play roles in carcinogenesis, as shown for many genes required for chromosome stability [51],[52]. In addition, examination of colchicine-arrested metaphases led to the detection of phenotypes such as precocious sister chromatid separation (CS2 phenocluster) and a lack of sister chromatid cohesion in the heterochromatic regions (CC1 phenocluster). These phenotypic traits allowed us to distinguish between genes required for proper chromosome segregation, permitting their assignment to different functional groups.

Although previous RNAi screens were not designed to detect subtle changes in chromosome structure, they identified many genes involved in spindle assembly, chromosome segregation and cytokinesis [1]–[10]. Of particular interest is a comparison between our screen and a recent genome-wide screen performed by Goshima and coworkers in S2 cells [10] (see Text S1 and Table S7 for details). Goshima *et al.* used automated microscopy to identify 189 genes required for spindle assembly and chromosome alignment at metaphase. Remarkably, 38% of these 189 genes are included in the first 1000 genes of our consensus co-expression list and 50% in the first 2000. We identified 98 genes involved in the same processes, 30 of which were not found in the Goshima et al. screen. However, we failed to detect 17 genes that elicited RNAi phenotypes in their screen. Together, these results further validate our co-expression-based method for the identification of mitotic genes by RNAi. We believe that elaboration of consensus co-expression lists using genes in the same phenocluster will provide many candidate genes for small-scale RNAi screens aimed at completing the inventory of proteins involved in specific mitotic processes.

One striking and unanticipated finding among our results merits special attention. We identified 17 highly conserved splicing factors that are required for sister chromatid separation at anaphase. RNAi for the genes encoding these factors resulted in two types of aberrant mitotic figures. Downregulation of the genes in the CS3 phenocluster resulted in mitotic cells showing scattered chromosomes without apparent kinetochore-spindle connections. This phenotype was identical to that caused by downregulation of genes encoding well-known kinetochore proteins such as *cid*, *CG9938/Ndc80/Hec1*, *CG8902/Nuf2* or *l(1)G023/Nsl1*. In RNAi cells for genes in the CS4 phenocluster, most chromosomes showed regular connections with the spindle fibers and remained at the center of the cell, a phenotype similar to that produced by downregulation of Separase. However, RNAi for all of the CS4 genes and some of the CS3 genes produced a fraction of cells with an intermediate CS3/CS4 phenotype. These observations raise the possibility that inactivation of the splicing factor genes of both phenoclusters causes the same primary defect in centromere/kinetochore organization. One can envisage that when this defect is strong, both sister chromatid separation and kinetechore MT-interaction are affected; when the defect is weak, sister chromatid separation would be disrupted with little effect on kinetochore function.

Splicing factors have previously been implicated in mitosis in fission yeast, *Drosophila* and human cells [10], [53]–[55]. However, the precise mitotic function of these splicing factors has never been described. We have clearly shown here that splicing factors are required for sister chromatid separation. However, the mechanisms by which splicing factors regulate centromere/kinetochore function remain unclear. It is possible that these factors mediate the splicing of one or more pre-mRNAs, whose protein products play crucial roles for proper centromere or spindle function. Alternatively, the splicing factors may be involved in the production and/or stabilization of spindle- or centromere-associated structural RNAs. Recent studies have shown that RNA associates with the mitotic spindle and plays a translation-independent role in spindle assembly [56]. Moreover, there is evidence that maize and human kinetochores are enriched in single-stranded RNAs encoded by centromeric DNA sequences. It has been suggested that these RNAs may facilitate proper assembly of centromere-specific nucleoprotein complexes [57],[58]. Deciphering the precise role of splicing factors in centromere/kinetochore assembly and functioning will be a challenging task for future studies.

## Methods

### Generation of Co-Expression Lists

Co-expression analysis was perfomed on a previously described gene expression dataset [14]. This comprised 267 GeneChip *Drosophila* Genome Arrays (Affymetrix, Santa Clara, CA, USA) that covered 89 different embryonic and adult experimental conditions and contained expression data for 13,166 probesets (corresponding to 12,229 genes in the current FlyBase release). Pearson correlation coefficients (PCC) were calculated on log_2_ transformed expression values, averaged for each experimental condition [14]. For each of the six prototype mitotic genes, we obtained a ranked coexpression list by calculating the PCC of the corresponding probeset with all the other probesets of the gene expression matrix. In the case of two or more probesets referring to the same gene, we considered only those showing the highest ranks. To obtain a ranked consensus co-expression list, we first scored every probeset for its presence in the upper 3000 ranks of the single co-expression lists and calculated the average PCC; we then ordered the probesets for decreasing values of these parameters (Table S1).

The first 3,000 genes in this consensus co-expression list are reported in Table S1. 56 of the putative genes in the first 1000 positions of the list were either too small (less than 300 bp) or otherwise could not be amplified by PCR using at least two different pairs of primers. These putative genes (highlighted in grey in Table S1) were not assayed in our RNAi screen. Thus, to total 1000 genes, we performed RNAi for 56 additional genes that ranked from 1001 to 1056 on the consensus coexpression list (Table S1).

### Validation of the Coxpression Lists

To ascertain the predictive value of our coexpression lists, we determined the ranks of 164 known mitotic genes in each list. These genes were selected because their ablation or knockdown by either mutation or RNAi has previously been shown to result in a strong mitotic phenotype. These 164 genes represent most of the *Drosophila* mitotic genes so far identified, but they do not include new mitotic genes detected in a recent RNAi screen performed by Goshima et al. [10] (see Text S1 and Table S7 for a comparison with this screen). As shown in Tables S2 and S3, 46% of these 164 genes are included in the first 1000 genes of our consensus coexpression list, which contains in total more than 13,000 genes. This non-random clustering indicates that there is both a strong tendency for mitotic genes to be transcriptionally coexpressed, as well as a strong positive correlation between the rank of a gene in the list and the probability of its involvement in mitosis. Examination of Tables S2 and S3 shows that the mitotic genes that are most tightly coexpressed are those required for proper chromosome structure and/or condensation. In contrast, the genes implicated in cytokinesis exhibit the broadest variation in expression patterns. This variation might reflect the complexity of the cytokinetic process, which requires several functions that are not specific for mitosis. For example, functions involved in regulation of the actin cytoskeleton or membrane trafficking are likely to be required in many cellular processes in addition to cell division.

### dsRNAs used for RNAi

Recent work has raised the issue of possible off-target effects (OTEs) associated with the use of long dsRNAs for RNAi screens. It has been suggested that short homology stretches of 19 base pairs (bp) within a long dsRNA can target a gene other than the intended one [24],[59],[60]. In designing the primers for dsRNA synthesis, we consistently tried to obtain RNAs longer than 600 bp with a minimum content of OT sequences. The average length of the RNAs used in our screen was 750 bp; none of these RNAs carried strings of CAR triplets that are known to result in OTEs [25], but 54% of our dsRNAs contained at least one OT sequence (Table S4; OT sequences were identified using the program developed by Flockhart et al. [61]: http://flyrnai.org/RNAi_find_frag_free.html). However, we have several reasons to believe that very few, if any, of these OT sequences contributed to the production of the observed phenotypes. First, except for the “no dividing cells” (NDC) phenotype, we looked at very specific, strong and reproducible mitotic phenotypes, which are unlikely to be elicited by an OT sequence highly diluted by the excess of sequences homologous to the target gene. Second, we never found homology between any OT sequences present in the genes of the same phenocluster. Third, we found that where the data are available, the mitotic phenotypes observed in our screen match those seen in fly mutants and/or in previous RNAi experiments, regardless of whether OT sequences were present in the dsRNAs (31% of the dsRNAs that resulted in expected phenotype contained OT sequences). Finally, examination of 100 randomly chosen RNAs that did not elicit mitotic phenotypes showed that 40% of them contain OT sequences.

Individual gene sequences were amplified by PCR from a pool of cDNAs obtained from 5 different libraries: 4 embryonic libraries from 0–4, 4–8, 8–12 and 12–24 hr embryos; and an imaginal disc library, all kindly provided by N. Brown [62]. If this cDNA pool did not provide the desired PCR product, DNA was amplified from genomic DNA. The primers used in the PCR reactions were 35 nt long and all contained a 5′ T7 RNA polymerase binding site (5′-TAATACGACTCACTATAGGGAGG-3′) joined to a gene-specific sequence. The primers used to amplify the 155 mitotic genes detected in the screen are reported in Table S4. dsRNA synthesis and analysis were performed as previously described [1].

### Cell cultures and RNAi Treatments

S2 cells were cultured at 25°C in Shields and Sang M3 medium (Sigma) supplemented with 10% heat-inactivated fetal bovine serum (FBS, Invitrogen). RNAi treatments were carried out according to Somma et al. [1]. 1×10^6^ cells were plated in 1 ml of serum-free medium in a well of a six-well culture dish (Sarstedt). Each culture was inoculated with 15 µg of dsRNA. After a 1 hr incubation at 25°C, 2 ml of medium supplemented with 15% FBS were added to each culture. Control cultures were prepared in the same way but without addition of dsRNA. Both RNA-treated and control cells were grown for 96 hr at 25°C, and then processed for cytological analyses.

### Cytological Procedures

RNAi treatments were performed using six-well plates. In a typical experiment, cells in 16 wells from three plates were treated with dsRNAs, while cells in the remaining two wells served as control. After four-day incubation with dsRNA, cells from 3 ml cultures were resuspended and processed in two ways. 2 ml of this suspension were centrifuged at 800 g for 5 min, washed in 10 ml PBS, and fixed for 7 min in 3 ml 3.7% formaldehyde in PBS. Fixed cells were spun down by centrifugation, resuspended in 500 µl PBS, and cytocentrifuged onto a clean slide using a Shandon cytocentrifuge at 900 rpm for 4 min. The slides were immersed in liquid nitrogen, washed in PBS, and incubated in PBT (PBS+0.1% TritonX-100) for 15 min, and then in PBS containing 3% BSA for 20 min. These preparations were immunostained using the following antibodies, all diluted 1∶100 in PBS: anti-α tubulin monoclonal DM1A (Sigma), rabbit anti-ZW10 [20], rabbit anti-cyclin B and anti-CENP-C (gifts of Christian Lehner, University of Bayreuth, Germany), and chicken anti-CID [18]. These primary antibodies were detected by incubation for 1 hr with FITC-conjugated anti-mouse IgG and Cy3-conjugated anti-rabbit IgG (Jackson Laboratories). All slides were mounted in Vectashield with DAPI (Vector) to stain DNA and reduce fluorescence fading.

To obtain metaphase chromosome preparations, 1 ml of cell suspension was left in its well and treated for 2 hr with colchicine (final concentration 10^−5^ M). Colchicine-treated cells were then centrifuged at 800 g for 5 min. Pelleted cells were washed in 10 ml PBS, spun down by centrifugation and resuspended in 5 ml hypotonic solution (0.5 M Na citrate) for 7 min. After further centrifugation, pelleted cells were fixed in 5 ml of methanol: acetic acid (3∶1), spun down again, and resuspended in the small volume of fixative left after the removal of supernatant. 10 µl of this suspension was dropped onto a microscope slide and air-dried. All slides were mounted in Vectashield with DAPI (Vector) to stain DNA.

All images were captured using a CoolSnap HQ CCD camera (Photometrics; Tucson, AZ) connected to a Zeiss Axioplan fluorescence microscope equipped with an HBO 100W mercury lamp as described previously [63]. Gray scale digital images were collected separately, converted to Photoshop format, pseudocolored, and merged.

### Phenotypic Analysis

We considered 18 major phenotypic traits indicated in the headings of Figures 2 and 3, and Table S5. In addition, we observed 13 relatively rare phenotypes that are reported under the OSD (other spindle defects) and OMD (other mitotic defects) headings of Figures 2 and 3, and Table S5. Each individual phenotypic trait was considered as genuine when its frequency in a dsRNA-treated sample was significantly higher than the frequency of that trait in controls with p<0.001, using the χ2 contingency test. The same trait was considered strong when its frequency was at least threefold (in the case of chromosome aberrations) or fivefold (in all the other cases) the control frequency. A phenotypic trait was considered weak when its frequency was significantly different from the control (with p<0.001), but below the above thresholds.

Cells from control wells without dsRNA were systematically compared with cells treated with dsRNAs that do not elicit mitotic phenotypes. We never observed phenotypic differences between any of these cells, indicating that none of the aberrant traits we detected was due simply to the addition of random dsRNA sequences to the culture.

Most of the mitotic genes identified in our screen are highly conserved and have putative human orthologs. The known function of these *Drosophila* genes and their human counterparts are reported in Table S6, together with the supporting references.

## Supporting Information

Figure S1 Precocious sister chromatid separation and defective chromosome segregation after RNAi for *Bub3*. Ctr, control; c-meta, colchicine/hypotonic-treated metaphase chromosomes. Note in addition the elongated and bent ana/telophase spindles. Scale bar, 5 *μ*m.
(2.10 MB TIF)

Figure S2 Lack of sister chromatid separation after RNAi for CS3 genes that encode kinetochore components. Note in addition the elongated and bent ana/telophase-like spindles in *l(1)G023/CG1558* and *CG9938/hec1/Ndc80* RNAi cells. Scale bar, 5 *μ*m.
(4.78 MB TIF)

Figure S3 Lack of sister chromatid separation after RNAi for CS3 genes that encode splicing factors. Similar to other cells in the CS3 phenocluster (Figure S2), ana/telophase-like spindles are elongated and bent in *CG3605*, *CG5931*, and *CG6015* RNAi cells. Scale bar, 5 *μ*m.
(4.26 MB TIF)

Figure S4 Examples of mitotic figures observed after RNAi for the splicing factor gene *CG10418*. *CG10418* knockdown results in a failure of sister chromatid separation. In some cells with ana/telophase-like spindles, the chromosomes appear to migrate to the poles, while in others the chromosomes remain at the center of the cell. *CG10418* was assigned to the CS3 phenocluster because the first type of cells is more frequent than the second one. Scale bar, 5 µm.
(2.59 MB TIF)

Figure S5 RNAi for genes of the CS3 group results in ana/telophase-like cells that assemble irregular actin-based contractile rings despite the failure of sister chromatid separation. Note that the actin rings form in regions that contain bundled microtubules. Scale bar, 5 µm.
(2.81 MB TIF)

Figure S6 Examples of mitotic cells observed after RNAi for genes of the CS4 phenocluster. The chromosomes remain at the cell equator while the spindle elongates to assume an ana/telophase-like morphology. The chromosomes at the center of the cell often decondense as occurs during normal telophase. Scale bar, 5 *μ*m.
(5.25 MB TIF)

Figure S7 Defects in chromosome segregation observed after RNAi for genes of the CS5 phenocluster. Ctr, control; c-meta, colchicine/hypotonic-treated metaphase chromosomes. Note that in the c-metaphase (c-meta) from *fzy* RNAi cells, chromosomes are extremely condensed and the sister chromatids are separated. However, sister chromatid separation (SCS) is not observed in *fzy* RNAi cells with normally condensed chromosomes. We thus consider SCS as a secondary consequence of the excessive chromosome condensation, rather than a direct effect of gene product depletion. In support of this idea, RNAi for genes of the CS2 phenocluster (Figure S1) causes sister chromatid separation regardless of the degree of chromosome condensation. Scale bar, 5 µm.
(5.70 MB TIF)

Figure S8 Pole-to-pole spindle lengths (mean±SE) of ana/telophase figures observed in the CS1-CS5 phenoclusters. The ana/telophase spindles observed in all the RNAi experiments included in the graph are significantly longer (p<0.001 in Student's t-test) than control (ctr) spindles.
(1.01 MB TIF)

Figure S9 RNAi for genes of the CC1 phenocluster results both in a lack of sister chromatid cohesion in the heterochromatic regions of the chromosomes and in defective chromosome segregation. Ctr, control; c-meta, colchicine/hypotonic-treated metaphase chromosomes. Scale bar, 5 µm.
(3.47 MB TIF)

Figure S10 Defects in chromosome condensation and segregation observed after RNAi for genes of the CC2 phenocluster. Ctr, control; c-meta, colchicine/hypotonic-treated metaphase chromosomes. Note the extensive chromatin bridges in the ana/telophase figures. Scale bar, 5 µm.
(3.37 MB TIF)

Figure S11 Monopolar spindles, short spindles and defective chromosome segregation observed after RNAi for *msps* and *tho2* (SA1 phenocluster). The telophase-like figures of *msps* RNAi cells display abnormally long astral microtubules. Scale bar, 5 *μ*m.
(3.48 MB TIF)

Figure S12 Extremely short spindles and monopolar spindles observed after RNAi for *eIF-3p66* (translation factor) and *CG4865*. Anaphases are very rare, suggesting that the tiny spindles in these cells are unable to support chromosome segregation. Scale bar, 5 µm.
(2.75 MB TIF)

Figure S13 Disorganized spindles with low microtubule density observed after RNAi for genes of the SA2 phenocluster. The elongated ana/telophase-like spindles contain scattered chromosomes with unseparated sister chromatids. Scale bar, 5 µm.
(2.06 MB TIF)

Figure S14 Anastral and broad spindle poles observed after RNAi for *abnormal spindle (asp)*. Scale bar, 5 µm.
(1.45 MB TIF)

Figure S15 RNAi phenotypes observed in the heterogeneous SA4 group. In most *cdc2* RNAi cells, the chromosomes remain at the center of the cells (as seen after RNAi for the CS4 group genes) and the centrosomes detach from the spindle poles. In *Klp61F* RNAi cells, spindles are either monopolar or monastral bipolar. In cells with monastral bipolar spindles, chromosome segregation does not occur and the chromosomes remain associated with the astral poles. Scale bar, 5 µm.
(3.93 MB TIF)

Figure S16 Abnormal chromosome condensation and multiple mitotic defects observed after RNAi for *Borr* (SC1) and *Myb* (SC2). Ctr, control; c-meta, colchicine/hypotonic-treated metaphase chromosomes. Note that in *Borr* RNAi cells chromosomes are abnormally long and irregularly condensed. In contrast, after RNAi for *Myb*, chromosomes are overcontracted and swollen with no resolution between sister chromatids. Scale bar, 5 µm.
(3.48 MB TIF)

Table S1 Coexpression analysis-based ranking of Drosophila genes.
(0.21 MB PDF)

Table S2 Distribution of 164 known mitotic genes in coexpression lists with cid, glu, eb1, zw10, ida and sti.
(0.03 MB PDF)

Table S3 Individual ranks of 164 mitotic genes in coexpression lists with cid, glu, eb1, zw10, ida and sti.
(0.06 MB PDF)

Table S4 List of the 155 mitotic genes detected in the screen and primers used for dsRNA synthesis.
(0.05 MB PDF)

Table S5 Characterization of the RNAi phenotypes elicited by the genes detected in the screen.
(0.10 MB PDF)

Table S6 Previously known functions of Drosophila mitotic genes and their putative human orthologs.
(0.23 MB PDF)

Table S7 Comparison with the Goshima et al. (2007) screen.
(0.04 MB PDF)

Text S1 Comparison with previous screens.
(0.05 MB DOC)
