# Supplementary figures and images for: Identification of Drosophila Mitotic Genes by Combining Co-Expression Analysis and RNA Interference
Source: PLoS Genet. 2008 Jul 18;4(7):e1000126. doi: 10.1371/journal.pgen.1000126 (PMC2537813; doi:10.1371/journal.pgen.1000126)

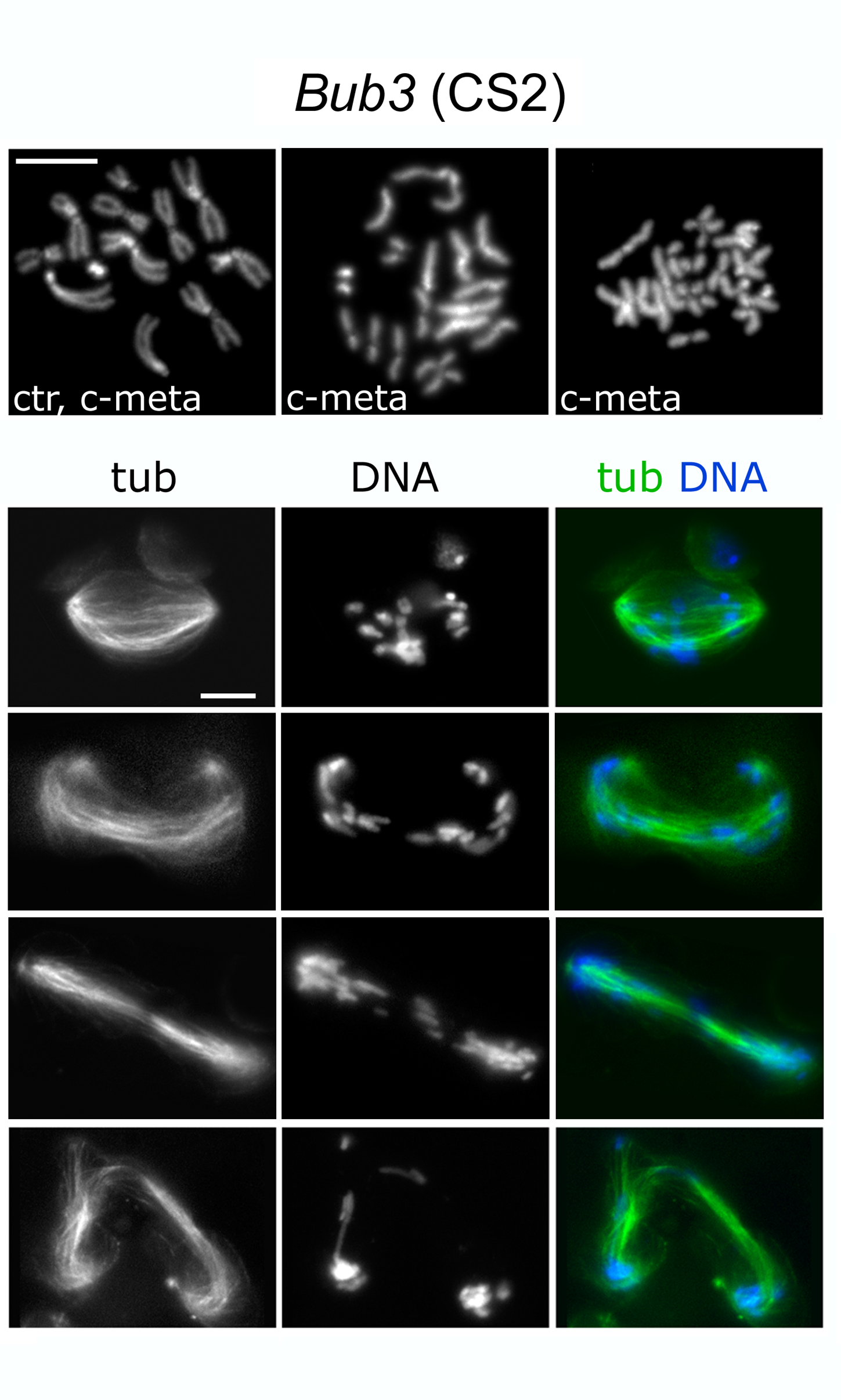

Supplement: Figure S1 — Precocious sister chromatid separation and defective chromosome segregation after RNAi for Bub3. Ctr, control; c-meta, colchicine/hypotonic-treated metaphase chromosomes. Note in addition the elongated and bent ana/telophase spindles. Scale bar, 5 μm. (2.10 MB TIF) [file pgen.1000126.s001.tif]

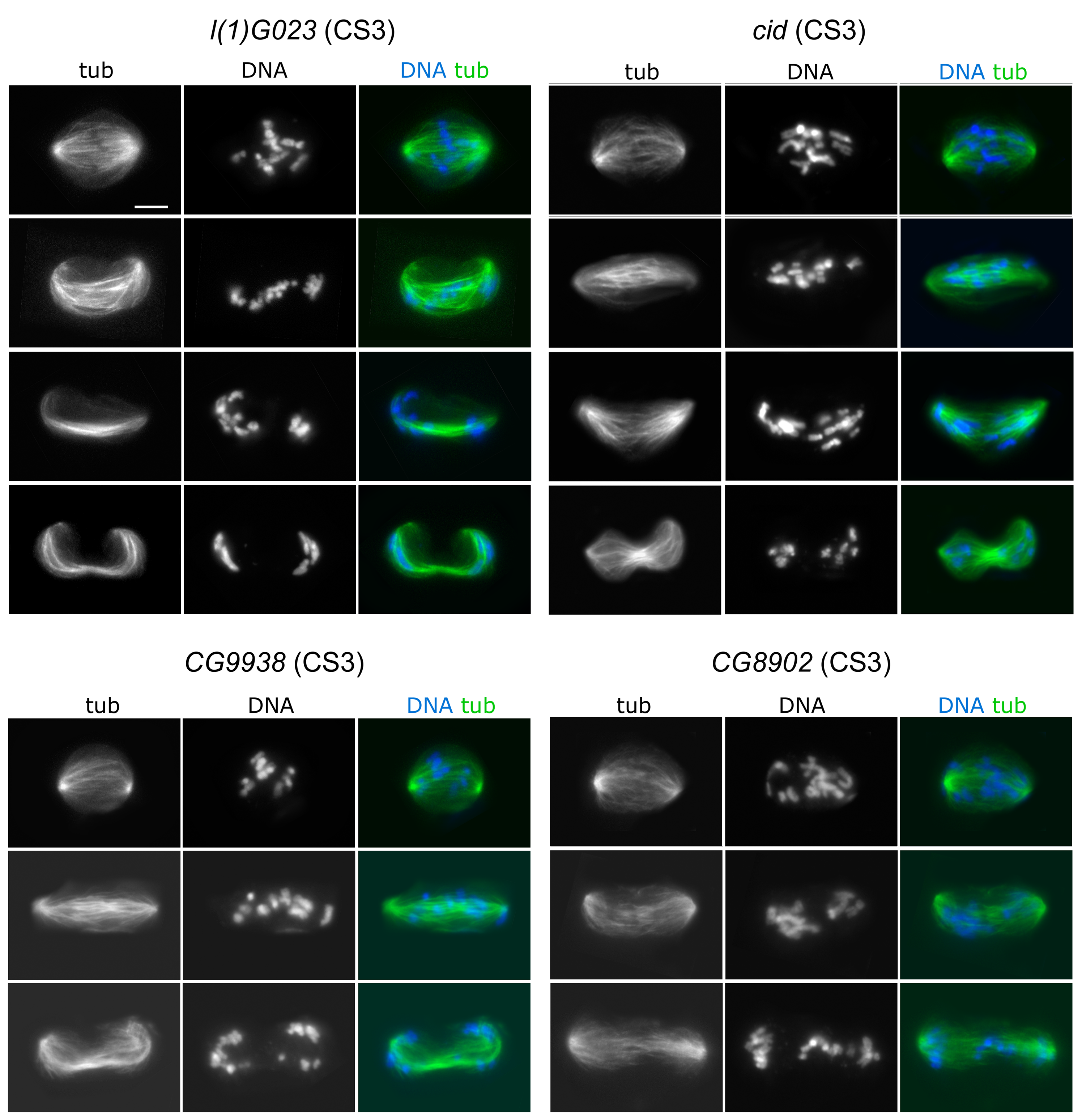

Supplement: Figure S2 — Lack of sister chromatid separation after RNAi for CS3 genes that encode kinetochore components. Note in addition the elongated and bent ana/telophase-like spindles in l(1)G023/CG1558 and CG9938/hec1/Ndc80 RNAi cells. Scale bar, 5 μm. (4.78 MB TIF) [file pgen.1000126.s002.tif]

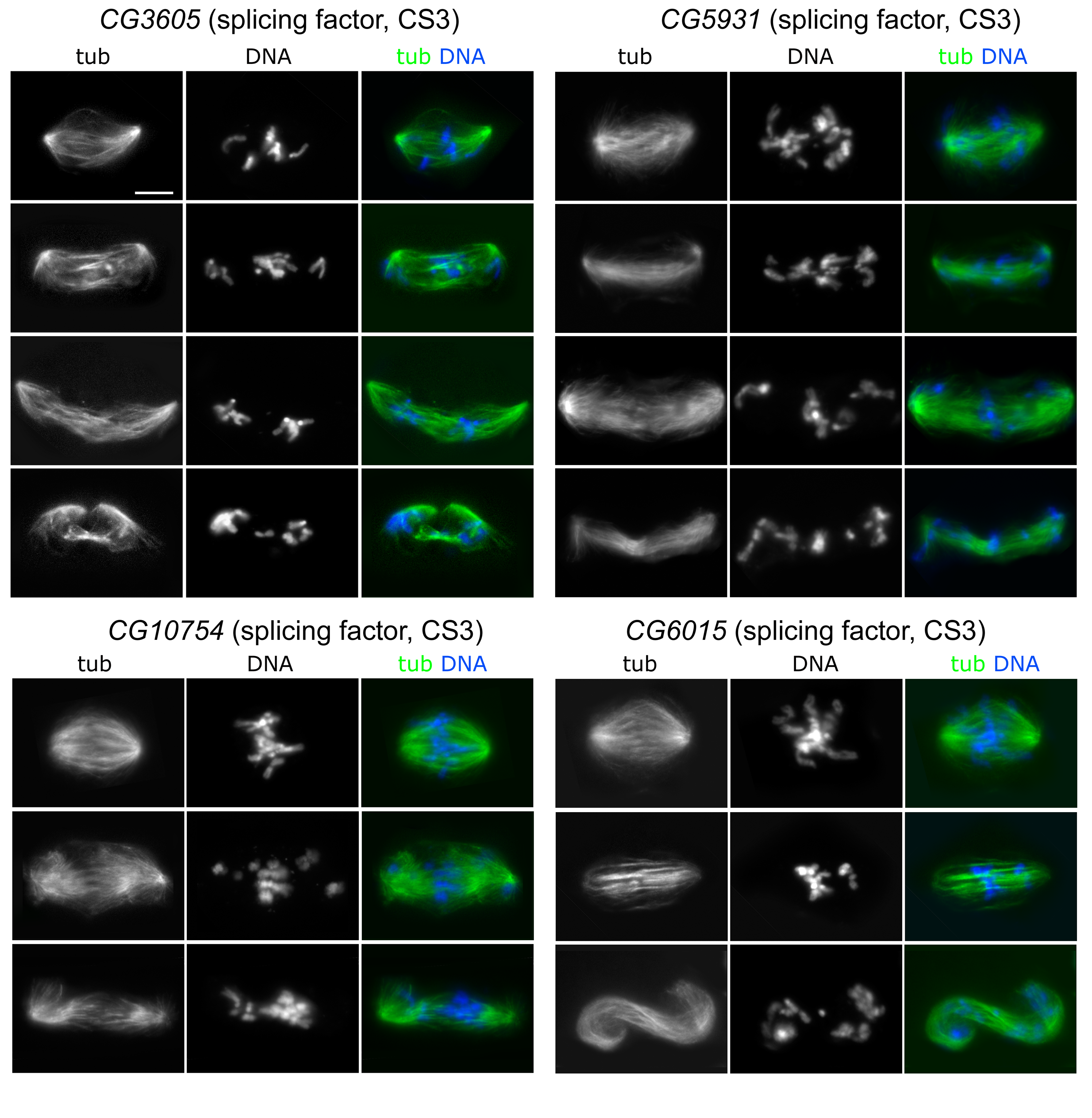

Supplement: Figure S3 — Lack of sister chromatid separation after RNAi for CS3 genes that encode splicing factors. Similar to other cells in the CS3 phenocluster (Figure S2), ana/telophase-like spindles are elongated and bent in CG3605, CG5931, and CG6015 RNAi cells. Scale bar, 5 μm. (4.26 MB TIF) [file pgen.1000126.s003.tif]

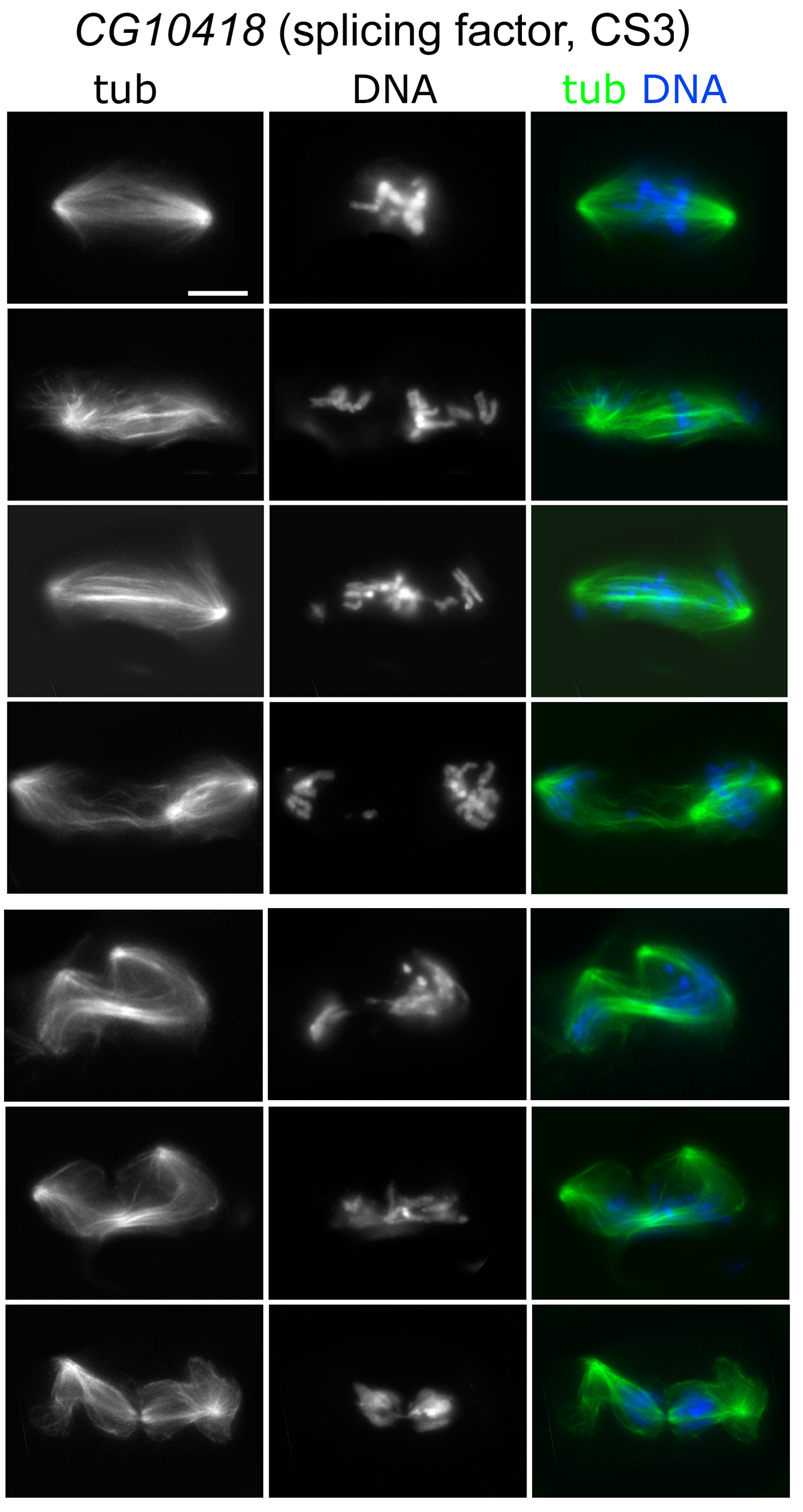

Supplement: Figure S4 — Examples of mitotic figures observed after RNAi for the splicing factor gene CG10418. CG10418 knockdown results in a failure of sister chromatid separation. In some cells with ana/telophase-like spindles, the chromosomes appear to migrate to the poles, while in others the chromosomes remain at the center of the cell. CG10418 was assigned to the CS3 phenocluster because the first type of cells is more frequent than the second one. Scale bar, 5 µm. (2.59 MB TIF) [file pgen.1000126.s004.tif]

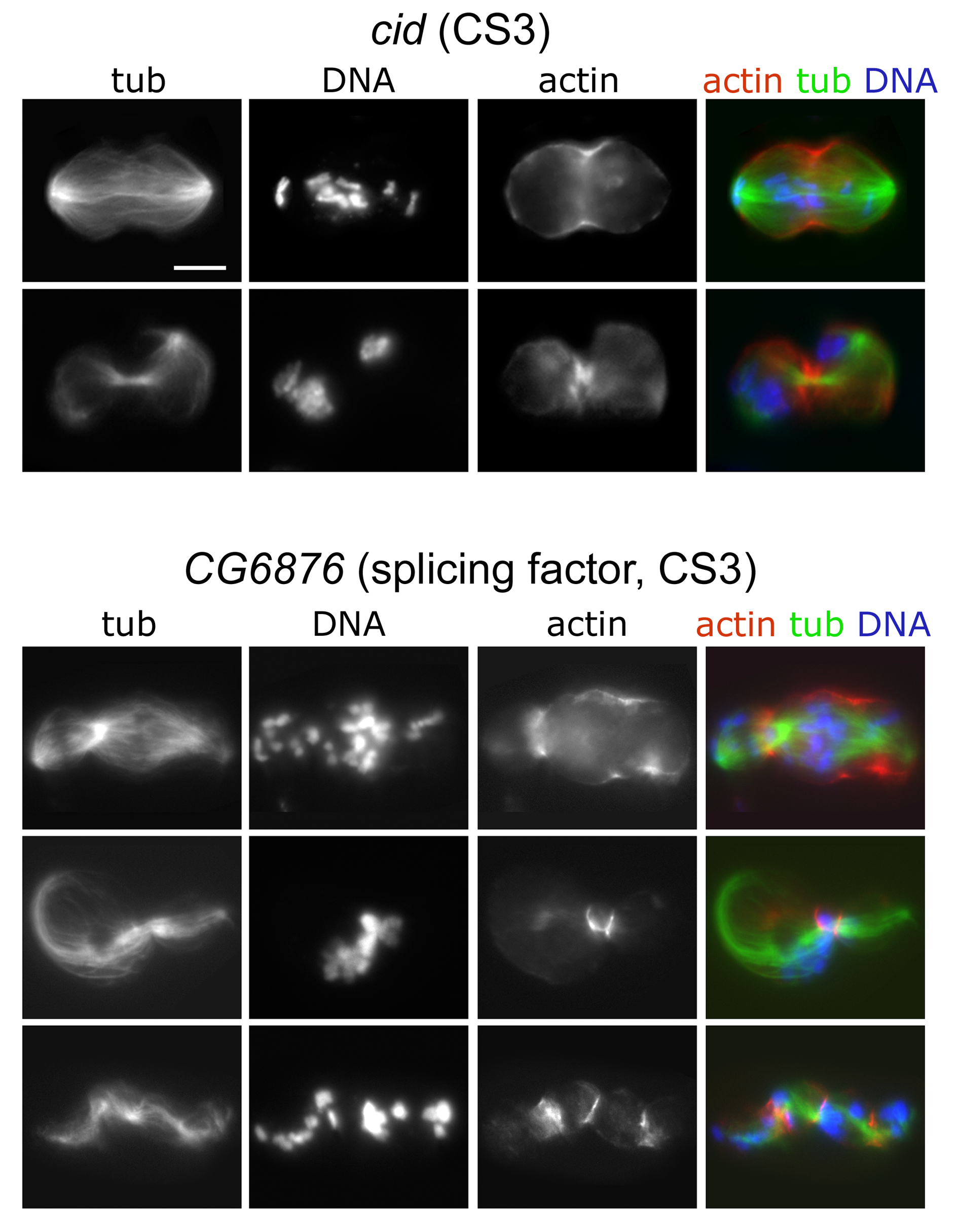

Supplement: Figure S5 — RNAi for genes of the CS3 group results in ana/telophase-like cells that assemble irregular actin-based contractile rings despite the failure of sister chromatid separation. Note that the actin rings form in regions that contain bundled microtubules. Scale bar, 5 µm. (2.81 MB TIF) [file pgen.1000126.s005.tif]

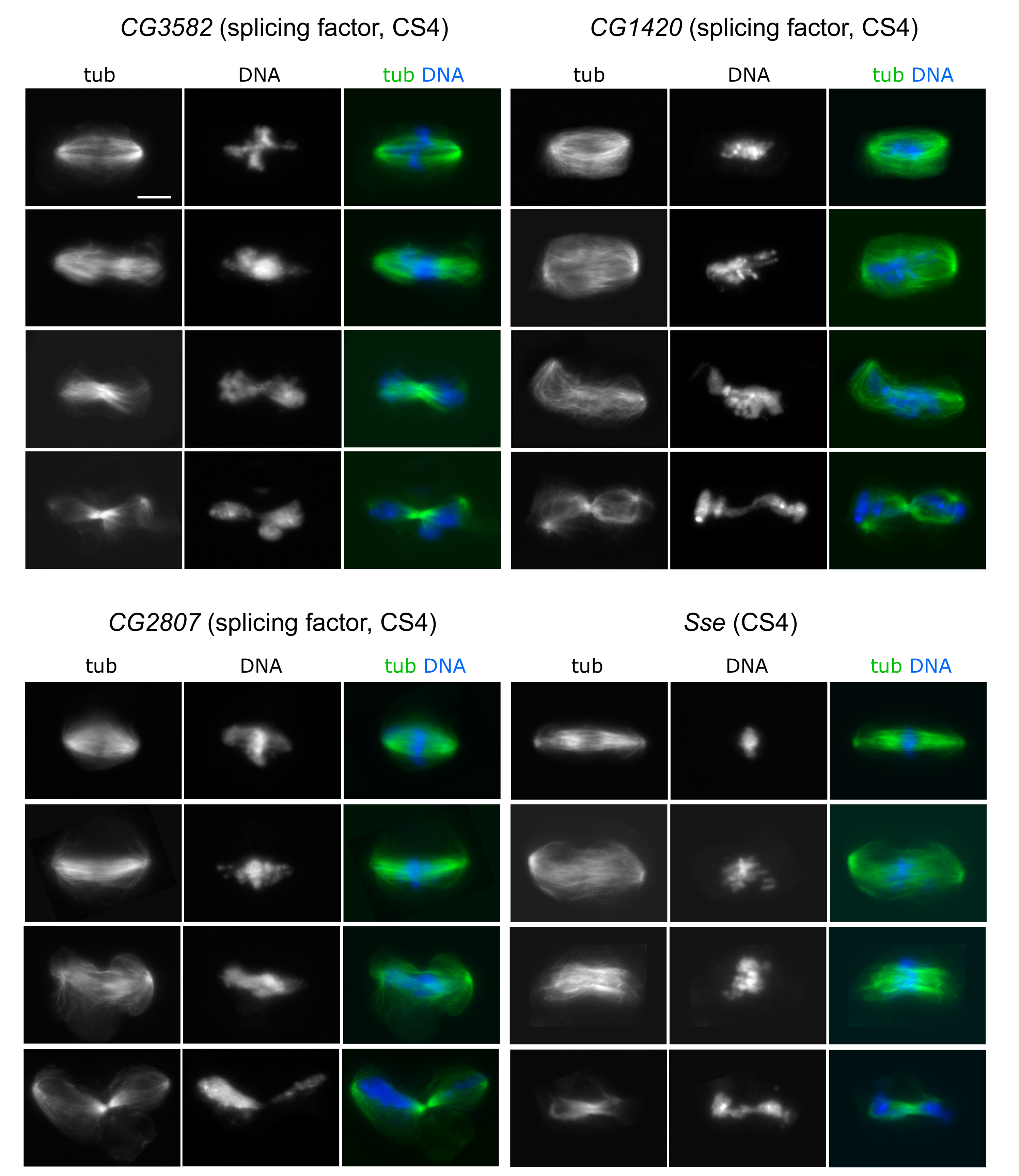

Supplement: Figure S6 — Examples of mitotic cells observed after RNAi for genes of the CS4 phenocluster. The chromosomes remain at the cell equator while the spindle elongates to assume an ana/telophase-like morphology. The chromosomes at the center of the cell often decondense as occurs during normal telophase. Scale bar, 5 μm. (5.25 MB TIF) [file pgen.1000126.s006.tif]

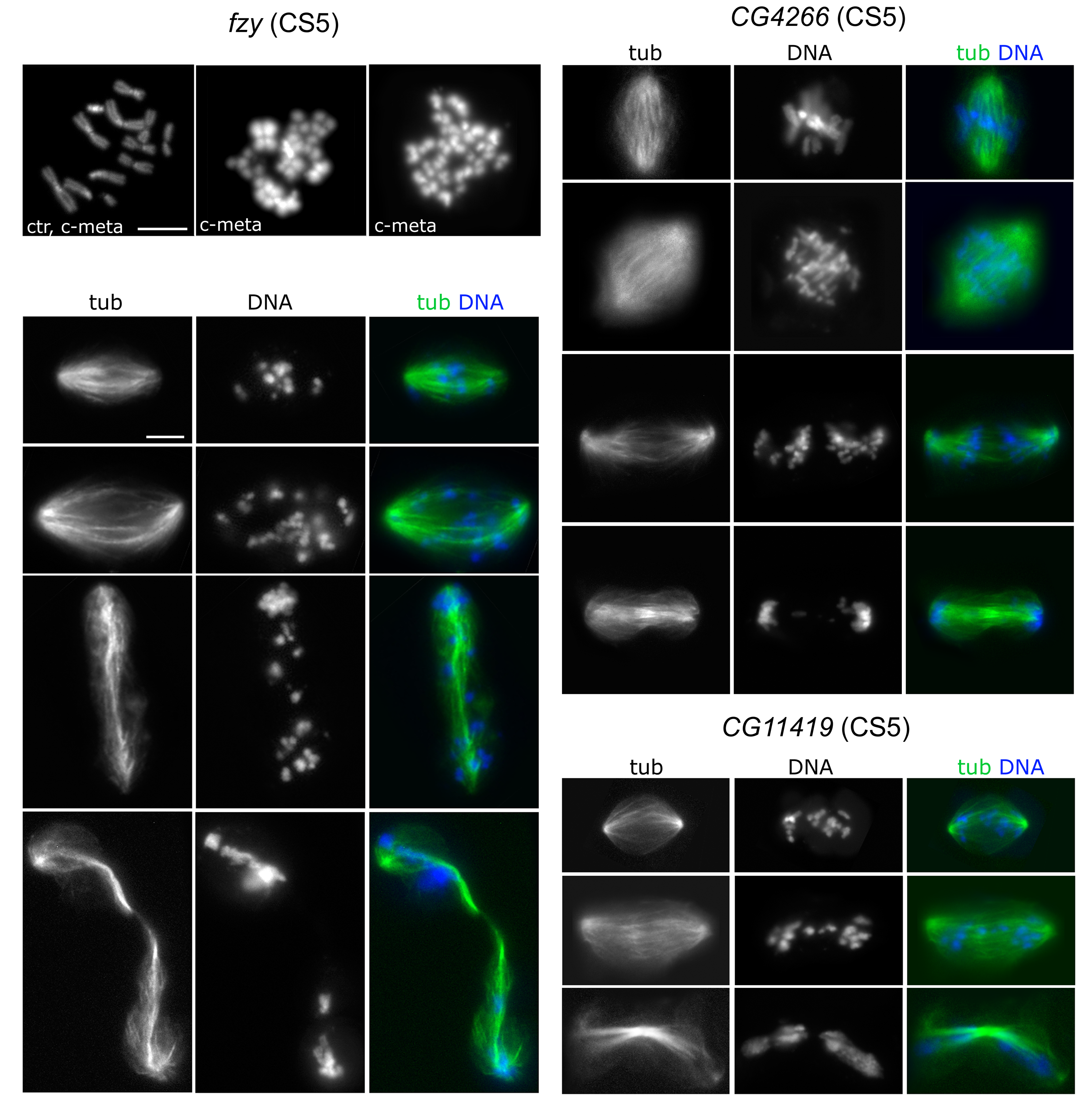

Supplement: Figure S7 — Defects in chromosome segregation observed after RNAi for genes of the CS5 phenocluster. Ctr, control; c-meta, colchicine/hypotonic-treated metaphase chromosomes. Note that in the c-metaphase (c-meta) from fzy RNAi cells, chromosomes are extremely condensed and the sister chromatids are separated. However, sister chromatid separation (SCS) is not observed in fzy RNAi cells with normally condensed chromosomes. We thus consider SCS as a secondary consequence of the excessive chromosome condensation, rather than a direct effect of gene product depletion. In support of this idea, RNAi for genes of the CS2 phenocluster (Figure S1) causes sister chromatid separation regardless of the degree of chromosome condensation. Scale bar, 5 µm. (5.70 MB TIF) [file pgen.1000126.s007.tif]

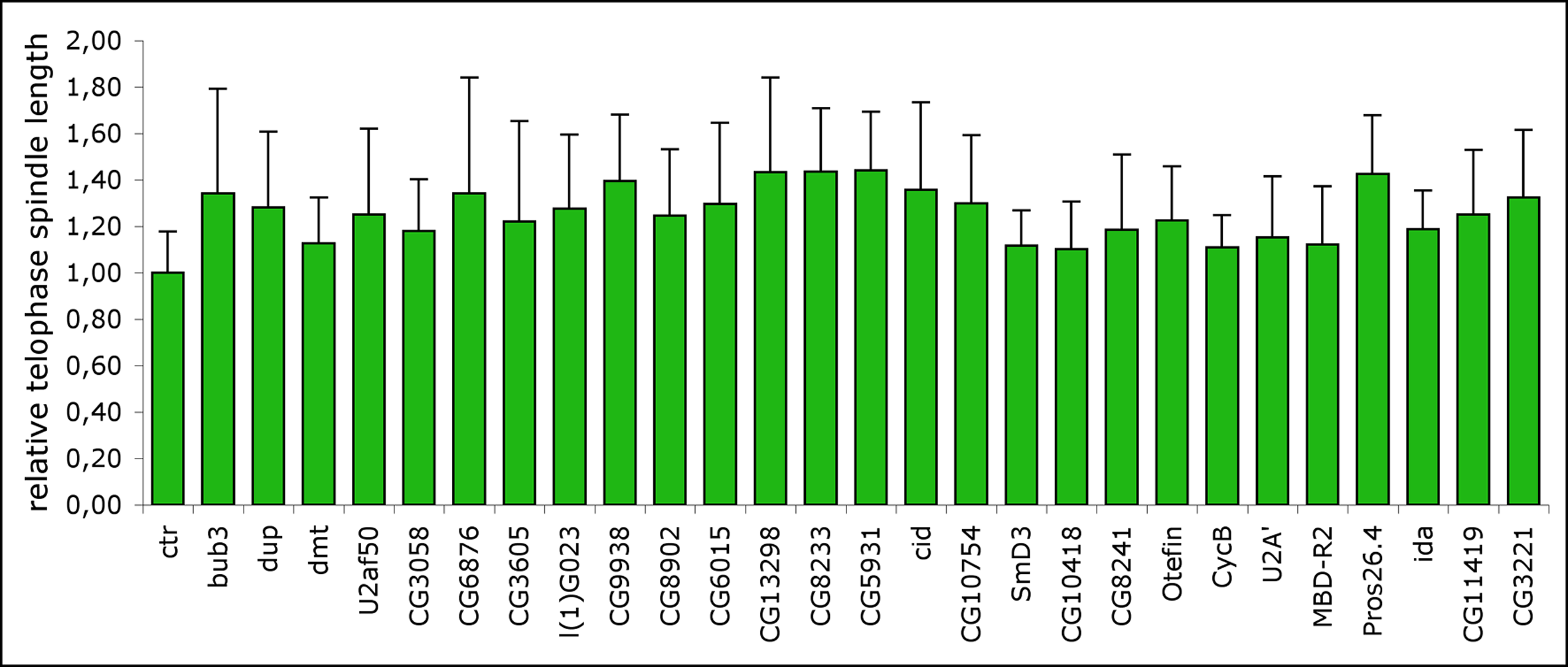

Supplement: Figure S8 — Pole-to-pole spindle lengths (mean±SE) of ana/telophase figures observed in the CS1-CS5 phenoclusters. The ana/telophase spindles observed in all the RNAi experiments included in the graph are significantly longer (p<0.001 in Student's t-test) than control (ctr) spindles. (1.01 MB TIF) [file pgen.1000126.s008.tif]

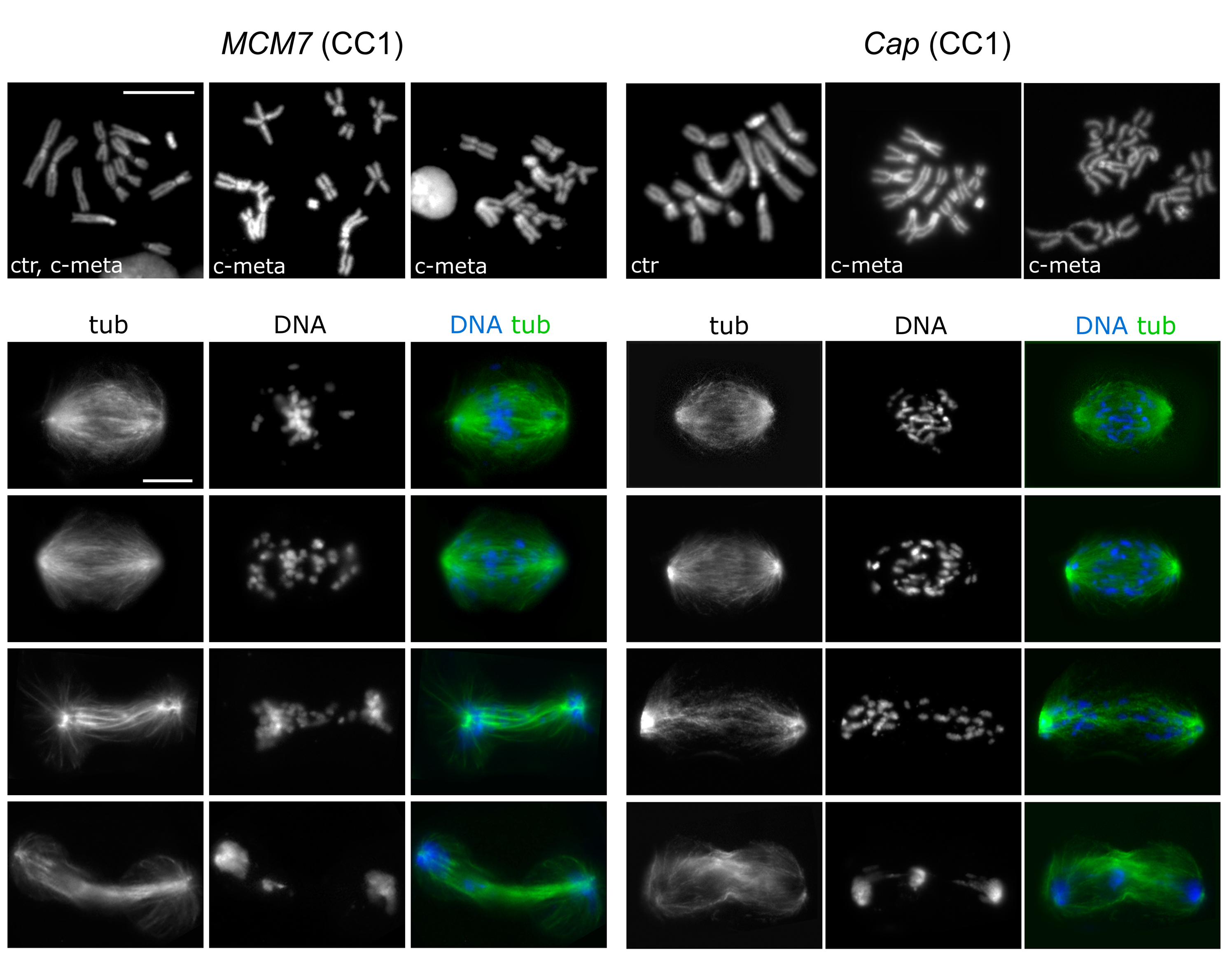

Supplement: Figure S9 — RNAi for genes of the CC1 phenocluster results both in a lack of sister chromatid cohesion in the heterochromatic regions of the chromosomes and in defective chromosome segregation. Ctr, control; c-meta, colchicine/hypotonic-treated metaphase chromosomes. Scale bar, 5 µm. (3.47 MB TIF) [file pgen.1000126.s009.tif]

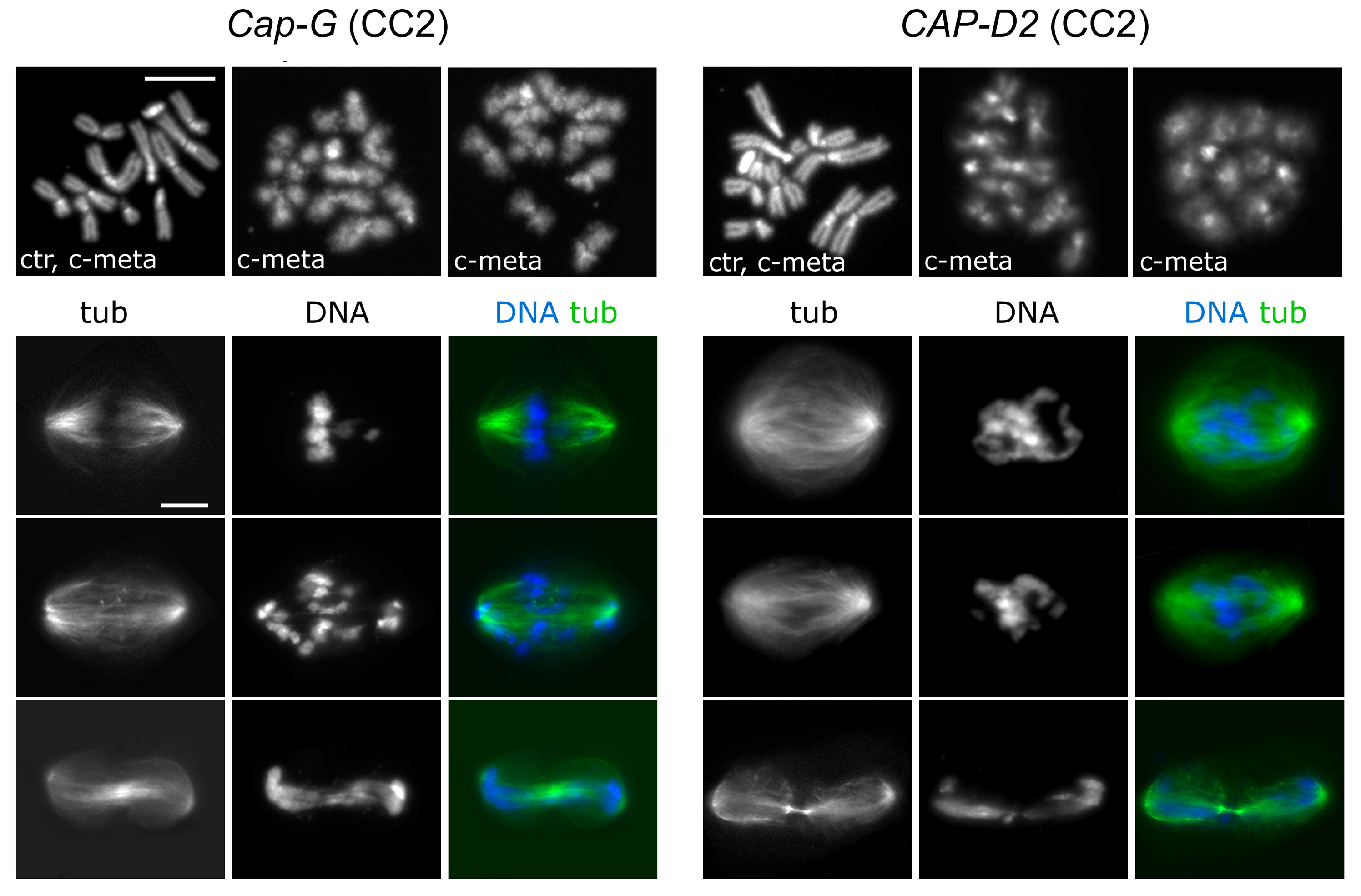

Supplement: Figure S10 — Defects in chromosome condensation and segregation observed after RNAi for genes of the CC2 phenocluster. Ctr, control; c-meta, colchicine/hypotonic-treated metaphase chromosomes. Note the extensive chromatin bridges in the ana/telophase figures. Scale bar, 5 µm. (3.37 MB TIF) [file pgen.1000126.s010.tif]

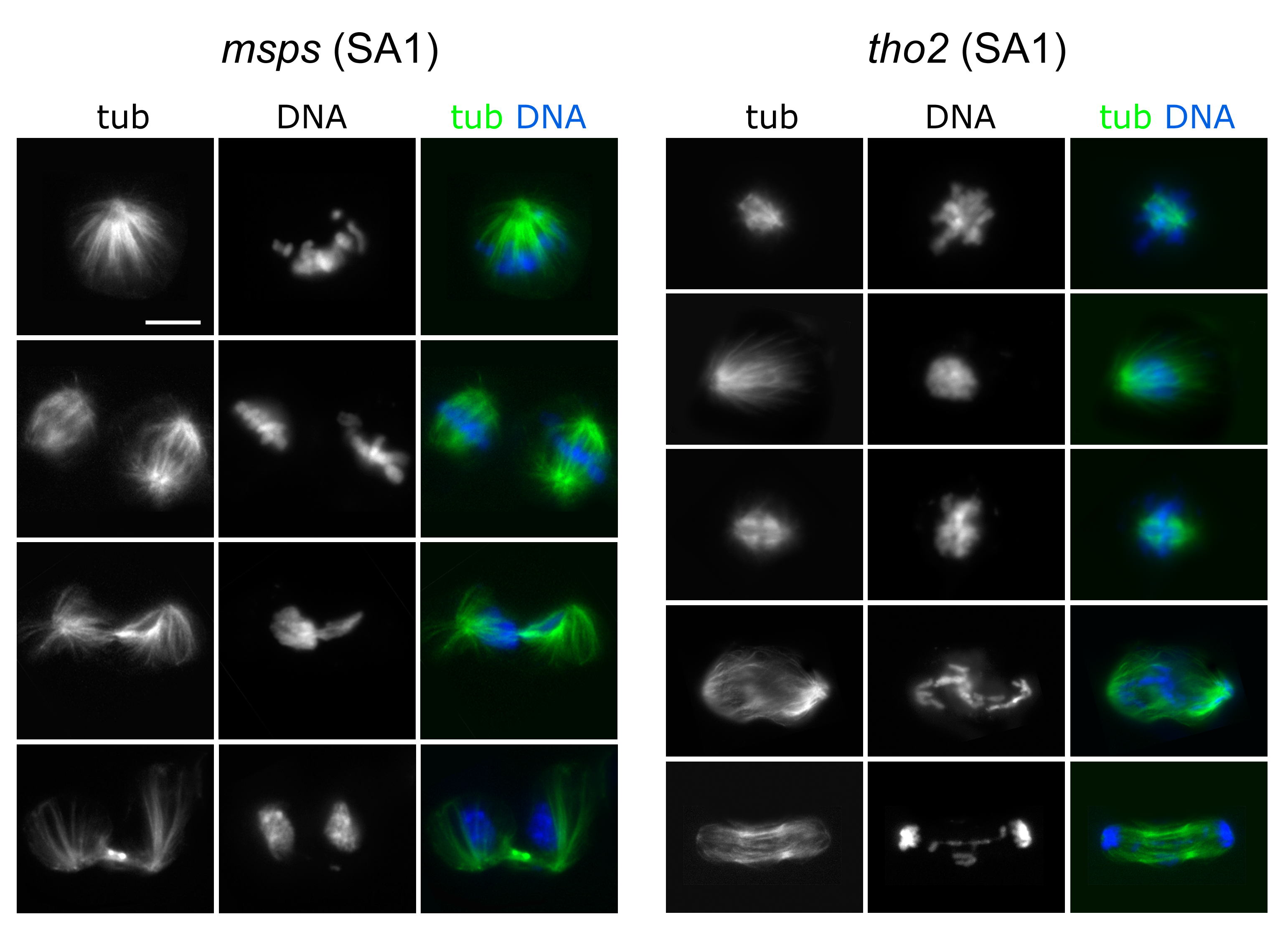

Supplement: Figure S11 — Monopolar spindles, short spindles and defective chromosome segregation observed after RNAi for msps and tho2 (SA1 phenocluster). The telophase-like figures of msps RNAi cells display abnormally long astral microtubules. Scale bar, 5 μm. (3.48 MB TIF) [file pgen.1000126.s011.tif]

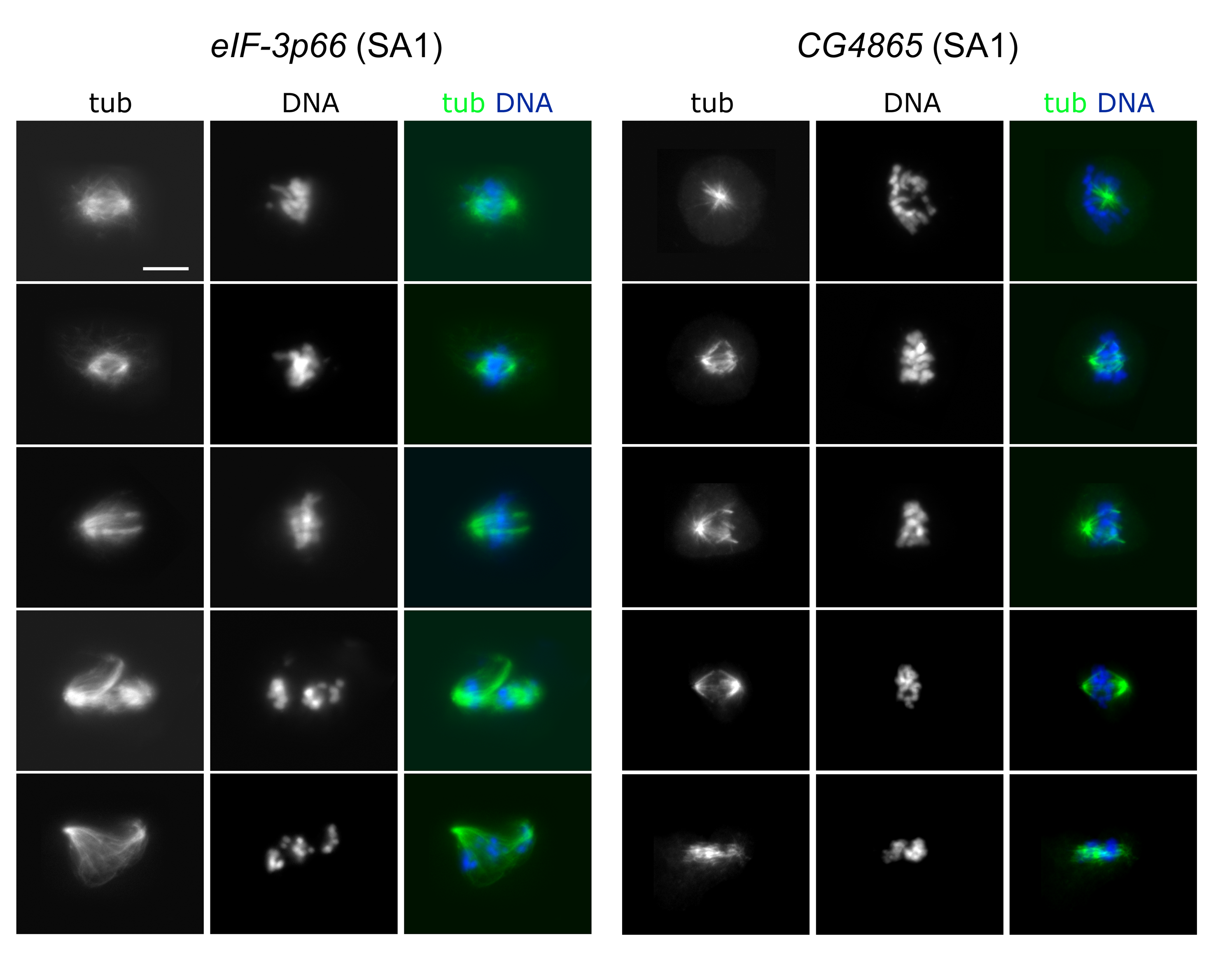

Supplement: Figure S12 — Extremely short spindles and monopolar spindles observed after RNAi for eIF-3p66 (translation factor) and CG4865. Anaphases are very rare, suggesting that the tiny spindles in these cells are unable to support chromosome segregation. Scale bar, 5 µm. (2.75 MB TIF) [file pgen.1000126.s012.tif]

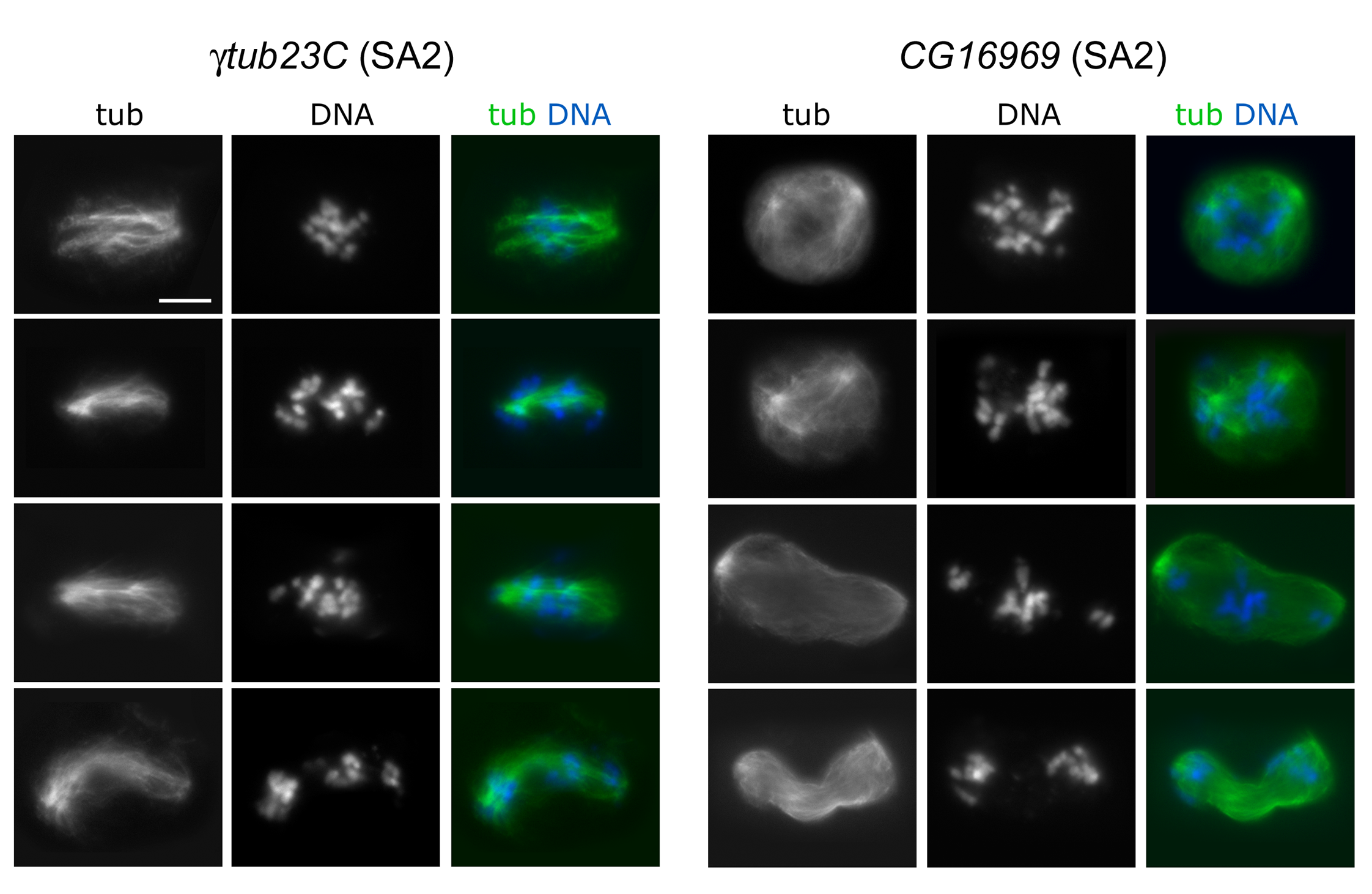

Supplement: Figure S13 — Disorganized spindles with low microtubule density observed after RNAi for genes of the SA2 phenocluster. The elongated ana/telophase-like spindles contain scattered chromosomes with unseparated sister chromatids. Scale bar, 5 µm. (2.06 MB TIF) [file pgen.1000126.s013.tif]

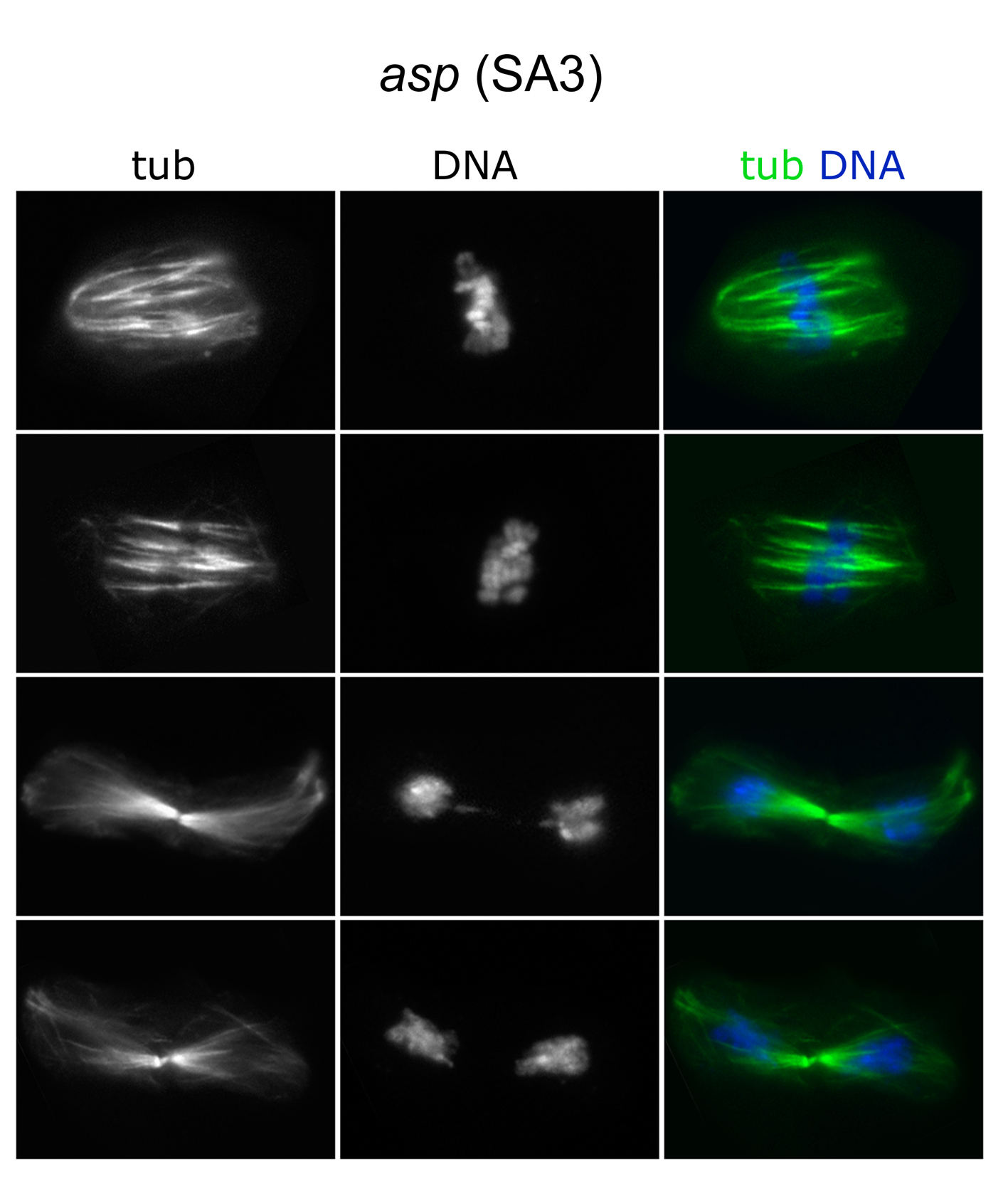

Supplement: Figure S14 — Anastral and broad spindle poles observed after RNAi for abnormal spindle (asp). Scale bar, 5 µm. (1.45 MB TIF) [file pgen.1000126.s014.tif]

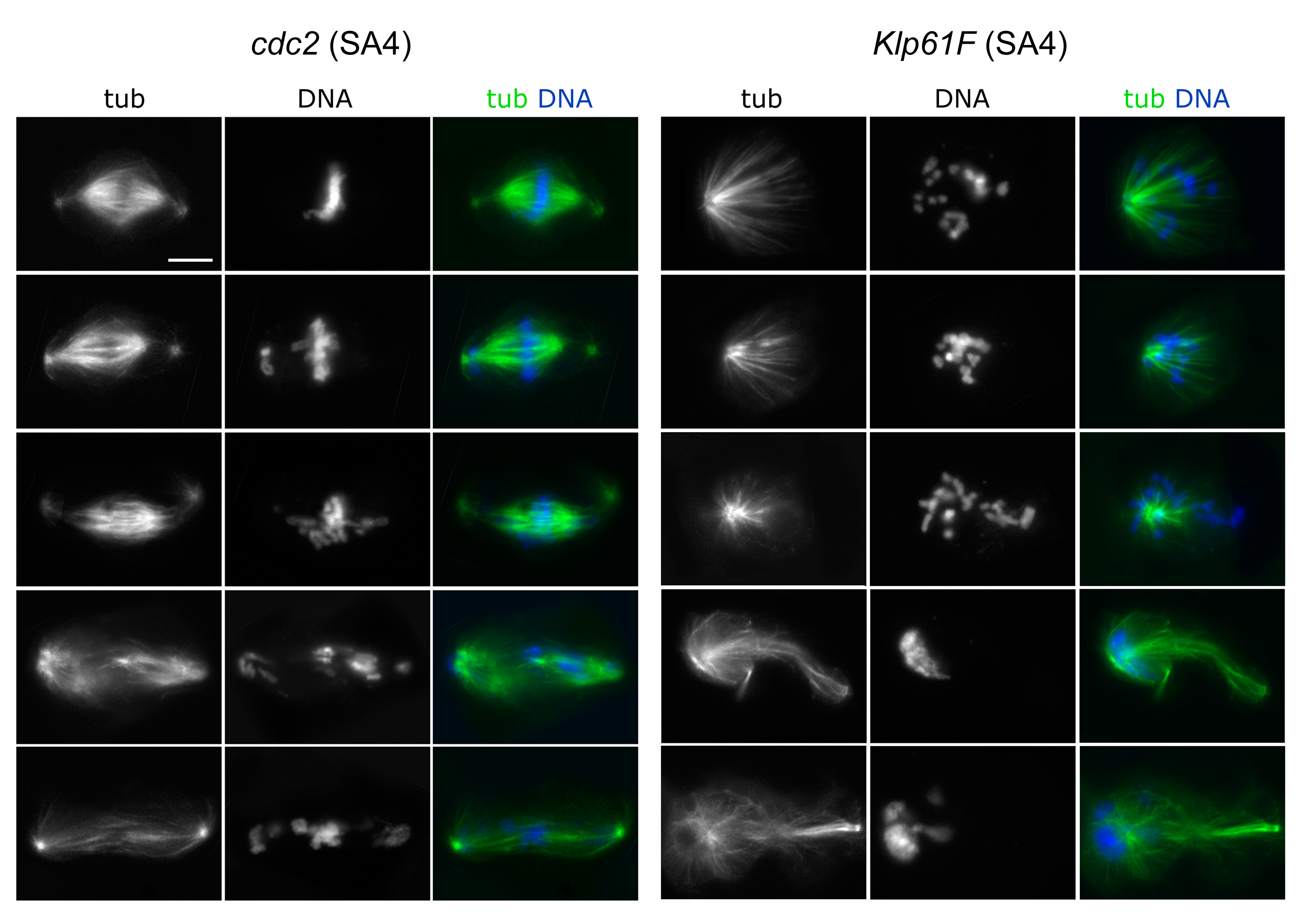

Supplement: Figure S15 — RNAi phenotypes observed in the heterogeneous SA4 group. In most cdc2 RNAi cells, the chromosomes remain at the center of the cells (as seen after RNAi for the CS4 group genes) and the centrosomes detach from the spindle poles. In Klp61F RNAi cells, spindles are either monopolar or monastral bipolar. In cells with monastral bipolar spindles, chromosome segregation does not occur and the chromosomes remain associated with the astral poles. Scale bar, 5 µm. (3.93 MB TIF) [file pgen.1000126.s015.tif]

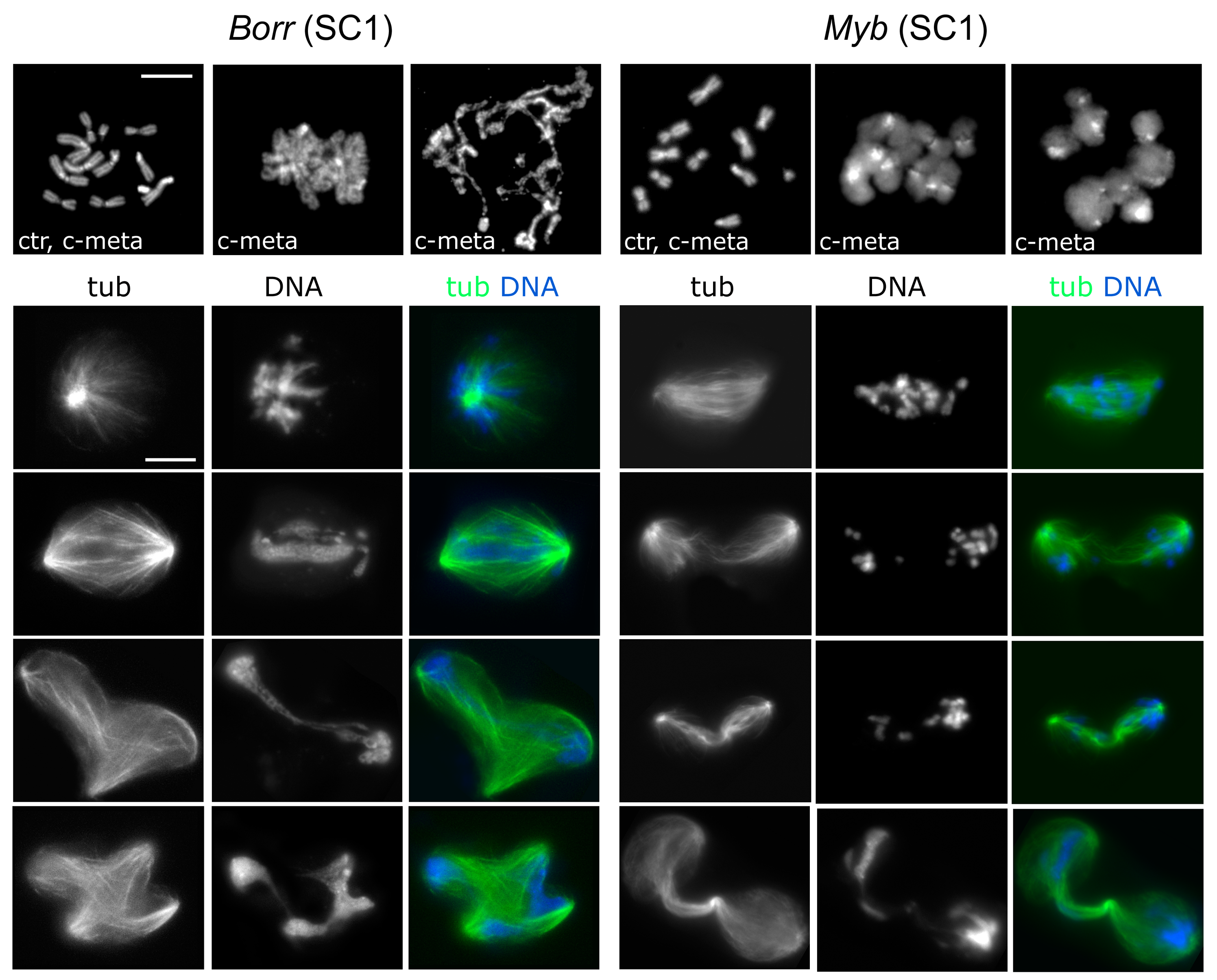

Supplement: Figure S16 — Abnormal chromosome condensation and multiple mitotic defects observed after RNAi for Borr (SC1) and Myb (SC2). Ctr, control; c-meta, colchicine/hypotonic-treated metaphase chromosomes. Note that in Borr RNAi cells chromosomes are abnormally long and irregularly condensed. In contrast, after RNAi for Myb, chromosomes are overcontracted and swollen with no resolution between sister chromatids. Scale bar, 5 µm. (3.48 MB TIF) [file pgen.1000126.s016.tif]
