# Supplementary material for: Identification of Drosophila Mitotic Genes by Combining Co-Expression Analysis and RNA Interference
Source: PLoS Genet. 2008 Jul 18;4(7):e1000126. doi: 10.1371/journal.pgen.1000126 (PMC2537813; doi:10.1371/journal.pgen.1000126)
Supplement: Table S1 — Coexpression analysis-based ranking of Drosophila genes. (0.21 MB PDF) [file pgen.1000126.s017.pdf]

Supplementary Table 1. Coexpression analysis-based ranking of *Drosophila* genes. Only the first 3000 genes are shown. RNAi was performed for genes ranked from 1 to 1056. Genes marked in grey were not assayed by RNAi.

Rank	CG	Gene symbol	Frequency in first 3000	Average Pearson
1	CG4082	Mcm5	6	0,9283
2	CG4976	Mes-4	6	0,9247
3	CG2092	scra	6	0,9244
4	CG9193	mus209	6	0,9240
5	CG15220	CG15220	6	0,9239
6	CG6502	E(z)	6	0,9238
7	CG7538	Mcm2	6	0,9236
8	CG7838	Bub1	6	0,9228
9	CG6743	Nup170	6	0,9220
10	CG5175	kuk	6	0,9220
11	CG8975	RnrS	6	0,9220
12	CG10159	BEAF-32	6	0,9219
13	CG1616	dpa	6	0,9218
14	CG12165	Incenp	6	0,9214
15	CG7581	Bub3	6	0,9212
16	CG14057	CG14057	6	0,9212
17	CG6386	ball	6	0,9210
18	CG4978	Mcm7	6	0,9206
19	CG1825	Map60	6	0,9204
20	CG9273	CG9273	6	0,9204
21	CG6546	Bap55	6	0,9201
22	CG9383	asf1	6	0,9200
23	CG12052	lola	6	0,9198
24	CG5352	SmB	6	0,9197
25	CG7269	Hel25E	6	0,9196
26	CG5313	RfC3	6	0,9196
27	CG4579	Nup154	6	0,9194
28	CG15736	Chrac-16	6	0,9193
29	CG10355	CG31697	6	0,9192
30	CG32721	CG32721	6	0,9191
31	CG12276	Aos1	6	0,9186
32	CG5363	cdc2	6	0,9186
33	CG5602	CG5602	6	0,9184
34	CG11886	Slbp	6	0,9183
35	CG1064	Snr1	6	0,9182
36	CG4303	Bap60	6	0,9182
37	CG4236	Caf1	6	0,9180
38	CG5519	Gbp	6	0,9179
39	CG4849	CG4849	6	0,9178
40	CG3183	geminin	6	0,9177
41	CG3178	Rrp1	6	0,9176
42	CG4584	dUTPase	6	0,9173
43	CG11397	glu	6	0,9172
44	CG3436	CG3436	6	0,9171
45	CG2158	CG2158	6	0,9171

46	CG8142	CG8142	6	0,9167
47	CG9401	mago	6	0,9167
48	CG6540	CG6540	6	0,9165
49	CG10364	msb1l	6	0,9164
50	CG11943	CG11943	6	0,9164
51	CG3918	CG3918	6	0,9162
52	CG5814	CycB3	6	0,9160
53	CG5971	CG5971	6	0,9159
54	CG3402	CG3402	6	0,9158
55	CG17153	CG17153	6	0,9156
56	CG15010	ago	6	0,9156
57	CG9135	CG9135	6	0,9154
58	CG5926	CG31184	6	0,9154
59	CG3430	CG3430	6	0,9151
60	CG8553	SelD	6	0,9151
61	CG7897	gp210	6	0,9149
62	CG8465	l(1)G0222	6	0,9148
63	CG14641	CG14641	6	0,9148
64	CG10498	cdc2c	6	0,9146
65	CG6897	CG6897	6	0,9144
66	CG6875	asp	6	0,9142
67	CG5899	CG5899	6	0,9142
68	CG6054	Su(fu)	6	0,9141
69	CG16792	DebB	6	0,9138
70	CG15610	CG33456	6	0,9138
71	CG9874	Tbp	6	0,9136
72	CG1828	dre4	6	0,9135
73	CG5220	CG5220	6	0,9135
74	CG7405	CycH	6	0,9134
75	CG7946	CG7946	6	0,9133
76	CG3238	CG3238	6	0,9133
77	CG7593	CG7593	6	0,9133
78	CG15929	lin-52	6	0,9132
79	CG13472	CG13472	6	0,9132
80	CG2615	ik2	6	0,9131
81	CG16941	CG16941	6	0,9130
82	CG7471	Rpd3	6	0,9129
83	CG12306	polo	6	0,9129
84	CG7055	dalao	6	0,9128
85	CG13096	CG13096	6	0,9128
86	CG8044	CG8044	6	0,9127
87	CG11207	feo	6	0,9125
88	CG4274	fzy	6	0,9125
89	CG4936	CG4936	6	0,9125
90	CG13879	CG13879	6	0,9124
91	CG4689	CG4877	6	0,9124
92	CG4039	Mcm6	6	0,9122
93	CG9998	U2af50	6	0,9121
94	CG9191	Klp61F	6	0,9121
95	CG1249	CG1249	6	0,9121
96	CG17252	BCL7-like	6	0,9121
97	CG10800	Rca1	6	0,9120
98	CG6874	l(3)neo26	6	0,9117

99	CG13921	CG13921	6	0,9116
100	CG6226	FK506-bp1	6	0,9115
101	CG5800	CG5800	6	0,9115
102	CG5581	Ote	6	0,9114
103	CG1101	Aly	6	0,9114
104	CG1966	Acf1	6	0,9114
105	CG8409	Su(var)205	6	0,9114
106	CG4299	Set	6	0,9113
107	CG6840	Rpb11	6	0,9113
108	CG7788	Ice	6	0,9113
109	CG1404	ran	6	0,9112
110	CG2097	CG2097	6	0,9112
111	CG8264	Bx42	6	0,9112
112	CG3642	Clp	6	0,9112
113	CG8571	smid	6	0,9111
114	CG5930	TfIIA-L	6	0,9110
115	CG1403	Sep1	6	0,9108
116	CG15141	CG15141	6	0,9107
117	CG3878	CG33525	6	0,9106
118	CG7210	kel	6	0,9105
119	CG3227	insv	6	0,9105
120	CG11979	Rpb5	6	0,9104
121	CG10417	CG10417	6	0,9104
122	CG7357	CG7357	6	0,9103
123	CG7262	CG7262	6	0,9103
124	CG7670	CG7670	6	0,9102
125	CG1957	CG1957	6	0,9101
126	CG2925	noi	6	0,9100
127	CG7741	CG7741	6	0,9100
128	CG18640	Amy-p	6	0,9100
129	CG31132	BRWD3	6	0,9099
130	CG6384	Cp190	6	0,9098
131	CG18740	mor	6	0,9097
132	CG3995	CG3995	6	0,9097
133	CG3018	lwr	6	0,9097
134	CG17078	CG17078	6	0,9097
135	CG14999	RfC40	6	0,9097
136	CG12892	Caf1-105	6	0,9097
137	CG9740	CG9740	6	0,9096
138	CG13345	RacGAP50C	6	0,9096
139	CG6695	CG6695	6	0,9096
140	CG7831	ncd	6	0,9095
141	CG11092	CG11092	6	0,9094
142	CG9241	Mcm10	6	0,9094
143	CG12752	Nxt1	6	0,9093
144	CG8591	CTCF	6	0,9093
145	CG12265	CG12265	6	0,9092
146	CG1542	CG1542	6	0,9091
147	CG10203	xl6	6	0,9089
148	CG5733	CG5733	6	0,9089
149	CG2091	CG2091	6	0,9088
150	CG17958	Sry-delta	6	0,9088
151	CG12359	Ulp1	6	0,9086

152	CG4206	Mcm3	6	0,9085
153	CG4118	nxf2	6	0,9085
154	CG11990	hyx	6	0,9083
155	CG7959	Bgb	6	0,9083
156	CG4980	CG4980	6	0,9082
157	CG1677	CG1677	6	0,9081
158	CG12006	CG12006	6	0,9081
159	CG5553	DNAprim	6	0,9081
160	CG13016	CG13016	6	0,9080
161	CG4654	Dp	6	0,9079
162	CG3738	Cks30A	6	0,9078
163	CG7704	Taf5	6	0,9077
164	CG8749	snRNP70K	6	0,9075
165	CG7885	RpII33	6	0,9075
166	CG10923	Klp67A	6	0,9074
167	CG6179	CG6179	6	0,9074
168	CG12050	CG12050	6	0,9073
169	CG6251	Nup62	6	0,9073
170	CG2890	PPP4R2r	6	0,9072
171	CG6474	e(y)1	6	0,9072
172	CG8877	prp8	6	0,9070
173	CG1622	CG1622	6	0,9069
174	CG2207	Df31	6	0,9068
175	CG7239	CG7239	6	0,9066
176	CG7041	HP1b	6	0,9066
177	CG6375	pit	6	0,9065
178	CG17950	HmgD	6	0,9065
179	CG17286	CG17286	6	0,9065
180	CG7137	CG7137	6	0,9064
181	CG11761	translin	6	0,9064
182	CG10061	l(3)s2214	6	0,9063
183	CG12113	l(1)G0095	6	0,9062
184	CG6819	mbo	6	0,9060
185	CG5941	CG5941	6	0,9060
186	CG4751	CG4751	6	0,9059
187	CG6049	CG6049	6	0,9059
188	CG2261	CstF-50	6	0,9059
189	CG7843	CG7843	6	0,9058
190	CG11770	lin	6	0,9058
191	CG5836	SF1	6	0,9057
192	CG1796	Tango4	6	0,9057
193	CG9999	RanGap	6	0,9056
194	CG7528	Uba2	6	0,9055
195	CG8648	Fen1	6	0,9054
196	CG3157	gammaTub23C	6	0,9054
197	CG7752	Z4	6	0,9054
198	CG1103	CG1103	6	0,9053
199	CG5940	CycA	6	0,9053
200	CG4639	CG31365	6	0,9053
201	CG8625	Iswi	6	0,9053
202	CG5452	dnk	6	0,9052
203	CG7281	CycC	6	0,9052
204	CG17269	Fancd2	6	0,9051

205	CG18608	prod	6	0,9050
206	CG13690	CG13690	6	0,9049
207	CG10522	sti	6	0,9046
208	CG4203	CG4203	6	0,9045
209	CG11856	Nup358	6	0,9044
210	CG17136	Rbp1	6	0,9044
211	CG9938	CG9938	6	0,9043
212	CG7671	CG7671	6	0,9043
213	CG11107	CG11107	6	0,9042
214	CG10851	B52	6	0,9042
215	CG1240	CG1240	6	0,9041
216	CG3058	CG3058	6	0,9041
217	CG16903	CG16903	6	0,9039
218	CG8013	Su(z)12	6	0,9039
219	CG8068	Su(var)2-10	6	0,9038
220	CG9302	CG9302	6	0,9036
221	CG12372	spt4	6	0,9035
222	CG9996	CG9996	6	0,9034
223	CG6258	RfC38	6	0,9034
224	CG3034	MED22	6	0,9034
225	CG4454	Borr	6	0,9033
226	CG2682	d4	6	0,9033
227	CG14228	Mer	6	0,9033
228	CG6990	HP1c	6	0,9033
229	CG6363	MRG15	6	0,9033
230	CG7035	Cbp80	6	0,9033
231	CG13427	CG13427	6	0,9031
232	CG6876	CG6876	6	0,9031
233	CG9797	CG9797	6	0,9031
234	CG10223	Top2	6	0,9030
235	CG5641	CG5641	6	0,9030
236	CG11723	CG11723	6	0,9029
237	CG6322	CG6322	6	0,9028
238	CG5083	Rbf2	6	0,9028
239	CG11120	CG11120	6	0,9028
240	CG8781	tsu	6	0,9026
241	CG6046	Bin1	6	0,9026
242	CG13400	D12	6	0,9025
243	CG13383	CSN8	6	0,9025
244	CG10728	vls	6	0,9024
245	CG2848	Trn-SR	6	0,9024
246	CG5745	CG5745	6	0,9023
247	CG17051	dod	6	0,9023
248	CG4455	CG4455	6	0,9022
249	CG5720	CG5720	6	0,9022
250	CG7744	CG7744	6	0,9022
251	CG3893	CG3893	6	0,9022
252	CG5422	Rox8	6	0,9022
253	CG4215	spel1	6	0,9022
254	CG1960	mu2	6	0,9022
255	CG7845	CG7845	6	0,9021
256	CG7971	CG7971	6	0,9021
257	CG6620	ial	6	0,9020

258	CG7376	CG7376	6	0,9020
259	CG9742	CG9742	6	0,9018
260	CG14781	CG14781	6	0,9018
261	CG18292	CG18292	6	0,9018
262	CG1109	CG1109	6	0,9018
263	CG4029	jumu	6	0,9017
264	CG7769	DDB1	6	0,9017
265	CG3605	CG3605	6	0,9016
266	CG3582	U2af38	6	0,9015
267	CG2905	Nipped	6	0,9015
268	CG16982	Roc1a	6	0,9012
269	CG1395	stg	6	0,9011
270	CG4738	CG4738	6	0,9011
271	CG11920	CG11920	6	0,9011
272	CG2980	thoc5	6	0,9011
273	CG3820	Nup214	6	0,9010
274	CG5785	thr	6	0,9008
275	CG6759	cdc16	6	0,9008
276	CG18528	CG18528	6	0,9008
277	CG9633	RpA-70	6	0,9007
278	CG3274	Bap170	6	0,9007
279	CG3797	CG3797	6	0,9007
280	CG1074	CG1074	6	0,9006
281	CG9915	CG9915	6	0,9006
282	CG3038	CG3038	6	0,9006
283	CG5442	SC35	6	0,9006
284	CG6556	cnk	6	0,9005
285	CG3704	CG3704	6	0,9005
286	CG3180	RpII140	6	0,9004
287	CG13090	CG13090	6	0,9004
288	CG9772	CG9772	6	0,9003
289	CG11132	DMAP1	6	0,9002
290	CG1258	pav	6	0,9001
291	CG6711	Taf2	6	0,8999
292	CG8114	pbl	6	0,8999
293	CG1558	l(1)G0237	6	0,8998
294	CG10354	CG10354	6	0,8997
295	CG9667	CG9667	6	0,8996
296	CG9548	CG9548	6	0,8996
297	CG4258	dbe	6	0,8996
298	CG1685	pen	6	0,8995
299	CG7483	eIF4AIII	6	0,8995
300	CG31671	tho2	6	0,8994
301	CG9723	CG9723	6	0,8990
302	CG13399	Chrac-14	6	0,8990
303	CG9771	Dip2	6	0,8989
304	CG6962	CG6962	6	0,8989
305	CG7183	CG7183	6	0,8989
306	CG5198	CG5198	6	0,8989
307	CG7833	Orc5	6	0,8989
308	CG5199	CG5199	6	0,8988
309	CG2945	cin	6	0,8988
310	CG11130	Rtc1	6	0,8987

311	CG2919	CG2919	6	0,8986
312	CG6349	CG6349	6	0,8986
313	CG12396	Nnp-1	6	0,8986
314	CG10161	CG10161	6	0,8986
315	CG6066	CG6066	6	0,8985
316	CG17947	alpha-Cat	6	0,8984
317	CG16788	RnpS1	6	0,8983
318	CG5874	CG5874	6	0,8982
319	CG11561	smo	6	0,8981
320	CG9862	Rae1	6	0,8981
321	CG8171	dup	6	0,8981
322	CG7128	Taf8	6	0,8980
323	CG5185	Tom	6	0,8980
324	CG5360	CG5360	6	0,8979
325	CG3998	zf30C	6	0,8979
326	CG5784	Mapmodulin	6	0,8978
327	CG3909	CG3909	6	0,8977
328	CG3542	CG3542	6	0,8975
329	CG4050	CG4050	6	0,8975
330	CG8715	lig	6	0,8974
331	CG11820	CG11820	6	0,8973
332	CG1891	sax	6	0,8973
333	CG8828	CG8828	6	0,8973
334	CG7098	dik	6	0,8972
335	CG16975	CG16975	6	0,8971
336	CG7006	CG7006	6	0,8971
337	CG17938	mdy	6	0,8971
338	CG13089	CG13089	6	0,8970
339	CG12941	CG30499	6	0,8968
340	CG12252	CG12252	6	0,8968
341	CG8223	CG8223	6	0,8967
342	CG7421	Nopp140	6	0,8967
343	CG5838	Dref	6	0,8966
344	CG8103	Mi-2	6	0,8966
345	CG3363	CG3363	6	0,8965
346	CG10753	snRNP69D	6	0,8965
347	CG5558	CG5558	6	0,8965
348	CG6610	CG6610	6	0,8964
349	CG5595	Sce	6	0,8964
350	CG6523	CG6523	6	0,8963
351	CG5857	CG5857	6	0,8962
352	CG4440	CG4440	6	0,8962
353	CG14749	CG14749	6	0,8961
354	CG3527	CG3527	6	0,8960
355	CG12260	mRpS22	6	0,8959
356	CG3991	TppII	6	0,8959
357	CG8092	CG8092	6	0,8957
358	CG7917	Nlp	6	0,8957
359	CG3938	CycE	6	0,8956
360	CG10060	G-ialpha65A	6	0,8956
361	CG7818	CG7818	6	0,8954
362	CG4400	CG4400	6	0,8954
363	CG10418	CG10418	6	0,8954

364	CG10805	CG10805	6	0,8954
365	CG13350	CG13350	6	0,8954
366	CG5499	His2Av	6	0,8954
367	CG8427	SmD3	6	0,8953
368	CG13867	MED8	6	0,8952
369	CG10726	barr	6	0,8952
370	CG5403	retn	6	0,8952
371	CG3071	CG3071	6	0,8951
372	CG3450	ubl	6	0,8950
373	CG5728	CG5728	6	0,8950
374	CG9984	TH1	6	0,8948
375	CG3096	Brd	6	0,8948
376	CG9373	CG9373	6	0,8946
377	CG16865	CG16865	6	0,8946
378	CG8890	Gmd	6	0,8945
379	CG8882	Trip1	6	0,8944
380	CG6843	CG6843	6	0,8944
381	CG15107	CG15107	6	0,8943
382	CG7185	CG7185	6	0,8943
383	CG8436	CG8436	6	0,8943
384	CG8902	CG8902	6	0,8943
385	CG16971	CG16971	6	0,8942
386	CG17383	CG17383	6	0,8940
387	CG6011	Prp18	6	0,8940
388	CG11985	CG11985	6	0,8938
389	CG2186	CG2186	6	0,8937
390	CG1523	CG1523	6	0,8937
391	CG10667	Orc1	6	0,8936
392	CG10565	CG10565	6	0,8935
393	CG1639	l(1)10Bb	6	0,8935
394	CG8929	CG8929	6	0,8935
395	CG3736	okr	6	0,8933
396	CG12366	O-fut1	6	0,8933
397	CG6538	TfIIFbeta	6	0,8933
398	CG3423	SA	6	0,8933
399	CG6712	CG6712	6	0,8933
400	CG7516	CG7516	6	0,8932
401	CG3510	CycB	6	0,8931
402	CG6015	CG6015	6	0,8931
403	CG11376	CG11376	6	0,8930
404	CG18591	CG18591	6	0,8930
405	CG7846	CG7846	6	0,8930
406	CG11906	CG11906	6	0,8929
407	CG7989	l(2)k07824	6	0,8928
408	CG13366	CG13364	6	0,8928
409	CG5000	msps	6	0,8927
410	CG8705	pnut	6	0,8927
411	CG10718	neb	6	0,8926
412	CG4266	CG4266	6	0,8926
413	CG12299	CG12299	6	0,8926
414	CG6249	Csl4	6	0,8925
415	CG4817	Ssrp	6	0,8925
416	CG8771	CG8771	6	0,8925

417	CG16838	CG16838	6	0,8924
418	CG5854	CG5854	6	0,8923
419	CG2260	CG2260	6	0,8923
420	CG6121	Tip60	6	0,8923
421	CG7942	CG7942	6	0,8921
422	CG2199	CG2199	6	0,8921
423	CG8933	exd	6	0,8920
424	CG5942	brm	6	0,8920
425	CG11881	CG11881	6	0,8919
426	CG33095	CG33095	6	0,8919
427	CG8274	Mtor	6	0,8918
428	CG6210	srt	6	0,8918
429	CG5965	woc	6	0,8917
430	CG7993	CG7993	6	0,8917
431	CG11563	CG11563	6	0,8917
432	CG18446	Lhr	6	0,8916
433	CG9277	betaTub56D	6	0,8916
434	CG3730	csul	6	0,8915
435	CG5033	CG5033	6	0,8914
436	CG12225	Spt6	6	0,8914
437	CG10414	CG10414	6	0,8914
438	CG12909	CG12909	6	0,8913
439	CG1740	Ntf-2	6	0,8912
440	CG6418	CG6418	6	0,8911
441	CG1216	mri	6	0,8910
442	CG5208	CG5208	6	0,8910
443	CG8892	CG8892	6	0,8910
444	CG2711	dwg	6	0,8909
445	CG8711	cul-4	6	0,8909
446	CG8108	CG8108	6	0,8909
447	CG14722	CG14722	6	0,8909
448	CG12196	egg	6	0,8909
449	CG5714	ecd	6	0,8909
450	CG14965	CG14965	6	0,8907
451	CG8153	mus210	6	0,8906
452	CG1866	Moca-cyp	6	0,8905
453	CG5003	CG5003	6	0,8904
454	CG9348	Taf6	6	0,8903
455	CG5694	CG5694	6	0,8903
456	CG8962	Paf-AHalpha	6	0,8903
457	CG5180	CG5180	6	0,8903
458	CG17498	mad2	6	0,8903
459	CG8730	drosha	6	0,8903
460	CG8374	dmt	6	0,8901
461	CG10975	Ptp69D	6	0,8901
462	CG9677	Int6	6	0,8901
463	CG5257	Dph5	6	0,8901
464	CG10473	CG10473	6	0,8900
465	CG13900	CG13900	6	0,8900
466	CG12391	CG12391	6	0,8899
467	CG5229	chm	6	0,8898
468	CG5787	CG5787	6	0,8898
469	CG7928	CG7928	6	0,8897

470	CG7836	mod(mdg4)	6	0,8897
471	CG4385	S	6	0,8897
472	CG10212	SMC2	6	0,8896
473	CG11228	hpo	6	0,8896
474	CG5370	Dcp-1	6	0,8895
475	CG5861	CG5861	6	0,8894
476	CG3041	Orc2	6	0,8893
477	CG10333	CG10333	6	0,8893
478	CG5371	RnrL	6	0,8892
479	CG9226	CG9226	6	0,8892
480	CG16728	CG16728	6	0,8891
481	CG17358	Taf12	6	0,8891
482	CG4602	Srp54	6	0,8889
483	CG11508	CG11508	6	0,8889
484	CG11375	polybromo	6	0,8888
485	CG5824	l(3)07882	6	0,8888
486	CG6899	Ptp4E	6	0,8888
487	CG11579	arm	6	0,8887
488	CG3193	crn	6	0,8886
489	CG16969	CG16969	6	0,8886
490	CG8989	His3.3B	6	0,8885
491	CG6563	Art3	6	0,8885
492	CG5510	CG5510	6	0,8883
493	CG1078	CG1078	6	0,8883
494	CG7472	CG31342	6	0,8883
495	CG2161	Rga	6	0,8882
496	CG4548	XNP	6	0,8881
497	CG3284	RpII15	6	0,8879
498	CG13298	CG13298	6	0,8879
499	CG7803	z	6	0,8878
500	CG3265	Eb1	6	0,8878
501	CG18081	CG18081	6	0,8878
502	CG7014	RpS5b	6	0,8878
503	CG12785	CG12785	6	0,8877
504	CG3522	Start1	6	0,8876
505	CG8149	CG8149	6	0,8876
506	CG1433	Atu	6	0,8875
507	CG13892	Cypl	6	0,8875
508	Nup98	Nup98	6	0,8874
509	CG7911	CG7911	6	0,8874
510	CG9484	hyd	6	0,8874
511	CG6143	Pep	6	0,8874
512	CG1518	CG1518	6	0,8874
513	CG10447	CG10447	6	0,8874
514	CG11987	tgo	6	0,8872
515	CG5109	Pcl	6	0,8872
516	CG8878	CG8878	6	0,8872
517	CG4281	CG4281	6	0,8871
518	CG5358	Art4	6	0,8871
519	CG4788	CG4788	6	0,8870
520	CG13329	cid	6	0,8870
521	CG18610	CG30122	6	0,8869
522	CG4528	snf	6	0,8869

523	CG6340	CG6340	6	0,8867
524	CG1430	bys	6	0,8866
525	CG8478	CG8478	6	0,8866
526	CG2173	Rs1	6	0,8866
527	CG6477	RhoGAP54D	6	0,8866
528	CG3358	CG3358	6	0,8866
529	CG9900	mit(1)15	6	0,8865
530	CG14802	MED18	6	0,8865
531	CG4494	smt3	6	0,8865
532	CG8928	CG8928	6	0,8863
533	CG18398	Tango6	6	0,8862
534	CG6413	Dis3	6	0,8861
535	CG1381	CG1381	6	0,8861
536	CG1943	CG1943	6	0,8860
537	CG16812	CG16812	6	0,8859
538	CG9045	Myb	6	0,8859
539	CG10754	CG10754	6	0,8859
540	CG5383	PSR	6	0,8859
541	CG14226	dome	6	0,8859
542	CG14646	CG14646	6	0,8859
543	CG9198	shtd	6	0,8858
544	CG3817	CG3817	6	0,8858
545	CG15218	CycK	6	0,8857
546	CG6755	EloA	6	0,8857
547	CG1911	CAP-D2	6	0,8855
548	CG5935	Dek	6	0,8855
549	CG15119	mip40	6	0,8854
550	CG16983	skpA	6	0,8854
551	CG13742	CG13742	6	0,8853
552	CG6617	CG6617	6	0,8852
553	CG10206	nop5	6	0,8851
554	CG8289	CG8289	6	0,8851
555	CG6197	CG6197	6	0,8851
556	CG12389	Fpps	6	0,8850
557	CG4709	CG4709	6	0,8849
558	CG1832	CG1832	6	0,8848
559	CG31917	CG31917	6	0,8848
560	CG5393	apt	6	0,8847
561	CG2213	CG2213	6	0,8847
562	CG8426	l(2)NC136	6	0,8846
563	CG1619	Hmr	6	0,8846
564	CG1783	Slip1	6	0,8845
565	CG2063	CG2063	6	0,8845
566	CG4152	l(2)35Df	6	0,8844
567	CG12357	Cbp20	6	0,8844
568	CG11866	CG11866	6	0,8844
569	CG4590	inx2	6	0,8843
570	CG17143	thoc7	6	0,8842
571	CG9745	D1	6	0,8841
572	CG7889	CG7889	6	0,8840
573	CG1234	CG1234	6	0,8840
574	CG11123	CG11123	6	0,8839
575	CG5869	CG5869	6	0,8839

576	CG9754	CG9754	6	0,8839
577	CG7626	Spt5	6	0,8839
578	CG7562	Trf	6	0,8838
579	CG3221	CG3221	6	0,8836
580	CG12340	CG12340	6	0,8836
581	CG9424	bocksbeutel	6	0,8835
582	CG8233	CG8233	6	0,8835
583	CG2614	CG2614	6	0,8835
584	CG3847	CG3847	6	0,8834
585	CG5649	kin17	6	0,8833
586	CG5757	CG5757	6	0,8833
587	CG8326	CG8326	6	0,8832
588	CG10318	NC2alpha	6	0,8832
589	CG5931	CG5931	6	0,8831
590	CG14124	CG31368	6	0,8831
591	CG15514	CG15514	6	0,8830
592	CG14941	esc	6	0,8829
593	CG7728	CG7728	6	0,8829
594	CG17559	dnt	6	0,8828
595	CG17161	grp	6	0,8828
596	CG3181	Ts	6	0,8828
597	CG11177	BthD	6	0,8828
598	CG10375	CG10375	6	0,8828
599	CG5642	CG5642	6	0,8827
600	CG14425	CG14425	6	0,8827
601	CG14710	CG14710	6	0,8826
602	CG5289	Pros26.4	6	0,8826
603	CG3924	Chi	6	0,8826
604	CG11988	neur	6	0,8825
605	CG31111	CG31111	6	0,8825
606	CG12076	YT521-B	6	0,8824
607	CG11266	CG11266	6	0,8824
608	CG12314	CG12314	6	0,8824
609	CG10225	CG10225	6	0,8824
610	CG4747	CG4747	6	0,8823
611	CG5193	TfIIB	6	0,8823
612	CG1459	CG32810	6	0,8822
613	CG6693	CG6693	6	0,8821
614	CG5640	CG5640	6	0,8821
615	CG15812	pfk	6	0,8821
616	CG6316	CG32069	6	0,8820
617	CG2021	CG2021	6	0,8820
618	CG13387	emb	6	0,8820
619	CG7265	CG7265	6	0,8819
620	CG1671	CG1671	6	0,8819
621	CG2182	CG2182	6	0,8818
622	CG8950	CG8950	6	0,8818
623	CG13689	CG13689	6	0,8817
624	CG10214	CG10214	6	0,8817
625	CG7957	MED17	6	0,8816
626	CG9375	Ras85D	6	0,8815
627	CG1017	CG1017	6	0,8814
628	CG3688	l(2)35Bd	6	0,8814

629	CG18600	CG18600	6	0,8813
630	CG11696	CG11696	6	0,8813
631	CG10069	CG10069	6	0,8813
632	CG6453	CG6453	6	0,8812
633	CG7154	CG7154	6	0,8812
634	CG8173	CG8173	6	0,8812
635	CG8631	msl-3	6	0,8812
636	CG14517	beta4GalNAcTB	6	0,8810
637	CG7275	CG7275	6	0,8810
638	CG16896	CG16896	6	0,8808
639	CG9211	iHog	6	0,8808
640	CG11875	CG11875	6	0,8808
641	CG3511	CG3511	6	0,8808
642	CG4886	cyp33	6	0,8807
643	CG5258	NHP2	6	0,8807
644	CG2845	phl	6	0,8807
645	CG6061	mip120	6	0,8806
646	CG7683	CG31232	6	0,8806
647	CG9888	Fib	6	0,8805
648	CG12134	CG12134	6	0,8805
649	CG9594	Chd3	6	0,8803
650	CG4184	MED15	6	0,8802
651	CG8886	l(2)05714	6	0,8801
652	CG8980	NiPp1	6	0,8801
653	CG15797	ric8a	6	0,8801
654	CG7012	nct	6	0,8800
655	CG11001	FK506-bp2	6	0,8798
656	CG10689	CG10689	6	0,8798
657	CG5179	Cdk9	6	0,8798
658	CG14985	CG14985	6	0,8798
659	CG12259	CG12259	6	0,8797
660	CG6219	cav	6	0,8797
661	CG9473	MED6	6	0,8797
662	CG8939	CG8939	6	0,8797
663	CG9207	Gas41	6	0,8795
664	CG10385	msl-1	6	0,8794
665	CG9107	CG9107	6	0,8794
666	CG10263	CG10263	6	0,8793
667	CG11964	CG11964	6	0,8793
668	CG6479	CG6479	6	0,8792
669	CG12848	CG12848	6	0,8792
670	CG2807	CG2807	6	0,8792
671	CG9351	flfl	6	0,8790
672	CG8243	CG8243	6	0,8787
673	CG11081	plexA	6	0,8787
674	CG32708	CG32708	6	0,8787
675	CG15481	Ski6	6	0,8787
676	CG17768	CG17768	6	0,8786
677	CG13849	Nop56	6	0,8786
678	CG11837	CG11837	6	0,8786
679	CG5739	CG5739	6	0,8784
680	CG8241	CG8241	6	0,8783
681	CG7643	ald	6	0,8782

682	CG15525	CG15525	6	0,8782
683	CG10756	Taf13	6	0,8782
684	CG15439	CG15439	6	0,8782
685	CG17293	CG17293	6	0,8782
686	CG1939	CG1939	6	0,8782
687	CG14614	CG14614	6	0,8781
688	CG5216	Sir2	6	0,8781
689	CG18041	CG18041	6	0,8780
690	CG11495	rasp	6	0,8780
691	CG16892	CG16892	6	0,8779
692	CG18632	CG32043	6	0,8779
693	CG17446	CG17446	6	0,8779
694	CG2368	psq	6	0,8779
695	CG1454	wdn	6	0,8779
696	CG11583	CG11583	6	0,8779
697	CG10850	ida	6	0,8777
698	CG32176	CG32176	6	0,8776
699	CG6554	Art1	6	0,8775
700	CG11246	Rpb8	6	0,8775
701	CG10336	CG10336	6	0,8773
702	CG10419	CG10419	6	0,8773
703	CG2109	mRpL44	6	0,8773
704	CG8924	CG8924	6	0,8773
705	CG9556	alien	6	0,8772
706	CG17002	CG17002	6	0,8772
707	CG3570	CG3570	6	0,8772
708	CG5786	ppan	6	0,8771
709	CG9802	Cap	6	0,8770
710	CG4700	Sema-2a	6	0,8770
711	CG2031	Hpr1	6	0,8770
712	CG4621	YL-1	6	0,8770
713	CG1569	rod	6	0,8769
714	CG17484	p120ctn	6	0,8765
715	CG10542	CG10542	6	0,8765
716	CG8383	Pnn	6	0,8765
717	CG3428	CG3428	6	0,8765
718	CG3887	CG3887	6	0,8763
719	CG9805	eIF3-S10	6	0,8760
720	CG7938	Sry-beta	6	0,8759
721	CG6768	DNApol-epsilon	6	0,8759
722	CG14884	CSN5	6	0,8759
723	CG3606	caz	6	0,8757
724	CG1836	Rad23	6	0,8757
725	CG10903	CG10903	6	0,8757
726	CG34099	Mkp	6	0,8756
727	CG2116	CG2116	6	0,8756
728	CG4922	sala	6	0,8756
729	CG5933	CG5933	6	0,8756
730	CG7705	CG7705	6	0,8755
731	CG9423	Kap-alpha3	6	0,8754
732	CG13914	CG13914	6	0,8753
733	CG6987	SF2	6	0,8753
734	CG5149	CG5149	6	0,8753

735	CG2617	CG2617	6	0,8753
736	CG7339	CG7339	6	0,8752
737	CG3941	pita	6	0,8751
738	CG31368	CG31368	6	0,8751
739	CG10981	CG10981	6	0,8750
740	CG9666	CG9666	6	0,8749
741	CG5994	Nelf-E	6	0,8749
742	CG5052	pim	6	0,8749
743	CG12135	c12.1	6	0,8748
744	CG5447	CG5447	6	0,8748
745	CG13599	CG13599	6	0,8747
746	CG5235	CG5235	6	0,8746
747	CG6506	CG6506	6	0,8746
748	CG32344	CG32344	6	0,8745
749	CG5121	MED28	6	0,8744
750	CG3403	CG3403	6	0,8743
751	CG1591	REG	6	0,8743
752	CG4832	cnn	6	0,8742
753	CG10722	CG10722	6	0,8742
754	CG12792	l(2)09851	6	0,8741
755	CG30349	CG30349	6	0,8741
756	CG2072	TXBP181-like	6	0,8741
757	CG17166	mRpL39	6	0,8740
758	CG9601	CG9601	6	0,8740
759	CG15835	CG15835	6	0,8740
760	CG6946	glo	6	0,8739
761	CG6157	dah	6	0,8737
762	CG15191	e(y)2	6	0,8735
763	CG6480	CG6480	6	0,8735
764	CG13192	CG13192	6	0,8735
765	CG12155	CG12155	6	0,8735
766	CG1871	e(r)	6	0,8735
767	CG5451	CG5451	6	0,8733
768	CG6937	CG6937	6	0,8733
769	CG4159	CG4159	6	0,8732
770	CG1218	CG1218	6	0,8730
771	CG12744	CG12744	6	0,8730
772	CG7757	CG7757	6	0,8729
773	CG13333	CG13333	6	0,8728
774	CG8039	mRpL19	6	0,8727
775	CG6176	Grip75	6	0,8727
776	CG2446	CG2446	6	0,8727
777	CG1624	dpld	6	0,8726
778	CG5893	D	6	0,8726
779	CG11006	CG11006	6	0,8725
780	CG4722	bib	6	0,8725
781	CG1877	lin19	6	0,8724
782	CG9775	CG9775	6	0,8723
783	CG8232	CG8232	6	0,8723
784	CG5163	TfIIA-S	6	0,8722
785	CG6724	CG6724	6	0,8722
786	CG3732	CG3732	6	0,8722
787	CG7108	DNApol-alpha50	6	0,8722

788	CG10042	MBD-R2	6	0,8722
789	CG3897	blot	6	0,8721
790	CG32491	mod(mdg4)	6	0,8721
791	CG1910	CG1910	6	0,8721
792	CG9018	CG9018	6	0,8720
793	CG10110	cpsf	6	0,8719
794	CG1119	Gnf1	6	0,8719
795	CG17033	CG17033	6	0,8719
796	CG8722	Nup44A	6	0,8718
797	CG7375	CG7375	6	0,8718
798	CG5336	Ced-12	6	0,8718
799	CG1420	CG1420	6	0,8716
800	CG11678	Actr13E	6	0,8716
801	CG1903	sno	6	0,8714
802	CG14478	CG14478	6	0,8714
803	CG8395	Rrp42	6	0,8714
804	CG11482	Mlh1	6	0,8713
805	CG8590	Klp3A	6	0,8713
806	CG18178	CG18178	6	0,8713
807	CG3351	mRpL11	6	0,8713
808	CG2685	CG2685	6	0,8713
809	CG8636	CG8636	6	0,8712
810	CG15019	CG15019	6	0,8712
811	CG12249	mira	6	0,8712
812	CG2671	l(2)gl	6	0,8710
813	CG18012	CG18012	6	0,8710
814	CG10719	brat	6	0,8709
815	CG4254	tsr	6	0,8709
816	CG2859	Taf10	6	0,8709
817	CG3458	Top3beta	6	0,8709
818	CG9615	tex	6	0,8708
819	CG8805	wun2	6	0,8708
820	CG5543	CG5543	6	0,8708
821	CG8597	lark	6	0,8708
822	CG1605	az2	6	0,8707
823	CG4916	me31B	6	0,8707
824	CG1676	cactin	6	0,8707
825	CG8545	CG8545	6	0,8706
826	CG5655	Rsf1	6	0,8704
827	CG6801	l(3)j2D3	6	0,8704
828	CG8573	su(Hw)	6	0,8703
829	CG4326	mRpS17	6	0,8703
830	CG15735	CG15735	6	0,8701
831	CG15845	Adf1	6	0,8701
832	CG10279	Rm62	6	0,8701
833	CG33097	CG33097	6	0,8700
834	CG9246	CG9246	6	0,8699
835	CG9281	CG9281	6	0,8699
836	CG1800	CG18004	6	0,8699
837	CG12223	Dsp1	6	0,8698
838	CG14514	Brd8	6	0,8698
839	CG13190	CG13190	6	0,8697
840	CG7933	janA	6	0,8697

841	CG12019	Cdc37	6	0,8697
842	CG4934	brn	6	0,8697
843	CG6686	CG6686	6	0,8696
844	CG32346	E(bx)	6	0,8695
845	CG10117	ttv	6	0,8694
846	CG13025	CG13025	6	0,8694
847	CG11798	chn	6	0,8693
848	CG3169	Spt3	6	0,8693
849	CG7999	MED24	6	0,8692
850	CG5408	trbl	6	0,8692
851	CG8025	Mtr3	6	0,8691
852	CG1276	TfIIEbeta	6	0,8691
853	CG6057	SMC1	6	0,8690
854	CG3056	CG3056	6	0,8689
855	CG3931	Rrp4	6	0,8689
856	CG17064	mars	6	0,8688
857	CG11755	CG11755	6	0,8688
858	CG10465	CG10465	6	0,8688
859	CG10954	Arc-p34	6	0,8688
860	CG13625	CG13625	6	0,8687
861	CG7099	CG7099	6	0,8686
862	CG2177	CG2177	6	0,8685
863	CG12301	CG12301	6	0,8685
864	CG4975	CG4975	6	0,8685
865	CG11115	Ssl1	6	0,8684
866	CG11419	CG11419	6	0,8683
867	CG10139	CG10139	6	0,8682
868	CG6193	Apc2	6	0,8682
869	CG1406	U2A	6	0,8682
870	CG10267	CG10267	6	0,8681
871	CG3983	CG3983	6	0,8679
872	CG7380	CG7380	6	0,8679
873	CG4539	Bka	6	0,8679
874	CG30349	CG30349	6	0,8678
875	CG4043	Rrp46	6	0,8678
876	CG5977	spas	6	0,8677
877	CG4282	CG4282	6	0,8677
878	CG7003	CG7003	6	0,8677
879	CG17266	CG17266	6	0,8675
880	CG4119	CG4119	6	0,8675
881	CG1554	RpII215	6	0,8674
882	CG6487	CG32066	6	0,8674
883	CG5102	da	6	0,8674
884	CG7372	CG7372	6	0,8673
885	CG5923	DNApol-alpha73	6	0,8673
886	CG2915	CG2915	6	0,8672
887	CG8960	CG8960	6	0,8672
888	CG7719	gwl	6	0,8670
889	CG11971	CG11971	6	0,8670
890	CG12031	MED14	6	0,8669
891	CG4898	Tm1	6	0,8669
892	CG6884	MED11	6	0,8669
893	CG4449	CG4449	6	0,8668

894	CG10035	CG10035	6	0,8668
895	CG3572	vimar	6	0,8668
896	CG8211	CG8211	6	0,8667
897	CG8396	Ssb-c31a	6	0,8666
898	CG6944	Lam	6	0,8666
899	CG15160	CG15160	6	0,8666
900	CG4785	CG4785	6	0,8666
901	CG14037	Pp4-19C	6	0,8665
902	CG6398	CG6398	6	0,8665
903	CG18780	MED20	6	0,8664
904	CG4768	CG4768	6	0,8663
905	CG7172	CG7172	6	0,8662
906	CG5252	Ranbp9	6	0,8662
907	CG5495	Txl	6	0,8662
908	CG10415	TfIIEalpha	6	0,8662
909	CG4951	CG4951	6	0,8657
910	CG5934	CG5934	6	0,8657
911	CG11859	CG11859	6	0,8655
912	CG10540	cpa	6	0,8653
913	CG18005	Trf2	6	0,8653
914	CG4247	mRpS10	6	0,8652
915	CG3957	CG3957	6	0,8652
916	CG5184	mRpS11	6	0,8651
917	CG8268	Srp9	6	0,8651
918	CG7162	MED1	6	0,8651
919	CG8606	RhoGEF4	6	0,8651
920	CG13381	CG13379	6	0,8650
921	CG4148	wek	6	0,8650
922	CG6637	CG6637	6	0,8650
923	CG12085	pUf68	6	0,8649
924	CG4453	Nup153	6	0,8649
925	CG2691	CG2691	6	0,8646
926	CG1775	Med	6	0,8645
927	CG17528	CG17528	6	0,8644
928	CG8961	tef	6	0,8644
929	CG17054	Cap-G	6	0,8644
930	CG3735	CG3735	6	0,8643
931	CG13425	bl	6	0,8642
932	CG4173	Sep2	6	0,8641
933	CG3000	rap	6	0,8640
934	CG7150	Rpb4	6	0,8640
935	CG4903	MESR4	6	0,8639
936	CG5303	mei-S332	6	0,8639
937	CG4268	Pitslre	6	0,8639
938	CG8461	CG8461	6	0,8639
939	CG9638	Ada2b	6	0,8636
940	CG9233	fu2	6	0,8634
941	CG5837	Hem	6	0,8634
942	CG8495	RpS29	6	0,8633
943	CG7837	CG7837	6	0,8633
944	CG1785	CG1785	6	0,8633
945	CG12287	pdm2	6	0,8631
946	CG10059	MAGE	6	0,8631

947	CG5972	Arc-p20	6	0,8631
948	CG10583	Sse	6	0,8630
949	CG1710	Hcf	6	0,8630
950	CG8414	CG8414	6	0,8629
951	CG14805	CG14805	6	0,8629
952	CG10122	RpI1	6	0,8627
953	CG18259	CG18259	6	0,8627
954	CG18582	mbt	6	0,8627
955	CG11777	CG11777	6	0,8627
956	CG6422	CG6422	6	0,8626
957	CG2917	Orc4	6	0,8626
958	CG4185	NC2beta	6	0,8625
959	CG9054	Ddx1	6	0,8625
960	CG15237	CG15237	6	0,8625
961	CG5108	mRpS7	6	0,8624
962	CG8454	Vps16A	6	0,8624
963	CG12942	CG12942	6	0,8624
964	CG18151	CG13364	6	0,8623
965	CG6370	CG6370	6	0,8623
966	CG12189	Rev1	6	0,8622
967	CG17068	CG17068	6	0,8622
968	CG13454	CG13454	6	0,8622
969	CG2534	cno	6	0,8621
970	CG3171	Tre1	6	0,8621
971	CG17295	CG17295	6	0,8621
972	CG3434	CG3434	6	0,8620
973	CG4200	sl	6	0,8620
974	CG1893	CG1893	6	0,8620
975	CG3956	sna	6	0,8619
976	CG2128	Hdac3	6	0,8618
977	CG9752	CG9752	6	0,8618
978	CG6854	CG6854	6	0,8617
979	CG7698	CG7698	6	0,8617
980	CG2916	Sep5	6	0,8616
981	CG10904	CG10904	6	0,8616
982	CG4202	Sas10	6	0,8615
983	CG6977	Cad87A	6	0,8615
984	CG14230	CG14230	6	0,8613
985	CG7637	CG7637	6	0,8612
986	CG7656	CG7656	6	0,8612
987	CG4195	l(3)73Ah	6	0,8612
988	CG5871	CG5871	6	0,8610
989	CG11994	Ada	6	0,8610
990	CG10563	CG10563	6	0,8610
991	CG15266	CG15266	6	0,8608
992	CG4865	CG4865	6	0,8607
993	CG7139	CG7139	6	0,8607
994	CG4866	CG4866	6	0,8607
995	CG7081	CG7081	6	0,8606
996	CG2051	CG2051	6	0,8606
997	CG11183	Dcp1	6	0,8606
998	CG8169	Pms2	6	0,8605
999	CG12317	JhI-21	6	0,8605

1000	CG1511	Eph	6	0,8605
1001	CG10496	CG10496	6	0,8604
1002	CG18004	CG18005	6	0,8604
1003	CG8815	Sin3A	6	0,8603
1004	CG5063	Trax	6	0,8603
1005	CG2855	aph-1	6	0,8603
1006	CG4038	CG4038	6	0,8603
1007	CG1646	CG1646	6	0,8603
1008	CG33096	CG33096	6	0,8602
1009	CG9606	Rrp45	6	0,8602
1010	CG3260	Zfrp8	6	0,8601
1011	CG16753	CG16753	6	0,8600
1012	CG14222	CG14222	6	0,8600
1013	CG9588	CG9588	6	0,8599
1014	CG8069	Phax	6	0,8599
1015	CG17149	CG17149	6	0,8599
1016	CG5859	CG5859	6	0,8598
1017	CG12109	Caf1-180	6	0,8596
1018	CG11305	CG11306	6	0,8595
1019	CG17486	CG17486	6	0,8595
1020	CG7175	CG7175	6	0,8594
1021	CG6744	CG6744	6	0,8593
1022	CG9053	CG9053	6	0,8593
1023	CG2050	mod	6	0,8593
1024	CG1587	Crk	6	0,8593
1025	CG3083	Prx6005	6	0,8593
1026	CG7074	mio	6	0,8592
1027	CG7655	CG7655	6	0,8591
1028	CG2875	CG2875	6	0,8591
1029	CG18350	Sxl	6	0,8591
1030	CG9253	CG9253	6	0,8591
1031	CG1584	Orc6	6	0,8591
1032	CG10217	CG10217	6	0,8590
1033	CG15387	CG15387	6	0,8589
1034	CG17385	CG17385	6	0,8588
1035	CG2972	CG2972	6	0,8587
1036	CG8616	CG8616	6	0,8587
1037	CG9000	CG9000	6	0,8586
1038	CG4207	bonsai	6	0,8586
1039	CG10327	TBPH	6	0,8586
1040	CG5462	scrib	6	0,8584
1041	CG6241	CG6241	6	0,8584
1042	CG12020	CG12020	6	0,8584
1043	CG11665	CG11665	6	0,8583
1044	CG6501	Ngp	6	0,8581
1045	CG10377	Hrb27C	6	0,8581
1046	CG9643	CG9643	6	0,8581
1047	CG5317	CG5317	6	0,8580
1048	CG3231	CG3231	6	0,8576
1049	CG7609	CG7609	6	0,8576
1050	CG6254	CG6254	6	0,8576
1051	CG15443	CG15443	6	0,8575
1052	CG4570	CG4570	6	0,8575

1053	CG31918	CG31918	6	0,8575
1054	CG13929	metl	6	0,8575
1055	CG15437	morgue	6	0,8574
1056	CG3680	CG3680	6	0,8574
1057	CG18190	CG18190	6	0,8574
1058	CG9022	Ost48	6	0,8573
1059	CG1354	CG1354	6	0,8573
1060	CG2926	CG2926	6	0,8573
1061	CG5454	CG5454	6	0,8572
1062	CG13072	PDCD-5	6	0,8572
1063	CG8207	CG8207	6	0,8571
1064	CG10395	CG10395	6	0,8570
1065	CG4954	eIF3-S8	6	0,8569
1066	CG9933	A16	6	0,8569
1067	CG3048	Traf1	6	0,8568
1068	CG5516	CG5516	6	0,8568
1069	CG8005	CG8005	6	0,8568
1070	CG6905	CG6905	6	0,8567
1071	CG9293	CG9293	6	0,8567
1072	CG8019	hay	6	0,8565
1073	CG9680	Dbp73D	6	0,8565
1074	CG2503	atms	6	0,8564
1075	CG8367	cg	6	0,8563
1076	CG4933	CG4933	6	0,8562
1077	CG2931	CG2931	6	0,8562
1078	CG1244	CG1244	6	0,8561
1079	CG7873	Src42A	6	0,8561
1080	CG6791	CG6791	6	0,8561
1081	CG4852	Sras	6	0,8561
1082	CG31349	pyd	6	0,8560
1083	CG15601	CG15601	6	0,8560
1084	CG13628	Rpb10	6	0,8559
1085	CG7611	CG7611	6	0,8559
1086	CG7358	CG7358	6	0,8559
1087	Nup98	Nup98	6	0,8558
1088	CG15440	CG15440	6	0,8557
1089	CG16974	CG16974	6	0,8556
1090	CG3163	CG3163	6	0,8556
1091	CG8195	CG8195	6	0,8556
1092	CG10229	katanin-60	6	0,8556
1093	CG8151	Tfb1	6	0,8554
1094	CG17952	LBR	6	0,8553
1095	CG7129	l(3)05822	6	0,8553
1096	CG4063	ebi	6	0,8552
1097	CG3619	Dl	6	0,8552
1098	CG6227	CG6227	6	0,8551
1099	CG12182	CG12182	6	0,8550
1100	CG11063	CG11063	6	0,8550
1101	CG6742	cenB1A	6	0,8549
1102	CG3226	CG3226	6	0,8548
1103	CG9576	CG9576	6	0,8548
1104	CG5497	mRpS28	6	0,8547
1105	CG2670	Taf7	6	0,8547

1106	CG9300	CG9300	6	0,8546
1107	CG11980	CG11980	6	0,8546
1108	CG8049	Btk29A	6	0,8543
1109	CG4931	Sra-1	6	0,8541
1110	CG4561	Aats-tyr	6	0,8540
1111	CG4448	CG4448	6	0,8540
1112	CG17291	Pp2A-29B	6	0,8540
1113	CG13098	mRpL51	6	0,8540
1114	CG6364	CG6364	6	0,8539
1115	CG3571	CG3571	6	0,8539
1116	CG9209	vap	6	0,8539
1117	CG30394	CG30394	6	0,8538
1118	CG5825	His3.3A	6	0,8538
1119	CG4912	eEF1delta	6	0,8537
1120	CG9776	CG9776	6	0,8536
1121	CG8674	l(2)k14505	6	0,8536
1122	CG6234	CG6234	6	0,8535
1123	CG3658	CDC45L	6	0,8535
1124	CG1135	Lim1	6	0,8535
1125	CG9949	sina	6	0,8534
1126	CG13277	CG13277	6	0,8534
1127	CG12393	CG12393	6	0,8534
1128	CG13213	CG13213	6	0,8533
1129	CG4003	pont	6	0,8532
1130	CG9839	CG9839	6	0,8530
1131	CG14966	CG14966	6	0,8530
1132	CG2867	Prat	6	0,8530
1133	CG18642	Bem46	6	0,8529
1134	CG6758	CG6758	6	0,8527
1135	CG8070	Mys45A	6	0,8526
1136	CG9020	Aats-arg	6	0,8525
1137	CG11604	CG11604	6	0,8525
1138	CG10572	Cdk8	6	0,8524
1139	CG7028	CG7028	6	0,8524
1140	CG2944	gus	6	0,8523
1141	CG5384	CG5384	6	0,8522
1142	CG12819	sle	6	0,8522
1143	CG11190	CG11190	6	0,8522
1144	CG9060	Zpr1	6	0,8521
1145	CG1236	CG1236	6	0,8519
1146	CG10080	CG10080	6	0,8519
1147	CG9125	CG9125	6	0,8518
1148	CG31441	CG31441	6	0,8517
1149	CG3889	CSN1b	6	0,8516
1150	CG3480	mip130	6	0,8516
1151	CG2198	Ama	6	0,8514
1152	CG8445	CG8445	6	0,8514
1153	CG14215	CG14215	6	0,8514
1154	CG6961	CG6961	6	0,8513
1155	CG6850	Ugt	6	0,8511
1156	CG7413	Rbf	6	0,8511
1157	CG13281	Cas	6	0,8511
1158	CG1405	l(3)neo38	6	0,8509

1159	CG6550	CG6550	6	0,8507
1160	CG12369	Lac	6	0,8505
1161	CG9750	rept	6	0,8505
1162	CG3838	CG3838	6	0,8505
1163	CG1989	Yippee	6	0,8504
1164	CG3329	Prosbeta2	6	0,8504
1165	CG15433	CG15433	6	0,8502
1166	CG11839	CG11839	6	0,8502
1167	CG6734	CG6734	6	0,8501
1168	CG7109	mts	6	0,8500
1169	CG4444	px	6	0,8498
1170	CG7020	DIP2	6	0,8498
1171	CG8400	CG8400	6	0,8498
1172	CG11100	Mes2	6	0,8498
1173	CG17249	CG17249	6	0,8496
1174	CG8208	MBD-like	6	0,8496
1175	CG3060	mr	6	0,8496
1176	CG5969	CG5969	6	0,8496
1177	CG8338	mRpS16	6	0,8496
1178	CG6814	Mat89Bb	6	0,8495
1179	CG4166	not	6	0,8495
1180	CG1341	Rpt1	6	0,8494
1181	CG3140	Adk2	6	0,8493
1182	CG4452	CG4452	6	0,8492
1183	CG1058	rpk	6	0,8492
1184	CG5986	CG5986	6	0,8492
1185	CG5114	CG5114	6	0,8487
1186	CG1603	CG1603	6	0,8487
1187	CG4798	l(2)k01209	6	0,8486
1188	CG4560	Arpc3A	6	0,8486
1189	CG17791	sqd	6	0,8485
1190	CG11808	CG11808	6	0,8484
1191	CG7638	CG7638	6	0,8484
1192	CG1463	CG1463	6	0,8483
1193	CG5815	CG5815	6	0,8481
1194	CG8378	CG8378	6	0,8480
1195	CG11504	CG11504	6	0,8480
1196	CG9799	CG9799	6	0,8480
1197	CG12397	CG31826	6	0,8480
1198	CG7200	CG7200	6	0,8480
1199	CG5253	CG31156	6	0,8478
1200	CG5190	CG5190	6	0,8478
1201	CG9596	CG9596	6	0,8478
1202	CG18145	Ripalpha	6	0,8478
1203	CG4887	CG4887	6	0,8477
1204	CG3707	wapl	6	0,8477
1205	CG4107	Pcaf	6	0,8477
1206	CG3733	Chd1	6	0,8476
1207	CG9945	CG9945	6	0,8476
1208	CG1440	CG1440	6	0,8475
1209	CG7338	CG7338	6	0,8475
1210	CG3815	CG3815	6	0,8473
1211	CG7487	RecQ4	6	0,8471

1212	CG18124	mTTF	6	0,8471
1213	CG8202	CG8202	6	0,8470
1214	CG8581	fra	6	0,8470
1215	CG4337	mtSSB	6	0,8469
1216	CG3109	mRpL16	6	0,8468
1217	CG8435	CG8435	6	0,8467
1218	CG6476	Su(var)3-9	6	0,8466
1219	CG9181	Ptp61F	6	0,8466
1220	CG7186	SAK	6	0,8465
1221	CG7749	fat2	6	0,8463
1222	CG9696	dom	6	0,8463
1223	CG1782	Uba1	6	0,8461
1224	CG11290	enok	6	0,8461
1225	CG11606	Rpp30	6	0,8460
1226	CG7110	CG7110	6	0,8460
1227	CG10880	CG10880	6	0,8459
1228	CG16840	Art8	6	0,8458
1229	CG11596	CG11596	6	0,8458
1230	CG4742	mRpL22	6	0,8458
1231	CG6838	CG6838	6	0,8458
1232	CG9123	CG9123	6	0,8457
1233	CG7182	CG7182	6	0,8457
1234	CG5140	CG5140	6	0,8457
1235	CG3776	CG3776	6	0,8457
1236	CG8441	CG8441	6	0,8457
1237	CG15480	CG15480	6	0,8456
1238	CG3715	Shc	6	0,8455
1239	CG10379	mbc	6	0,8455
1240	CG4500	CG4500	6	0,8455
1241	CG10627	CG10627	6	0,8454
1242	CG11377	CG11377	6	0,8453
1243	CG14788	l(1)G0431	6	0,8453
1244	CG7948	spn-A	6	0,8452
1245	CG18419	ytr	6	0,8452
1246	CG11451	CG11451	6	0,8451
1247	CG4033	RpI135	6	0,8450
1248	CG9028	CG9028	6	0,8450
1249	CG1371	CG1371	6	0,8449
1250	CG4806	CG4806	6	0,8449
1251	CG6070	CG6070	6	0,8449
1252	CG1810	mRNA-capping-enz	6	0,8448
1253	CG14213	CG14213	6	0,8448
1254	CG9437	CG9437	6	0,8448
1255	CG12749	Hrb87F	6	0,8447
1256	CG32763	l(1)G0045	6	0,8446
1257	CG6236	CG6236	6	0,8446
1258	CG11593	CG11593	6	0,8445
1259	CG6958	CG6958	6	0,8443
1260	CG18766	CG18766	6	0,8442
1261	CG34104	CG34104	6	0,8440
1262	CG7524	Src64B	6	0,8440
1263	CG6116	CG6116	6	0,8440
1264	CG10984	CG10984	6	0,8440

1265	CG13123	CG13123	6	0,8439
1266	CG15087	CG15087	6	0,8438
1267	CG11212	Ptr	6	0,8437
1268	CG11127	CG11127	6	0,8437
1269	CG16724	tra	6	0,8437
1270	CG3637	Cortactin	6	0,8436
1271	CG11788	CG11788	6	0,8436
1272	CG1044	dos	6	0,8434
1273	CG6284	Sirt6	6	0,8434
1274	CG11274	SRm160	6	0,8433
1275	CG6725	Sulf1	6	0,8433
1276	CG32604	l(1)G0007	6	0,8431
1277	CG11301	Sirt7	6	0,8430
1278	CG8856	Sr-CII	6	0,8430
1279	CG17509	CG17509	6	0,8428
1280	CG17743	pho	6	0,8428
1281	CG4016	Spt-I	6	0,8428
1282	CG10392	Ogt	6	0,8427
1283	CG16725	Smn	6	0,8426
1284	CG8569	CG8569	6	0,8426
1285	CG6379	CG6379	6	0,8425
1286	CG3165	CG3165	6	0,8424
1287	CG15877	CG15877	6	0,8422
1288	CG8611	CG8611	6	0,8422
1289	CG6189	l(1)1Bi	6	0,8421
1290	CG7926	Axn	6	0,8421
1291	CG1435	CBP	6	0,8420
1292	CG9853	CG9853	6	0,8420
1293	CG5439	CG5439	6	0,8417
1294	CG6146	Top1	6	0,8417
1295	CG13097	CG13097	6	0,8416
1296	CG7484	CG7484	6	0,8416
1297	CG5068	CG5068	6	0,8416
1298	CG5537	CG5537	6	0,8415
1299	CG4086	Su(P)	6	0,8414
1300	CG10230	Rpn9	6	0,8413
1301	CG8816	CG8816	6	0,8412
1302	CG4364	CG4364	6	0,8412
1303	CG11586	CG11586	6	0,8412
1304	CG4069	CG4069	6	0,8411
1305	CG12756	Eaf6	6	0,8410
1306	CG7011	CG7011	6	0,8410
1307	CG10685	CG10685	6	0,8409
1308	CG6995	CG6995	6	0,8409
1309	CG9764	yrt	6	0,8408
1310	CG6877	Aut1	6	0,8408
1311	CG6833	CG6833	6	0,8407
1312	CG13608	mRpS24	6	0,8407
1313	CG7564	CG7564	6	0,8406
1314	CG6315	fl(2)d	6	0,8404
1315	CG15110	botv	6	0,8403
1316	CG9769	CG9769	6	0,8403
1317	CG9213	CG9213	6	0,8402

1318	CG5274	CG5274	6	0,8401
1319	CG7467	osa	6	0,8398
1320	CG7810	CG7810	6	0,8398
1321	CG5748	Hsf	6	0,8397
1322	CG5445	CG5445	6	0,8396
1323	CG6133	CG6133	6	0,8396
1324	CG4949	CG4949	6	0,8396
1325	CG11329	opa	6	0,8395
1326	CG32390	CG32390	6	0,8394
1327	CG3278	Tif-IA	6	0,8393
1328	CG3696	kis	6	0,8393
1329	CG6182	CG6182	6	0,8391
1330	CG1434	CG1434	6	0,8390
1331	CG3228	kz	6	0,8390
1332	CG6654	CG6654	6	0,8390
1333	CG14816	CG14816	6	0,8390
1334	CG3962	Keap1	6	0,8389
1335	CG17238	Jupiter	6	0,8389
1336	CG7636	mRpL2	6	0,8389
1337	CG5993	os	6	0,8388
1338	CG30388	Magi	6	0,8388
1339	CG9200	CG9200	6	0,8387
1340	CG31650	CG31650	6	0,8386
1341	CG2048	dco	6	0,8384
1342	CG16928	mre11	6	0,8384
1343	CG13993	CG13993	6	0,8384
1344	CG2922	exba	6	0,8383
1345	CG4144	GNBP2	6	0,8383
1346	CG16940	CG16940	6	0,8381
1347	CG8598	eco	6	0,8380
1348	CG17256	Nek2	6	0,8379
1349	CG10536	cbx	6	0,8379
1350	CG12750	ncm	6	0,8378
1351	CG1658	Doa	6	0,8377
1352	CG5684	Pop2	6	0,8377
1353	CG1716	CG1716	6	0,8377
1354	CG10897	tou	6	0,8375
1355	CG11045	Ent2	6	0,8374
1356	CG9215	CG9215	6	0,8374
1357	CG5277	Ip259	6	0,8373
1358	CG4879	RecQ5	6	0,8372
1359	CG11357	CG11357	6	0,8370
1360	CG8787	Asx	6	0,8370
1361	CG1697	rho-4	6	0,8368
1362	CG4606	alpha-Man-IIb	6	0,8368
1363	CG9946	eIF-2alpha	6	0,8368
1364	CG7747	CG7747	6	0,8367
1365	CG7563	CalpA	6	0,8367
1366	CG7597	CG7597	6	0,8364
1367	CG4078	CG4078	6	0,8364
1368	CG7038	mRpL30	6	0,8363
1369	CG1738	CG1738	6	0,8362
1370	CG9983	Hrb98DE	6	0,8362

1371	CG12128	CG12128	6	0,8362
1372	CG5505	mule	6	0,8362
1373	CG5591	CG5591	6	0,8360
1374	CG7102	CG7102	6	0,8360
1375	CG3335	CG3335	6	0,8359
1376	CG5215	Zn72D	6	0,8357
1377	CG3712	mRpL33	6	0,8357
1378	CG6721	Gap1	6	0,8356
1379	CG14220	CG14220	6	0,8356
1380	CG1311	CG1311	6	0,8355
1381	CG7008	Tudor-SN	6	0,8354
1382	CG10334	spi	6	0,8352
1383	CG10653	hk	6	0,8352
1384	CG31212	Ino80	6	0,8351
1385	CG15793	Dsor1	6	0,8351
1386	CG16784	pr	6	0,8350
1387	CG3929	dx	6	0,8349
1388	CG7713	CG7713	6	0,8348
1389	CG5262	CG5262	6	0,8346
1390	CG8328	HLHmdelta	6	0,8345
1391	CG18213	CG18213	6	0,8345
1392	CG8003	CG8003	6	0,8343
1393	CG2222	Psf3	6	0,8342
1394	CG13142	CG13142	6	0,8342
1395	CG6302	l(3)01239	6	0,8342
1396	CG6230	CG6230	6	0,8342
1397	CG3287	CG3287	6	0,8342
1398	CG10679	Nedd8	6	0,8341
1399	CG12530	Cdc42	6	0,8341
1400	CG14210	CG14210	6	0,8340
1401	CG3291	pcm	6	0,8340
1402	CG8031	CG8031	6	0,8340
1403	CG5888	CG5888	6	0,8340
1404	CG1489	Pros45	6	0,8338
1405	CG11738	l(1)G0004	6	0,8338
1406	CG7764	Tfb2	6	0,8337
1407	CG3910	mtTFB2	6	0,8336
1408	CG11076	CG11076	6	0,8334
1409	CG8526	CG8526	6	0,8333
1410	CG8860	CG8860	6	0,8333
1411	CG12298	sub	6	0,8332
1412	CG3727	dock	6	0,8331
1413	CG31256	Brf	6	0,8331
1414	CG5841	mib1	6	0,8330
1415	CG5954	l(3)mbt	6	0,8329
1416	CG3407	CG3407	6	0,8327
1417	CG3689	CG3689	6	0,8325
1418	CG2677	eIF2B-beta	6	0,8325
1419	CG11164	CG11164	6	0,8325
1420	CG9804	CG9804	6	0,8325
1421	CG15626	CG15626	6	0,8323
1422	CG10949	CG10949	6	0,8322
1423	CG7870	CG7870	6	0,8321

1424	CG5692	raps	6	0,8321
1425	CG11849	dan	6	0,8320
1426	CG9749	Abi	6	0,8320
1427	CG6297	JIL-1	6	0,8320
1428	CG8612	mRpL50	6	0,8319
1429	CG4554	CG4554	6	0,8319
1430	CG10406	mRpS33	6	0,8318
1431	CG11555	CG11555	6	0,8318
1432	CG5227	sdk	6	0,8318
1433	CG7037	Cbl	6	0,8317
1434	CG2512	alphaTub84D	6	0,8317
1435	CG3186	eIF-5A	6	0,8317
1436	CG13567	CG13567	6	0,8316
1437	CG10907	CG10907	6	0,8316
1438	CG3093	dor	6	0,8316
1439	CG7222	CG7222	6	0,8316
1440	CG5138	Ocho	6	0,8315
1441	CG11956	SP1029	6	0,8315
1442	CG14647	CG14647	6	0,8315
1443	CG12487	BobA	6	0,8314
1444	CG4643	CG4643	6	0,8313
1445	CG3223	CG3223	6	0,8313
1446	CG4878	eIF3-S9	6	0,8312
1447	CG9422	CG9422	6	0,8309
1448	CG4943	lack	6	0,8309
1449	CG10188	CG10188	6	0,8308
1450	CG14135	CG14135	6	0,8308
1451	CG3679	CG3679	6	0,8308
1452	CG33052	CG33052	6	0,8308
1453	CG1660	Tim9a	6	0,8308
1454	CG14544	CG14544	6	0,8307
1455	CG3173	CG3173	6	0,8307
1456	CG31120	CG31120	6	0,8306
1457	CG1977	alpha-Spec	6	0,8305
1458	CG7696	CG31224	6	0,8303
1459	CG11877	CG11877	6	0,8302
1460	CG10873	p53	6	0,8302
1461	CG12702	CG12702	6	0,8302
1462	CG5196	CG5196	6	0,8301
1463	CG3189	Dpit47	6	0,8301
1464	CG17652	CG17652	6	0,8301
1465	CG17216	KP78b	6	0,8301
1466	CG7903	CG7903	6	0,8299
1467	CG14549	Sld5	6	0,8299
1468	CG10321	CG10321	6	0,8298
1469	CG1795	Ogg1	6	0,8297
1470	CG8370	CG8370	6	0,8296
1471	CG6056	AP-2sigma	6	0,8296
1472	CG8486	CG8486	6	0,8295
1473	CG8589	CG8589	6	0,8295
1474	CG10778	CG10778	6	0,8294
1475	CG2525	Hus1-like	6	0,8294
1476	CG7706	CG7706	6	0,8294

1477	CG6751	CG6751	6	0,8293
1478	CG5585	CG5585	6	0,8293
1479	CG3771	a6	6	0,8292
1480	CG17735	CG17735	6	0,8291
1481	CG12220	mRpL32	6	0,8291
1482	CG2469	CG2469	6	0,8291
1483	CG5821	qkr58E-2	6	0,8291
1484	CG11811	CG11811	6	0,8291
1485	CG18555	CG18555	6	0,8291
1486	CG3313	CG3313	6	0,8290
1487	CG8270	CG8270	6	0,8289
1488	CG5809	CaBP1	6	0,8288
1489	CG1772	dap	6	0,8288
1490	CG3710	TfIIS	6	0,8286
1491	CG11109	CG11109	6	0,8284
1492	CG7583	CtBP	6	0,8284
1493	CG13570	spag	6	0,8284
1494	CG32146	dlp	6	0,8283
1495	CG10144	CG10144	6	0,8283
1496	CG7197	CG7197	6	0,8283
1497	CG12026	CG12026	6	0,8282
1498	CG2144	CG2144	6	0,8282
1499	CG9015	en	6	0,8280
1500	CG1888	CG1888	6	0,8279
1501	CG8729	rnh1	6	0,8277
1502	CG5490	Tl	6	0,8277
1503	CG9096	CycD	6	0,8277
1504	CG3764	CG3764	6	0,8276
1505	CG6392	cmet	6	0,8276
1506	CG12010	CG12010	6	0,8275
1507	CG4076	Nufip	6	0,8271
1508	CG13393	CG13393	6	0,8271
1509	CG3455	Rpt4	6	0,8271
1510	CG10108	phyl	6	0,8270
1511	CG18362	Mio	6	0,8270
1512	CG17612	CG17612	6	0,8270
1513	CG4882	CG4882	6	0,8269
1514	CG18273	CG18273	6	0,8266
1515	CG18217	CG18217	6	0,8266
1516	CG15104	Topors	6	0,8265
1517	CG7761	pcs	6	0,8262
1518	CG1621	CG1621	6	0,8262
1519	CG1965	CG1965	6	0,8262
1520	CG4217	TFAM	6	0,8258
1521	CG6627	Dnz1	6	0,8258
1522	CG5690	CG5690	6	0,8258
1523	CG1666	Hlc	6	0,8258
1524	CG14561	CG14561	6	0,8258
1525	CG7800	CG7800	6	0,8257
1526	CG11970	CG11970	6	0,8257
1527	CG10895	lok	6	0,8256
1528	CG10286	CG10286	6	0,8255
1529	CG4153	eIF-2beta	6	0,8253

1530	CG5018	CG5018	6	0,8253
1531	CG4005	yki	6	0,8252
1532	CG5664	CG5664	6	0,8252
1533	CG7839	CG7839	6	0,8252
1534	CG2201	CG2201	6	0,8251
1535	CG11371	dbr	6	0,8250
1536	CG8333	HLHmgamma	6	0,8249
1537	CG5589	CG5589	6	0,8249
1538	CG6632	Ing3	6	0,8248
1539	CG11280	trn	6	0,8247
1540	CG1512	cul-2	6	0,8247
1541	CG11137	CG11137	6	0,8246
1542	CG14650	CG14650	6	0,8246
1543	CG11825	CG11825	6	0,8244
1544	CG10582	Sin	6	0,8244
1545	CG8064	CG8064	6	0,8243
1546	CG17158	cpb	6	0,8242
1547	CG13162	CG13162	6	0,8242
1548	CG6773	sec13	6	0,8240
1549	CG3166	aop	6	0,8240
1550	CG7494	mRpL1	6	0,8239
1551	CG11781	CG11781	6	0,8238
1552	CG3883	CG3883	6	0,8236
1553	CG3333	Nop60B	6	0,8236
1554	CG8474	Meics	6	0,8233
1555	CG3793	CG3793	6	0,8232
1556	CG7250	Toll-6	6	0,8231
1557	CG9905	gw	6	0,8229
1558	CG9786	hb	6	0,8228
1559	CG5807	CG5807	6	0,8228
1560	CG12022	CG12022	6	0,8227
1561	CG6551	fu	6	0,8226
1562	CG13773	CG13773	6	0,8226
1563	CG10751	robl	6	0,8225
1564	CG5746	CG5746	6	0,8225
1565	CG17678	cta	6	0,8222
1566	CG1448	inx3	6	0,8222
1567	CG6954	CG6954	6	0,8221
1568	CG10620	Tsf2	6	0,8221
1569	CG6736	Ilp4	6	0,8220
1570	CG6841	CG6841	6	0,8215
1571	CG6535	tefu	6	0,8215
1572	CG32300	oxt	6	0,8214
1573	CG3068	aur	6	0,8214
1574	CG14683	CG14683	6	0,8213
1575	CG15319	nej	6	0,8211
1576	CG6391	Aps	6	0,8210
1577	CG9346	CG9346	6	0,8208
1578	CG5041	Tfb4	6	0,8208
1579	CG8987	tam	6	0,8206
1580	CG8777	CG8777	6	0,8204
1581	CG10711	CG10711	6	0,8203
1582	CG1951	CG1951	6	0,8202

1583	CG1703	CG1703	6	0,8201
1584	CG12108	Ppt1	6	0,8199
1585	CG7654	Tom20	6	0,8198
1586	CG13396	fy	6	0,8197
1587	CG6198	CHORD	6	0,8196
1588	CG12238	l(1)G0084	6	0,8195
1589	CG9967	CG9967	6	0,8192
1590	CG12028	dib	6	0,8191
1591	CG1422	p115	6	0,8191
1592	CG10109	L	6	0,8191
1593	CG3917	Grip84	6	0,8189
1594	CG4854	CG4854	6	0,8189
1595	CG1163	RpII18	6	0,8188
1596	CG3978	pnr	6	0,8188
1597	CG10590	CG10590	6	0,8188
1598	CG4813	CG4813	6	0,8188
1599	CG3365	drongo	6	0,8187
1600	CG6171	CG6171	6	0,8187
1601	CG14270	CG14270	6	0,8186
1602	CG4947	Tgt	6	0,8185
1603	CG6697	CG6697	6	0,8183
1604	CG8866	CG8866	6	0,8182
1605	CG10341	CG10341	6	0,8181
1606	CG11184	Upf3	6	0,8180
1607	CG3496	vir	6	0,8179
1608	CG3613	qkr58E-1	6	0,8178
1609	CG5013	CG5013	6	0,8177
1610	CG10686	tral	6	0,8177
1611	CG4591	Tsp86D	6	0,8177
1612	CG5219	mRpL15	6	0,8176
1613	CG6754	nbs	6	0,8175
1614	CG1307	CG1307	6	0,8175
1615	CG8432	Rep	6	0,8174
1616	CG9267	CG9267	6	0,8174
1617	CG7602	DNApol-iota	6	0,8172
1618	CG10191	CG10191	6	0,8171
1619	CG15863	CG15863	6	0,8171
1620	CG4413	CG4413	6	0,8169
1621	CG5546	MED19	6	0,8169
1622	CG2038	CSN7	6	0,8169
1623	CG4065	CG4065	6	0,8169
1624	CG10743	CG10743	6	0,8168
1625	CG3923	Exp6	6	0,8166
1626	CG18247	shark	6	0,8166
1627	CG7480	Pgant35A	6	0,8165
1628	CG12047	mud	6	0,8164
1629	CG2941	CG2941	6	0,8163
1630	CG4074	CG4074	6	0,8163
1631	CG8121	CG8121	6	0,8163
1632	CG5902	CG5902	6	0,8161
1633	CG17943	comm	6	0,8160
1634	CG11784	CG11784	6	0,8160
1635	CG6568	CG6568	6	0,8157

1636	CG13746	MrgBP	6	0,8156
1637	CG4647	mRpL49	6	0,8155
1638	CG17218	CG17218	6	0,8152
1639	CG8247	CG8247	6	0,8149
1640	CG10128	tra2	6	0,8147
1641	CG2331	TER94	6	0,8147
1642	CG10955	CG10955	6	0,8147
1643	CG12948	CG12948	6	0,8146
1644	CG2818	CG2818	6	0,8146
1645	CG12659	CG12659	6	0,8146
1646	CG7879	CG7879	6	0,8145
1647	CG9881	p16-ARC	6	0,8144
1648	CG6685	CG6685	6	0,8143
1649	CG17233	CG17233	6	0,8141
1650	CG15645	CG15645	6	0,8139
1651	CG12759	Dbp45A	6	0,8139
1652	CG3773	CG3773	6	0,8138
1653	CG3004	CG3004	6	0,8138
1654	CG7065	CG7065	6	0,8137
1655	CG14637	abs	6	0,8137
1656	CG17596	S6kII	6	0,8134
1657	CG17697	fz	6	0,8134
1658	CG3353	CG3353	6	0,8133
1659	CG15014	CG15014	6	0,8133
1660	CG4208	XRCC1	6	0,8133
1661	CG1597	CG1597	6	0,8133
1662	CG12267	CG12267	6	0,8132
1663	CG13418	RpI12	6	0,8132
1664	CG14715	CG14715	6	0,8132
1665	CG4495	CG4495	6	0,8129
1666	CG12592	CG12592	6	0,8129
1667	CG2126	CG2126	6	0,8128
1668	CG6443	CG6443	6	0,8128
1669	CG14438	CG14438	6	0,8127
1670	CG2025	CG2025	6	0,8123
1671	CG6181	CG6181	6	0,8122
1672	CG10346	Grip71	6	0,8121
1673	CG11417	CG11417	6	0,8119
1674	CG3497	Su(H)	6	0,8118
1675	CG9304	CG9304	6	0,8116
1676	CG6090	RpL34a	6	0,8115
1677	CG11750	CG11750	6	0,8115
1678	CG5417	Srp14	6	0,8111
1679	CG31731	CG31731	6	0,8110
1680	CG8009	CG8009	6	0,8110
1681	CG7088	bnb	6	0,8109
1682	CG3029	or	6	0,8109
1683	CG1994	l(1)G0020	6	0,8107
1684	CG5181	CG5181	6	0,8106
1685	CG3281	CG3281	6	0,8106
1686	CG12532	Bap	6	0,8104
1687	CG9187	Psf1	6	0,8104
1688	CG14005	CG14005	6	0,8103

1689	CG7479	Aats-leu	6	0,8102
1690	CG12373	mRpL18	6	0,8102
1691	CG1906	CG1906	6	0,8101
1692	CG7785	CG7785	6	0,8098
1693	CG12261	CG12263	6	0,8098
1694	CG2125	ci	6	0,8097
1695	CG6222	su(s)	6	0,8096
1696	CG9695	Dab	6	0,8095
1697	CG6868	tld	6	0,8095
1698	CG8165	CG8165	6	0,8093
1699	CG17343	CG17343	6	0,8091
1700	CG17219	CG17219	6	0,8091
1701	CG12547	CG12547	6	0,8091
1702	CG4889	wg	6	0,8091
1703	CG17829	CG17829	6	0,8090
1704	CG3204	Rap2l	6	0,8090
1705	CG1898	HBS1	6	0,8090
1706	CG2982	CG2982	6	0,8090
1707	CG8361	HLHm7	6	0,8089
1708	CG5708	CG5708	6	0,8089
1709	CG5353	Aats-thr	6	0,8088
1710	CG1793	MED26	6	0,8088
1711	CG12975	CG12975	6	0,8088
1712	CG6715	KP78a	6	0,8087
1713	CG7143	DNApol-eta	6	0,8086
1714	CG14480	CG14480	6	0,8085
1715	CG18332	CSN3	6	0,8085
1716	CG9890	CG9890	6	0,8083
1717	CG5669	CG5669	6	0,8082
1718	CG9143	CG9143	6	0,8082
1719	CG4662	CG4662	6	0,8079
1720	CG4140	CG4140	6	0,8079
1721	CG14896	CG14896	6	0,8079
1722	CG6985	CG6985	6	0,8079
1723	CG5884	par-6	6	0,8078
1724	CG10569	Myt1	6	0,8077
1725	CG9867	CG9867	6	0,8076
1726	CG5429	Atg6	6	0,8076
1727	CG5148	CG5148	6	0,8076
1728	CG1359	CG1359	6	0,8076
1729	CG12320	CG12320	6	0,8073
1730	CG15881	CG15881	6	0,8073
1731	CG3273	sced	6	0,8072
1732	CG6951	CG6951	6	0,8071
1733	CG8004	CG8004	6	0,8069
1734	CG31478	mRpL9	6	0,8069
1735	CG5709	ari-2	6	0,8068
1736	CG10387	tos	6	0,8068
1737	CG4360	CG4360	6	0,8067
1738	CG6932	CSN6	6	0,8067
1739	CG17973	CG17982	6	0,8066
1740	CG7619	Pros54	6	0,8066
1741	CG4491	noc	6	0,8063

1742	CG8600	CG8600	6	0,8063
1743	CG5738	lolal	6	0,8061
1744	CG3851	odd	6	0,8059
1745	CG17273	CG17273	6	0,8058
1746	CG4004	CG4004	6	0,8058
1747	CG4611	CG4611	6	0,8057
1748	CG10435	CG10435	6	0,8056
1749	CG7519	CG7519	6	0,8056
1750	CG9738	Mkk4	6	0,8055
1751	CG1484	fliI	6	0,8054
1752	CG8038	CG8038	6	0,8053
1753	CG6177	ldlCp	6	0,8053
1754	CG18174	Rpn11	6	0,8052
1755	CG6948	Clc	6	0,8052
1756	CG12728	CG12728	6	0,8052
1757	CG3340	Kr	6	0,8052
1758	CG6120	Tsp96F	6	0,8051
1759	CG18042	lmg	6	0,8050
1760	CG17437	wds	6	0,8050
1761	CG5912	arr	6	0,8048
1762	CG12343	CG12343	6	0,8047
1763	CG6195	CG6195	6	0,8046
1764	CG10628	CG10628	6	0,8045
1765	CG3848	trr	6	0,8043
1766	CG5481	lea	6	0,8043
1767	CG10682	vih	6	0,8042
1768	CG1536	CG31640	6	0,8041
1769	CG9688	mRpS18C	6	0,8041
1770	CG10306	CG10306	6	0,8040
1771	CG6323	Tsp97E	6	0,8040
1772	CG31716	CG31716	6	0,8039
1773	CG6631	CG6631	6	0,8039
1774	CG10577	siz	6	0,8039
1775	CG17255	CG17255	6	0,8037
1776	CG3593	r-l	6	0,8036
1777	CG4008	und	6	0,8035
1778	CG33691	CG33691	6	0,8035
1779	CG5855	cni	6	0,8035
1780	CG17328	CG17328	6	0,8035
1781	CG8290	CG8290	6	0,8034
1782	CG11471	Aats-ile	6	0,8033
1783	CG9630	CG9630	6	0,8032
1784	CG2910	nito	6	0,8032
1785	CG9344	CG9344	6	0,8028
1786	CG31550	CG31550	6	0,8028
1787	CG9866	CG9866	6	0,8027
1788	CG7904	put	6	0,8026
1789	CG14026	tkv	6	0,8025
1790	CG6920	mus309	6	0,8025
1791	CG5290	CG5290	6	0,8025
1792	CG8075	Vang	6	0,8024
1793	CG14211	CG14211	6	0,8020
1794	CG6525	CG6525	6	0,8018

1795	CG5554	CG5554	6	0,8018
1796	CG32443	Pc	6	0,8016
1797	CG32533	CG32533	6	0,8014
1798	CG9203	CG9203	6	0,8014
1799	CG9884	oaf	6	0,8013
1800	CG5808	CG5808	6	0,8013
1801	CG9047	CG9047	6	0,8011
1802	CG6915	CG6915	6	0,8011
1803	CG1416	CG1416	6	0,8011
1804	CG14906	CG14906	6	0,8009
1805	CG7435	Arf84F	6	0,8009
1806	CG11763	micr	6	0,8006
1807	CG2984	Pp2C1	6	0,8005
1808	CG6159	sec10	6	0,8004
1809	CG6019	mus308	6	0,8004
1810	CG7840	CG7840	6	0,8004
1811	CG4211	nonA	6	0,8004
1812	CG9634	CG9634	6	0,8004
1813	CG15016	mRpS6	6	0,8000
1814	CG3584	qkr58E-3	6	0,7997
1815	CG7379	CG7379	6	0,7997
1816	CG17712	CG17712	6	0,7996
1817	CG11237	Oseg6	6	0,7996
1818	CG6096	HLHm5	6	0,7995
1819	CG14321	CG14321	6	0,7995
1820	CG7292	Rrp6	6	0,7994
1821	CG7940	CG7940	6	0,7994
1822	CG10933	CG10933	6	0,7992
1823	CG4617	CG4617	6	0,7992
1824	CG16738	slp1	6	0,7991
1825	CG13807	CG13807	6	0,7990
1826	CG6964	Gug	6	0,7988
1827	CG4917	wfs1	6	0,7988
1828	CG16804	CG33671	6	0,7987
1829	CG8194	RNaseX25	6	0,7985
1830	CG17724	CG17724	6	0,7985
1831	CG11660	CG11660	6	0,7985
1832	CG5522	CG5522	6	0,7984
1833	CG11858	CG11858	6	0,7983
1834	CG10328	nonA-l	6	0,7982
1835	CG10489	Pole2	6	0,7981
1836	CG10065	gfzf	6	0,7981
1837	CG18028	lt	6	0,7980
1838	CG3460	Nmd3	6	0,7979
1839	CG8365	E(spl)	6	0,7979
1840	CG6949	mRpL45	6	0,7977
1841	CG10376	CG10376	6	0,7974
1842	CG7341	CG7341	6	0,7974
1843	CG4924	icln	6	0,7972
1844	CG12124	CG12124	6	0,7971
1845	CG2041	lgs	6	0,7970
1846	CG9004	CG9004	6	0,7970
1847	CG11188	CG11188	6	0,7969

1848	CG4567	CG4567	6	0,7968
1849	CG14413	mRpS25	6	0,7968
1850	CG3895	ph-d	6	0,7967
1851	CG1550	CG1550	6	0,7965
1852	CG5498	CG5498	6	0,7965
1853	CG9250	Mpp6	6	0,7965
1854	CG4141	Pi3K92E	6	0,7964
1855	CG7138	r2d2	6	0,7962
1856	CG3033	CG3033	6	0,7962
1857	CG8710	CG8710	6	0,7959
1858	CG3641	CG33331	6	0,7959
1859	CG11030	CG11030	6	0,7959
1860	CG10261	aPKC	6	0,7959
1861	CG12219	CG12219	6	0,7958
1862	CG9282	RpL24	6	0,7957
1863	CG12325	CG12325	6	0,7955
1864	CG7586	Mcr	6	0,7950
1865	CG3025	mof	6	0,7950
1866	CG4332	CG4332	6	0,7949
1867	CG5206	bon	6	0,7948
1868	CG7005	Esp	6	0,7948
1869	CG10326	CG10326	6	0,7948
1870	CG12263	CG30020	6	0,7947
1871	CG4694	her	6	0,7947
1872	CG5525	CG5525	6	0,7947
1873	CG9257	CG9257	6	0,7946
1874	CG2611	CG2611	6	0,7943
1875	CG1100	Rpn5	6	0,7943
1876	CG2310	CG2310	6	0,7943
1877	CG10281	TfIIFalpha	6	0,7941
1878	CG5038	CG5038	6	0,7940
1879	CG5776	CG5776	6	0,7939
1880	CG10011	CG10011	6	0,7938
1881	CG12352	san	6	0,7938
1882	CG17045	yellow-e3	6	0,7937
1883	CG9952	ppa	6	0,7936
1884	CG3722	shg	6	0,7935
1885	CG2218	CG2218	6	0,7935
1886	CG8190	eIF2B-gamma	6	0,7934
1887	CG8434	lbk	6	0,7933
1888	CG14779	pck	6	0,7932
1889	CG8079	CG8079	6	0,7931
1890	CG14217	Tao-1	6	0,7930
1891	CG12935	CG12935	6	0,7930
1892	CG7762	Rpn1	6	0,7927
1893	CG11436	CG11436	6	0,7927
1894	CG7242	CG7242	6	0,7926
1895	CG6033	drk	6	0,7924
1896	CG6683	CG6683	6	0,7924
1897	CG3949	hoip	6	0,7923
1898	CG3298	JhI-1	6	0,7923
1899	CG5104	CG5104	6	0,7923
1900	CG12217	PpV	6	0,7921

1901	CG8276	bin3	6	0,7921
1902	CG4800	Tctp	6	0,7920
1903	CG14464	CG14464	6	0,7919
1904	CG12653	btd	6	0,7919
1905	CG32685	CG32685	6	0,7919
1906	CG7952	gt	6	0,7918
1907	CG2843	CG2843	6	0,7917
1908	CG4164	CG4164	6	0,7914
1909	CG12245	gcm	6	0,7914
1910	CG8470	mRpS30	6	0,7913
1911	CG7504	CG7504	6	0,7912
1912	CG4278	CG4278	6	0,7911
1913	CG3702	CG3702	6	0,7907
1914	CG5093	Doc3	6	0,7906
1915	CG9591	omd	6	0,7906
1916	CG10721	CG10721	6	0,7905
1917	CG3886	Psc	6	0,7902
1918	CG4659	Srp54k	6	0,7902
1919	CG3654	CG3654	6	0,7899
1920	CG3295	CG3295	6	0,7898
1921	CG8184	CG8184	6	0,7898
1922	CG7282	CG7282	6	0,7897
1923	CG14430	CG14430	6	0,7896
1924	CG6099	m4	6	0,7896
1925	CG6539	Dhh1	6	0,7895
1926	CG1344	CG1344	6	0,7892
1927	CG13434	CG13434	6	0,7891
1928	CG9247	CG9247	6	0,7891
1929	CG3969	PR2	6	0,7890
1930	CG12254	MED25	6	0,7889
1931	CG17723	CG17723	6	0,7888
1932	CG1956	R	6	0,7887
1933	CG10914	CG10914	6	0,7885
1934	CG6359	CG6359	6	0,7884
1935	CG2714	crm	6	0,7883
1936	CG8203	Cdk5	6	0,7881
1937	CG1107	auxillin	6	0,7881
1938	CG7048	CG7048	6	0,7879
1939	CG1515	l(1)G0155	6	0,7879
1940	CG11103	CG11103	6	0,7878
1941	CG6038	CG6038	6	0,7876
1942	CG17183	MED30	6	0,7875
1943	CG8706	CG33087	6	0,7874
1944	CG2863	Nle	6	0,7874
1945	CG3065	CG3065	6	0,7874
1946	CG4180	l(2)35Bg	6	0,7873
1947	CG6582	Aac11	6	0,7873
1948	CG1868	CG1868	6	0,7872
1949	CG17361	CG17361	6	0,7872
1950	CG33522	scaf6	6	0,7871
1951	CG8309	Tango7	6	0,7871
1952	CG9795	CG9795	6	0,7869
1953	CG15744	CG15744	6	0,7866

1954	CG9854	hrg	6	0,7866
1955	CG14048	mRpL14	6	0,7866
1956	CG8725	CSN4	6	0,7865
1957	CG5780	CG5780	6	0,7864
1958	CG5904	mRpS31	6	0,7861
1959	CG10123	Top3alpha	6	0,7861
1960	CG2135	CG2135	6	0,7860
1961	CG1641	sisA	6	0,7860
1962	CG14231	CG14231	6	0,7858
1963	CG8390	vlc	6	0,7858
1964	CG1837	CG1837	6	0,7857
1965	CG5130	CG5130	6	0,7853
1966	CG9124	eIF-3p40	6	0,7853
1967	CG10023	Fak56D	6	0,7852
1968	CG7544	CG7544	6	0,7852
1969	CG17721	CG17721	6	0,7850
1970	CG15561	CG15561	6	0,7850
1971	CG7364	CG7364	6	0,7849
1972	CG16734	CG16734	6	0,7848
1973	CG4424	CG4424	6	0,7847
1974	CG5792	CG5792	6	0,7846
1975	CG1763	nod	6	0,7845
1976	CG8580	bhr	6	0,7844
1977	CG6817	foi	6	0,7844
1978	CG11744	mRpS18A	6	0,7843
1979	CG2608	CG2608	6	0,7843
1980	CG6583	CG6583	6	0,7842
1981	CG1789	CG1789	6	0,7842
1982	CG6444	CG6444	6	0,7841
1983	CG10988	l(1)dd4	6	0,7840
1984	CG15435	CG15435	6	0,7837
1985	CG13895	CG13895	6	0,7837
1986	CG31163	CG31163	6	0,7837
1987	CG1245	MED27	6	0,7836
1988	CG12288	CG12288	6	0,7835
1989	CG33207	pxb	6	0,7835
1990	CG6025	Arf72A	6	0,7835
1991	CG2899	ksr	6	0,7833
1992	CG5632	thoc6	6	0,7832
1993	CG12125	CG12125	6	0,7832
1994	CG8485	CG8485	6	0,7832
1995	CG9809	CG9809	6	0,7832
1996	CG3885	CG3885	6	0,7831
1997	CG8269	Dmn	6	0,7831
1998	CG11247	CG11247	6	0,7829
1999	CG7187	Ssdp	6	0,7829
2000	CG9636	CG9636	6	0,7827
2001	CG10107	CG10107	6	0,7826
2002	CG9894	CG9894	6	0,7825
2003	CG10391	Cyp310a1	6	0,7823
2004	CG5055	baz	6	0,7823
2005	CG14074	CG14074	6	0,7820
2006	CG10632	CG10632	6	0,7819

2007	CG9539	Sec61alpha	6	0,7818
2008	CG10330	bgcn	6	0,7818
2009	CG12701	CG12701	6	0,7817
2010	CG9554	eya	6	0,7815
2011	CG8314	CG8314	6	0,7815
2012	CG2247	CG2247	6	0,7813
2013	CG5094	Sgt	6	0,7812
2014	CG15085	edl	6	0,7812
2015	CG6907	CG6907	6	0,7812
2016	CG3806	eIF2B-epsilon	6	0,7812
2017	CG11208	CG11208	6	0,7811
2018	CG5970	CG5970	6	0,7810
2019	CG3779	numb	6	0,7809
2020	CG13001	CG13001	6	0,7808
2021	CG9922	CG9922	6	0,7807
2022	CG4165	CG4165	6	0,7806
2023	CG1291	CG1291	6	0,7806
2024	CG11523	CG11523	6	0,7804
2025	CG9901	Arp14D	6	0,7804
2026	CG11560	CG11560	6	0,7803
2027	CG6998	ctp	6	0,7802
2028	CG5625	CG5625	6	0,7801
2029	CG5323	CG5323	6	0,7800
2030	CG1424	mst	6	0,7799
2031	CG3167	MAN1	6	0,7799
2032	CG10670	Gen	6	0,7799
2033	CG13605	CG13605	6	0,7798
2034	CG2163	Pabp2	6	0,7798
2035	CG6829	Ark	6	0,7797
2036	CG6677	ash2	6	0,7797
2037	CG4756	CG4756	6	0,7796
2038	CG4399	east	6	0,7795
2039	CG6586	tan	6	0,7795
2040	CG8998	Roc2	6	0,7794
2041	CG5515	CG5515	6	0,7789
2042	CG1057	MED31	6	0,7787
2043	CG10083	CG10083	6	0,7786
2044	CG10153	CG10153	6	0,7785
2045	CG15706	CG15706	6	0,7783
2046	CG4012	gek	6	0,7781
2047	CG7956	CG7956	6	0,7780
2048	CG10809	CG10809	6	0,7779
2049	CG1298	CG1298	6	0,7779
2050	CG12399	Mad	6	0,7777
2051	CG4679	CG4679	6	0,7776
2052	CG9586	CG9586	6	0,7776
2053	CG5222	CG5222	6	0,7774
2054	CG14194	CG14194	6	0,7773
2055	CG14671	CG14671	6	0,7773
2056	CG4124	PNUTS	6	0,7772
2057	CG15792	zip	6	0,7771
2058	CG8174	SRPK	6	0,7771
2059	CG1527	RpS14b	6	0,7771

2060	CG12713	CG12713	6	0,7771
2061	CG8609	MED4	6	0,7770
2062	CG5605	eRF1	6	0,7769
2063	CG11444	CG11444	6	0,7766
2064	CG14895	Pak3	6	0,7766
2065	CG9403	jing	6	0,7766
2066	CG4968	CG4968	6	0,7765
2067	CG8337	malpha	6	0,7764
2068	CG4510	Surf6	6	0,7763
2069	CG10449	Catsup	6	0,7763
2070	CG15438	CG15438	6	0,7762
2071	CG5133	Doc1	6	0,7758
2072	CG5012	mRpL12	6	0,7756
2073	CG3457	CG3457	6	0,7754
2074	CG18657	NetA	6	0,7752
2075	CG2101	mRpS35	6	0,7752
2076	CG9987	CG9987	6	0,7750
2077	CG9495	Scm	6	0,7745
2078	CG10495	CG10495	6	0,7744
2079	CG9398	king-tubby	6	0,7740
2080	CG9705	CG9705	6	0,7739
2081	CG6459	CG6459	6	0,7737
2082	CG17265	CG17265	6	0,7736
2083	CG7748	OstStt3	6	0,7734
2084	CG7729	Fit2	6	0,7732
2085	CG4857	CG4857	6	0,7731
2086	CG11888	Rpn2	6	0,7731
2087	CG8896	18w	6	0,7730
2088	CG6694	CG6694	6	0,7730
2089	CG5661	Sema-5c	6	0,7727
2090	CG18013	Psf2	6	0,7727
2091	CG8176	CG8176	6	0,7724
2092	CG7697	CstF-64	6	0,7724
2093	CG14814	CG14814	6	0,7724
2094	CG11927	CG11927	6	0,7723
2095	CG1316	CG1316	6	0,7722
2096	CG7004	fwd	6	0,7722
2097	CG4532	pod1	6	0,7720
2098	CG31122	CG31122	6	0,7719
2099	CG31672	CG31672	6	0,7716
2100	CG18616	CG18616	6	0,7715
2101	CG5671	Pten	6	0,7714
2102	CG31886	CG31886	6	0,7714
2103	CG5092	Tor	6	0,7713
2104	CG3338	CG3338	6	0,7713
2105	CG3874	frc	6	0,7712
2106	CG5753	stau	6	0,7712
2107	CG8340	128up	6	0,7711
2108	CG17680	CG17680	6	0,7710
2109	CG1524	RpS14a	6	0,7708
2110	CG17568	CG17568	6	0,7707
2111	CG2009	bip2	6	0,7705
2112	CG12077	CG12077	6	0,7705

2113	CG4699	CG4699	6	0,7704
2114	CG3149	CG3149	6	0,7703
2115	CG4637	hh	6	0,7703
2116	CG8635	CG8635	6	0,7701
2117	CG1664	sbr	6	0,7700
2118	CG1822	bif	6	0,7699
2119	CG7883	eIF2B-alpha	6	0,7699
2120	CG4996	CG4996	6	0,7693
2121	CG11907	Ent1	6	0,7693
2122	CG12157	Tom40	6	0,7691
2123	CG17603	Taf1	6	0,7691
2124	CG3651	CG3651	6	0,7690
2125	CG15084	CG15084	6	0,7687
2126	CG6098	Lrr47	6	0,7685
2127	CG14803	CG14803	6	0,7684
2128	CG4406	CG4406	6	0,7683
2129	CG9356	CG9356	6	0,7682
2130	CG8366	CG8366	6	0,7682
2131	CG13922	mRpL46	6	0,7681
2132	CG6383	crb	6	0,7677
2133	CG8433	Ext2	6	0,7676
2134	CG8408	CG8408	6	0,7676
2135	CG2762	ush	6	0,7674
2136	CG10635	CG10635	6	0,7672
2137	CG3271	CG3271	6	0,7672
2138	CG7057	AP-50	6	0,7672
2139	CG4261	Hel89B	6	0,7672
2140	CG7842	CG7842	6	0,7672
2141	CG12244	lic	6	0,7672
2142	CG6187	RluA-2	6	0,7671
2143	CG11873	CG11873	6	0,7671
2144	CG17600	CG17600	6	0,7670
2145	CG3443	pcx	6	0,7667
2146	CG11526	CG11526	6	0,7666
2147	CG12877	CG12877	6	0,7666
2148	CG3796	ac	6	0,7663
2149	CG18565	CG32226	6	0,7662
2150	CG15535	CG15535	6	0,7660
2151	CG3827	sc	6	0,7659
2152	CG12788	CG12788	6	0,7658
2153	CG10535	CG10535	6	0,7656
2154	CG10673	CG10673	6	0,7652
2155	CG3766	scat	6	0,7651
2156	CG3891	CG3891	6	0,7651
2157	CG1862	Ephrin	6	0,7650
2158	CG3420	CG3420	6	0,7650
2159	CG8090	CG8090	6	0,7649
2160	CG4908	CG4908	6	0,7648
2161	CG9986	CG9986	6	0,7648
2162	CG7360	Nup58	6	0,7645
2163	CG2245	l(3)s1921	6	0,7645
2164	CG17117	hth	6	0,7645
2165	CG10238	CG10238	6	0,7643

2166	CG6824	ovo	6	0,7639
2167	CG1070	Alh	6	0,7637
2168	CG9099	CG9099	6	0,7635
2169	CG4764	CG4764	6	0,7633
2170	CG11857	CG11857	6	0,7633
2171	CG2019	disp	6	0,7632
2172	CG11494	BtbVII	6	0,7631
2173	CG9559	fog	6	0,7630
2174	CG1768	dia	6	0,7628
2175	CG12767	Dip3	6	0,7628
2176	CG8578	CG8578	6	0,7625
2177	CG12316	CG12316	6	0,7625
2178	CG33232	CG33232	6	0,7622
2179	CG13037	mRpS34	6	0,7622
2180	CG5688	Grip163	6	0,7620
2181	CG8155	CG8155	6	0,7616
2182	CG10757	mRpS18B	6	0,7614
2183	CG3412	slmb	6	0,7608
2184	CG12342	CG12342	6	0,7605
2185	CG7902	bap	6	0,7605
2186	CG5241	CG5241	6	0,7603
2187	CG2108	Rab23	6	0,7603
2188	CG16833	CG16833	6	0,7602
2189	CG14216	CG14216	6	0,7602
2190	CG5281	CG5281	6	0,7600
2191	CG7925	tko	6	0,7600
2192	CG17262	CG17262	6	0,7600
2193	CG16807	CG16807	6	0,7600
2194	CG31638	CG31638	6	0,7600
2195	CG6446	Sema-1b	6	0,7599
2196	CG10484	Dox-A2	6	0,7599
2197	CG7212	cdm	6	0,7599
2198	CG7206	CG7206	6	0,7598
2199	CG10479	CG10479	6	0,7594
2200	CG12058	mxc	6	0,7593
2201	CG11874	CG11874	6	0,7593
2202	CG2904	ec	6	0,7592
2203	CG15897	CG15897	6	0,7592
2204	CG17081	CG17081	6	0,7590
2205	CG11307	insc	6	0,7590
2206	CG12938	CG12938	6	0,7590
2207	CG7554	comm2	6	0,7589
2208	CG6137	aub	6	0,7588
2209	CG7101	CG7101	6	0,7588
2210	CG3388	gsb	6	0,7588
2211	CG5072	Cdk4	6	0,7583
2212	CG6341	Ef1beta	6	0,7583
2213	CG11007	CG11007	6	0,7581
2214	CG4049	CG4049	6	0,7581
2215	CG7067	NitFhit	6	0,7580
2216	CG8675	CG8675	6	0,7578
2217	CG2712	CG2712	6	0,7578
2218	CG10965	Corp	6	0,7572

2219	CG3758	esg	6	0,7572
2220	CG8235	CG8235	6	0,7572
2221	CG5562	gbb	6	0,7570
2222	CG8491	kto	6	0,7569
2223	CG5960	CG32560	6	0,7568
2224	CG4646	CG4646	6	0,7566
2225	CG5444	Taf4	6	0,7565
2226	CG4904	Pros35	6	0,7562
2227	CG12676	ed	6	0,7557
2228	CG1696	l(1)G0269	6	0,7557
2229	CG5248	loco	6	0,7557
2230	CG10594	spo	6	0,7557
2231	CG4300	CG4300	6	0,7557
2232	CG5146	CG5146	6	0,7555
2233	CG12324	RpS15Ab	6	0,7555
2234	CG6719	CG6719	6	0,7554
2235	CG5514	CG5514	6	0,7554
2236	CG10189	CG10189	6	0,7552
2237	CG10283	CG10283	6	0,7551
2238	CG13852	mats	6	0,7550
2239	CG10595	d	6	0,7547
2240	CG17904	CG17904	6	0,7545
2241	CG16910	key	6	0,7545
2242	CG1486	CG1486	6	0,7545
2243	CG6246	nub	6	0,7544
2244	CG4488	wee	6	0,7543
2245	CG11324	CG11329	6	0,7543
2246	CG9296	CG9296	6	0,7543
2247	CG8344	RpIII128	6	0,7542
2248	CG2219	CG2219	6	0,7541
2249	CG17642	mRpL48	6	0,7540
2250	CG8679	CG8679	6	0,7539
2251	CG8617	CG8617	6	0,7539
2252	CG8704	dpn	6	0,7538
2253	CG7215	CG7215	6	0,7536
2254	CG9669	CG9669	6	0,7536
2255	CG1972	CG1972	6	0,7536
2256	CG14728	sad	6	0,7535
2257	CG10274	CG10274	6	0,7533
2258	CG8411	gcl	6	0,7528
2259	CG9883	CG9883	6	0,7526
2260	CG9936	skd	6	0,7525
2261	CG6963	gish	6	0,7523
2262	CG5263	smg	6	0,7522
2263	CG5465	MED16	6	0,7522
2264	CG31012	CG31012	6	0,7521
2265	CG9240	CG9240	6	0,7519
2266	CG2988	ems	6	0,7518
2267	CG12129	CG12129	6	0,7515
2268	CG4194	CG4194	6	0,7514
2269	CG18542	CG18542	6	0,7514
2270	CG7787	CG7787	6	0,7513
2271	CG17807	CG17807	6	0,7513

2272	CG6689	CG6689	6	0,7512
2273	CG17941	ds	6	0,7512
2274	CG7148	CG7148	6	0,7511
2275	CG14939	CG14939	6	0,7510
2276	CG8078	CG8078	6	0,7509
2277	CG1575	CG1575	6	0,7509
2278	CG17931	CG17931	6	0,7508
2279	CG2244	MTA1-like	6	0,7505
2280	CG6382	Elf	6	0,7504
2281	CG12936	CG12936	6	0,7501
2282	CG4293	CG4293	6	0,7498
2283	CG7071	CG7071	6	0,7498
2284	CG12005	Mms19	6	0,7497
2285	CG30169	CG30169	6	0,7497
2286	CG5202	escl	6	0,7496
2287	CG9715	CG9715	6	0,7494
2288	CG6699	beta'Cop	6	0,7493
2289	CG7369	CG7369	6	0,7493
2290	CG5813	chif	6	0,7491
2291	CG17569	gry	6	0,7489
2292	CG9088	lid	6	0,7488
2293	CG15524	CG15524	6	0,7488
2294	CG10640	Uev1A	6	0,7486
2295	CG7628	CG7628	6	0,7485
2296	CG2813	CG2813	6	0,7483
2297	CG12082	CG12082	6	0,7482
2298	CG9330	CG9330	6	0,7480
2299	CG5978	CG5978	6	0,7479
2300	CG3839	l(1)sc	6	0,7478
2301	CG16868	CG16868	6	0,7477
2302	CG4061	CG4061	6	0,7476
2303	CG10603	mRpL13	6	0,7476
2304	CG4825	CG4825	6	0,7476
2305	CG3312	Rnp4F	6	0,7476
2306	CG3289	Ptpa	6	0,7475
2307	CG12818	CG12818	6	0,7474
2308	CG5355	CG5355	6	0,7469
2309	CG9493	Pez	6	0,7467
2310	CG1896	CG1896	6	0,7465
2311	CG3756	CG3756	6	0,7465
2312	CG12230	car	6	0,7464
2313	CG6623	CG6623	6	0,7464
2314	CG4497	CG4497	6	0,7463
2315	CG14725	CG32803	6	0,7462
2316	CG3561	CG3561	6	0,7462
2317	CG10733	loj	6	0,7461
2318	CG10492	CG10492	6	0,7459
2319	CG3760	CG3760	6	0,7458
2320	CG10990	CG10990	6	0,7458
2321	CG2530	corto	6	0,7458
2322	CG8191	CG8191	6	0,7457
2323	CG6339	rad50	6	0,7452
2324	CG14232	CG14232	6	0,7450

2325	CG10338	CG10338	6	0,7448
2326	CG3102	l(1)G0232	6	0,7448
2327	CG9414	Rep4	6	0,7444
2328	CG9848	CG30193	6	0,7444
2329	CG13367	CG13366	6	0,7443
2330	CG4045	CG4045	6	0,7443
2331	CG6983	CG6983	6	0,7440
2332	CG10270	D19B	6	0,7439
2333	CG32016	CG32016	6	0,7436
2334	CG14286	CG14286	6	0,7430
2335	CG6215	CkIIalpha-i1	6	0,7429
2336	CG6764	CG6764	6	0,7429
2337	CG12032	CG32278	6	0,7428
2338	CG13880	mRpL17	6	0,7427
2339	CG12202	Nat1	6	0,7427
2340	CG17187	CG17187	6	0,7423
2341	CG17556	CG17556	6	0,7422
2342	CG4620	unk	6	0,7421
2343	CG11136	CG11136	6	0,7421
2344	CG10228	Pcf11	6	0,7418
2345	CG4616	CG4616	6	0,7416
2346	CG11993	Mst85C	6	0,7413
2347	CG2791	CG2791	6	0,7412
2348	CG5147	CG5147	6	0,7410
2349	CG8831	CG8831	6	0,7410
2350	CG1320	mRpL23	6	0,7410
2351	CG6169	CG6169	6	0,7406
2352	CG5057	MED10	6	0,7406
2353	CG1659	unc-119	6	0,7405
2354	CG7331	CG7331	6	0,7404
2355	CG4730	CG4730	6	0,7403
2356	CG12664	ld14	6	0,7403
2357	CG5412	CG5412	6	0,7401
2358	CG9305	CG9305	6	0,7401
2359	CG18011	CG18011	6	0,7400
2360	CG8963	CG8963	6	0,7398
2361	CG11989	Ard1	6	0,7397
2362	CG10973	CG10973	6	0,7396
2363	CG14341	CG14341	6	0,7395
2364	CG2079	Dok	6	0,7395
2365	CG10585	CG10585	6	0,7386
2366	CG5742	CG5742	6	0,7386
2367	CG13384	CG13384	6	0,7385
2368	CG3107	CG3107	6	0,7384
2369	CG5483	CG5483	6	0,7382
2370	CG3678	CG3678	6	0,7374
2371	CG12363	Dlc90F	6	0,7370
2372	CG1577	mRpL52	6	0,7369
2373	CG13784	CG13784	6	0,7368
2374	CG7824	CG7824	6	0,7368
2375	CG3558	CG3558	6	0,7367
2376	CG17765	CG17765	6	0,7365
2377	CG12107	CG12107	6	0,7363

2378	CG3501	CG3501	6	0,7359
2379	CG9205	CG9205	6	0,7359
2380	CG16932	Eps-15	6	0,7358
2381	CG7147	kuz	6	0,7358
2382	CG15345	CG15345	6	0,7356
2383	CG5676	CG5676	6	0,7356
2384	CG11170	CG11170	6	0,7355
2385	CG9885	dpp	6	0,7353
2386	CG5064	Srp68	6	0,7351
2387	CG7565	CG7565	6	0,7351
2388	CG7895	tin	6	0,7349
2389	CG7556	CG7556	6	0,7349
2390	CG31043	gukh	6	0,7349
2391	CG14750	Vps25	6	0,7348
2392	CG1401	cul-5	6	0,7348
2393	CG10366	CG10366	6	0,7348
2394	CG11926	CG11926	6	0,7345
2395	CG12054	CG12054	6	0,7344
2396	CG2937	mRpS2	6	0,7343
2397	CG3379	His4r	6	0,7342
2398	CG11454	CG11454	6	0,7341
2399	CG7668	CG7668	6	0,7340
2400	CG12096	CG12096	6	0,7340
2401	CG1388	CG32104	6	0,7339
2402	CG12253	CG12253	6	0,7338
2403	CG4317	Mipp2	6	0,7337
2404	CG8614	Neos	6	0,7335
2405	CG6522	CG6522	6	0,7334
2406	CG15112	ena	6	0,7334
2407	CG6597	CG6597	6	0,7332
2408	CG7354	mRpS26	6	0,7332
2409	CG2448	FucT6	6	0,7328
2410	CG12878	btz	6	0,7328
2411	CG2852	CG2852	6	0,7322
2412	CG2189	Dfd	6	0,7321
2413	CG3421	RhoGAP93B	6	0,7321
2414	CG5872	CG5872	6	0,7320
2415	CG3024	torp4a	6	0,7318
2416	CG15012	CG15012	6	0,7315
2417	CG4221	CG4221	6	0,7314
2418	CG8490	CG8490	6	0,7314
2419	CG13151	CG13151	6	0,7302
2420	CG14414	CG14414	6	0,7300
2421	CG1709	Vha100-1	6	0,7300
2422	CG7049	CG7049	6	0,7299
2423	CG8014	Rme-8	6	0,7297
2424	CG3632	CG3632	6	0,7290
2425	CG10466	CG10466	6	0,7289
2426	CG2939	slp2	6	0,7288
2427	CG16973	msn	6	0,7284
2428	CG11050	CG11050	6	0,7283
2429	CG3249	CG3249	6	0,7282
2430	CG10084	swm	6	0,7282

2431	CG3073	l(1)G0144	6	0,7281
2432	CG8516	CG8516	6	0,7281
2433	CG18683	CG31033	6	0,7278
2434	CG7650	CG7650	6	0,7278
2435	CG13410	mRpL35	6	0,7275
2436	CG1487	krz	6	0,7272
2437	CG13126	CG13126	6	0,7265
2438	CG7558	Arp66B	6	0,7264
2439	CG3744	CG3744	6	0,7262
2440	CG4237	Gap69C	6	0,7257
2441	CG3445	phol	6	0,7256
2442	CG12379	CG12379	6	0,7254
2443	CG7127	exo70	6	0,7253
2444	CG9075	eIF-4a	6	0,7251
2445	CG10127	CG30077	6	0,7244
2446	CG14543	CG14543	6	0,7243
2447	CG32428	CG32428	6	0,7243
2448	CG1708	cos	6	0,7243
2449	CG1594	hop	6	0,7239
2450	CG8145	CG8145	6	0,7235
2451	CG14812	CG14812	6	0,7232
2452	CG14121	CG14121	6	0,7232
2453	CG10545	Gbeta13F	6	0,7221
2454	CG5212	Pli	6	0,7220
2455	CG14283	mRpL55	6	0,7220
2456	CG10648	CG10648	6	0,7219
2457	CG12734	CG12734	6	0,7217
2458	CG9520	CG9520	6	0,7213
2459	CG7398	Trn	6	0,7209
2460	CG8002	rictor	6	0,7207
2461	CG11180	CG11180	6	0,7204
2462	CG4937	RhoGAP15B	6	0,7202
2463	CG9635	RhoGEF2	6	0,7192
2464	CG30183	CG30183	6	0,7189
2465	CG32210	CG32210	6	0,7189
2466	CG7793	Sos	6	0,7186
2467	CG7261	CG7261	6	0,7182
2468	CG17611	eIF6	6	0,7181
2469	CG6567	CG6567	6	0,7181
2470	CG1821	RpL31	6	0,7178
2471	CG11882	CG11882	6	0,7177
2472	CG11887	StIP	6	0,7177
2473	CG8010	CG8010	6	0,7175
2474	CG6136	CG6136	6	0,7172
2475	CG9791	CG9791	6	0,7172
2476	CG4204	Elongin-B	6	0,7168
2477	CG1378	tll	6	0,7166
2478	CG9954	maf-S	6	0,7162
2479	CG8833	CG8833	6	0,7158
2480	CG10255	Lap1	6	0,7157
2481	CG12156	Rab39	6	0,7155
2482	CG8182	GalNAc-T1	6	0,7150
2483	CG2924	CG2924	6	0,7139

2484	CG15224	CkIIbeta	6	0,7136
2485	CG8629	CG8629	6	0,7136
2486	CG6859	CG6859	6	0,7132
2487	CG4157	Rpn12	6	0,7115
2488	CG18549	CG18549	6	0,7114
2489	CG18802	alpha-Man-II	6	0,7112
2490	CG9515	CG9515	6	0,7111
2491	CG4619	CG4619	6	0,7109
2492	CG9951	CG9951	6	0,7107
2493	CG4925	CG4925	6	0,7098
2494	CG8458	wntD	6	0,7073
2495	CG9063	CG9063	6	0,7067
2496	CG4792	Dcr-1	6	0,7066
2497	CG10922	La	6	0,7060
2498	CG33785	CG33785	6	0,7058
2499	CG8135	CG8135	6	0,7058
2500	CG11901	Ef1gamma	6	0,7044
2501	CG17660	CG17660	6	0,7042
2502	CG1233	CG1233	6	0,7035
2503	CG5721	CG5721	6	0,7031
2504	CG8300	CG8300	6	0,7029
2505	CG9386	CG9386	6	0,7019
2506	CG5682	CG5682	6	0,7017
2507	CG31998	CG31998	6	0,7017
2508	CG6017	CG6017	6	0,6994
2509	CG6005	CG6005	6	0,6984
2510	CG7950	CG7950	6	0,6956
2511	CG7849	CG7849	6	0,6939
2512	CG8743	CG8743	6	0,6892
2513	CG1116	CG1116	6	0,6825
2514	CG1824	CG1824	6	0,6806
2515	CG2662	CG2662	5	0,7900
2516	CG12051	Act42A	5	0,7671
2517	CG2669	CG2669	5	0,7659
2518	CG5354	pie	5	0,7652
2519	CG8291	CG8291	5	0,7615
2520	CG12581	CG12581	5	0,7594
2521	CG5650	Pp1-87B	5	0,7588
2522	CG3240	Rad1	5	0,7573
2523	CG4350	APC4	5	0,7573
2524	CG8212	Ranbp11	5	0,7559
2525	CG4816	qkr54B	5	0,7520
2526	CG8825	gkt	5	0,7520
2527	CG8734	CG8734	5	0,7500
2528	CG6588	Fas1	5	0,7493
2529	CG6401	CG6401	5	0,7481
2530	CG4599	Tpr2	5	0,7477
2531	CG7223	htl	5	0,7470
2532	CG14513	yemalpha	5	0,7469
2533	CG6395	Csp	5	0,7466
2534	CG4622	CG4622	5	0,7461
2535	CG14711	CG14711	5	0,7461
2536	CG13588	CG13588	5	0,7457

2537	CG3158	spn-E	5	0,7449
2538	CG1362	cdc2rk	5	0,7448
2539	CG7830	CG7830	5	0,7437
2540	CG5300	Klp31E	5	0,7434
2541	CG5680	bsk	5	0,7432
2542	CG2248	Rac1	5	0,7432
2543	CG11295	l(2)dtl	5	0,7431
2544	CG12121	CG12121	5	0,7430
2545	CG16876	CG16876	5	0,7429
2546	CG18321	miple2	5	0,7414
2547	CG10703	CG10703	5	0,7394
2548	CG11844	CG11844	5	0,7381
2549	CG4841	CG4841	5	0,7376
2550	CG4553	CG4553	5	0,7376
2551	CG17957	Sry-alpha	5	0,7366
2552	CG7343	btsz	5	0,7361
2553	CG10795	CG10795	5	0,7356
2554	CG3780	Spx	5	0,7347
2555	CG10739	pigeon	5	0,7344
2556	CG7163	mkg-p	5	0,7343
2557	CG18156	CG18156	5	0,7342
2558	CG31855	CG31855	5	0,7341
2559	CG8384	gro	5	0,7341
2560	CG3039	ogre	5	0,7341
2561	CG2211	CG2211	5	0,7338
2562	CG1227	CG1227	5	0,7330
2563	CG6982	CG6982	5	0,7297
2564	CG6205	por	5	0,7296
2565	CG5916	CG5916	5	0,7294
2566	CG1764	CG1764	5	0,7290
2567	CG3305	CG3305	5	0,7286
2568	CG1901	mav	5	0,7282
2569	CG17800	Dscam	5	0,7280
2570	CG9318	CG9318	5	0,7280
2571	CG12721	CG12721	5	0,7271
2572	CG8639	Cirl	5	0,7259
2573	CG2103	pgant6	5	0,7258
2574	CG9114	CG9114	5	0,7258
2575	CG10622	Sucb	5	0,7255
2576	CG8386	CG8386	5	0,7250
2577	CG9286	CG9286	5	0,7249
2578	CG10463	CG10463	5	0,7248
2579	CG5170	Dp1	5	0,7241
2580	CG5359	CG5359	5	0,7237
2581	CG15889	Ravus	5	0,7235
2582	CG12864	Su(var)2-HP2	5	0,7234
2583	CG9298	CG9298	5	0,7232
2584	CG7891	CG7891	5	0,7229
2585	CG13379	CG13367	5	0,7227
2586	CG31211	CG31211	5	0,7219
2587	CG2637	Fs(2)Ket	5	0,7211
2588	CG6645	CG32054	5	0,7207
2589	CG9429	Crc	5	0,7205

2590	CG2168	RpS3A	5	0,7202
2591	CG9648	Max	5	0,7202
2592	CG12236	CG12236	5	0,7200
2593	CG2411	ptc	5	0,7193
2594	CG8183	Khc-73	5	0,7190
2595	CG11680	mle	5	0,7186
2596	CG6194	CG6194	5	0,7186
2597	CG13163	CG13163	5	0,7186
2598	CG9774	rok	5	0,7183
2599	CG3695	MED23	5	0,7178
2600	CG2956	twi	5	0,7168
2601	CG3342	CG3342	5	0,7164
2602	CG17186	CG17186	5	0,7163
2603	CG33214	CG33214	5	0,7161
2604	CG9663	CG9663	5	0,7160
2605	CG8865	Rgl	5	0,7159
2606	CG10555	CG10555	5	0,7157
2607	CG5091	CG5091	5	0,7155
2608	CG8818	CG8818	5	0,7154
2609	CG5123	W	5	0,7154
2610	CG15015	Cip4	5	0,7154
2611	CG18024	SoxN	5	0,7151
2612	CG11518	pygo	5	0,7149
2613	CG11589	VhaM9.7-1	5	0,7146
2614	CG11306	CG11307	5	0,7142
2615	CG10874	CG10874	5	0,7135
2616	CG10210	tst	5	0,7130
2617	CG12499	CG12499	5	0,7130
2618	CG3587	CG3587	5	0,7129
2619	CG18428	CG18431	5	0,7128
2620	CG7428	halo	5	0,7128
2621	CG9491	Gef26	5	0,7118
2622	CG10009	Noa36	5	0,7106
2623	CG2713	CG2713	5	0,7103
2624	CG11622	Rlip	5	0,7094
2625	CG9893	l(2)06496	5	0,7094
2626	CG1531	CG1531	5	0,7088
2627	CG7427	CG7427	5	0,7087
2628	CG3054	l(2)k05819	5	0,7085
2629	CG7692	CG7692	5	0,7082
2630	CG4537	CG4537	5	0,7076
2631	CG7770	CG7770	5	0,7074
2632	CG15168	CG15168	5	0,7071
2633	CG2162	CG2162	5	0,7070
2634	CG8258	CG8258	5	0,7067
2635	CG3279	CG3279	5	0,7066
2636	CG12204	CG12204	5	0,7066
2637	CG13604	CG13604	5	0,7065
2638	CG4408	CG4408	5	0,7058
2639	CG11399	CG11399	5	0,7058
2640	CG32133	CG32133	5	0,7057
2641	CG7033	CG7033	5	0,7052
2642	CG16916	Rpt3	5	0,7046

2643	CG6325	CG6325	5	0,7045
2644	CG8862	CG8862	5	0,7044
2645	CG2957	mRpS9	5	0,7041
2646	CG18361	dsh	5	0,7040
2647	CG12863	CG12863	5	0,7038
2648	CG13295	CG13295	5	0,7038
2649	CG9977	CG9977	5	0,7038
2650	CG12190	CG12190	5	0,7034
2651	CG1916	Wnt2	5	0,7034
2652	CG4429	Rbp2	5	0,7031
2653	CG12234	Ranbp21	5	0,7030
2654	CG4743	CG4743	5	0,7026
2655	CG17060	Rab10	5	0,7025
2656	CG4426	ast	5	0,7022
2657	CG5794	CG5794	5	0,7017
2658	CG2252	fs(1)h	5	0,7009
2659	CG8152	CG8152	5	0,7008
2660	CG17370	CG17370	5	0,7001
2661	CG5434	Srp72	5	0,6995
2662	CG13775	CG13775	5	0,6992
2663	CG5187	Doc2	5	0,6991
2664	CG4707	CG4707	5	0,6990
2665	CG4196	CG4196	5	0,6990
2666	CG10068	CG10068	5	0,6984
2667	CG3633	mRpS29	5	0,6982
2668	CG17337	CG17337	5	0,6980
2669	CG13896	CG13896	5	0,6977
2670	CG4079	Taf11	5	0,6973
2671	CG3373	Hmu	5	0,6973
2672	CG7861	CG7861	5	0,6973
2673	CG5385	CG5385	5	0,6973
2674	CG10428	CG10428	5	0,6972
2675	CG2656	CG2656	5	0,6970
2676	CG7649	Neu3	5	0,6967
2677	CG30295	Ipk1	5	0,6966
2678	CG14648	CG14648	5	0,6965
2679	CG4820	CG4820	5	0,6964
2680	CG10652	RpL30	5	0,6961
2681	CG2976	CG2976	5	0,6961
2682	CG9655	nes	5	0,6957
2683	CG14967	CG14967	5	0,6954
2684	CG12921	mRpL42	5	0,6954
2685	CG10825	CG10825	5	0,6953
2686	CG12213	CG12213	5	0,6952
2687	CG1529	CG1529	5	0,6950
2688	CG2720	Hop	5	0,6948
2689	CG8912	Psi	5	0,6945
2690	CG8651	trx	5	0,6943
2691	CG11652	CG11652	5	0,6939
2692	CG4575	CG4575	5	0,6938
2693	CG18593	viaf	5	0,6929
2694	CG6549	fws	5	0,6907
2695	CG12753	CG12753	5	0,6907

2696	CG1636	CG1636	5	0,6901
2697	CG8887	ash1	5	0,6899
2698	CG1241	Atg2	5	0,6899
2699	CG9704	Nrt	5	0,6894
2700	CG14980	CG14980	5	0,6893
2701	CG7192	CG7192	5	0,6889
2702	CG9773	CG9773	5	0,6888
2703	CG15027	CG15027	5	0,6885
2704	CG9585	CG32982	5	0,6885
2705	CG10997	Clic	5	0,6882
2706	CG3297	mnd	5	0,6875
2707	CG8359	CG8359	5	0,6872
2708	CG3225	CG3225	5	0,6870
2709	CG8977	Cctgamma	5	0,6867
2710	CG6796	CG6796	5	0,6866
2711	CG10302	bsf	5	0,6859
2712	CG7949	CG7949	5	0,6854
2713	CG14411	CG14411	5	0,6852
2714	CG7623	sll	5	0,6845
2715	CG14030	CG14030	5	0,6843
2716	CG14937	CG14937	5	0,6842
2717	CG5341	sec6	5	0,6842
2718	CG15747	CG15747	5	0,6839
2719	CG4558	CG4558	5	0,6831
2720	CG15415	CG15415	5	0,6816
2721	CG1559	Upf1	5	0,6816
2722	CG2995	CG2995	5	0,6808
2723	CG10053	CG10053	5	0,6805
2724	CG4901	CG4901	5	0,6803
2725	CG10961	Traf2	5	0,6800
2726	CG11975	CG11975	5	0,6795
2727	CG6896	MYPT-75D	5	0,6787
2728	CG8389	CG8389	5	0,6775
2729	CG12211	Opbp	5	0,6762
2730	CG10915	CG10915	5	0,6761
2731	CG12141	Aats-lys	5	0,6757
2732	CG15432	CG15432	5	0,6755
2733	CG12304	CG12304	5	0,6731
2734	CG8824	fdl	5	0,6696
2735	CG9841	EfSec	5	0,6659
2736	CG5374	T-cp1	5	0,6645
2737	CG5949	DNApol-delta	4	0,7679
2738	CG14789	O-fut2	4	0,7470
2739	CG9012	Chc	4	0,7336
2740	CG7892	nmo	4	0,7334
2741	CG17359	CG17359	4	0,7304
2742	CG1034	bcd	4	0,7295
2743	CG3903	Gli	4	0,7280
2744	CG4973	CG4973	4	0,7276
2745	CG6104	m2	4	0,7236
2746	CG5818	mRpL4	4	0,7231
2747	CG18414	ph-p	4	0,7227
2748	CG3242	sob	4	0,7221

2749	CG8895	Rtnl1	4	0,7215
2750	CG15436	CG15436	4	0,7211
2751	CG13388	Akap200	4	0,7210
2752	CG15738	CG15738	4	0,7201
2753	CG16908	CG16908	4	0,7198
2754	CG4771	CG4771	4	0,7172
2755	CG7614	Mat1	4	0,7172
2756	CG4158	wor	4	0,7158
2757	CG7664	crp	4	0,7157
2758	CG12153	Hira	4	0,7147
2759	CG7457	CG7457	4	0,7146
2760	CG11312	homer	4	0,7129
2761	CG7230	rib	4	0,7128
2762	CG3992	srp	4	0,7128
2763	CG13484	CG13484	4	0,7123
2764	CG9177	eIF5	4	0,7120
2765	CG4609	fax	4	0,7119
2766	CG2277	CG2277	4	0,7106
2767	CG3375	stumps	4	0,7106
2768	CG5651	CG5651	4	0,7102
2769	CG13051	CG13051	4	0,7101
2770	CG2864	Parg	4	0,7096
2771	CG5524	CG5524	4	0,7092
2772	CG10038	CG10038	4	0,7091
2773	CG18250	Dg	4	0,7081
2774	CG1133	cort	4	0,7080
2775	CG9138	SP1070	4	0,7077
2776	CG4420	CG4420	4	0,7076
2777	CG1007	emc	4	0,7075
2778	CG31646	CG31646	4	0,7074
2779	CG12972	CG12972	4	0,7073
2780	CG4946	CG4946	4	0,7067
2781	CG1374	tsh	4	0,7063
2782	CG14685	Cap-H2	4	0,7055
2783	CG3595	sqh	4	0,7052
2784	CG14291	CG14291	4	0,7051
2785	CG11412	CG11412	4	0,7046
2786	CG6345	CG6345	4	0,7043
2787	CG10289	CG10289	4	0,7038
2788	CG4953	CG4953	4	0,7024
2789	CG14434	CG14434	4	0,7023
2790	CG11416	ori	4	0,7021
2791	CG9204	Ate1	4	0,7015
2792	CG12598	Adar	4	0,7015
2793	CG1751	Spase25	4	0,7014
2794	CG8297	CG8297	4	0,7011
2795	CG10295	Pak	4	0,7009
2796	CG7156	CG7156	4	0,7005
2797	CG9948	CG9948	4	0,7001
2798	CG14548	HLHmbeta	4	0,6998
2799	CG3810	CG3810	4	0,6998
2800	CG3437	CG3437	4	0,6992
2801	CG5695	jar	4	0,6966

2802	CG1609	Gcn2	4	0,6948
2803	CG3500	CG3500	4	0,6942
2804	CG4257	Stat92E	4	0,6938
2805	CG9328	CG9328	4	0,6934
2806	CG11427	rb	4	0,6933
2807	CG18319	ben	4	0,6931
2808	CG2970	CG2970	4	0,6926
2809	CG18176	defl	4	0,6917
2810	CG11367	CG11367	4	0,6916
2811	CG34374	Rapgap1	4	0,6904
2812	CG11940	CG11940	4	0,6900
2813	CG10811	eIF-4G	4	0,6892
2814	CG4336	rux	4	0,6890
2815	CG6185	CG6185	4	0,6890
2816	CG5924	CG5924	4	0,6880
2817	CG16980	RhoGAP71E	4	0,6880
2818	CG5474	SsRbeta	4	0,6879
2819	CG13671	CG13671	4	0,6877
2820	CG10324	CG10324	4	0,6876
2821	CG3792	CG3792	4	0,6875
2822	CG7974	CG7974	4	0,6868
2823	CG5099	msi	4	0,6866
2824	CG10166	CG10166	4	0,6866
2825	CG7986	Atg18	4	0,6865
2826	CG4416	hang	4	0,6857
2827	CG4719	tankyrase	4	0,6851
2828	CG2943	CG2943	4	0,6835
2829	CG9849	CG9849	4	0,6835
2830	CG34380	CG34380	4	0,6834
2831	CG13550	CG13550	4	0,6828
2832	CG4880	CG4880	4	0,6825
2833	CG8460	CG8460	4	0,6821
2834	CG9553	chic	4	0,6820
2835	CG14657	CG14657	4	0,6819
2836	CG13777	milt	4	0,6815
2837	CG1651	Ank	4	0,6804
2838	CG10423	RpS27	4	0,6800
2839	CG3035	cm	4	0,6798
2840	CG1360	CG1360	4	0,6797
2841	CG1643	Atg5	4	0,6796
2842	CG9086	CG9086	4	0,6788
2843	CG9662	CG9662	4	0,6785
2844	CG2226	CG30372	4	0,6776
2845	CG11986	CG11986	4	0,6768
2846	CG4272	CG4272	4	0,6763
2847	CG9144	CG9144	4	0,6756
2848	CG11003	CG32113	4	0,6752
2849	CG17059	CG17059	4	0,6747
2850	CG9735	Aats-trp	4	0,6744
2851	CG5345	Eip55E	4	0,6744
2852	CG2095	CG2095	4	0,6734
2853	CG5284	CG5284	4	0,6733
2854	CG7506	CG7506	4	0,6728

2855	CG8351	CG8351	4	0,6718
2856	CG13201	ix	4	0,6706
2857	CG4884	CG4884	4	0,6698
2858	CG6338	Ets97D	4	0,6696
2859	CG3697	mei-9	4	0,6691
2860	CG6808	CG6808	4	0,6688
2861	CG9139	CG9139	4	0,6685
2862	CG10978	jagn	4	0,6685
2863	CG5039	CG5039	4	0,6684
2864	CG6769	CG6769	4	0,6679
2865	CG7066	CG7066	4	0,6678
2866	CG7518	CG7518	4	0,6677
2867	CG7825	Rad17	4	0,6676
2868	CG8325	l(2)k14710	4	0,6665
2869	CG4101	CG4101	4	0,6663
2870	CG10470	CG10470	4	0,6636
2871	CG31989	Cap-D3	4	0,6634
2872	CG3386	CG3386	4	0,6634
2873	CG1528	gammaCop	4	0,6633
2874	CG6965	mthl5	4	0,6631
2875	CG12757	CG31126	4	0,6631
2876	CG11958	Cnx99A	4	0,6626
2877	CG9730	mRpL21	4	0,6617
2878	CG1691	Imp	4	0,6612
2879	CG17737	CG17737	4	0,6610
2880	CG7872	CG7872	4	0,6600
2881	CG10072	sgl	4	0,6594
2882	CG6224	dbo	4	0,6577
2883	CG15008	CG15008	4	0,6566
2884	CG7927	CG7927	4	0,6560
2885	CG12114	CG12114	4	0,6556
2886	CG8811	muskelin	4	0,6553
2887	CG1839	CG1839	4	0,6546
2888	CG18596	CG18596	4	0,6531
2889	CG1934	ImpE2	4	0,6527
2890	CG5339	CG5339	4	0,6512
2891	CG3075	CG3075	4	0,6509
2892	CG10798	dm	4	0,6505
2893	CG12081	CG12081	4	0,6480
2894	CG4084	l(2)not	3	0,7127
2895	CG8965	CG8965	3	0,7114
2896	CG10079	Egfr	3	0,7087
2897	CG9272	CG9272	3	0,7029
2898	CG10695	Pat1	3	0,7022
2899	CG11605	jbug	3	0,7016
2900	CG14992	Ack	3	0,7007
2901	CG15390	CG15390	3	0,7005
2902	CG3322	LanB2	3	0,6967
2903	CG10521	NetB	3	0,6966
2904	CG11614	nkd	3	0,6954
2905	CG34401	CG34401	3	0,6914
2906	CG7013	ARP-like	3	0,6906
2907	CG4807	ab	3	0,6897

2908	CG8346	HLHm3	3	0,6892
2909	CG11577	CG11577	3	0,6892
2910	CG14873	CG33455	3	0,6889
2911	CG6520	CG6520	3	0,6869
2912	CG5186	slim	3	0,6865
2913	CG17598	CG17598	3	0,6862
2914	CG14182	CG14182	3	0,6858
2915	CG12007	CG12007	3	0,6857
2916	CG10236	LanA	3	0,6852
2917	CG18105	ETH	3	0,6849
2918	CG4291	CG4291	3	0,6835
2919	CG9322	CG9322	3	0,6830
2920	CG16717	CG16717	3	0,6816
2921	CG8714	sut1	3	0,6810
2922	CG12011	CG12011	3	0,6800
2923	CG4531	argos	3	0,6777
2924	CG9062	CG9062	3	0,6774
2925	CG5126	CG5126	3	0,6766
2926	CG8885	CG8885	3	0,6764
2927	CG3059	NTPase	3	0,6762
2928	CG5520	Gp93	3	0,6760
2929	CG7334	Sug	3	0,6758
2930	CG5530	CG32594	3	0,6753
2931	CG7823	RhoGDI	3	0,6753
2932	CG8561	CG8561	3	0,6746
2933	CG4006	Akt1	3	0,6738
2934	CG12186	Aats-pro	3	0,6726
2935	CG17188	Hs6st	3	0,6725
2936	CG3262	CG3262	3	0,6724
2937	CG8801	CG8801	3	0,6723
2938	CG17964	pan	3	0,6714
2939	CG33129	CG33129	3	0,6712
2940	CG18177	CG18177	3	0,6709
2941	CG7246	CG7246	3	0,6703
2942	CG3495	Gmer	3	0,6700
2943	CG3253	CG3253	3	0,6698
2944	CG30089	CG30089	3	0,6698
2945	CG9353	mRpL54	3	0,6697
2946	CG10637	Nak	3	0,6697
2947	CG15817	CG15817	3	0,6690
2948	CG17223	alpha4GT1	3	0,6689
2949	CG3201	Mlc-c	3	0,6689
2950	CG12235	Arp11	3	0,6687
2951	CG6842	CG6842	3	0,6681
2952	CG9433	Xpd	3	0,6681
2953	CG6700	CG6700	3	0,6678
2954	CG18111	Obp99a	3	0,6676
2955	CG8610	Cdc27	3	0,6675
2956	CG11156	mus101	3	0,6673
2957	CG3947	CG3947	3	0,6670
2958	CG10309	pad	3	0,6670
2959	CG13800	CG13800	3	0,6665
2960	CG5247	Irbp	3	0,6665

2961	CG11447	CG11447	3	0,6659
2962	CG7414	CG7414	3	0,6652
2963	CG6410	CG6410	3	0,6651
2964	CG17802	CG17802	3	0,6650
2965	CG8503	CG8503	3	0,6650
2966	CG10741	CG10741	3	0,6649
2967	CG7436	Nmt	3	0,6646
2968	CG14033	CG14033	3	0,6640
2969	CG17046	klar	3	0,6635
2970	CG1925	mus205	3	0,6629
2971	CG10268	CG10268	3	0,6626
2972	CG16745	CG32486	3	0,6622
2973	CG6621	CG6621	3	0,6618
2974	CG1084	Cont	3	0,6616
2975	CG8931	CG8931	3	0,6613
2976	CG8443	CG8443	3	0,6610
2977	CG12018	CG12018	3	0,6609
2978	CG10234	Hs2st	3	0,6606
2979	CG13551	CG13551	3	0,6593
2980	CG33506	CG33506	3	0,6591
2981	CG9931	Nsf2	3	0,6591
2982	CG12177	CG12177	3	0,6590
2983	CG6890	Tollo	3	0,6590
2984	CG9537	DLP	3	0,6587
2985	CG8097	CG8097	3	0,6583
2986	CG5706	CG5706	3	0,6582
2987	CG6097	rt	3	0,6582
2988	CG9418	CG9418	3	0,6574
2989	CG1115	CG1115	3	0,6570
2990	CG16952	CG16952	3	0,6570
2991	CG9115	mtm	3	0,6568
2992	CG7725	rogdi	3	0,6568
2993	CR15910	ric8b	3	0,6567
2994	CG9031	Rsu-1	3	0,6567
2995	CG3631	CG3631	3	0,6561
2996	CG15871	mRpL38	3	0,6557
2997	CG13344	CG13344	3	0,6546
2998	CG7425	eff	3	0,6545
2999	CG7884	CG7884	3	0,6539
3000	CG15743	CG15743	3	0,6539
