# Supplementary material for: Identification of Drosophila Mitotic Genes by Combining Co-Expression Analysis and RNA Interference
Source: PLoS Genet. 2008 Jul 18;4(7):e1000126. doi: 10.1371/journal.pgen.1000126 (PMC2537813; doi:10.1371/journal.pgen.1000126)
Supplement: Table S2 — Distribution of 164 known mitotic genes in coexpression lists with cid, glu, eb1, zw10, ida and sti. (0.03 MB PDF) [file pgen.1000126.s018.pdf]

Supplementary Table 2. Distribution of 164 known mitotic genes in coexpression lists with *cid* (centromeric histone CenpA), *glu* (SMC4 subunit of the condensin complex), *eb1* (microtubule plus end binding protein), *zw10* (component of the spindle checkpoint RZZ complex), *ida* (APC5 subunit of the APC/C) and *sti* (Citron kinase); consensus refers to a consensus list generated from the single gene coexpression lists.

Gene classes: KSC, kinetochore and spindle checkpoint; CSP, centrosome and spindle poles; MMF, multiple mitotic functions; SPA, spindle assembly; CSC, chromosome structure and condensation; CYT, cytokinesis. The numbers of genes in each class are reported in the third column; the numbers in columns 4-10 are the percentages of genes of a given functional class included in the indicated rank interval.

			percent of genes included in the rank interval						
Rank	Gene class	# of genes	*cid*	*glu*	*eb1*	*zw10*	*ida*	*sti*	consensus
1 - 500	KSC	18	39	28	17	39	44	33	28
	CSP	19	21	32	32	26	26	21	32
	MMF	32	31	38	19	19	34	28	28
	SPA	24	33	25	17	4	25	21	25
	CSC	33	52	46	15	52	55	37	46
	CYT	38	18	24	16	13	21	24	21
	**Total**	**164**	**32**	**32**	**18**	**25**	**34**	**28**	**32**
501 - 1000	KSC	18	11	11	11	11	6		17
	CSP	19	26	15		16	16	16	10
	MMF	32	13	9	28	19	9	12	13
	SPA	24	4	12	12	21	12	8	12
	CSC	33	21	21	27	12	15	15	21
	CYT	38	8	5	13	16	5	2	8
	**Total**	**164**	**13**	**12**	**17**	**16**	**10**	**9**	**14**
1001 - 2000	KSC	18	17	33	33	17	17	39	22
	CSP	19	21	21	21	32	32	21	32
	MMF	32	12	9	12	19	16	22	16
	SPA	24	13	17	29	33	21	21	25
	CSC	33	9	18	40	18	12	30	15
	CYT	38	11	10	10	5	8	13	3
	**Total**	**164**	**13**	**17**	**23**	**19**	**16**	**23**	**16**
> 2000	KSC	18	33	28	39	33	33	28	33
	CSP	19	32	32	47	26	26	42	26
	MMF	32	44	44	41	43	41	38	43
	SPA	24	50	46	42	42	42	50	38
	CSC	33	18	15	18	18	18	18	18
	CYT	38	63	61	61	66	66	61	68
	**Total**	**164**	**42**	**39**	**42**	**40**	**40**	**40**	**38**
