# Supplementary material for: Identification of Drosophila Mitotic Genes by Combining Co-Expression Analysis and RNA Interference
Source: PLoS Genet. 2008 Jul 18;4(7):e1000126. doi: 10.1371/journal.pgen.1000126 (PMC2537813; doi:10.1371/journal.pgen.1000126)
Supplement: Table S3 — Individual ranks of 164 mitotic genes in coexpression lists with cid, glu, eb1, zw10, ida and sti. (0.06 MB PDF) [file pgen.1000126.s019.pdf]

Supplementary Table 3. Individual ranks of 164 mitotic genes in coexpression lists with *cid* , *glu* , *eb1, zw10, ida* and *sti;* consensus refers to a consensus list generated from the single gene coexpression lists. n.f. (not found) means that the gene was not included in the microarray data used to generate our coexpression lists. The numbers in the columns are the ranks of the genes in the coexpression lists. (K), kinase.

**Kinetochore and spindle checkpoint (KSC)**								
		**gene ranks**						
**Gene name**	**CG**	***cid***	***glu***	***eb1***	***zw10***	***ida***	***sti***	**consensus**
bubR1 (K)	7838	162	4	271	266	63	28	8
bub3	7581	36	13	169	433	154	57	15
Ndc80	9938	427	383	902	35	149	462	211
l(1)G0237	1558	495	412	436	291	433	342	293
Nuf2	8902	637	175	904	764	371	159	384
Mad-2	17498	74	583	1231	803	35	1193	458
cid/cenpA	13329	1	1122	1885	487	107	1650	520
zw10	9900	534	1172	1856	1	230	1495	529
rod	1569	399	547	1635	124	819	1154	713
Spc105	11451	1349	1094	1469	1620	1505	446	1246
cmet	6392	1516	1186	2281	1201	1353	1369	1505
mitch	7242	1544	1916	2507	2356	1042	1902	1894
NnfIR-2	13434	2098	1624	1631	2424	2540	1224	1927
dm (Mis12)	18156	2602	2510	3161	1510	2021	2561	2557
Bub1	14030	3326	2764	2980	2585	2753	2596	2715
Spindly	15415	2559	2884	3264	2980	2780	2656	2720
Cenp-C	31258	4541	4480	5017	4926	4640	4720	4734
cana	4831	7170	6998	7065	6474	6329	7457	6913
NnfIR-1	31658	n.f.	n.f.	n.f.	n.f.	n.f.	n.f.	n.f.
zwilch	18729	n.f.	n.f.	n.f.	n.f.	n.f.	n.f.	n.f.

**Centrosome & spindle poles (CSP)**								
		**gene ranks**						
**Gene name**	**CG**	***cid***	***glu***	***eb1***	***zw10***	***ida***	***sti***	**consensus**
asp	6875	86	97	473	436	57	249	66
Sas-4	10061	37	492	1005	120	131	469	182
gtub23C	3157	55	348	333	729	177	525	196
asl	2919	410	95	496	965	454	171	311
msps	5000	539	497	194	257	886	702	409
skpa	16983	1061	536	49	365	1088	677	550
cnn	4832	1297	481	376	1191	1175	211	752
Grip75	6176	768	843	1528	939	157	1021	775
SAK/PLK4	7186	1251	963	1146	1935	1001	1116	1220
aur (K)	3068	546	1541	2418	1763	911	1995	1573
Grip84	3917	801	2072	2310	90	1051	2575	1593
mud	12047	926	1649	2190	1892	642	2262	1628
vih	10682	1500	1312	1406	2628	2167	1507	1767
l(1)dd4/Dgrip91	10988	2101	1893	2439	1579	1656	2285	1983
slmb	3412	2105	2007	2058	2820	2128	2291	2183
sas-6	15524	2106	2540	2983	1716	1829	2529	2293
tacc	9765	4339	3829	3604	4012	4315	3800	3978
CG3216	3216	5899	5937	5978	5927	5574	5920	5864
cp309/d-plp	33957	5384	6595	5033	5249	6763	4697	6826

**Multiple mitotic functions (MMF)**								
		**gene ranks**						
**Gene name**	**CG**	***cid***	***glu***	***eb1***	***zw10***	***ida***	***sti***	**consensus**
Incenp	12165	53	3	530	308	61	72	14
ik2 (K)	2615	581	229	66	65	304	131	80
polo (K)	12306	52	12	507	600	85	453	83

fzy	4274	132	32	187	742	212	152	88
ran	1404	199	64	29	395	412	397	109
Cks30A (K)	3738	84	190	667	432	37	671	162
CycA	5940	240	96	605	458	128	563	199
Borr	4454	93	151	564	819	219	482	225
ial	6620	252	213	800	794	235	75	257
CycB	3510	263	251	898	315	313	1148	401
Myb	9045	868	314	9	1080	1326	258	538
dnt (K)	17559	652	463	290	1363	971	265	594
ida/APC5	10850	396	1232	2269	572	1	1729	697
Cdc37	12019	955	818	576	1470	880	609	841
Pp2A-29B	17291	1427	1237	461	1302	1167	1136	1112
mts (P)	7109	1054	842	1270	1557	1379	1040	1168
mr	3060	1095	1321	1594	834	806	1544	1175
Src64B (K)	7524	1555	978	952	1601	1641	852	1262
gek (K)	4012	2449	2003	1466	2165	2474	1685	2046
wee (K)	4488	2209	2450	2120	2179	1893	2784	2244
nmo (K)	7892	2854	2113	1743	3097	3044	1687	2740
Gcn2 (K)	1609	3027	2792	2803	2213	2543	3150	2802
Lis-1	8440	3797	3562	2904	3914	4001	3393	3358
Gl	9206	3806	4016	3890	3240	3424	4106	3777
CG10967 (K)	10967	4146	3656	3588	3760	3903	3693	3795
mnb (K)	7826	4884	4872	4809	5225	5495	4834	5003
Ilk (K)	10504	5331	5348	4804	5047	5368	5399	5213
lkb1 (K)	9374	5425	5614	5578	5450	5147	5860	5511
Nrk (K)	4007	5762	5487	5353	5851	5905	5115	5541
CG8565 (K)	8565	10936	11071	11213	11806	10961	11398	11237
Dhc64C	7507	11869	11871	12318	12373	11826	12127	12120
Pka-C2 (K)	12066	11784	12113	12036	3859	12161	12568	12277

**Spindle assembly (SPA)**								
		**gene ranks**						
**Gene name**	**CG**	***cid***	***glu***	***eb1***	***zw10***	***ida***	***sti***	**consensus**
Klp61F	9191	189	15	391	750	248	23	94
ncd	7831	61	27	893	583	226	77	140
Klp67A	10923	269	103	697	445	150	190	166
cdc16	6759	311	294	365	699	132	666	275
hpo (K)	11228	377	312	344	1124	812	432	473
Eb1	3265	998	564	1	999	1104	489	500
grp (K)	17161	160	690	1002	678	187	1576	595
Cdk9 (K)	5179	450	442	625	1357	670	894	657
Klp3A	8590	461	708	1588	1135	324	1117	805
Subito	12298	1468	1450	2161	1386	594	1442	1411
CG1951 (K)	1951	1593	1830	1653	1025	1032	2229	1582
tkv (K)	14026	2229	1860	1399	1648	2286	1206	1789
CG1344 (K)	1344	2421	2098	1859	1360	1839	1990	1926
Cdk5 (K)	8203	2082	2191	1482	2155	1638	2164	1936
nod	1763	1428	2118	2872	1316	1067	2574	1975
gish (K)	6963	2695	1896	1965	2095	2950	2087	2261
Cdc27	8610	3154	2777	2909	3381	3084	2409	2955
wdb	5643	3646	3280	3438	3117	3556	3543	3504
MAPk-Ak2 (K)	3086	4092	3824	3072	3843	3938	3824	3773
Klp10A	1453	3085	3750	4040	3939	3519	3306	3819
for (K)	10033	4917	4786	4594	4564	4560	4947	4738
fry (K)	7693	8633	8334	8235	7500	7713	8453	8150
CG15072 (K)	15072	10619	10701	11312	10714	10168	10467	10622
Klp59C	3219	12342	12041	12646	12837	11859	12467	12455
chb/Mast/Orbit	32435	n.f.	n.f.	n.f.	n.f.	n.f.	n.f.	n.f.

Chromosome structure and condensation (CSC)								
		gene ranks						
**Gene name**	**CG**	***cid***	***glu***	***eb1***	***zw10***	***ida***	***sti***	**consensus**
Mcm5	4082	12	50	262	37	33	217	1
mus209/PCNA	9193	10	16	412	158	86	182	4
E(z)	6502	41	155	452	14	38	314	6
Mcm2	7538	25	51	618	57	19	278	7
dpa/Mcm4	1616	7	61	467	114	90	308	13
glu/SMC4	11397	309	1	689	308	112	259	43
Rfc40	14999	4	295	1102	30	48	1051	135
Mcm10	9241	195	277	632	70	51	790	142
prod	18608	336	148	466	349	609	61	205
Su(var)2-10	8068	293	124	760	291	123	665	219
Top2	10223	212	264	700	554	325	142	234
thr	5785	426	456	1171	160	44	440	274
Orc5	7833	28	732	1606	78	10	1048	307
barr/CAP-H	10726	185	125	568	1263	480	367	369
SMC2	10212	501	162	737	1009	755	262	472
Orc2	3041	248	608	1893	123	4	1232	476
CAP-D2	1911	386	482	1078	1172	518	237	547
pita/spdk	3941	1371	597	1248	184	569	876	737
pim	5052	640	558	1221	559	462	1436	742
SMC1/cohesin	6057	948	893	773	641	1468	582	853
gwl	7719	707	878	1723	481	139	1693	888
Cap-G	17054	974	675	1532	1354	785	540	929
Sse	10583	716	1245	2022	342	207	1366	948
CDC45L	3658	194	1535	2184	74	368	2137	1123
wapl	3707	1390	1013	1224	977	1309	1447	1204
eco	8598	878	1275	1731	1421	1495	1409	1347
Mkk4 (K)	9738	2050	1755	1249	1425	2247	1796	1750
san	12352	1271	1846	1916	2329	1349	2484	1881
Ppi-87B	5650	2732	2070	928	2450	3051	1749	2521
mus101	11156	2300	3190	3596	2333	2528	3286	2956
tws	6235	6395	5787	5500	5570	6488	5893	5894
CG9488 (K)	9488	6064	6394	6458	6271	5888	6130	6196
Invadolysin	3953	7100	7015	7079	6686	6526	6865	6882
Rad21	17436	n.f.	n.f.	n.f.	n.f.	n.f.	n.f.	n.f.

Cytokinesis (CYT)								
		gene ranks						
**Gene name**	**CG**	***cid***	***glu***	***eb1***	***zw10***	***ida***	***sti***	**consensus**
scra	2092	85	2	207	246	136	8	3
Bap55	6546	163	42	98	331	191	17	21
feo	11207	108	68	678	252	64	441	87
tum	13345	180	152	664	138	174	406	138
sti	10522	811	211	544	576	478	1	207
Nipped-A	2905	490	216	38	382	540	648	267
pav	1258	780	121	528	530	484	32	290
pbl	8114	222	444	1143	580	18	479	292
pnut	8705	763	402	23	742	885	348	410
tsr	4254	1126	820	53	891	1289	1020	815
Orc-6	1584	392	1284	2199	873	408	1675	831
Sep2	4173	1127	724	198	1650	1508	387	932
Tl	5490	1899	1159	978	1599	1757	1550	1502
baz	5055	2609	1824	1254	2143	2715	1133	2004
zip	15792	2452	1773	1300	2363	2774	1439	2057
dia	1768	1595	2014	2412	2777	9475	2628	2108
rok (K)	9774	3047	2378	2871	2166	2563	2411	2696
sqh	3595	3177	2740	1533	2747	3281	2328	2783

chic	9553	2952	2631	2214	3186	3355	2724	2834
gCop	1528	3217	3046	2561	2989	3274	2793	2873
cap/act up	5061	3054	3229	2860	3504	2659	3436	3112
rho1	8416	4110	3766	3259	3566	4132	3621	3758
Rab35	9575	3541	3071	2893	2835	3516	3043	3978
Syx1A	5448	4258	4002	3880	4337	4589	3950	4169
Aip1	10724	4551	4587	4414	4599	4405	4899	4561
Drp1	3210	5368	5619	5375	5048	5494	5763	5442
Fad2	7923	5663	5713	5588	5354	5713	6162	5683
kek1 (K)	12283	5602	5748	5948	6118	5869	5418	5767
Phkgamma (K)	1830	9880	10473	10657	6188	9758	6476	6579
Toll-4	18241	6832	6891	7255	6186	6950	7164	7000
kst	12008	7943	7048	6288	6976	8361	6529	7109
Rop	15811	7460	7230	6336	7333	7556	7072	7121
CG7236 (K)	7236	7806	7196	7410	7498	6882	7005	7282
Syx5	4214	7582	7415	6931	7361	7449	7733	7395
Snap	6625	9695	9016	7665	7359	9939	8861	9011
shi	18102	9725	8804	9006	9477	9456	8959	9220
cmp44E	8739	9983	9859	9288	9475	9436	9938	9595
gio	5269	10399	10267	10084	10104	11137	10368	10333
