# Supplementary material for: Identification of Drosophila Mitotic Genes by Combining Co-Expression Analysis and RNA Interference
Source: PLoS Genet. 2008 Jul 18;4(7):e1000126. doi: 10.1371/journal.pgen.1000126 (PMC2537813; doi:10.1371/journal.pgen.1000126)
Supplement: Table S4 — List of the 155 mitotic genes detected in the screen and primers used for dsRNA synthesis. (0.05 MB PDF) [file pgen.1000126.s020.pdf]

Supplementary Table 4. List of the 155 genes detected in the screen and primers used for dsRNA synthesis. Each primer contained the T7 polymerase binding site (not shown), and led to the synthesis of a single dsRNA. The number of off-target (OT) sequences in each primer was calculated using a program available at http://flyrnai.org/RNAi_find_frag_free.html.

PHC, phenocluster: NM, no mitotic division; CA, chromosome aberrations; CS, chromosome segregation; CC, chromosome condensation; SA, spindle assembly; SC, spindle assembly and chromosome condensation; CY, cytokinesis.

Rank	Gene	CG	PHC	Off-target	PCR size	Primer 5'	Primer 3'
20	*RPA2*	9273	NM	3	600	gttgttcggcat	tttgatttacat
40	*geminin*	3183	NM	2	750	aaattgcatgtg	acctccaccaaa
64	*Cdc2c*	10498	NM	0	990	gcacctacggta	atggggaatgca
69	*DebB*	16792	NM	6	350	tcggcacgcttg	tgtgggtgtatg
108	*ran*	1404	NM	4	1000	ccagtgtgccgt	ccagctattgcg
110	*Bx42*	8264	NM	2	1090	caccggagaact	tgcacatcaacg
169	*prp8*	8877	NM	0	800	aatggattaagg	cgtcagcttgta
188	*SF1*	5836	NM	0	850	tcgcgctggtga	atacgggcgcca
196	*CycA*	5940	NM	1	740	tcggtgagtttg	ttgtcaatcggc
264	*stg*	1395	NM	5	870	atcagcagcgat	atctgctcggtg
280	*RpII140*	3180	NM	0	550	tgctaattgccc	ctaagagcagcc
350	*CycE*	3938	NM	1	730	catcgtccgcct	aggacgacgag
851	*RpII215*	1554	NM	1	550	acgtcgcgctgt	acggtcacgatc
4	*mus209*	9193	CA	0	580	tggcttcgacaa	atgtgacccaga
5	*CG15220*	15220	CA	0	540	ccgctgcgatag	atgcctttgatc
11	*RnrS*	8975	CA	1	700	cgccatttcat	tatcatctccat
12	*BEAF-32*	10159	CA	0	630	tggcgcattgcg	ccaagacgctga
125	*noi*	2925	CA	2	880	cggagattgccg	cctccacatcgc
134	*RfC40*	14999	CA	1	950	acgacaagcgca	cggcaatgcaga
156	*DNAprim*	5553	CA	1	750	tcgtgggtgtta	acgtgcacatag
158	*Dp*	4654	CA	0	600	aatcccatacgc	taccacctacac
165	*CG12050*	12050	CA	1	750	tcaaagtatggt	ttcataacatct
199	*dnk*	5452	CA	3	990	cgggttgaatca	ctgttggttac
216	*Su(var)2-10*	8068	CA	0	750	agcccaagcgtc	acgacgcttagc
228	*CG13427*	13427	CA	0	640	ccctggttgtag	aagttcgtggcc
260	*DDB1*	7769	CA	1	880	aggtcaaccaca	aacgtatcgtcg
272	*RpA70*	9633	CA	1	700	atctggcgccta	atcacaggctgc
288	*CG10354*	10354	CA	0	850	agttcctgccga	cacagccctgat
299	*Orc5*	7833	CA	3	730	cggccattgaaa	ctccaagtatgt
304	*DNApol*	6349	CA	0	1100	ccaactaaccac	agttcgacagcg
376	*CG17383*	17383	CA	2	620	gtgaacttctgc	cctttccacaca
385	*okr*	3736	CA	2	740	ctgtagtactca	attacggtggta
396	*CG11906*	11906	CA	3	800	caattgctccgt	gcttcgcacaaa
410	*CG2260*	2260	CA	1	1120	acacagaaacgg	tctccagatagg
417	*Mtor*	8274	CA	1	970	atctgcacacct	gtagataaggcc
419	*woc*	5965	CA	0	1040	aagctactccag	cagttgatggag
435	*cul-4*	8711	CA	2	520	acacgtacgtga	ttgcgtcgagga
444	*Taf6*	9348	CA	0	600	aactagcggagg	ggaagcatttcg
464	*Dcp-1*	5370	CA	0	650	agttgcgctgat	tgaggcacggta
480	*His3.3B*	8989	CA	4	750	ctcgtactaagc	agaagaaggagc
505	*CG8878*	8878	CA	0	450	aagtgcatcgcg	atcctgcgatag
540	*CG6197*	6197	CA	0	570	cgccgaataatg	agtgcaccttgt

546	*l (2) NC136*	8426	CA	2	750	tggttgagcgcg	ctagccctccaa
580	*Ts*	3181	CA	0	540	tgggcactctgt	cgatgttgaagg
660	*CG1939*	1939	CA	0	580	acgccagggtat	ttaggaacagcc
667	*CG17446*	17446	CA	1	580	tggttgcgagga	tgccttgtagct
682	*ppan*	5786	CA	2	970	atgaaccgcatc	gatcgctgtcag
716	*c12.1*	12135	CA	2	700	atctgagtgcac	cgtcattgacga
735	*CG6480*	6480	CA	0	620	ctggatcttcca	ctccgtatcgat
743	*CG7757*	7757	CA	0	1120	agcatgtgcgcg	cctcaagcacgg
778	*CG2685*	2685	CA	3	670	cgcaagtggaga	tggcatgcgtat
813	*CG6686*	6686	CA	6	550	tgccggtacagg	acatcgccggct
823	*SMC1*	6057	CA	0	650	gacatccggaag	cagcctgtgatg
846	*CG7003*	7003	CA	2	610	agaatctccaga	tgctcttatcgt
850	*CG32066*	32066	CA	0	520	attccacggaga	ccaacaaacgct
866	*CG4785*	4785	CA	0	730	tctggaaacctg	tcttccgcaact
937	*CG6854*	6854	CA	1	770	attcggttcccg	atcaacgcctcc
313	*dup*	8171	CS1	0	730	aactggtcgtgc	tgccggcttata
8	*Bub1*	7838	CS2	1	850	tccattatcgac	tcaggtatctt
15	*Bub3*	7581	CS2	1	600	ggagcccatcag	tggccagggcgc
450	*dmt*	8374	CS2	0	970	cgacaatgcagt	acacgatcatcg
517	*mit(1)15*	9900	CS2	3	900	cgaatgtcaaga	tctcgtacttct
687	*rod*	1569	CS2	2	950	agggccacctga	atggcagagcac
81	*CG16941*	16941	CS3	1	850	gcaatgatgccg	cttcgtccatgt
93	*U2af50*	9998	CS3	0	860	gatcgacgccat	tcaccacattgg
208	*CG9938*	9938	CS3	1	820	atggcagcttgg	cggttaacaggc
213	*CG3058*	3058	CS3	0	1050	ccattctctccg	caccaatgcgta
229	*CG6876*	6876	CS3	0	950	cggagaagctgg	tttgggcagcgg
261	*CG3605*	3605	CS3	0	700	acgaggacgatg	ttccgtctggga
287	*l(1)G0237*	1558	CS3	3	550	aatacgtgggca	ttggccatattc
354	*CG10418*	10418	CS3	0	470	cccacaagccac	caccctgcactc
358	*SmD3*	8427	CS3	1	490	ccaaagtgccca	gagcgagcacgc
374	*CG8902*	8902	CS3	0	840	ctgttcccagtg	cgttatcagtcg
392	*CG6015*	6015	CS3	0	970	atcacctgctgc	acactacatcgc
394	*CG18591*	18591	CS3	1	700	ctggtcgatagt	cagcgcatttgg
488	*CG13298*	13298	CS3	1	830	acattcatcccg	cgcaaatgtctg
509	*cid*	13329	CS3	0	500	acaacaatcggc	ccatcaatgcca
525	*CG10754*	10754	CS3	0	540	atccgtacttca	atcttaggttcg
566	*CG8233*	8233	CS3	0	500	tagctcttggtg	aacgggacagaa
573	*CG5931*	5931	CS3	0	790	aacttcagcctg	acctgattacgc
656	*CG8241*	8241	CS3	1	600	cctgcttactcg	cattgggctcct
102	*Ote*	5581	CS4	0	850	aaccagtgcgtc	cctcggatacgc
262	*U2af38*	3582	CS4	1	530	cgatcattgcca	atggctcccatc
269	*thr*	5785	CS4	1	710	cattgattgacg	aatacctctatg
391	*CycB*	3510	CS4	0	700	acagcaatctgt	aagtgaatcggc
648	*CG2807*	2807	CS4	0	880	gactcctggaca	cataagcaacgg
758	*MBD-R2*	10042	CS4	1	900	agatcgaagcag	ccatgtccagca
769	*CG1420*	1420	CS4	5	500	ttatcaagggcg	ctcgtttgcgat
838	*U2A'*	1406	CS4	1	570	accagtttgata	atctgcgataga
911	*Sse*	10583	CS4	0	800	aattctcccgcg	tcgactccacga
88	*fzy*	4274	CS5	1	800	cgtctctaaatg	ccaaggatccca
402	*CG4266*	4266	CS5	2	920	gtgtgaaaagca	ttcagacgcgct
563	*CG3221*	3221	CS5	0	550	caagattcactg	ttcagcatgctc
569	*kin17*	5649	CS5	1	570	cttcaagtgcca	tcgcttggctag
585	*Pros26.4*	5289	CS5	2	570	gttcatccgcaa	actagcttgggt

671	*ida*	10850	CS5	0	600	agcttcccttgt	gccaagagcaga
775	*Klp3A*	8590	CS5	0	860	gccatgagtcac	ctgtgctcagca
835	*CG11419*	11419	CS5	0	500	atataaccgcca	atagtgcttgcc
18	*Mcm7*	4978	CC1	1	670	gctgactccaga	gtccttagacat
149	*Mcm3*	4206	CC1	1	830	atcagggcatct	gcgccaaactct
683	*Cap*	9802	CC1	2	870	agcagccaacagt	ggcataaaggtc
43	*glu*	11397	CC2	0	740	agttggaaaagg	gcaaacacaagt
360	*barr*	10726	CC2	1	900	gtatcgtctcct	tggactccaatg
462	*SMC2*	10212	CC2	1	1000	gtcaagaccta	acaagggacaga
533	*CAP-D2*	1911	CC2	2	560	gcactacatcca	tgccttgcatct
892	*Cap-G*	17054	CC2	0	850	aactgatcaccg	ccgcgaacgatt
231	*Top2*	10223	CC3	0	830	ccttgcgctgct	caaagattgcgc
466	*Orc2*	3041	CC3	4	650	cggttacaagac	agttgccagcat
855	*gwl*	7719	CC3	0	900	gcgacgactaat	gtggaatgccgg
19	*Map60*	1825	SA1	0	800	aagccccacaaa	cgagttgcatag
80	*ik2*	2615	SA1	1	1100	ataatcagccgt	tccattgatcgt
256	*CG14781*	14781	SA1	0	620	cgtccaaggatg	tggaagggtctg
293	*tho2*	31671	SA1	0	1040	tcccaacacgtt	cgggaatcgtca
305	*Nnp-1*	12396	SA1	2	880	aggacgcttcac	catgtgaacgtc
306	*eIF-3p66*	10161	SA1	0	700	aggacgagacaa	ggagttgcatga
369	*Trip1*	8882	SA1	0	650	cacatcagggac	gccacgagcgtt
399	*msps*	5000	SA1	0	1100	taggcaccatgt	cgatgtgtcgat
423	*BTub56b*	9277	SA1	4	1000	ccagatcttcag	tactgctggtac
452	*Int6*	9677	SA1	2	800	ccaccgacattc	ttgacgatccag
490	*Eb1*	3265	SA1	3	650	acgaggagtatt	ttcggctcttgt
557	*CG1234*	1234	SA1	3	820	aagacaaggagg	cgtccttagacc
603	*CG8950*	8950	SA1	1	1000	ggatgaggaggg	atgccggacact
659	*CG17293*	17293	SA1	1	600	gagaacacggac	tccattcgtgct
693	*eIF3-S10*	9805	SA1	1	970	cactgatctgac	tccggatacatg
779	*CG8636*	8636	SA1	2	500	agatcatcatgc	atcaagtggtcg
826	*mars*	17064	SA1	1	580	tgaactcgcgct	aactgctgcaga
950	*CG4865*	4865	SA1	1	850	gcaccatggacg	agatcctccaca
193	*gTub23C*	3157	SA2	1	800	cgagatcagtga	cagtcaacagtt
263	*NippedA*	2905	SA2	0	560	accgaccagatg	caccttgggctt
415	*CG11881*	11881	SA2	0	720	atcggaccataa	ttgttctcggct
479	*CG16969*	16969	SA2	2	480	atgatccatccg	cggtctccttga
746	*Grip75*	6176	SA2	1	770	agatcgctgtga	tcgacttcatcc
66	*asp*	6875	SA3	0	960	ctgcgatctttc	agatgattacgc
176	*CG17286*	17286	SA3	3	900	ttgtcaaccacc	tcgtgtgggtca
179	*Sas-4*	10061	SA3	2	850	agcggcagtcca	catcagctactc
632	*NiPp1*	8980	SA3	0	500	tgatgataccaa	cggccaactttt
725	*cnn*	18370	SA3	0	870	acgaaacctcca	atgtgtaaccgg
740	*CG6937*	6937	SA3	3	650	tgaccaaggcca	atgcatcaacgt
32	*cdc2*	5363	SA4	0	700	tggacctcaaga	actaagcccgat
94	*Klp61F*	9191	SA4	1	1000	tggagtacacta	ccaccaactcct
139	*ncd*	7831	SA4	1	720	agcagccgccaa	agagcgggtgga
163	*Klp67A*	10923	SA4	0	700	ctcatccgggtc	acattctgtttc
14	*Incenp*	12165	SC1	0	810	gccgctaagctg	ctcgtcctccgt
37	*Caf1*	4236	SC1	0	780	cagaagctgat	atctctggctcc
222	*Borr*	4454	SC1	0	500	gtgtcctcgtcc	tgcagaaggagc
254	*ial*	6620	SC1	4	750	agcaccaggagc	tgctctccgtgc
83	*polo*	12306	SC2	1	920	attcgatgcacc	atactttctggg
524	*Myb*	9045	SC2	1	900	agtccgagtact	tgtgccagattg

87	*feo*	11207	CY1	0	730	agaaggagcgca	agcgactggtcg
137	*RacGAP50C*	13345	CY1	0	750	acgacttcctcg	ccaacctcggtc
284	*pav*	1258	CY1	1	550	gtcaagaactcc	tgacaaacaccg
286	*pbl*	8114	CY1	0	900	cagtagtagtgc	gtcgtacattcc
3	*scra*	2092	CY2	0	750	agccgctgcgtc	cctggctgaccg
204	*sti*	10522	CY2	3	1100	ccacggcataga	atccaccaagcc
785	*tsr*	4254	CY2	0	420	tgtaactgtgtc	agtttctcctcg
