# Supplementary material for: Identification of Drosophila Mitotic Genes by Combining Co-Expression Analysis and RNA Interference
Source: PLoS Genet. 2008 Jul 18;4(7):e1000126. doi: 10.1371/journal.pgen.1000126 (PMC2537813; doi:10.1371/journal.pgen.1000126)
Supplement: Table S5 — Characterization of the RNAi phenotypes elicited by the genes detected in the screen. (0.10 MB PDF) [file pgen.1000126.s021.pdf]

Supplementary Table 5. Characterization of the RNAi phenotypes elicited by the genes detected in the screen. Genes that have never been implicated in the maintenance of chromosome integrity and/or mitosis are marked in yellow. Other colors refer to the strength of the phenotype: pale blue, weak; blue, strong.The numbers in the CAB column are frequencies of chromosome aberrations per cell. The other numbers refer to the notes reported at the end of the table.

PHC, phenocluster: NM, no mitotic division; CA, chromosome aberrations; CS, chromosome segregation; CC, chromosome condensation; SA, spindle assembly; SC, spindle assembly and chromosome condensation; CY, cytokinesis.

Phenotypic traits: NCD no dividing cells; CAB chromosome aberrations; ACC abnormal chromosome condensation; PCS precocious sister chromatid separation; DCC defective chromosome congression at metaphase; HCC hypercontracted chromosomes; LFA, low relative frequency of anaphases; DCS defective chromosome segregation following sister chromatid separation; NSS no sister chromatid separation with scattered chromosomes; NSC no sister chromatid separation with chromosomes at the center of the cell; CBA chromatin bridges at anaphase; DDA, chromosome decondensation during anatelophase; LTS long anatelophase spindles; MPS monopolar spindles; DSP defective spindle poles (defective asters and/or broad poles); SSP short spindles; LMD, spindles with low MT density; OSD other spindle defects; BIN binucleated cells (cytokinesis failure); OMD, other mitotic defects.

Rank	Gene	CG	PHC	NDC	CAB	ACC	PCS	DCC	HCC	LFA	DCS	NSS	NSC	CBA	DDA	LTS	MPS	DSP	SSP	LMD	OSD	BIN	OMD
20	*RPA2*	*9273*	NM																				
40	*geminin*	*3183*	NM																				
108	*ran*	*1404*	NM																				
110	*Bx42*	*8264*	NM																				
196	*CycA*	*5940*	NM																				
264	*stg*	*1395*	NM																				
280	*RpII140*	*3180*	NM																				
350	*CycE*	*3938*	NM																				
851	*RpII215*	*1554*	NM																				
64	*Cdc2c*	*10498*	NM																				
69	*DebB*	*16792*	NM																				
169	*prp8*	*8877*	NM																				
188	*SF1*	*5836*	NM																				
4	*mus209*	*9193*	CA		1.16																		
5	*CG15220*	*15220*	CA		0.87																		
11	*RnrS*	*8975*	CA		1.33																		
12	*BEAF-32*	*10159*	CA		0.58																		
125	*noi*	*2925*	CA		1.12																		
134	*RfC40*	*14999*	CA		0.64																		
156	*DNAprim*	*5553*	CA		0.60																		
158	*Dp*	*4654*	CA		0.69																		
165	*CG12050*	*12050*	CA		0.67																		
199	*dnk*	*5452*	CA		1.50																		
216	*Su(var)2-10*	*8068*	CA		0.78																		
228	*CG13427*	*13427*	CA		0.60																		
260	*DDB1*	*7769*	CA		0.51																		
272	*RpA70*	*9633*	CA		>20																		
288	*CG10354*	*10354*	CA		0.62																		
299	*Orc5*	*7833*	CA		0.52																		
304	*DNApol*	*6349*	CA		0.72																		
376	*CG17383*	*17383*	CA		0.80																		
385	*okr*	*3736*	CA		0.80																		
396	*CG11906*	*11906*	CA		0.51																		
410	*CG2260*	*2260*	CA		0.52																		
417	*Mtor*	*8274*	CA		0.92																		
419	*woc*	*5965*	CA		0.52																		
435	*cul-4*	*8711*	CA		1.04																		
444	*Taf6*	*9348*	CA		0.62																		
464	*Dcp-1*	*5370*	CA		0.59																		
480	*His3.3B*	*8989*	CA		0.82																		
505	*CG8878*	*8878*	CA		0.64																		
540	*CG6197*	*6197*	CA		0.80																		
546	*l (2) NC136*	*8426*	CA		0.86																		
580	*Ts*	*3181*	CA		0.81																		
660	*CG1939*	*1939*	CA		0.53																		
667	*CG17446*	*17446*	CA		0.50																		
682	*ppan*	*5786*	CA		0.60																		
716	*c12.1*	*12135*	CA		0.95																		
735	*CG6480*	*6480*	CA		0.57																		
743	*CG7757*	*7757*	CA		0.74																		
778	*CG2685*	*2685*	CA		0.60																		
813	*CG6686*	*6686*	CA		0.68																		

823	SMC1	6057	CA		0.50																		
846	CG7003	7003	CA		0.50																		
850	CG32066	6487	CA		0.75																		
866	CG4785	4785	CA		0.73																		
937	CG6854	6854	CA		0.76																		
313	dup	8171	CS1																				1
8	Bub1	7838	CS2																				
15	Bub3	7581	CS2																				
450	dmt	8374	CS2																				
517	zw10	9900	CS2																				
687	rod	1569	CS2																				
93	U2af50	9998	CS3																				
208	CG9938	9938	CS3																				
213	CG3058	3058	CS3																				
229	CG6876	6876	CS3																				
261	CG3605	3605	CS3																				
287	l(1)G0237	1558	CS3																				
374	CG8902	8902	CS3																				
392	CG6015	6015	CS3																				
488	CG13298	13298	CS3																				
509	cid	13329	CS3																				
525	CG10754	10754	CS3																				2
566	CG8233	8233	CS3																				
573	CG5931	5931	CS3																				
354	CG10418	10418	CS3																				
358	SmD3	8427	CS3																				
394	CG18591	18591	CS3																				
656	CG8241	8241	CS3																				
81	CG16941	16941	CS3																				
102	Ote	5581	CS4																				
269	thr	5785	CS4																				3
391	CycB	3510	CS4																				
769	CG1420	1420	CS4																				
838	U2A'	1406	CS4																				
758	MBD-R2	10042	CS4		1.26																		
911	Sse	10583	CS4																				
648	CG2807	2807	CS4																				
262	U2af38	3582	CS4																				
88	fzy	4274	CS5																				
563	CG3221	3221	CS5																				
585	Pros26.4	5289	CS5																				
671	ida	10850	CS5																				
835	CG11419	11419	CS5																				
402	CG4266	4266	CS5																				
569	kin17	5649	CS5																				
775	Klp3A	8590	CS5																				
18	Mcm7	4978	CC1		1.24																		4
149	Mcm3	4206	CC1		0.60																		4
683	Cap	9802	CC1																				4
43	glu	11397	CC2																				
360	barr	10726	CC2																				
462	SMC2	10212	CC2																				
533	CAP-D2	1911	CC2																				
892	Cap-G	17054	CC2																				
231	Top2	10223	CC3																				
855	gwl	7719	CC3																				
466	Orc2	3041	CC3																				
369	Trip1	8882	SA1																				
557	CG1234	1234	SA1																				
19	Map60	1825	SA1																				
452	Int6	9677	SA1																	5			6
659	CG17293	17293	SA1																				
80	ik2	2615	SA1																	7			
305	Nnp-1	12396	SA1																				
693	eIF3-S10	9805	SA1																				
779	CG8636	8636	SA1																				
293	tho2	31671	SA1																				

399	*msps*	*5000*	SA1																		5		
950	*CG4865*	*4865*	SA1																		7		
256	*CG14781*	*14781*	SA1																		7		
306	*eIF-3p66*	*10161*	SA1																				
826	*mars*	*17064*	SA1																		7		
490	*Eb1*	*3265*	SA1																		7		
603	*CG8950*	*8950*	SA1																		5		
423	*BTub56b*	*9277*	SA1																		8		
263	*Nipped-A*	*2905*	SA2																				
415	*CG11881*	*11881*	SA2																				
479	*CG16969*	*16969*	SA2																				
746	*Grip75*	*6176*	SA2																		7		
193	*gTub23C*	*3157*	SA2																		7		
66	*asp*	*6875*	SA3																				
176	*CG17286*	*17286*	SA3																				
179	*Sas-4*	*10061*	SA3																				
632	*NiPp1*	*8980*	SA3																				
725	*cnn*	*18370*	SA3																				
740	*CG6937*	*6937*	SA3		0.58																		
139	*ncd*	*7831*	SA4																		9		
104	*Klp61F*	*9191*	SA4																		10		
163	*Klp67A*	*10923*	SA4																		7		
32	*cdc2*	*5363*	SA4																		11		
14	*Incenp*	*12165*	SC1																			12	
222	*Borr*	*4454*	SC1																		9	12	
254	*ial*	*6620*	SC1																			12	
37	*Cafl*	*4236*	SC1																			12	
83	*polo*	*12306*	SC2																			12	
524	*Myb*	*9045*	SC2																		7		
87	*feo*	*11207*	CY1																			12	
137	*RacGAP50C*	*13345*	CY1																			12	
284	*pav*	*1258*	CY1																			12	
286	*pbl*	*8114*	CY1																			12	
3	*scra*	*2092*	CY2																			13	
204	*sti*	*10522*	CY2																			13	
785	*tsr*	*4254*	CY2																			13	

(1) metaphase-like figures contain unreplicated chromosomes; (2) many anaphase-like figures but few telophase-like figures; (3) endoreduplicated metaphases (diplochromosomes); (4) lack of sister chromatid cohesion in the heterochromatic regions of the chromosomes; (5) long astral MTs in telophase; (6) drastic undercondensation of the heterochromatic regions of the chromosomes; (7) disorganized spindles; (8): split spindle poles; (9) multipolar spindles; (10) umbrella-like telophase spindles with all chromosomes at the astral pole; (11) centrosomes/asters detached from the spindle poles; (12) early cytokinesis defects: central spindle and contractile ring are both abnormal; (13) late cytokinesis defects: central spindle and contractile ring are normally assembled.
