# Supplementary material for: Identification of Drosophila Mitotic Genes by Combining Co-Expression Analysis and RNA Interference
Source: PLoS Genet. 2008 Jul 18;4(7):e1000126. doi: 10.1371/journal.pgen.1000126 (PMC2537813; doi:10.1371/journal.pgen.1000126)
Supplement: Table S6 — Previously known functions of Drosophila mitotic genes and their putative human orthologs. (0.23 MB PDF) [file pgen.1000126.s022.pdf]

Supplementary Table 6. Previously known functions of *Drosophila* mitotic genes and their putative human orthologs. PHC, phenocluster; NM, no mitotic division; CA, chromosome aberrations; CS, chromosome segregation; CC, Chromosome condensation; SA, spindle assembly; SC, spindle assembly and chromosome condensation; CY, cytokinesis. n.c., not conserved in humans

Gene name	PHC	Putative human ortholog	Known Functions
cdc2c *CG10498*	NM	*CDK2* (p33)	*cdc2c* encodes an ortholog of CDK2, a positive regulator of mammalian cell cycle progression. A *cdc2c* transgene does not rescue the *cdc2* (the ortholog of CDK1) mutant phenotype, indicating that the kinases encoded by these genes control different processes [1]. RNAi revealed that *cdc2c* is required for cell growth and viability [2].
DebB *CG16792*	NM	*snRNP-F*	The DebB protein is highly homologous to the human small ribonucleoprotein F (snRNP-F) involved in RNA splicing [3]. RNAi for *DebB* results in chromosome misalignment at metaphase [4].
prp8 *CG8877*	NM	*PRPF8*	*prp8* is an essential gene that encodes a protein highly homologous to human PRPF8, a central component of the splicesome. Mutations in PRPF8 cause Retinitis Pigmentosa [5]. RNAi for *prp8* results in cell death [6].
SF1 *CG5836*	NM	*SF1*	*SFI* encodes Splicing factor 1, a highly conserved Zn finger protein required for early stages of pre-mRNA splicing [3].
RPA2 *CG9273*	NM	*RPA2*	*RPA2* encodes the *Drosophila* ortholog of RPA2, the 32-kDa subunit of RPA, required for DNA recombination, repair and replication [7]
geminin *CG3183*	NM	*GMNN*	*geminin* is an essential gene that encodes a conserved protein. Both the *Drosophila* protein and its mammalian ortholog inhibit DNA replication [8,9]. Mutant *Drosophila* embryos undergoing cycle 16 show anaphase defects [9].
ran *CG1404*	NM	*RAN*	*ran* encodes the ortholog of the human RAN, a GTPase of the RAS superfamily required for multiple aspects of cell cycle progression and cell division [10]. RNAi for *ran* results in defective actin organization [11].
Bx42 *CG8264*	NM	*SKIP* (SKI-interacting protein)	The *Drosophila* Bx42 protein is associated with polytene chromosome puffs and is a cofactor involved in Notch signaling [12]. Human SKIP interacts with the SKI oncoprotein [13] and is required for cell division [14]
CycA *CG5940*	NM	*CCNA2*	*CyclinA (CycA)* encodes a highly conserved cyclin that activates Cdk, allowing cell cycle progression. Mutations in *Drosophila CycA* gene cause embryonic lethality and prevent cells to enter mitosis [15]. CCNA2, the human orthologue of Drosophila CycA, is also involved in mitotic regulation [16]
stg *CG1395*	NM	*CDC25*	*string (stg)* encodes an ortholg of CDC25, a highly conserved phosphatase that positively regulates the CDC2/CDK1 kinase. Mutations in *stg* cause G2 arrest during the 14th cell division cycle of *Drosophila* embryogenesis [17].
RpII140 *CG3180*	NM	*POLR2B*	*RpII140* is an essential gene that encodes the highly conserved 140kDa subunit of RNA polymerase II [18]. RNAi for *RpII140* results in chromosome misalignment [4].

CycE<br>*CG3938*	NM	*CCNE1*	*CyclinE (CycE)* is an essential gene that encodes a highly conserved cyclin required for progression through S phase [19]. Human Cyclin E (CCNE1) controls entry into S phase and is a prognostic marker for breast cancer [20].
RpII215<br>*CG1554*	NM	*POLR2A*	*RpII215* is an essential gene that encodes the highly conserved 215 kDa subunit of RNA polymerase II [21].
mus209<br>*CG9193*	CA	*PCNA*	*mus209* encodes the *Drosophila* ortholog of PCNA, a highly conserved DNA-damage inducible protein required for DNA replication and repair. *mus209* mutants are mutagen-sensitive but viable [22]. PCNA is an auxiliary factor of DNA polymerase delta [23,24]
CG15220	CA	n. c.	No information on gene function and protein structure
RnrS<br>*CG8975*	CA	*RRM2*	*RnrS* encodes the *Drosophila* ortholog of RRM2, a protein that catalyzes biosynthesis of deoxyribonucleotides from the corresponding ribonucleotides (FlyBase). RNAi for *RnrS* results in an S phase arrest [25]. RNAi for *RRM2* causes human cell death [14]
BEAF-32<br>*CG10159*	CA	n.c.	The BEAF-32 protein binds chromatin boundary elements and functions as a chromatin insulator factor [26,27]
noi<br>*CG2925*	CA	*SF3A3*	*noisette (noi)* encodes the *Drosophila* ortholog of SF3A3, a subunit of the essential heterotrimeric splicing factor SF3A [28,29]. Null mutations in *noi* cause embryonic lethality; hypomorphic alleles strongly affect germline cell proliferation [29]. RNAi for *noi* results in chromosome misalignment at metaphase [4], which is probably due to chromosome breakage (this study).
RfC40<br>*CG14999*	CA	*RFC2*	*RfC40* encodes the *Drosophila* ortholog of the human replication factor RFC2, which interacts with BRCA1 and PCNA [30]. Mutations in *RfC40* cause larval lethality with defects in cell proliferation. Mutant larval neuroblasts exhibit fragmented metaphase and anaphase chromosomes [31].
DNAprim<br>*CG 5553*	CA	*PRIM2A*	*DNAprim* is an essential gene that encodes the *Drosophila* ortholog of PRIM2A, the DNA primase large subunit [32].
Dp<br>*CG4654*	CA	*TFDP2*	Dp is a transcription factor that forms a heterodimer with E2F, which drives the G1-S transition [33]. RNAi for *Dp* results in reduced cell proliferation [11,34,35] and chromosome misalignment at metaphase [4]. The latter phenotype is probably due to chromosome breakage (this study).
CG12050	CA	*WDR75*	No information on gene function and protein structure
dnk<br>*CG 5452*	CA	*TK2*	*dnk* encodes a deoxynucleoside kinase that can phosphorylate all four deoxynucleosides [36]. Defects in the mitochondrial TK2 are responsible for the myopathic mitochondrial DNA depletion syndrome [37]
Su(var)2-10<br>*CG8068*	CA	*PIAS1*	*Su(var)2-10*, which encodes a member of the PIAS protein family, is a dominant suppressor of position effect variegation. Mutations in *Su(var)2-10* cause lethality and defects in chromosome structure [38]. Human PIAS1 (protein inhibitor of activated STAT1) functions as a SUMO ligase [39,40]
CG13427	CA		No information on gene function and protein structure
DDB1	CA	*DDB1*	*DDB1* encodes the *Drosophila* ortholog of the DNA

CG7769			Damage Binding protein 1. RNAi-mediated downregulation of *DDB1* causes larval lethality, melanotic tumors and a severe rough eye phenotype [41]. Human DDB1 forms an ubiquitin ligase complex with CUL4A and ROC1 and it is implicated in xeroderma pigmentosum group E [42,43]
RpA70<br>*CG9633*	CA	*RPA1*	*Rpa70* encodes the 70kDa subunit of the ssDNA-binding Replication Factor A [44]. Human RPA1 is required for DNA replication and repair [45]; it recruits the ATR-ATRIP complex to sites of DNA damage [7]. Mutations in *RPA1* cause chromosome instability and cancer in mice [46]. RNAi for *Rpa70* results in chromosome misalignment at metaphase [4], which is probably due to an extensive chromosome breakage (this study).
CG10354	CA	*XRN2*	The human homolog of the *CG10354*-encoded protein has 5'-3'exoribonuclease activity and is involved in transcription termination and homologous recombination [47,48]
Orc5<br>*CG 7833*	CA	*ORC5L*	*Orc5* is an essential gene that encodes the *Drosophila* ortholog of ORC5L, a component of the origin recognition complex required for DNA replication initiation [49]. Larval brain neuroblasts of *orc5* mutants show fragmented and overcondensed chromosomes and poorly organized spindles [50]
DNApol-α180<br>*CG6349*	CA	*POLA1*	*DNApol-α180* encodes the *Drosophila* alpha DNA polymerase (FlyBase).
CG17383	CA	n.c.	No information on gene function and protein structure
okr<br>*CG3736*	CA	*RAD54L*	*okra (okr)* encodes the *Drosophila* ortholog of RAD54L, a protein required for recombination and repair. *okr* mutants show defects in oogenesis; mutant larvae are highly sensitive to X rays and EMS [51]. Mutations in *RAD54L* have been associated with several primary cancers [52]
CG11906	CA	n.c.	No information on gene function and protein structure
CG2260	CA	*WDR46*	No information on gene and protein function
Mtor<br>*CG8274*	CA	*TRP*	Mutations in *Megator (Mtor)* cause lethality. During interphase, the Mtor protein is localized at the nuclear rim and in the nuclear space around the chromatin. During mitosis, the protein is associated with the putative spindle matrix [53]. The Mtor protein also binds the 93D puff upon heat shock, suggesting an involvement in RNA metabolism [54].
woc<br>*CG 5965*	CA	*ZMYM2*<br>*ZMYM3*<br>*ZMYM4*	*woc* is an essential gene encoding a putative transcription factor required to prevent telomeric fusions [55]. The Woc protein has 8 Zn fingers and shares homology with the human polypeptides ZMYM2, ZMYM3 and ZMYM4; ZMYM2 is implicated in a leukemia/lymphoma syndrome [56]
cul-4<br>*CG8711*	CA	*CUL4B*	*cul-4* encodes the *Drosophila* ortholog of CUL4B, a member of the cullin-RING ligase complex that mediates ubiquitination of proteins involved in DNA repair. Mutations in *cul-4* are cell lethal in somatic clones [57]; mutations in *CUL4B* cause an X-linked mental retardation syndrome [58]
Taf6	CA	*TAF6*	*Taf6* encodes a highly conserved subunit of the TFIID

CG9348			general transcription factor [59]. Mutations in *Taf6* reduce transcription of *bicoid*-dependent target genes and cause lethality [60].
Dcp-1<br>*CG5370*	CA	*CASP3*<br>*CASP6*<br>*CASP7*	*Death caspase-1* (*Dcp1*) is a *Drosophila* member of the caspase family of apoptotic proteases; mutations in *dcp1* cause larval lethality [61]. The Dcp1 protein shares homology with at least three human caspases.
His3.3B<br>*CG8989*	CA	*H3F3B*	*His3.3B* encodes a conserved *Drosophila* histone variant enriched in the active chromatin [62].
CG8878	CA	n.c.	*CG8878* encodes a putative serine/threonine kinase. No information on gene function [63]
CG6197	CA	*XAB2*	The protein product of *CG6197* shares homology with human XAB2 that binds XPA (xeroderma pigmentosum complementation group A). XAB2 is involved in transcription-coupled DNA repair [64]
l(2)NC136<br>*CG8426*	CA	*CNOT3*	*l(2)NC136* is identified by 2 lethal alleles. The product of the *Drosophila* gene is homologous to CNOT3, a global negative regulator of RNApol II transcription [65]
Ts<br>*CG3181*	CA	*TYMS*	The protein encoded by the *Drosophila Ts* gene is orthologous to the human thymdylate synthase (TYMS) required for DNA replication and repair. TYMS is a target for cancer chemotherapy; amplification of the *TYMS* gene causes resistance to 5-fluorouracil in metastatic colorectal cancer patients [66]
CG1939	CA	*DCAKD*	No information on the *Drosophila* gene function. The product of the *Drosophila* gene is homologous to human DCAKD, a protein that contains a dephospho-CoA kinase domain.
CG17446	CA	*CXXC1*	No information on the *Drosophila* gene function. The product of the *Drosophila* gene is homologous to human CXXC1, a component of the mammalian Set1 histone methyltransferase complex [67].
ppan<br>*CG5786*	CA	*PPAN*	Mutations in *peter pan* (*ppan)* cause lethality and reduced DNA replication [68]. Studies in human cells have identified PPAN as a putative tumor suppressor [69].
c12.1<br>*CG12135*	CA	*CWC15*	No reliable information on gene function and protein structure. RNAi for *c12.1* results in chromosome misalignment at metaphase [4], which is probably due to chromosome breakage (this study).
CG6480	CA	*FRG1*	No information about *Drosophila* gene function. Human *FRG1* is thought to be involved in pre-mRNA splicing of genes implicated in muscular distrophy [70]
CG7757	CA	*PRPF3*	*CG7757* encodes a conserved pre-mRNA splicing factor (U4/U6 snRNP) [3]
CG2685	CA	*WBP11*	No reliable information on gene function and protein structure.
CG6686	CA	*SART1*	No information about *Drosophila* gene function. Human SART1 is a putative splicing factor expressed in several malignant cell lines [71]. An RNAi screen showed that SART1 is required for mitotic division in human cells [14]
SMC1<br>*CG6057*	CA	*SMC1A*	*SMC1* encodes a conserved a component of the cohesin complex [72]. Mutations in human *SMC1A* are responsible for the Cornelia De Lange syndrome [73]

CG7003	CA	*MSH6*	The human ortholog of the *CG7003*-encoded protein is a component of the DNA mismatch recognition complex MSH2-MSH6 [74]. Mutations in the *MSH6* gene cause cancer susceptibility [75]
CG32066	CA	*FAM49B*	No information on gene function and protein structure.
CG4785	CA	*Q96HX5*	No information on gene function and protein structure.
CG6854	CA	*CTPS*	The protein product of *CG6854* is an ortholog of the highly conserved CTP synthase, an essential enzyme, required for the synthesis of nucleic acids and membrane phospholipids [76]
dup<br>*CG8171*	CS1	*CDT1*	*double parked* (*dup*) encodes the *Drosophila* ortholog of CDT1, a DNA replication licensing protein inhibited by geminin [77]. Mutations in *dup* cause lethality and affect DNA replication; mutant embryos exhibit mitotic cells with unreplicated chromosomes [78,79]. RNAi for *dup* results in abnormally compact chromatin and reduced cell viability [2,4].
bub1/bubR1<br>*CG7838*	CS2	*BUBR1*	*bubR1* encodes the conserved BubR1 kinase involved in the spindle checkpoint machinery. Mutations in *bubR1* cause larval lethality; mitotic divisions in larval brain display precocious sister chromatid separation and chromosome missegregation during anaphase [80]. RNAi for *bubR1* results in defects in chromosome alignment and segregation leading to aneuploidy [4,81]. Mutations in human *BUBR1* (*BUB1B*) cause cancer susceptibility (reviewed by [82])
Bub3<br>*CG7581*	CS2	*BUB3*	*Bub3* encodes a conserved component of the spindle checkpoint machinery (reviewed by [82]). RNAi for *bub3* causes defects in chromosome alignment, precocious sister chromatid separation and chromosome missegregation during anaphase, leading to aneuploidy [4,83].
dmt<br>*CG8374*	CS2	n.c.	*dalmatian* (*dmt*) is required for the development of *Drosophila* nervous system [84]. RNAi for *dmt* results in chromosome misalignment [4]
mit(1)15<br>*l(1)zw10*<br>*CG9900*	CS2	*ZW10*	The Zw10 protein is a conserved component of the Zw10-Rod-Zwilch complex involved in the spindle checkpoint (reviewed by [82,85]). Mutations in *l(1)zw10* cause lethality, precocious sister chromatid separation and chromosome missegregation at anaphase, resulting in frequent aneuploid cells. RNAi for *zw10* results in defective cell morphology [11]. Mutations in human *ZW10* have been associated with colorectal cancers with chromosome instability [86].
rod<br>*CG1569*	CS2	*KNTC1*<br>*Rod*	The Rod protein is a conserved component of the Zw10-Rod-Zwilch complex involved in the spindle checkpoint (reviewed by [82,85]). Mutations in *rough deal* (*rod*) cause lethality and chromosome missegregation, resulting in frequent aneuploid cells [87]. Mutations in human *Rod* have been associated with colorectal cancers with chromosome instability [86].
U2af50<br>*CG9998*	CS3	*U2AF2*<br>*U2AF65*	*U2af50* is an essential gene that encodes a highly conserved component of the U2AF heterodimer required for pre-mRNA splicing [88,89]. RNAi for *U2af50* causes

			chromosome misalignment at metaphase [4]
CG3058	CS3	*TXN4A*	*CG3058* encodes the highly conserved spliceosomal U5 snRNP-specific 15 kDa protein [3]. RNAi for *CG3058* results in chromosome misalignment at metaphase [4]. Dim1, the fission yeast ortholog of the CG3058 product is required for chromosome segregation during mitosis [90].
CG6876	CS3	*PRPF31*	*CG6876* encodes the highly conserved spliceosomal U4/U6 snRNP 61 kDa protein [3]. Mutations in human *PRPF31* are associated with autosomal dominant retinitis pigmentosa [91].
CG3605	CS3	*SF3B2*	*CG3605* encodes the highly conserved splicing factor 3B subunit 2 (SAP 145) [3].
l(1)G0237<br>*CG1558*	CS3	n.c.	*l(1)G023* is an essential gene that encodes a component of the Mis12 kinetochore-associated complex. The product of *l(1)G023*, also called Kmn1 or DmNsl1R, shares homology with the yeast Nsl1p kinetochore protein. RNAi for *l(1)G023* causes defects in chromosome alignment and segregation [92,93].
CG9938	CS3	*KNTC2*<br>*HEC1*	*CG9938* encodes a component of the conserved Hec1/Ndc80 kinetochore-associated complex. RNAi for *CG9938* causes defects in chromosome alignment and segregation [4,92,93]. Hec1 regulates kinetochore microtubule plus-end dynamics [94].
CG8902	CS3	*NUF2*	*CG8902* is an essential gene that encodes a component of the conserved Hec1/Ndc80 kinetochore-associated complex. RNAi for *CG8902* causes defects in chromosome alignment and segregation [4,92,93]. Nuf2 is a component of the kinetochore outer plate essential for microtubule attachment [95].
CG6015	CS3	*PRP17*<br>*CDC40*	*CG6015* encodes the highly conserved splicing factor PRP17 [3].
CG13298	CS3	*PM14*<br>*SF3B14*	*CG13298* encodes the highly conserved 14kDa subunit of the SF3B splicing factor complex [96]. RNAi for *CG13298* results in chromosome misalignment at metaphase [4].
CG8233	CS3	*K1310*	No information on gene function and protein structure.
CG5931	CS3	*U520*	*CG5931* encodes a highly conserved U5 snRNP-specific RNA helicase involved in pre-mRNA splicing [97].
cid<br>*CG13329*	CS3	*CENP-A*	*cid* is an essential gene that encodes the centromere-specific histone H3 variant homologous to human CENP-A [98,99]. RNAi for *cid* causes defects in chromosome alignment and segregation [4,93,99].
CG10754	CS3	*SF3A2*<br>*SAP62*	*CG10754* encodes a subunit of the conserved splicing factor SF3A [3]. RNAi for *CG1075* results in chromosome misalignment at metaphase [4].
SmD3<br>*CG8427*	CS3	*SMD3*	*CG8427* encodes an ortholog of the conserved small ribonucleoprotein SmD3 (snRNP core protein D3), which is part of a complex involved in pre-mRNA splicing [3]. Mutations in *CG8427* cause lethality during embryonic, larval or pupal stages. Mutants exhibit neoplastic abnormalities in imaginal discs and brains [100,101]. RNAi for *CG8427* causes chromosome misalignment at metaphase [4].

CG10418	CS3	*LSM2*	*CG10418* encodes a highly conserved ortholog of U6 snRNA-associated Sm-like protein LSm2, a component of the heptameric Sm complex required for pre-mRNA splicing [3].
CG18591	CS3	*SNRPEL1*	*CG18591* encodes a highly conserved ortholog of the snRNP E-like protein 1, a component of the heptameric Sm complex required for pre-mRNA splicing [3].
CG8241	CS3	*DHX8*	*CG8241* encodes a highly conserved RNA helicase involved in pre-mRNA splicing [3]. RNAi for human *DHX8* causes mitotic defects [14].
CG16941	CS3	*SF3A1*	*CG16941* encodes a subunit of the splicing factor SF3A [3]. RNAi for *CG16941* reduces cell viability and causes chromosome misalignment at metaphase[2,4].
Ote<br>*CG5581*	CS4	n.c.	*Otefin (Ote)* encodes a nuclear envelope-associated protein that interacts with lamin [102]
thr<br>*CG5785*	*CS4*	n.c.	Mutations in *three rows (thr)* cause death during embryogenesis. In *thr* mutants, chromosome congression is normal but sister chromatid separation does not occur. The arrested metaphase figures exhibit extended spindles [103,104]. RNAi for *thr* results in chromosome misalignment at metaphase [4]. The Thr protein interacts with *Drosophila* securin (Pimples) and is cleaved by separase after the metaphase-to-anaphase transition [105].
CycB<br>*CG3510*	CS4	*CCNB1*	Null mutats in *CycB* gene are viable but *CycB CycB3* double mutants are early lethal, suggesting that the two cyclins are redundant. These double mutants often exhibit chromosomes with unseparated sister chromatids at the equator of the dividing cells [106]. RNAi for *CycB* reduces cell growth and viability [2].
CG1420	CS4	*SLU7*	*CG16941* encodes the conserved SLU7 splicing factor [3]. RNAi for *CG1420* results in chromosome misalignment at metaphase [4]
U2A<br>*CG1406*	CS4	*Q9NU36*<br>*SNRPA1*	*U2A* is an essential gene that encodes the conserved the U2A' snRNP protein required for pre-mRNA splicing [107]. RNAi for *U2A* results in chromosome misalignment at metaphase [4].
MBD-R2<br>*CG10042*	CS4	*PHF20*	*MBD-R2* gene encodes a conserved putative transcription factor, which contains a methyl-CpG-binding domain [108]
Sse<br>*CG10583*	CS4	*ESPL1*<br>*Separin*	*Sse* encodes a divergent homolog of Separase/ Separin [109]. Separase serves conserved function from yeast to humans; it mediates proteolytic cleavage of a cohesin subunit, allowing anaphase onset [110,111].
CG2807	CS4	*SF3B1*<br>*SAP155*	*CG2807* encodes a highly conserved subunit of splicing factor 3B [3]. RNAi for *CG2807* reduces cell growth and viability [2].
U2af38<br>*CG3582*	CS4	*U2AF1*	*U2af38* is an essential gene that encodes a highly conserved component of the U2AF heterodimer required for pre-mRNA splicing [112]. RNAi for *U2af38* results in chromosome misalignment at metaphase [4].
fzy<br>*CG4274*	CS5	*CDC20*	*fizzy* (*fzy*) encodes the highly conserved Cdc20 regulator of APC/C activity. Mutations in *fzy* cause cells in mitosis to arrest at metaphase [113]. RNAi for *fzy* causes metaphase arrest and reduced cell viability [2,11].

Pros26.4 *CG5289*	CS5	*PSMC1* *PRS4*	*Pros 26.4* encodes the highly conserved Proteasome 26S subunit [114]. RNAi for *Pros 26.4* results in reduced cell viability and defective spindle assembly [2,4].
ida *CG10850*	CS5	*ANAPC5* *APC5*	*ida* is an essential gene that encodes a protein homologous to the APC5 subunit of the APC/C. *ida* mutant brains have a high mitotic index but cells are not arrested in metaphase; chromosomes congression and segregation are compromised leading to aneuploidy [115].
CG11419	CS5	*ANAPC10* *APC10*	*CG11419* encodes the highly conserved APC10 subunit of the APC/C [116].
CG3221 *dgt3*	CS5	n.c.	The gene product has a low degree of conservation; there is no information on protein structure and function. RNAi for *CG3221* results in diminished gamma tubulin at the centrosomes and long spindles [4].
CG4266	CS5	*SFR15*	*CG4266* encodes a putative RNA binding protein with domains highly conserved in vertebrates [117].
kin17 *CG5649*	CS5	*KIN17*	*kin17* encodes a conserved protein. Human Kin17 is a stress-activated protein that accumulates at sites of active DNA replication [118].
Klp3A *CG8590*	CS5	*KIF4*	*Klp3A*, an allele of *mei-352*, encodes a conserved kinesin-like protein required for both male and female meiosis but not essential for viability [119,120]. RNAi for *Klp3A* results in defective spindle assembly and chromosome misalignment at metaphase [4,121,122].
Mcm7 *CG4978*	CC1	*MCM7*	*Mcm7* encodes a highly conserved component of the MCM helicase complex involved in DNA replication [123]. Human MCM7 interacts with the ATM and ATR checkpoint kinases [124,125].
Mcm3 *CG4206*	CC1	*MCM3*	*Mcm3* encodes a highly conserved component of the MCM helicase complex involved in DNA replication [123]. The murine MCM3 protein accumulates transiently in the heterochromatic regions of the chromosomes [123].
Cap *CG9802*	CC1	*SMC3*	*Cap* encodes the Smc3 conserved component of the cohesin complex required for sister chromatids cohesion after DNA replication [126]. Mutations in human *SMC3* cause a mild variant of the Cornelia de Lange syndrome [127].
glu *CG11397*	CC2	*SMC4*	*gluon (glu)* encodes the highly conserved SMC4 subunit of the condensin complex. Mutations in *glu* cause defective chromosome condensation and chromatin bridges at anaphase [128]. RNAi for *glu* results in abnormal chromosome condensation and chromosome misalignment at metaphase [4,129,130].
barr *CG10726*	CC2	*CND2* *XCAP-H*	*barren (barr)* encodes the conserved XCAP-H, non-SMC subunit, of the condensin complex. Mutations in *barr* cause embryonic lethality and chromatin bridges at anaphase [131]. RNAi for *barr* results in abnormal chromosome condensation and chromosome misalignment at metaphase [4,130,132,133].
SMC2 *CG10212*	CC2	*SMC2*	*SMC2* encodes the conserved SMC2 subunit of the condensin complex. Mutations in *SMC2* cause lethality; the chromosomes of mutant cells are fuzzy with no sister chromatid resolution, resulting in chromatin bridges at

			anatelophase [134]. RNAi for *SMC2* results in chromosome misalignment at metaphase [4].
CAP-D2<br>*CG1911*	CC2	*NCAPD2*	*CAPD2* encodes the conserved CAPD2, non-SMC subunit, of the condensin complex. RNAi for *CAPD2* results in abnormal chromosome condensation, chromosome misalignment at metaphase and chromatin bridges at anatelophase [4,135].
Cap-G<br>*CG17054*	CC2	*CND3*<br>*NCAPG*	*Cap-G* encodes the conserved CAP-G, non-SMC subunit, of the condensin complex. Mutations in *Cap-G* cause lethality and affect chromosome condensation particularly during prophase and prometaphase; sister chromatid resolution is defective, resulting in chromatin bridges at anatelophase [136]. RNAi for *Cap-G* results in chromosome misalignment at metaphase, chromatin bridges at anaphase and mild defects in cytokinesis [4,137].
Top2<br>*CG10223*	CC3	*TOP2B*	*Top2* encodes the conserved topoisomerase II enzyme. RNAi for *Top2* causes defects in chromosome condensation and sister chromatid separation at anaphase [4,138].
gwl<br>*CG7719*	CC3	*MASTL*	*gwl* encodes a conserved Ser/Thr kinase. Mutations in *gwl* cause lethality and affect mitotic chromosome condensation [129]. RNAi for *gwl* results in abnormal chromosome condensation and segregation, and defects in spindle morphology [81,129].
Orc2<br>*CG3041*	CC3	*ORC2L*	*Orc2* encodes a conserved subunit of the ORC complex. Mutations in *Orc2* cause lethality; mutant cells display irregularly condensed chromosomes, which fail to align properly at metaphase [50,139]
Trip1<br>*CG8882*	SA1	*IF32*<br>*eIF3 p36*	*Trip1* encodes the highly conserved subunit 2 of the eukaryotic translation initiation factor 3 (eIF3 p36) [117,140]
CG1234	SA1	*NOC3L*	*CG1234* encodes the NOC3 conserved component of the NOC complex required for ribosome maturation and transport [141]. Human NOC3L is a spindle-associated, phosphopeptide-containing protein [142].
Map60<br>*CG1825*	SA1	n.c.	*Map60* encodes the microtubule-associated centrosomal protein CP60 [143]. RNAi for *Map60* did not result in mitotic defects [144].
Int6<br>*CG9677*	SA1	*EIF3S6*	*Int6* is an essential *Drosophila* gene [145] that encodes the highly conserved P48 component of eukaryotic translation initiation factor 3 [146]. *Int6* was first identified as a frequent integration site of the MMTV virus in mouse mammary tumors [147]. *Int6/EIF3S6* silencing leads to defects in spindle formation, chromosome segregation and cytokinesis in human cells [148].
CG17293	SA1	*LOC730735*	*CG17293* encodes a highly conserved protein of unknown function (FlyBase).
ik2<br>*CG2615*	SA1	*TBK1*	*ik2* is an essential gene that encodes a conserved kinase. Germ line clone analysis showed that *ik2* mutant oocytes are defective in *oskar* and *gurken* mRNA localization [149]. RNAi for *ik2* results in spindle abnormalities and multiple mitotic defects [81].
Nnp-1	SA1	*RRP1*	*Nnp-1* is an essential gene [145] that encodes a conserved

CG12396			protein product probably required for generation of 28S rRNA [150]. RNAi for *Nnp-1* results in short bipolar and monopolar spindles with low microtubule density [4]. RRP1 has been recently implicated in breast cancer progression [151].
eIF3-S10<br>*CG9805*	SA1	*IF3A*	*eIf3-S10* encodes the highly conserved subunit 10 of the eukaryotic translation initiation factor 3 [117]. RNAi for *eIf3-S10* results in defective cell growth and viability [2] and produces short bipolar and monopolar spindles with low microtubule density [4]. RNAi for the human *IF3A* gene causes spindle abnormalities [14].
CG8636	SA1	*IF34*	*CG8636* encodes the highly conserved subunit 4 of the eukaryotic translation initiation factor 3 (eIF3S4) [117]. RNAi for *CG8636* results in short bipolar and monopolar spindles with low microtubule density [4].
tho2<br>*CG31671*	SA1	*THOC2*	*tho2* encodes a conserved subunit of the THO complex that couples transcription with mRNA export [152,153].
msps<br>*CG5000*	SA1	*CKAP5*<br>*XMAP215*	*minispindles* (*msps*) is an essential gene that encodes a conserved product required for spindle organization. *msps* mutants exhibit one or more additional minispindles in diploid cells [154]. RNAi for *msps* results in short bipolar and monopolar spindles [4,155]. The human CKAP5/XMAP215 protein is also essential for spindle pole organization [156].
CG4865<br>*dgt4*	SA1	n.c.	*CG4865* encodes a protein of unknown function. RNAi for *CG4865* results in diminished gamma-tubulin at the spindle poles and long spindles [4].
CG14781<br>*ssp1*	SA1	n.c.	*CG14781* encodes a protein of unknown function. RNAi for *CG14781* results in short bipolar and monopolar spindles [4].
eIF-3p66<br>*CG10161*	SA1	*IF37*<br>*eIF3 p66*	*eIf3-p66* encodes the highly conserved subunit 7 of the eukaryotic translation initiation factor 3 (eIF3S7 or eIF3 p66) [117].
mars<br>*CG17064*	SA1	*HURP*	*mars* encodes the *Drosophila* ortholog of the human hetapoma up-regulated protein (HURP). HURP is a Ran-interacting factor that binds and stabilizes spindle microtubules [157-159]. Overexpression of *mars* results in chromosome misalignment and mispositioned centrosomes [160].
Eb1<br>*CG3265*	SA1	*MARE1*<br>*EB1*	*Eb1* is an essential gene that encodes a conserved protein that localizes to the plus ends of growing microtubules (FlyBase). RNAi for Eb1 results in short and disorganized spindles [4,155,161].
CG8950	SA1	*TF3C3*	*CG8950* encodes the conserved gamma subunit of transcription factor 3C required for synthesis of most polIII products, including tRNAs and 5SrRNA. TF3C3 is overexpressed in tumors [162].
βTub56D<br>*CG9277*	SA1	*TUBB2C*	*βtub56D* is an essential gene that encodes a beta-tubulin isoform expressed throughout development and in adult tissues [145]. RNAi for *Btub56D* results in short bipolar and monopolar spindles [4]
Nipped-A<br>*CG2905*	SA2	*TRRAP*	*Nipped*-A is an essential gene mapping to the second chromosome heterochromatin [163]; it encodes the

			Drosophila ortholog of the TRRAP pseudokinase involved in chromatin remodelling, gene expression and DNA repair [164]. Null mutations in mouse *Trrap* gene cause defects in spindle formation, chromosome segregation and cytokinesis [165]. RNAi for *Nipped-A* results in cytokinesis failure [137].
CG11881<br>*dgt6*	SA2	*FAM29A*	*CG11881* encodes a conserved protein of unknown function. RNAi for *CG11881* results in diminished gamma-tubulin at the spindle poles and long spindles [4]
CG16969<br>*dgt2*	SA2	n.c.	No information on protein structure. RNAi for *CG16969* results in diminished gamma-tubulin at the spindle poles and long spindles [4].
Grip75<br>*CG6176*	SA2	*TUBGCP4*	*Grip75* encodes a conserved component of the gamma-tubulin ring complex required for microtubule nucleation [166]. Null mutants in *Grip75* are viable but female sterile; embryos laid from *Grip75* mutant mothers do not show spindles or dividing nuclei [167]. RNAi for *Grip75* results in diminished gamma-tubulin at the spindle poles and long monopolar and bipolar spindles with low MT density [4,168].
γTub23C<br>*CG3157*	SA2	*TUBG1*	*γtub23C* is an essential gene that encodes the major gamma-tubulin isoform, a conserved component of the gamma-tubulin ring complex required for microtubule nucleation [166]. Mutants in *γtub23C* exhibit morphologically abnormal spindles that lack astral microtubules [169]. RNAi for *γtub23C* results in monopolar spindles and anastral bipolar spindles with poorly focused poles [4,170].
asp<br>*CG6875*	SA3	*ASPM*	*abnormal spindle (asp)* is an essential gene that encodes a conserved protein required for spindle pole focusing ([171-173]). RNAi for *asp* results in unfocused spindle poles with frequent pole detachment [4]. Mutations in human *ASPM* cause primary microcephaly type 5 [174]
CG17286<br>*Dspd-2*	SA3	*CEP192*	*CG17286* encodes a protein homologous to the human 192kDa centrosomal protein [175]. *Dspd-2* is required for astral MT nucleation and PCM recruitment at the sperm centriole [176,177]. RNAi for *CG17286* results in diminished gamma-tubulin at the spindle poles [4].
Sas-4<br>*CG10061*	SA3	*CENP-J*	*Sas-4* encodes a protein required for centriole replication. *Sas-4* mutant animals loose centrioles during embryogenesis and, by third instar larval stage, lack centrioles, centrosomes and astral microtubules [178]. RNAi for *Sas-4* results in anastral and monastral bipolar spindles [4]. Mutations in *CENPJ*, the human ortholog of *Sas-4,* cause primary microcephaly type 6 [179].
NiPp1<br>*CG8980*	SA3	*PP1R8*<br>*NIPP-1*	*Nuclear inhibitor of Protein phosphatase 1* (*NiPp1*) encodes a conserved inhibitor of the nuclear protein phosphatase-1 (PP1) [180].
cnn<br>*CG18370*	SA3	*CK5P2*<br>*CDK5RAP2*	*centrosomin* (*cnn*) encodes a conserved component of the centrosome [175]. *cnn* mutants are viable but sterile in both sexes; mutant cells do not exhibit astral microtubules [181]. RNAi for *cnn* results in anastral spindles with unfocused poles [4,170,181]. Mutations in human *CDK5RAP2* cause

			primary microcephaly type 3 (MCPH3) [179]
CG6937	SA3	*MK67I*	*CG6937* encodes a putative RNA binding protein homologous to MK67I (nucleolar protein interacting with the FHA domain of pKI-67), a polypeptide that interacts with pKI-67 during mitosis [117,182]. pKi-67 is a cell proliferation marker that localizes around the chromosomes [183].
ncd<br>*CG7831*	SA4	*KIFC1*	*non-claret disjunctional* (*ncd*) is an essential gene that encodes a conserved kinesin with minus-end-directed microtubule motor activity. Mutations in *ncd* affect meiotic and mitotic chromosome segregation and disrupt meiotic spindle formation in females [184]. RNAi for *ncd* results in disorganized and multipolar spindles [4,121].
Klp61F<br>*CG9191*	SA4	*KIF11*<br>*Eg5*	*Klp61F* is an essential gene that encodes a conserved kinesin-like protein with plus-end-directed microtubule motor activity. *Klp61F* mutant brain cells are severely defective in centrosome separation and exhibit monopolar spindles[185]. RNAi for *Klp61F* causes frequent monopolar spindles blocking mitotic progression [4,11,121]. Downregulation of human Eg5 prevents centrosome migration causing mitotic arrest with monoastral spindles [186].
Klp67A<br>*CG10923*	SA4	*KI18A*<br>*KIF18A*	*Klp67A* encodes a conserved plus-end directed kinesin-like protein with microtubule depolymerizing activity [187-189]. RNAi for *Klp67A* causes monopolar spindles, an increase in spindle length and chromosome misalignment at metaphase [4,121,189]. RNAi for human *KIF18A* induces aberrantly long mitotic spindles and defective chromosome alignment and segregation [188].
cdc2<br>*CG5363*	SA4	*CDC2*<br>*CDK1*	*cdc2* is an essential gene that encodes a conserved cyclin-dependent protein kinase. Mutations in *cdc2* cause lethality at the larval/pupal transition and disrupt imaginal disc cell proliferation [1]. RNAi for cdc2 results in reduced cell growth and viability, defective cell shape and delayed progression through mitosis [2,11,81]. CDC2/CDK1 is the catalytic subunit of a kinase complex that controls multiple aspects of cell cycle progression, including the DNA damage response [190].
Incenp<br>*CG12165*	SC1	*INCENP*	*Incenp* encodes a conserved component of the chromosome passenger complex (CPC), which also includes the Aurora B kinase, Borealin and Survivin. The CPC is required for multiple aspects of cell division including chromosome condensation, chromosome segregation and cytokinesis [191]. Hypomorphic *Incenp* mutant alleles cause premature sister chromatid separation and defective chromosome segregation during male meiosis [192]. RNAi for *Incenp* causes defects in chromosome and spindle morphology, chromosome alignment and segregation, and cytokinesis [4,193].
Borr<br>*CG4454*	SC1	*CDCA8*<br>*BOREALIN*<br>*DASRA B*	*Borealin-related* (*Borr*) is an essential gene that encodes a conserved component of the chromosome passenger complex (CPC), which also includes the Aurora B kinase, Incenp and Survivin [191,194]. The CPC is required for

			multiple aspects of cell division including chromosome condensation, chromosome segregation and cytokinesis [191]. RNAi for *Borr* causes defects in chromosome and spindle morphology, chromosome alignment at metaphase, and cytokinesis [4,195].
ial<br>*CG6620*	SC1	*AURKB*<br>*Aurora-B*	*IplI-aurora-like kinase* (*ial*) encodes a conserved component of the chromosome passenger complex (CPC), which also includes Incenp, Survivin and Borealin. The CPC is required for multiple aspects of cell division including chromosome condensation, chromosome segregation and cytokinesis [191]. RNAi for *ial* causes defects in chromosome condensation and alignment, chromosome segregation, spindle organization and cytokinesis [4,81,137,193,195,196].
Caf1<br>*CG4236*	SC1	*RBBP4*<br>*CAF-1 subunit C*	*Chromatin assembly factor 1 subunit* (*Caf1*) encodes a conserved histone-binding protein, which is a component of several complexes that regulate chromatin metabolism [197]. RNAi for *Caf1* results in defective cytokinesis [137].
polo<br>*CG12306*	SC2	*PLK1*	*polo* is an essential gene that encodes a conserved kinase required for multiple aspects of cell division, including chromosome condensation, spindle assembly, chromosome alignment and segregation, and cytokinesis [198]. RNAi for *polo* causes spindle and centrosome abnormalities, and delayed progression through mitosis [4,11,81]. Human PLK1 has been implicated in the regulation of mitotic entry, spindle formation and cytokinesis, and has been proposed as a target in cancer therapies [199].
Myb<br>*CG9045*	SC2	*MYB*	*Myb* is an essential gene that encodes a protein homologous to the product of the *c-Myb* oncogene. Mutations in *Myb* cause multiple mitotic defects, including abnormally condensed chromosomes, supernumerary centrosomes, disorganized spindles and abnormal chromosome segregation, resulting in aneuploid and polyploid cells [200,201]. RNAi for *Myb* results in long monastral bipolar spindles with low microtubule density, which often exhibit pole detachment [4].
feo<br>*CG11207*	CY1	*PRC1*	*fascetto (feo)* is an essential gene that encodes a conserved microtubule binding protein [202]. Mutations in *feo* affect both central spindle and contractile ring formation and disrupt cytokinesis [202]. RNAi for *feo* results in strong cytokinesis defects [137,195,202]. PRC1, the human ortholog of Feo, is required for cytokinesis [203].
tum<br>*RacGAP50C*<br>*CG13345*	CY1	*RACGAP1*	*RacGAP50C* is an essential gene that encodes a Rho GAP that forms the conserved centralspindlin complex with the Pav kinesin-like protein [204]. RNAi for *RacGAP50C* disrupts both central spindle and contractile ring formation leading to cytokinesis failures. [137,195,205,206]. The mammalian ortholog of RacGAP50C is required for cytokinesis [204].
pav<br>*CG1258*	CY1	*KIF23*	*pavarotti (pav)* is an essential gene that encodes a kinesin-like protein that forms the conserved centralspindlin complex with Rho GAP [204,207]. Mutations in *pav* affect central spindle formation and inhibit cytokinesis [207].

			RNAi for *pav* disrupts both central spindle and contractile ring formation leading to cytokinesis failures. [11,121,137,195,205]. The mammalian ortholog of Pav is required for cytokinesis [204].
pbl *CG8114*	CY1	*ECT2*	*pebble (pbl)* is an essential gene that encodes a conserved Rho GEF that interacts with the centralspindlin complex [204,208]. Mutations in *pbl* disrupt cytokinesis [209,210]. RNAi for *pbl* inhibits both central spindle and contractile ring formation leading to cytokinesis failures [11,137,195,205].
scra *CG2092*	CY2	*ANLN*	*scraps (scra)* is an essential gene that encodes anillin, a conserved actin- and myosin-binding protein [211,212]. RNAi for *scra* causes defects in late stages of cytokinesis. [11,137,195,205,206]. Human anillin is required for cytokinesis [213].
sti *citron kinase* *CG10522*	CY2	*STPK21*	*sticky (sti)* is an essential gene that encodes the conserved citron kinase. Mutations in *sti* disrupt the final steps of cytokinesis [214-216]). RNAi for *sti* causes strong defects in cytokinesis [11,81,137,195,206,214,216]. The mammalian homolog of Sti is required for cytokinesis [217]
tsr *CG4254*	CY2	*DSTN* *ADF*	*twinstar (tsr)* is an essential gene that encodes the conserved actin-severing protein cofilin [218]. Mutations in *tsr* inhibit contractile ring disassembly and disrupt the final stages of cytokinesis [218]. RNAi for *tsr* causes late defects in cytokinesis [11,137,195,205,206].

1. Stern B, Ried G, Clegg NJ, Grigliatti TA, Lehner CF (1993) Genetic analysis of the Drosophila cdc2 homolog. Development 117: 219-232.
2. Boutros M, Kiger AA, Armknecht S, Kerr K, Hild M, et al. (2004) Genome-wide RNAi analysis of growth and viability in Drosophila cells. Science 303: 832-835.
3. Mount SM, Salz HK (2000) Pre-messenger RNA processing factors in the Drosophila genome. J Cell Biol 150: F37-44.
4. Goshima G, Wollman R, Goodwin SS, Zhang N, Scholey JM, et al. (2007) Genes required for mitotic spindle assembly in Drosophila S2 cells. Science 316: 417-421.
5. McKie AB, McHale JC, Keen TJ, Tarttelin EE, Goliath R, et al. (2001) Mutations in the pre-mRNA splicing factor gene PRPC8 in autosomal dominant retinitis pigmentosa (RP13). Hum Mol Genet 10: 1555-1562.
6. Ramet M, Manfruelli P, Pearson A, Mathey-Prevot B, Ezekowitz RA (2002) Functional genomic analysis of phagocytosis and identification of a Drosophila receptor for E. coli. Nature 416: 644-648.
7. Zou L, Elledge SJ (2003) Sensing DNA damage through ATRIP recognition of RPA-ssDNA complexes. Science 300: 1542-1548.
8. McGarry TJ, Kirschner MW (1998) Geminin, an inhibitor of DNA replication, is degraded during mitosis. Cell 93: 1043-1053.
9. Quinn LM, Herr A, McGarry TJ, Richardson H (2001) The Drosophila Geminin homolog: roles for Geminin in limiting DNA replication, in anaphase and in neurogenesis. Genes Dev 15: 2741-2754.
10. Sanderson HS, Clarke PR (2006) Cell biology: Ran, mitosis and the cancer connection. Curr Biol 16: R466-468.
11. Kiger AA, Baum B, Jones S, Jones MR, Coulson A, et al. (2003) A functional genomic analysis of cell morphology using RNA interference. J Biol 2: 27.

12. Negeri D, Eggert H, Gienapp R, Saumweber H (2002) Inducible RNA interference uncovers the Drosophila protein Bx42 as an essential nuclear cofactor involved in Notch signal transduction. Mech Dev 117: 151-162.
13. Dahl R, Wani B, Hayman MJ (1998) The Ski oncoprotein interacts with Skip, the human homolog of Drosophila Bx42. Oncogene 16: 1579-1586.
14. Kittler R, Putz G, Pelletier L, Poser I, Heninger AK, et al. (2004) An endoribonuclease-prepared siRNA screen in human cells identifies genes essential for cell division. Nature 432: 1036-1040.
15. Lehner CF, O'Farrell PH (1989) Expression and function of Drosophila cyclin A during embryonic cell cycle progression. Cell 56: 957-968.
16. Girard F, Strausfeld U, Fernandez A, Lamb NJ (1991) Cyclin A is required for the onset of DNA replication in mammalian fibroblasts. Cell 67: 1169-1179.
17. Edgar BA, O'Farrell PH (1989) Genetic control of cell division patterns in the Drosophila embryo. Cell 57: 177-187.
18. Falkenburg D, Dworniczak B, Faust DM, Bautz EK (1987) RNA polymerase II of Drosophila. Relation of its 140,000 Mr subunit to the beta subunit of Escherichia coli RNA polymerase. J Mol Biol 195: 929-937.
19. Knoblich JA, Sauer K, Jones L, Richardson H, Saint R, et al. (1994) Cyclin E controls S phase progression and its down-regulation during Drosophila embryogenesis is required for the arrest of cell proliferation. Cell 77: 107-120.
20. Keyomarsi K, Tucker SL, Buchholz TA, Callister M, Ding Y, et al. (2002) Cyclin E and survival in patients with breast cancer. N Engl J Med 347: 1566-1575.
21. Biggs J, Searles LL, Greenleaf AL (1985) Structure of the eukaryotic transcription apparatus: features of the gene for the largest subunit of Drosophila RNA polymerase II. Cell 42: 611-621.
22. Henderson DS, Bailey DA, Sinclair DA, Grigliatti TA (1987) Isolation and characterization of second chromosome mutagen-sensitive mutations in Drosophila melanogaster. Mutat Res 177: 83-93.
23. Bravo R, Frank R, Blundell PA, Macdonald-Bravo H (1987) Cyclin/PCNA is the auxiliary protein of DNA polymerase-delta. Nature 326: 515-517.
24. Henderson DS, Banga SS, Grigliatti TA, Boyd JB (1994) Mutagen sensitivity and suppression of position-effect variegation result from mutations in mus209, the Drosophila gene encoding PCNA. Embo J 13: 1450-1459.
25. Bjorklund M, Taipale M, Varjosalo M, Saharinen J, Lahdenpera J, et al. (2006) Identification of pathways regulating cell size and cell-cycle progression by RNAi. Nature 439: 1009-1013.
26. Zhao K, Hart CM, Laemmli UK (1995) Visualization of chromosomal domains with boundary element-associated factor BEAF-32. Cell 81: 879-889.
27. Yamaguchi M, Yoshida H, Hirose F, Inoue YH, Hayashi Y, et al. (2001) Ectopic expression of BEAF32A in the Drosophila eye imaginal disc inhibits differentiation of photoreceptor cells and induces apoptosis. Chromosoma 110: 313-321.
28. Chiara MD, Champion-Arnaud P, Buvoli M, Nadal-Ginard B, Reed R (1994) Specific protein-protein interactions between the essential mammalian spliceosome-associated proteins SAP 61 and SAP 114. Proc Natl Acad Sci U S A 91: 6403-6407.
29. Meyer V, Oliver B, Pauli D (1998) Multiple developmental requirements of noisette, the Drosophila homolog of the U2 snRNP-associated polypeptide SP3a60. Mol Cell Biol 18: 1835-1843.
30. Bowman GD, O'Donnell M, Kuriyan J (2004) Structural analysis of a eukaryotic sliding DNA clamp-clamp loader complex. Nature 429: 724-730.
31. Dobie KW, Kennedy CD, Velasco VM, McGrath TL, Weko J, et al. (2001) Identification of chromosome inheritance modifiers in Drosophila melanogaster. Genetics 157: 1623-1637.

32. Chen X, Li Q, Fischer JA (2000) Genetic analysis of the Drosophila DNAprim gene. The function of the 60-kd primase subunit of DNA polymerase opposes the fat facets signaling pathway in the developing eye. Genetics 156: 1787-1795.
33. Duronio RJ, Bonnette PC, O'Farrell PH (1998) Mutations of the Drosophila dDP, dE2F, and cyclin E genes reveal distinct roles for the E2F-DP transcription factor and cyclin E during the G1-S transition. Mol Cell Biol 18: 141-151.
34. Dimova DK, Stevaux O, Frolov MV, Dyson NJ (2003) Cell cycle-dependent and cell cycle-independent control of transcription by the Drosophila E2F/RB pathway. Genes Dev 17: 2308-2320.
35. Wang Z, Lin H (2005) The division of Drosophila germline stem cells and their precursors requires a specific cyclin. Curr Biol 15: 328-333.
36. Munch-Petersen B, Piskur J, Sondergaard L (1998) Four deoxynucleoside kinase activities from Drosophila melanogaster are contained within a single monomeric enzyme, a new multifunctional deoxynucleoside kinase. J Biol Chem 273: 3926-3931.
37. Saada A, Shaag A, Mandel H, Nevo Y, Eriksson S, et al. (2001) Mutant mitochondrial thymidine kinase in mitochondrial DNA depletion myopathy. Nat Genet 29: 342-344.
38. Hari KL, Cook KR, Karpen GH (2001) The Drosophila Su(var)2-10 locus regulates chromosome structure and function and encodes a member of the PIAS protein family. Genes Dev 15: 1334-1348.
39. Liu B, Liao J, Rao X, Kushner SA, Chung CD, et al. (1998) Inhibition of Stat1-mediated gene activation by PIAS1. Proc Natl Acad Sci U S A 95: 10626-10631.
40. Kahyo T, Nishida T, Yasuda H (2001) Involvement of PIAS1 in the sumoylation of tumor suppressor p53. Mol Cell 8: 713-718.
41. Takata K, Yoshida H, Yamaguchi M, Sakaguchi K (2004) Drosophila damaged DNA-binding protein 1 is an essential factor for development. Genetics 168: 855-865.
42. Keeney S, Eker AP, Brody T, Vermeulen W, Bootsma D, et al. (1994) Correction of the DNA repair defect in xeroderma pigmentosum group E by injection of a DNA damage-binding protein. Proc Natl Acad Sci U S A 91: 4053-4056.
43. Angers S, Li T, Yi X, MacCoss MJ, Moon RT, et al. (2006) Molecular architecture and assembly of the DDB1-CUL4A ubiquitin ligase machinery. Nature 443: 590-593.
44. Mitsis PG, Kowalczykowski SC, Lehman IR (1993) A single-stranded DNA binding protein from Drosophila melanogaster: characterization of the heterotrimeric protein and its interaction with single-stranded DNA. Biochemistry 32: 5257-5266.
45. Erdile LF, Heyer WD, Kolodner R, Kelly TJ (1991) Characterization of a cDNA encoding the 70-kDa single-stranded DNA-binding subunit of human replication protein A and the role of the protein in DNA replication. J Biol Chem 266: 12090-12098.
46. Wang Y, Putnam CD, Kane MF, Zhang W, Edelmann L, et al. (2005) Mutation in Rpa1 results in defective DNA double-strand break repair, chromosomal instability and cancer in mice. Nat Genet 37: 750-755.
47. Shobuike T, Sugano S, Yamashita T, Ikeda H (1995) Characterization of cDNA encoding mouse homolog of fission yeast dhp1+ gene: structural and functional conservation. Nucleic Acids Res 23: 357-361.
48. West S, Gromak N, Proudfoot NJ (2004) Human 5' --> 3' exonuclease Xrn2 promotes transcription termination at co-transcriptional cleavage sites. Nature 432: 522-525.
49. Ishiai M, Dean FB, Okumura K, Abe M, Moon KY, et al. (1997) Isolation of human and fission yeast homologues of the budding yeast origin recognition complex subunit ORC5: human homologue (ORC5L) maps to 7q22. Genomics 46: 294-298.
50. Pflumm MF, Botchan MR (2001) Orc mutants arrest in metaphase with abnormally condensed chromosomes. Development 128: 1697-1707.

51. Ghabrial A, Ray RP, Schupbach T (1998) okra and spindle-B encode components of the RAD52 DNA repair pathway and affect meiosis and patterning in Drosophila oogenesis. Genes Dev 12: 2711-2723.
52. Matsuda M, Miyagawa K, Takahashi M, Fukuda T, Kataoka T, et al. (1999) Mutations in the RAD54 recombination gene in primary cancers. Oncogene 18: 3427-3430.
53. Qi H, Rath U, Wang D, Xu YZ, Ding Y, et al. (2004) Megator, an essential coiled-coil protein that localizes to the putative spindle matrix during mitosis in Drosophila. Mol Biol Cell 15: 4854-4865.
54. Zimowska G, Paddy MR (2002) Structures and dynamics of Drosophila Tpr inconsistent with a static, filamentous structure. Exp Cell Res 276: 223-232.
55. Raffa GD, Cenci G, Siriaco G, Goldberg ML, Gatti M (2005) The putative Drosophila transcription factor woc is required to prevent telomeric fusions. Mol Cell 20: 821-831.
56. Xiao S, Nalabolu SR, Aster JC, Ma J, Abruzzo L, et al. (1998) FGFR1 is fused with a novel zinc-finger gene, ZNF198, in the t(8;13) leukaemia/lymphoma syndrome. Nat Genet 18: 84-87.
57. Singh A, Chan J, Chern JJ, Choi KW (2005) Genetic interaction of Lobe with its modifiers in dorsoventral patterning and growth of the Drosophila eye. Genetics 171: 169-183.
58. Tarpey PS, Raymond FL, O'Meara S, Edkins S, Teague J, et al. (2007) Mutations in CUL4B, which encodes a ubiquitin E3 ligase subunit, cause an X-linked mental retardation syndrome associated with aggressive outbursts, seizures, relative macrocephaly, central obesity, hypogonadism, pes cavus, and tremor. Am J Hum Genet 80: 345-352.
59. Weinzierl RO, Ruppert S, Dynlacht BD, Tanese N, Tjian R (1993) Cloning and expression of Drosophila TAFII60 and human TAFII70 reveal conserved interactions with other subunits of TFIID. Embo J 12: 5303-5309.
60. Sauer F, Wassarman DA, Rubin GM, Tjian R (1996) TAF(II)s mediate activation of transcription in the Drosophila embryo. Cell 87: 1271-1284.
61. Song Z, McCall K, Steller H (1997) DCP-1, a Drosophila cell death protease essential for development. Science 275: 536-540.
62. McKittrick E, Gafken PR, Ahmad K, Henikoff S (2004) Histone H3.3 is enriched in covalent modifications associated with active chromatin. Proc Natl Acad Sci U S A 101: 1525-1530.
63. Morrison DK, Murakami MS, Cleghon V (2000) Protein kinases and phosphatases in the Drosophila genome. J Cell Biol 150: F57-62.
64. Nakatsu Y, Asahina H, Citterio E, Rademakers S, Vermeulen W, et al. (2000) XAB2, a novel tetratricopeptide repeat protein involved in transcription-coupled DNA repair and transcription. J Biol Chem 275: 34931-34937.
65. Albert TK, Lemaire M, van Berkum NL, Gentz R, Collart MA, et al. (2000) Isolation and characterization of human orthologs of yeast CCR4-NOT complex subunits. Nucleic Acids Res 28: 809-817.
66. Wang TL, Diaz LA, Jr., Romans K, Bardelli A, Saha S, et al. (2004) Digital karyotyping identifies thymidylate synthase amplification as a mechanism of resistance to 5-fluorouracil in metastatic colorectal cancer patients. Proc Natl Acad Sci U S A 101: 3089-3094.
67. Lee JH, Skalnik DG (2005) CpG-binding protein (CXXC finger protein 1) is a component of the mammalian Set1 histone H3-Lys4 methyltransferase complex, the analogue of the yeast Set1/COMPASS complex. J Biol Chem 280: 41725-41731.
68. Migeon JC, Garfinkel MS, Edgar BA (1999) Cloning and characterization of peter pan, a novel Drosophila gene required for larval growth. Mol Biol Cell 10: 1733-1744.

69. Welch PJ, Marcusson EG, Li QX, Beger C, Kruger M, et al. (2000) Identification and validation of a gene involved in anchorage-independent cell growth control using a library of randomized hairpin ribozymes. Genomics 66: 274-283.
70. Gabellini D, D'Antona G, Moggio M, Prelle A, Zecca C, et al. (2006) Facioscapulohumeral muscular dystrophy in mice overexpressing FRG1. Nature 439: 973-977.
71. Shichijo S, Nakao M, Imai Y, Takasu H, Kawamoto M, et al. (1998) A gene encoding antigenic peptides of human squamous cell carcinoma recognized by cytotoxic T lymphocytes. J Exp Med 187: 277-288.
72. Sumara I, Vorlaufer E, Gieffers C, Peters BH, Peters JM (2000) Characterization of vertebrate cohesin complexes and their regulation in prophase. J Cell Biol 151: 749-762.
73. Musio A, Selicorni A, Focarelli ML, Gervasini C, Milani D, et al. (2006) X-linked Cornelia de Lange syndrome owing to SMC1L1 mutations. Nat Genet 38: 528-530.
74. Gradia S, Acharya S, Fishel R (1997) The human mismatch recognition complex hMSH2-hMSH6 functions as a novel molecular switch. Cell 91: 995-1005.
75. Rowley PT (2005) Inherited susceptibility to colorectal cancer. Annu Rev Med 56: 539-554.
76. Yamauchi M, Yamauchi N, Meuth M (1990) Molecular cloning of the human CTP synthetase gene by functional complementation with purified human metaphase chromosomes. Embo J 9: 2095-2099.
77. Wohlschlegel JA, Dwyer BT, Dhar SK, Cvetic C, Walter JC, et al. (2000) Inhibition of eukaryotic DNA replication by geminin binding to Cdt1. Science 290: 2309-2312.
78. Whittaker AJ, Royzman I, Orr-Weaver TL (2000) Drosophila double parked: a conserved, essential replication protein that colocalizes with the origin recognition complex and links DNA replication with mitosis and the down-regulation of S phase transcripts. Genes Dev 14: 1765-1776.
79. Parry DH, Hickson GR, O'Farrell PH (2003) Cyclin B destruction triggers changes in kinetochore behavior essential for successful anaphase. Curr Biol 13: 647-653.
80. Basu J, Bousbaa H, Logarinho E, Li Z, Williams BC, et al. (1999) Mutations in the essential spindle checkpoint gene bub1 cause chromosome missegregation and fail to block apoptosis in Drosophila. J Cell Biol 146: 13-28.
81. Bettencourt-Dias M, Giet R, Sinka R, Mazumdar A, Lock WG, et al. (2004) Genome-wide survey of protein kinases required for cell cycle progression. Nature 432: 980-987.
82. Musacchio A, Salmon ED (2007) The spindle-assembly checkpoint in space and time. Nat Rev Mol Cell Biol 8: 379-393.
83. Lopes CS, Sampaio P, Williams B, Goldberg M, Sunkel CE (2005) The Drosophila Bub3 protein is required for the mitotic checkpoint and for normal accumulation of cyclins during G2 and early stages of mitosis. J Cell Sci 118: 187-198.
84. Prokopenko SN, He Y, Lu Y, Bellen HJ (2000) Mutations affecting the development of the peripheral nervous system in Drosophila: a molecular screen for novel proteins. Genetics 156: 1691-1715.
85. Karess R (2005) Rod-Zw10-Zwilch: a key player in the spindle checkpoint. Trends Cell Biol 15: 386-392.
86. Wang Z, Cummins JM, Shen D, Cahill DP, Jallepalli PV, et al. (2004) Three classes of genes mutated in colorectal cancers with chromosomal instability. Cancer Res 64: 2998-3001.
87. Karess RE, Glover DM (1989) rough deal: a gene required for proper mitotic segregation in Drosophila. J Cell Biol 109: 2951-2961.
88. Zamore PD, Patton JG, Green MR (1992) Cloning and domain structure of the mammalian splicing factor U2AF. Nature 355: 609-614.

89. Kanaar R, Roche SE, Beall EL, Green MR, Rio DC (1993) The conserved pre-mRNA splicing factor U2AF from Drosophila: requirement for viability. Science 262: 569-573.
90. Berry LD, Gould KL (1997) Fission yeast dim1(+) encodes a functionally conserved polypeptide essential for mitosis. J Cell Biol 137: 1337-1354.
91. Vithana EN, Abu-Safieh L, Allen MJ, Carey A, Papaioannou M, et al. (2001) A human homolog of yeast pre-mRNA splicing gene, PRP31, underlies autosomal dominant retinitis pigmentosa on chromosome 19q13.4 (RP11). Mol Cell 8: 375-381.
92. Schittenhelm RB, Heeger S, Althoff F, Walter A, Heidmann S, et al. (2007) Spatial organization of a ubiquitous eukaryotic kinetochore protein network in Drosophila chromosomes. Chromosoma 116: 385-402.
93. Przewloka MR, Zhang W, Costa P, Archambault V, D'Avino PP, et al. (2007) Molecular analysis of core kinetochore composition and assembly in Drosophila melanogaster. PLoS ONE 2: e478.
94. DeLuca JG, Gall WE, Ciferri C, Cimini D, Musacchio A, et al. (2006) Kinetochore microtubule dynamics and attachment stability are regulated by Hec1. Cell 127: 969-982.
95. DeLuca JG, Dong Y, Hergert P, Strauss J, Hickey JM, et al. (2005) Hec1 and nuf2 are core components of the kinetochore outer plate essential for organizing microtubule attachment sites. Mol Biol Cell 16: 519-531.
96. Will CL, Schneider C, MacMillan AM, Katopodis NF, Neubauer G, et al. (2001) A novel U2 and U11/U12 snRNP protein that associates with the pre-mRNA branch site. Embo J 20: 4536-4546.
97. Lauber J, Fabrizio P, Teigelkamp S, Lane WS, Hartmann E, et al. (1996) The HeLa 200 kDa U5 snRNP-specific protein and its homologue in Saccharomyces cerevisiae are members of the DEXH-box protein family of putative RNA helicases. Embo J 15: 4001-4015.
98. Henikoff S, Ahmad K, Platero JS, van Steensel B (2000) Heterochromatic deposition of centromeric histone H3-like proteins. Proc Natl Acad Sci U S A 97: 716-721.
99. Blower MD, Karpen GH (2001) The role of Drosophila CID in kinetochore formation, cell-cycle progression and heterochromatin interactions. Nat Cell Biol 3: 730-739.
100. Torok T, Tick G, Alvarado M, Kiss I (1993) P-lacW insertional mutagenesis on the second chromosome of Drosophila melanogaster: isolation of lethals with different overgrowth phenotypes. Genetics 135: 71-80.
101. Roch F, Serras F, Cifuentes FJ, Corominas M, Alsina B, et al. (1998) Screening of larval/pupal P-element induced lethals on the second chromosome in Drosophila melanogaster: clonal analysis and morphology of imaginal discs. Mol Gen Genet 257: 103-112.
102. Ashery-Padan R, Ulitzur N, Arbel A, Goldberg M, Weiss AM, et al. (1997) Localization and posttranslational modifications of otefin, a protein required for vesicle attachment to chromatin, during Drosophila melanogaster development. Mol Cell Biol 17: 4114-4123.
103. D'Andrea RJ, Stratmann R, Lehner CF, John UP, Saint R (1993) The three rows gene of Drosophila melanogaster encodes a novel protein that is required for chromosome disjunction during mitosis. Mol Biol Cell 4: 1161-1174.
104. Philp AV, Axton JM, Saunders RD, Glover DM (1993) Mutations in the Drosophila melanogaster gene three rows permit aspects of mitosis to continue in the absence of chromatid segregation. J Cell Sci 106 (Pt 1): 87-98.
105. Herzig A, Lehner CF, Heidmann S (2002) Proteolytic cleavage of the THR subunit during anaphase limits Drosophila separase function. Genes Dev 16: 2443-2454.
106. Jacobs HW, Knoblich JA, Lehner CF (1998) Drosophila Cyclin B3 is required for female fertility and is dispensable for mitosis like Cyclin B. Genes Dev 12: 3741-3751.

107. Nagengast AA, Salz HK (2001) The Drosophila U2 snRNP protein U2A' has an essential function that is SNF/U2B" independent. Nucleic Acids Res 29: 3841-3847.
108. Hendrich B, Tweedie S (2003) The methyl-CpG binding domain and the evolving role of DNA methylation in animals. Trends Genet 19: 269-277.
109. Jager H, Herzig A, Lehner CF, Heidmann S (2001) Drosophila separase is required for sister chromatid separation and binds to PIM and THR. Genes Dev 15: 2572-2584.
110. Nasmyth K (2002) Segregating sister genomes: the molecular biology of chromosome separation. Science 297: 559-565.
111. Chestukhin A, Pfeffer C, Milligan S, DeCaprio JA, Pellman D (2003) Processing, localization, and requirement of human separase for normal anaphase progression. Proc Natl Acad Sci U S A 100: 4574-4579.
112. Rudner DZ, Kanaar R, Breger KS, Rio DC (1996) Mutations in the small subunit of the Drosophila U2AF splicing factor cause lethality and developmental defects. Proc Natl Acad Sci U S A 93: 10333-10337.
113. Dawson IA, Roth S, Artavanis-Tsakonas S (1995) The Drosophila cell cycle gene fizzy is required for normal degradation of cyclins A and B during mitosis and has homology to the CDC20 gene of Saccharomyces cerevisiae. J Cell Biol 129: 725-737.
114. Holzl H, Kapelari B, Kellermann J, Seemuller E, Sumegi M, et al. (2000) The regulatory complex of Drosophila melanogaster 26S proteasomes. Subunit composition and localization of a deubiquitylating enzyme. J Cell Biol 150: 119-130.
115. Bentley AM, Williams BC, Goldberg ML, Andres AJ (2002) Phenotypic characterization of Drosophila ida mutants: defining the role of APC5 in cell cycle progression. J Cell Sci 115: 949-961.
116. Grossberger R, Gieffers C, Zachariae W, Podtelejnikov AV, Schleiffer A, et al. (1999) Characterization of the DOC1/APC10 subunit of the yeast and the human anaphase-promoting complex. J Biol Chem 274: 14500-14507.
117. Lasko P (2000) The drosophila melanogaster genome: translation factors and RNA binding proteins. J Cell Biol 150: F51-56.
118. Miccoli L, Frouin I, Novac O, Di Paola D, Harper F, et al. (2005) The human stress-activated protein kin17 belongs to the multiprotein DNA replication complex and associates in vivo with mammalian replication origins. Mol Cell Biol 25: 3814-3830.
119. Williams BC, Riedy MF, Williams EV, Gatti M, Goldberg ML (1995) The Drosophila kinesin-like protein KLP3A is a midbody component required for central spindle assembly and initiation of cytokinesis. J Cell Biol 129: 709-723.
120. Page SL, Hawley RS (2005) The Drosophila meiotic mutant mei-352 is an allele of klp3A and reveals a role for a kinesin-like protein in crossover distribution. Genetics 170: 1797-1807.
121. Goshima G, Vale RD (2003) The roles of microtubule-based motor proteins in mitosis: comprehensive RNAi analysis in the Drosophila S2 cell line. J Cell Biol 162: 1003-1016.
122. Kwon M, Morales-Mulia S, Brust-Mascher I, Rogers GC, Sharp DJ, et al. (2004) The chromokinesin, KLP3A, dives mitotic spindle pole separation during prometaphase and anaphase and facilitates chromatid motility. Mol Biol Cell 15: 219-233.
123. Moyer SE, Lewis PW, Botchan MR (2006) Isolation of the Cdc45/Mcm2-7/GINS (CMG) complex, a candidate for the eukaryotic DNA replication fork helicase. Proc Natl Acad Sci U S A 103: 10236-10241.
124. Cortez D, Glick G, Elledge SJ (2004) Minichromosome maintenance proteins are direct targets of the ATM and ATR checkpoint kinases. Proc Natl Acad Sci U S A 101: 10078-10083.
125. Tsao CC, Geisen C, Abraham RT (2004) Interaction between human MCM7 and Rad17 proteins is required for replication checkpoint signaling. Embo J 23: 4660-4669.

126. Hirano T (2006) At the heart of the chromosome: SMC proteins in action. Nat Rev Mol Cell Biol 7: 311-322.
127. Deardorff MA, Kaur M, Yaeger D, Rampuria A, Korolev S, et al. (2007) Mutations in cohesin complex members SMC3 and SMC1A cause a mild variant of cornelia de Lange syndrome with predominant mental retardation. Am J Hum Genet 80: 485-494.
128. Steffensen S, Coelho PA, Cobbe N, Vass S, Costa M, et al. (2001) A role for Drosophila SMC4 in the resolution of sister chromatids in mitosis. Curr Biol 11: 295-307.
129. Yu J, Fleming SL, Williams B, Williams EV, Li Z, et al. (2004) Greatwall kinase: a nuclear protein required for proper chromosome condensation and mitotic progression in Drosophila. J Cell Biol 164: 487-492.
130. Somma MP, Fasulo B, Siriaco G, Cenci G (2003) Chromosome condensation defects in barren RNA-interfered Drosophila cells. Genetics 165: 1607-1611.
131. Bhat MA, Philp AV, Glover DM, Bellen HJ (1996) Chromatid segregation at anaphase requires the barren product, a novel chromosome-associated protein that interacts with Topoisomerase II. Cell 87: 1103-1114.
132. Coelho PA, Queiroz-Machado J, Sunkel CE (2003) Condensin-dependent localisation of topoisomerase II to an axial chromosomal structure is required for sister chromatid resolution during mitosis. J Cell Sci 116: 4763-4776.
133. Oliveira RA, Coelho PA, Sunkel CE (2005) The condensin I subunit Barren/CAP-H is essential for the structural integrity of centromeric heterochromatin during mitosis. Mol Cell Biol 25: 8971-8984.
134. Cobbe N, Savvidou E, Heck MM (2006) Diverse mitotic and interphase functions of condensins in Drosophila. Genetics 172: 991-1008.
135. Savvidou E, Cobbe N, Steffensen S, Cotterill S, Heck MM (2005) Drosophila CAP-D2 is required for condensin complex stability and resolution of sister chromatids. J Cell Sci 118: 2529-2543.
136. Dej KJ, Ahn C, Orr-Weaver TL (2004) Mutations in the Drosophila condensin subunit dCAP-G: defining the role of condensin for chromosome condensation in mitosis and gene expression in interphase. Genetics 168: 895-906.
137. Echard A, Hickson GR, Foley E, O'Farrell PH (2004) Terminal cytokinesis events uncovered after an RNAi screen. Curr Biol 14: 1685-1693.
138. Chang CJ, Goulding S, Earnshaw WC, Carmena M (2003) RNAi analysis reveals an unexpected role for topoisomerase II in chromosome arm congression to a metaphase plate. J Cell Sci 116: 4715-4726.
139. Loupart ML, Krause SA, Heck MS (2000) Aberrant replication timing induces defective chromosome condensation in Drosophila ORC2 mutants. Curr Biol 10: 1547-1556.
140. Mathews MB, Sonenberg N, Hershey JWB (2007) Translational Control in Biology and Medicine; Press CSHL, editor.
141. Milkereit P, Gadal O, Podtelejnikov A, Trumtel S, Gas N, et al. (2001) Maturation and intranuclear transport of pre-ribosomes requires Noc proteins. Cell 105: 499-509.
142. Nousiainen M, Sillje HH, Sauer G, Nigg EA, Korner R (2006) Phosphoproteome analysis of the human mitotic spindle. Proc Natl Acad Sci U S A 103: 5391-5396.
143. Kellogg DR, Oegema K, Raff J, Schneider K, Alberts BM (1995) CP60: a microtubule-associated protein that is localized to the centrosome in a cell cycle-specific manner. Mol Biol Cell 6: 1673-1684.
144. Butcher RD, Chodagam S, Basto R, Wakefield JG, Henderson DS, et al. (2004) The Drosophila centrosome-associated protein CP190 is essential for viability but not for cell division. J Cell Sci 117: 1191-1199.

145. Spradling AC, Stern D, Beaton A, Rhem EJ, Laverty T, et al. (1999) The Berkeley Drosophila Genome Project gene disruption project: Single P-element insertions mutating 25% of vital Drosophila genes. Genetics 153: 135-177.
146. Miyazaki S, Rasmussen S, Imatani A, Diella F, Sullivan DT, et al. (1999) Characterization of the Drosophila ortholog of mouse eIF-3p48/INT-6. Gene 233: 241-247.
147. Marchetti A, Buttitta F, Miyazaki S, Gallahan D, Smith GH, et al. (1995) Int-6, a highly conserved, widely expressed gene, is mutated by mouse mammary tumor virus in mammary preneoplasia. J Virol 69: 1932-1938.
148. Morris C, Jalinot P (2005) Silencing of human Int-6 impairs mitosis progression and inhibits cyclin B-Cdk1 activation. Oncogene 24: 1203-1211.
149. Shapiro RS, Anderson KV (2006) Drosophila Ik2, a member of the I kappa B kinase family, is required for mRNA localization during oogenesis. Development 133: 1467-1475.
150. Savino TM, Bastos R, Jansen E, Hernandez-Verdun D (1999) The nucleolar antigen Nop52, the human homologue of the yeast ribosomal RNA processing RRP1, is recruited at late stages of nucleologenesis. J Cell Sci 112 (Pt 12): 1889-1900.
151. Crawford NP, Qian X, Ziogas A, Papageorge AG, Boersma BJ, et al. (2007) Rrp1b, a new candidate susceptibility gene for breast cancer progression and metastasis. PLoS Genet 3: e214.
152. Strasser K, Masuda S, Mason P, Pfannstiel J, Oppizzi M, et al. (2002) TREX is a conserved complex coupling transcription with messenger RNA export. Nature 417: 304-308.
153. Rehwinkel J, Herold A, Gari K, Kocher T, Rode M, et al. (2004) Genome-wide analysis of mRNAs regulated by the THO complex in Drosophila melanogaster. Nat Struct Mol Biol 11: 558-566.
154. Cullen CF, Deak P, Glover DM, Ohkura H (1999) mini spindles: A gene encoding a conserved microtubule-associated protein required for the integrity of the mitotic spindle in Drosophila. J Cell Biol 146: 1005-1018.
155. Goshima G, Wollman R, Stuurman N, Scholey JM, Vale RD (2005) Length control of the metaphase spindle. Curr Biol 15: 1979-1988.
156. Gergely F, Draviam VM, Raff JW (2003) The ch-TOG/XMAP215 protein is essential for spindle pole organization in human somatic cells. Genes Dev 17: 336-341.
157. Wong J, Fang G (2006) HURP controls spindle dynamics to promote proper interkinetochore tension and efficient kinetochore capture. J Cell Biol 173: 879-891.
158. Koffa MD, Casanova CM, Santarella R, Kocher T, Wilm M, et al. (2006) HURP is part of a Ran-dependent complex involved in spindle formation. Curr Biol 16: 743-754.
159. Sillje HH, Nagel S, Korner R, Nigg EA (2006) HURP is a Ran-importin beta-regulated protein that stabilizes kinetochore microtubules in the vicinity of chromosomes. Curr Biol 16: 731-742.
160. Yang CP, Chen MS, Liaw GJ, Chen SF, Chou G, et al. (2005) Using Drosophila eye as a model system to characterize the function of mars gene in cell-cycle regulation. Exp Cell Res 307: 183-193.
161. Rogers SL, Rogers GC, Sharp DJ, Vale RD (2002) Drosophila EB1 is important for proper assembly, dynamics, and positioning of the mitotic spindle. J Cell Biol 158: 873-884.
162. White RJ (2005) RNA polymerases I and III, growth control and cancer. Nat Rev Mol Cell Biol 6: 69-78.
163. Rossi F, Moschetti R, Caizzi R, Corradini N, Dimitri P (2007) Cytogenetic and molecular characterization of heterochromatin gene models in Drosophila melanogaster. Genetics 175: 595-607.
164. Boudeau J, Miranda-Saavedra D, Barton GJ, Alessi DR (2006) Emerging roles of pseudokinases. Trends Cell Biol 16: 443-452.

165. Herceg Z, Hulla W, Gell D, Cuenin C, Lleonart M, et al. (2001) Disruption of Trrap causes early embryonic lethality and defects in cell cycle progression. Nat Genet 29: 206-211.
166. Wiese C, Zheng Y (2006) Microtubule nucleation: gamma-tubulin and beyond. J Cell Sci 119: 4143-4153.
167. Schnorrer F, Luschnig S, Koch I, Nusslein-Volhard C (2002) Gamma-tubulin37C and gamma-tubulin ring complex protein 75 are essential for bicoid RNA localization during drosophila oogenesis. Dev Cell 3: 685-696.
168. Verollet C, Colombie N, Daubon T, Bourbon HM, Wright M, et al. (2006) Drosophila melanogaster gamma-TuRC is dispensable for targeting gamma-tubulin to the centrosome and microtubule nucleation. J Cell Biol 172: 517-528.
169. Sunkel CE, Gomes R, Sampaio P, Perdigao J, Gonzalez C (1995) Gamma-tubulin is required for the structure and function of the microtubule organizing centre in Drosophila neuroblasts. Embo J 14: 28-36.
170. Mahoney NM, Goshima G, Douglass AD, Vale RD (2006) Making microtubules and mitotic spindles in cells without functional centrosomes. Curr Biol 16: 564-569.
171. Saunders RD, Avides MC, Howard T, Gonzalez C, Glover DM (1997) The Drosophila gene abnormal spindle encodes a novel microtubule-associated protein that associates with the polar regions of the mitotic spindle. J Cell Biol 137: 881-890.
172. do Carmo Avides M, Glover DM (1999) Abnormal spindle protein, Asp, and the integrity of mitotic centrosomal microtubule organizing centers. Science 283: 1733-1735.
173. Wakefield JG, Bonaccorsi S, Gatti M (2001) The drosophila protein asp is involved in microtubule organization during spindle formation and cytokinesis. J Cell Biol 153: 637-648.
174. Bond J, Roberts E, Mochida GH, Hampshire DJ, Scott S, et al. (2002) ASPM is a major determinant of cerebral cortical size. Nat Genet 32: 316-320.
175. Andersen JS, Wilkinson CJ, Mayor T, Mortensen P, Nigg EA, et al. (2003) Proteomic characterization of the human centrosome by protein correlation profiling. Nature 426: 570-574.
176. Dix CI, Raff JW (2007) Drosophila Spd-2 recruits PCM to the sperm centriole, but is dispensable for centriole duplication. Curr Biol 17: 1759-1764.
177. Giansanti MG, Bucciarelli E, Bonaccorsi S, Gatti M (2008) Drosophila SPD-2 is an essential centriole component required for PCM recruitment and astral-microtubule nucleation. Curr Biol 18: 303-309.
178. Basto R, Lau J, Vinogradova T, Gardiol A, Woods CG, et al. (2006) Flies without centrioles. Cell 125: 1375-1386.
179. Bond J, Roberts E, Springell K, Lizarraga SB, Scott S, et al. (2005) A centrosomal mechanism involving CDK5RAP2 and CENPJ controls brain size. Nat Genet 37: 353-355.
180. Bennett D, Szoor B, Gross S, Vereshchagina N, Alphey L (2003) Ectopic expression of inhibitors of protein phosphatase type 1 (PP1) can be used to analyze roles of PP1 in Drosophila development. Genetics 164: 235-245.
181. Megraw TL, Kao LR, Kaufman TC (2001) Zygotic development without functional mitotic centrosomes. Curr Biol 11: 116-120.
182. Takagi M, Sueishi M, Saiwaki T, Kametaka A, Yoneda Y (2001) A novel nucleolar protein, NIFK, interacts with the forkhead associated domain of Ki-67 antigen in mitosis. J Biol Chem 276: 25386-25391.
183. Gerdes J, Lemke H, Baisch H, Wacker HH, Schwab U, et al. (1984) Cell cycle analysis of a cell proliferation-associated human nuclear antigen defined by the monoclonal antibody Ki-67. J Immunol 133: 1710-1715.
184. Endow SA (2003) Kinesin motors as molecular machines. Bioessays 25: 1212-1219.

185. Heck MM, Pereira A, Pesavento P, Yannoni Y, Spradling AC, et al. (1993) The kinesin-like protein KLP61F is essential for mitosis in Drosophila. J Cell Biol 123: 665-679.
186. Blangy A, Lane HA, d'Herin P, Harper M, Kress M, et al. (1995) Phosphorylation by p34cdc2 regulates spindle association of human Eg5, a kinesin-related motor essential for bipolar spindle formation in vivo. Cell 83: 1159-1169.
187. Goshima G, Vale RD (2005) Cell cycle-dependent dynamics and regulation of mitotic kinesins in Drosophila S2 cells. Mol Biol Cell 16: 3896-3907.
188. Mayr MI, Hummer S, Bormann J, Gruner T, Adio S, et al. (2007) The human kinesin Kif18A is a motile microtubule depolymerase essential for chromosome congression. Curr Biol 17: 488-498.
189. Gandhi R, Bonaccorsi S, Wentworth D, Doxsey S, Gatti M, et al. (2004) The Drosophila kinesin-like protein KLP67A is essential for mitotic and male meiotic spindle assembly. Mol Biol Cell 15: 121-131.
190. Ira G, Pellicioli A, Balijja A, Wang X, Fiorani S, et al. (2004) DNA end resection, homologous recombination and DNA damage checkpoint activation require CDK1. Nature 431: 1011-1017.
191. Vagnarelli P, Earnshaw WC (2004) Chromosomal passengers: the four-dimensional regulation of mitotic events. Chromosoma 113: 211-222.
192. Resnick TD, Satinover DL, MacIsaac F, Stukenberg PT, Earnshaw WC, et al. (2006) INCENP and Aurora B promote meiotic sister chromatid cohesion through localization of the Shugoshin MEI-S332 in Drosophila. Dev Cell 11: 57-68.
193. Adams RR, Maiato H, Earnshaw WC, Carmena M (2001) Essential roles of Drosophila inner centromere protein (INCENP) and aurora B in histone H3 phosphorylation, metaphase chromosome alignment, kinetochore disjunction, and chromosome segregation. J Cell Biol 153: 865-880.
194. Hanson KK, Kelley AC, Bienz M (2005) Loss of Drosophila borealin causes polyploidy, delayed apoptosis and abnormal tissue development. Development 132: 4777-4787.
195. Eggert US, Kiger AA, Richter C, Perlman ZE, Perrimon N, et al. (2004) Parallel chemical genetic and genome-wide RNAi screens identify cytokinesis inhibitors and targets. PLoS Biol 2: e379.
196. Giet R, Glover DM (2001) Drosophila aurora B kinase is required for histone H3 phosphorylation and condensin recruitment during chromosome condensation and to organize the central spindle during cytokinesis. J Cell Biol 152: 669-682.
197. Mello JA, Almouzni G (2001) The ins and outs of nucleosome assembly. Curr Opin Genet Dev 11: 136-141.
198. Glover DM (2005) Polo kinase and progression through M phase in Drosophila: a perspective from the spindle poles. Oncogene 24: 230-237.
199. van Vugt MA, Medema RH (2005) Getting in and out of mitosis with Polo-like kinase-1. Oncogene 24: 2844-2859.
200. Manak JR, Mitiku N, Lipsick JS (2002) Mutation of the Drosophila homologue of the Myb protooncogene causes genomic instability. Proc Natl Acad Sci U S A 99: 7438-7443.
201. Fung SM, Ramsay G, Katzen AL (2002) Mutations in Drosophila myb lead to centrosome amplification and genomic instability. Development 129: 347-359.
202. Verni F, Somma MP, Gunsalus KC, Bonaccorsi S, Belloni G, et al. (2004) Feo, the Drosophila homolog of PRC1, is required for central-spindle formation and cytokinesis. Curr Biol 14: 1569-1575.
203. Jiang W, Jimenez G, Wells NJ, Hope TJ, Wahl GM, et al. (1998) PRC1: a human mitotic spindle-associated CDK substrate protein required for cytokinesis. Mol Cell 2: 877-885.

204. Glotzer M (2005) The molecular requirements for cytokinesis. Science 307: 1735-1739.
205. Somma MP, Fasulo B, Cenci G, Cundari E, Gatti M (2002) Molecular dissection of cytokinesis by RNA interference in Drosophila cultured cells. Mol Biol Cell 13: 2448-2460.
206. Rogers SL, Wiedemann U, Stuurman N, Vale RD (2003) Molecular requirements for actin-based lamella formation in Drosophila S2 cells. J Cell Biol 162: 1079-1088.
207. Adams RR, Tavares AA, Salzberg A, Bellen HJ, Glover DM (1998) pavarotti encodes a kinesin-like protein required to organize the central spindle and contractile ring for cytokinesis. Genes Dev 12: 1483-1494.
208. Prokopenko SN, Brumby A, O'Keefe L, Prior L, He Y, et al. (1999) A putative exchange factor for Rho1 GTPase is required for initiation of cytokinesis in Drosophila. Genes Dev 13: 2301-2314.
209. Lehner CF (1992) The pebble gene is required for cytokinesis in Drosophila. J Cell Sci 103 (Pt 4): 1021-1030.
210. Hime G, Saint R (1992) Zygotic expression of the pebble locus is required for cytokinesis during the postblastoderm mitoses of Drosophila. Development 114: 165-171.
211. Field CM, Alberts BM (1995) Anillin, a contractile ring protein that cycles from the nucleus to the cell cortex. J Cell Biol 131: 165-178.
212. Field CM, Coughlin M, Doberstein S, Marty T, Sullivan W (2005) Characterization of anillin mutants reveals essential roles in septin localization and plasma membrane integrity. Development 132: 2849-2860.
213. Oegema K, Savoian MS, Mitchison TJ, Field CM (2000) Functional analysis of a human homologue of the Drosophila actin binding protein anillin suggests a role in cytokinesis. J Cell Biol 150: 539-552.
214. Naim V, Imarisio S, Di Cunto F, Gatti M, Bonaccorsi S (2004) Drosophila citron kinase is required for the final steps of cytokinesis. Mol Biol Cell 15: 5053-5063.
215. Shandala T, Gregory SL, Dalton HE, Smallhorn M, Saint R (2004) Citron kinase is an essential effector of the Pbl-activated Rho signalling pathway in Drosophila melanogaster. Development 131: 5053-5063.
216. D'Avino PP, Savoian MS, Glover DM (2004) Mutations in sticky lead to defective organization of the contractile ring during cytokinesis and are enhanced by Rho and suppressed by Rac. J Cell Biol 166: 61-71.
217. Di Cunto F, Imarisio S, Hirsch E, Broccoli V, Bulfone A, et al. (2000) Defective neurogenesis in citron kinase knockout mice by altered cytokinesis and massive apoptosis. Neuron 28: 115-127.
218. Gunsalus KC, Bonaccorsi S, Williams E, Verni F, Gatti M, et al. (1995) Mutations in twinstar, a Drosophila gene encoding a cofilin/ADF homologue, result in defects in centrosome migration and cytokinesis. J Cell Biol 131: 1243-1259.
