# Supplementary material for: Identification of Drosophila Mitotic Genes by Combining Co-Expression Analysis and RNA Interference
Source: PLoS Genet. 2008 Jul 18;4(7):e1000126. doi: 10.1371/journal.pgen.1000126 (PMC2537813; doi:10.1371/journal.pgen.1000126)
Supplement: Table S7 — Comparison with the Goshima et al. (2007) screen. (0.04 MB PDF) [file pgen.1000126.s023.pdf]

Supplementary Table 7. A comparison between this screen and the screen performed by Goshima et al. (2007). Numbers and frequencies refer to the first 1000 genes of the consensus coexpression list (Table S1). Known genes are those listed in Table S3 and included in the first 1000 genes of the consensus coexpression list (Table S1). See supplementary text for explanation.

	Goshima et al. 2007			Present screen			both screens
Phenotypic class	# of detected genes	detected/ known (a)	# of novel genes	# of detected genes	detected/ known (b)	# of novel genes	undetected known genes (c)
chromosome breakage	3(d)	0/4	3	44	7/7	37	0
other mitotic phenotypes	71	31/75 (41%)	40	98(e)	68/96 (71%)	30	21

(a) # of known genes detected in the screen/ total known genes.

(b) # of known genes detected in the screen/ total known genes (in this case the known genes include those listed in Table S3 plus the novel genes uncovered by Goshima et al.

(c) # of known genes that were not detected in either screen.

(d) Goshima et al. identified 3 genes required to prevent chromosome breakage, because RNAi to these genes generated acentric fragments that were detected as misaligned chromosomes.

(e) this number includes the genes that were not detectable in the Goshima et al. screen.
