# Supplementary material for: Identification of Drosophila Mitotic Genes by Combining Co-Expression Analysis and RNA Interference
Source: PLoS Genet. 2008 Jul 18;4(7):e1000126. doi: 10.1371/journal.pgen.1000126 (PMC2537813; doi:10.1371/journal.pgen.1000126)
Supplement: Text S1 — Comparison with previous screens. (0.05 MB DOC) [file pgen.1000126.s024.doc]

## Comparison with previous screens

Several RNAi-based screens have been performed to identify *Drosophila* mitotic genes. Some of these screens were genome wide surveys aimed at detecting changes in cell and nuclear morphology [1-3], defects in cytokinesis [3,4] or in spindle and centrosome structure [5]. Other screens were designed to investigate the possible mitotic functions of selected gene groups, such as those encoding kinesins, actin-binding proteins, kinases or phosphatases [6-9].

Our screen shares certain features with both the genome-wide and the gene-class-specific approaches. We performed RNAi experiments on a limited number of genes, but these genes include about one half of the “mitotic genome,” making our screen in this sense comparable to large-scale screens. Moreover, we looked in detail at a very wide spectrum of mitotic phenotypes including abnormalities in chromosome structure and integrity. The screen most similar to our own is the recent genome-wide survey performed by Goshima and coworkers [5]. They conducted their RNAi screen in the presence of dsRNA to *Cdc27* (that encodes a subunit of the APC/C) to induce a partial metaphase block. This treatment resulted in an increase in metaphase figures, which facilitated examination of cells by automated microscopy to detect mitotic defects. The effects of dsRNAs causing mitotic abnormalities were subsequently reexamined in the absence of Cdc27 depletion to confirm the mitotic phenotype. In comparing our data with those of Goshima et al., we have taken into account only the 189 genes from their work whose inactivation results in a clear mitotic phenotype in both the presence and the absence of *Cdc27* dsRNA.

The screen of Goshima et al. was not designed to detect metaphase-arrest and cytokinesis failures and, as expected, they detected only two genes that elicit these phenotypes after RNAi. The automatic microscopic methods employed by these investigators could also in general not directly visualize chromosome aberrations. However, they were able to identify three dsRNAs that cause chromosome breakage because the acentric fragments generated by RNAi to these genes could be detected as misaligned chromosomes [5]. If we consider only the mitotic genes that were identified by both screens, Goshima et al. detected 31 known/expected genes and 40 novel genes among the first 1000 genes of our coexpression list. In our screen, we detected 58 known genes (counting as known both genes found prior to the work of Goshima et al. as well as the novel genes they found) and 30 completely novel genes (Supplementary Table 7). 21 known/expected mitotic genes were not detected in either screen (Supplementary Table 7). Excluding the genes required for chromosome integrity, together the two screens identified 105 mitotic genes in the first 1000 genes of the consensus coexpression list (Supplementary Table 1). 63% of these 105 genes were detected in the Goshima et al. screen and 83% in our screen. Thus, both screens were reasonably efficient, even if Goshima et al. used automated microscopy. However, it is likely that at least 20%of the mitotic genes included in the first 1000 genes of our coexpression list have remained undetected.

**Supplementary References**

1. Kiger AA, Baum B, Jones S, Jones MR, Coulson A, et al. (2003) A functional genomic analysis of cell morphology using RNA interference. J Biol 2: 27.

2. Boutros M, Kiger AA, Armknecht S, Kerr K, Hild M, et al. (2004) Genome-wide RNAi analysis of growth and viability in Drosophila cells. Science 303: 832-835.

3. Eggert US, Kiger AA, Richter C, Perlman ZE, Perrimon N, et al. (2004) Parallel chemical genetic and genome-wide RNAi screens identify cytokinesis inhibitors and targets. PLoS Biol 2: e379.

4. Echard A, Hickson GR, Foley E, O'Farrell PH (2004) Terminal cytokinesis events uncovered after an RNAi screen. Curr Biol 14: 1685-1693.

5. Goshima G, Wollman R, Goodwin SS, Zhang N, Scholey JM, et al. (2007) Genes required for mitotic spindle assembly in Drosophila S2 cells. Science 316: 417-421.

6. Goshima G, Vale RD (2003) The roles of microtubule-based motor proteins in mitosis: comprehensive RNAi analysis in the Drosophila S2 cell line. J Cell Biol 162: 1003-1016.

7. Rogers SL, Wiedemann U, Stuurman N, Vale RD (2003) Molecular requirements for actin-based lamella formation in Drosophila S2 cells. J Cell Biol 162: 1079-1088.

8. Bettencourt-Dias M, Giet R, Sinka R, Mazumdar A, Lock WG, et al. (2004) Genome-wide survey of protein kinases required for cell cycle progression. Nature 432: 980-987.

9. Chen F, Archambault V, Kar A, Lio P, D'Avino PP, et al. (2007) Multiple protein phosphatases are required for mitosis in Drosophila. Curr Biol 17: 293-303.
